# Improvements in micelle promoted DNA-encoded library synthesis by surfactant optimisation†

**DOI:** 10.1039/d5ob00864f

**Published:** 2025-06-13

**Authors:** Jake A. Odger, Matthew J. Anderson, Thomas P. Carton, Bao Nguyen, Kevin Foote, Michael J. Waring

**Affiliations:** a Cancer Research Horizons Newcastle Drug Discovery Group, Chemistry, Newcastle University Newcastle upon Tyne NE1 7RU UK mike.waring@ncl.ac.uk; b School of Chemistry, University of Leeds Woodhouse Lane Leeds LS2 9JT UK; c Pharmaron, West Hill Innovation Park Hertford Road Hoddesdon Hertfordshire EN11 9FH UK

## Abstract

DNA-encoded libraries are increasingly important in hit identification at the early stage of the drug discovery process. The approach relies on efficient methods for synthesis of drug-like compounds attached to coding DNA sequences. Many reactions employed for library synthesis are inefficient and result in significant DNA-damage, incomplete conversion and the formation of side products, which compromise the fidelity of the resulting library. We have developed a wide array of reactions that are promoted by the micelle-forming surfactant TPGS-750-M that address these issues and lead to improved efficiency. Here we demonstrate further improvements to key reactions Suzuki–Miyaura coupling, reductive amination and amide coupling by surfactant screening using principal component-based surfactant maps which lead to improved conversion for problematic substrates. This work demonstrates the utility of surfactant maps in reaction optimisation for DNA-encoded library synthesis and leads to further improvements in these important transformations.

## Abstract 2

A principal components map was used to optimise on-DNA Suzuki–Miyaura coupling, reductive amination and reverse amide coupling reactions.

## Graphical abstract

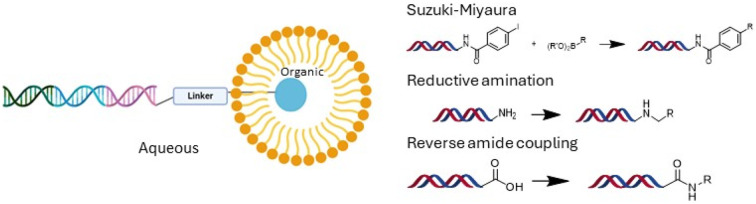

## Introduction

**Fig. 1:**
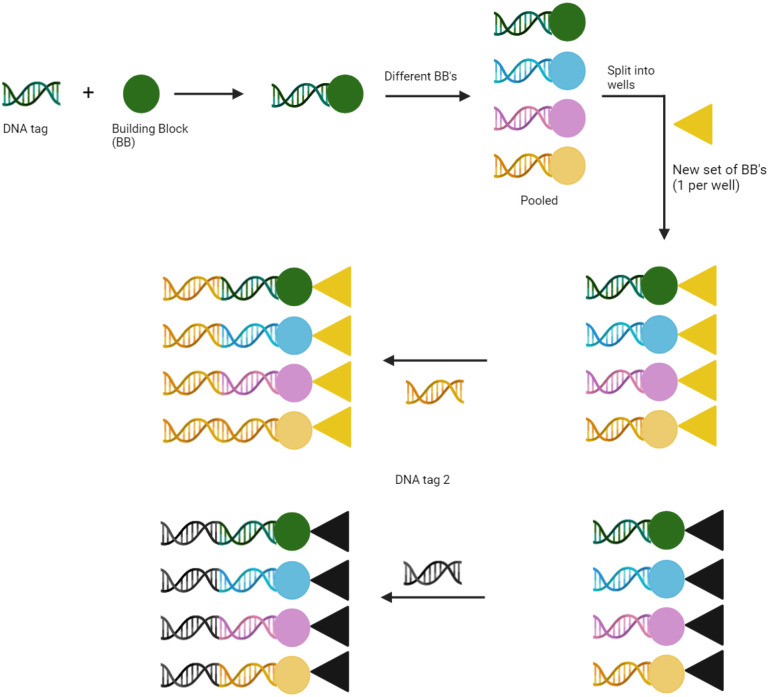
Combinatorial split-and-pool synthesis of DNA-encoded libraries.

DNA-encoded libraries (DELs) are an emerging technology in the field of medicinal chemistry for effective identification of hit compounds.^1–4^ DELs consist of a vast number of organic molecules, with each member covalently attached to a specific DNA sequence, which functions as a unique, amplifiable barcode. Libraries are commonly synthesised through the use of combinatorial split-and-pool methodology (Fig. 1). This technique couples the conjugation of chemical building blocks with the enzymatic ligation of an encoding DNA oligomer, whereby each DNA sequence is specific to the chemical monomer it encodes.^5^ This approach allows for the generation of large libraries covering a broad range of chemical space. In fact, DELs can be synthesised with in excess of 10^9^ library members; libraries of this size would be practically impossible to produce using traditional chemical synthesis. DELs can be screened as a mixture against an immobilised protein of interest, and polymerase chain reaction (PCR) used to amplify the DNA barcodes of any eluted binders. Next-generation sequencing (NGS) is then used to read the DNA, identifying sequences of target binders and enabling subsequent elucidation of corresponding small molecules. Hit compounds are then typically validated *via* off-DNA synthesis and testing in orthogonal assays.

Effective hit identification through DEL technology requires high-quality libraries; *i.e.* containing lead-like compounds with the majority of the library correctly encoded. This must be achieved through the employment of efficient on-DNA chemical transformations in library synthesis.^6^ However, diversity of the libraries is often limited, owing to the challenges associated with some on-DNA synthetic methods. Conventionally, DEL reactions should be carried out in aqueous media at high dilutions, avoid damaging the integrity of the DNA barcode, proceed with high yields and afford little to no side products.^7^

Surfactants have been applied in organic chemistry since the 1970s to facilitate reactions in aqueous media.^8^ Micelle-forming surfactants can aid solubilisation and increase the effective concentration of organic reagents in an aqueous solution.^9^ Interest in this field has surged in recent years due to the desire to reduce the use of organic solvents.^10^

**Fig. 2:**
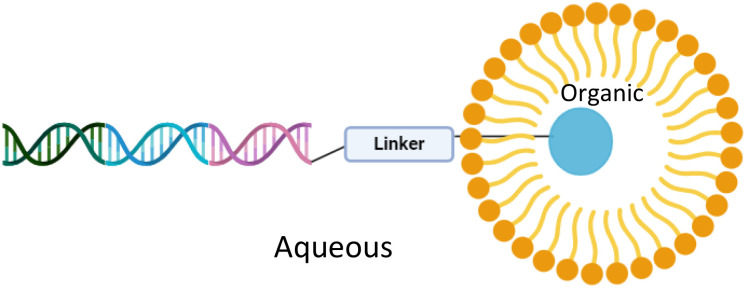
Representation of micellar catalysis for DEL reactions.

The use of surfactants in DEL syntheses has been shown to be advantageous. It is theorised that micellar structures localise organic reagents within the hydrophobic core of the micelles, whilst the DNA barcode remains in the aqueous compartment (Fig. 2).^11^ We have applied TPGS-750-M,^12^ a second-generation designer surfactant developed by Lipshutz *et al.*, to various reactions on DNA, including Suzuki–Miyaura, Heck, Sonogashira and Buchwald–Hartwig cross-coupling; amide coupling; transfer hydrogenation and hydrogenolysis; and reductive amination reactions.^11,13–18^ Brunschweiger *et al.* developed sulfonic acid-containing, micelle-forming block copolymers to perform Povarov, Groebke–Blackburn–Bienaymé and Biginelli reactions.^19^ More recently, decarboxylative photoredox arylations were performed on DNA using cationic surfactant molecules to catalyse the reaction.^20^

Despite the significant application of micellar catalysis to DEL synthesis, little work has been done to evaluate the use of different surfactant molecules for these reactions. Almost all micellar chemistry on DNA has been limited to the commercially available TPGS-750-M or expensive specialist surfactants. This study aims to compare alternative readily-accessible surfactants to perform these reactions with the aim of improving the efficiencies achieved with TPGS-750-M. Nguyen *et al.* have recently developed a 3D surfactant map which employs the NIPALS principal component analysis algorithm, based on 22 experimental and computational descriptors of the surfactant molecules (*e.g.* hydrophobic and hydrophilic fragments, number of C=C bonds, and Hirshfeld charges), their interactions with water (*e.g.* SASA, Δ*G*_solv_) and their micellar and emulsion properties (*e.g.* CMC, aggregation number, zeta potential, contact angle and HLB).^21^ Importantly, the designer surfactants occupy a relatively small, but central portion of surfactant space. The map has been successfully used to optimise surfactants for specific surfactant enabled reactions, therefore it was theorised that this could be applied to micellar reactions on DNA.

**Fig. 3:**
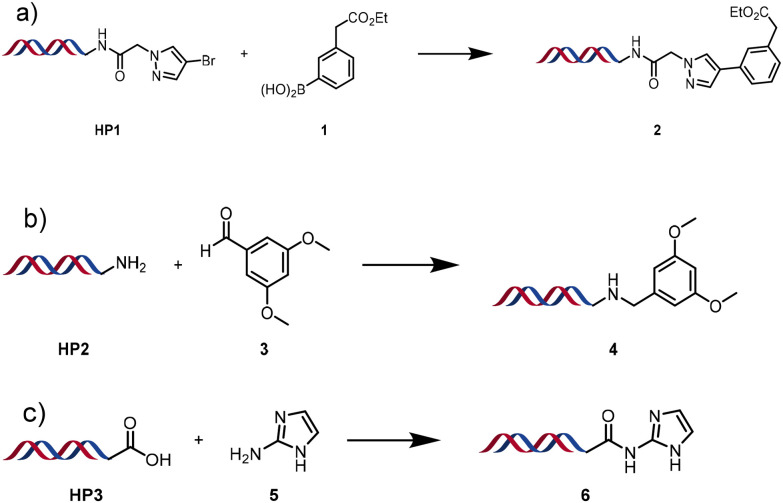
Selected reactions for this work: (a) Suzuki–Miyaura cross coupling of DNA-tagged bromo pyrazole HP1 with boronic acid 1, conditions: Pd(dtbpf)Cl_2_ (7.3 mM), boronate (500 mM), K_3_PO_4_ (530 mM), surfactant (2.0 wt%), 15% THF, 60 °C, 5 h (b) reductive amination of DNA-tagged amine HP2 with aldehyde 3, conditions: borate buffer (350 mM, pH = 10.8) aldehyde (400 mM in MeCN, 2.4 μmol) surfactant (1.2 wt%), rt, 1.5 h. Then: NaBH_4_ (440 mM in H_2_O/MeCN, 1 : 1 v/v, 2.2 μmol), rt, 16 h (c) “reverse” amide coupling of DNA-tagged acid HP3 with amine 5, conditions: amine (0.5 M), HOAt (0.5 M), lutidine (1.5 M), DIC (0.5 M), surfactant (4.5 wt%), 45 °C, 3 h.

Three reactions that we have previously optimised using TPGS-750-M as the surfactant were chosen for this study: Suzuki–Miyaura cross-coupling (Fig. 3a), reductive amination (Fig. 3b) and “reverse” amide coupling (*i.e.* DNA-tagged carboxylic acid, Fig. 3c). These reactions are well-established and offer good to excellent conversions for a wide range of substrates. The preliminary reactions used substrates that were selected based on their being relatively low-yielding when TPGS-750-M was employed.

## Results and discussion

### Suzuki–Miyaura cross-coupling

**Fig. 4:**
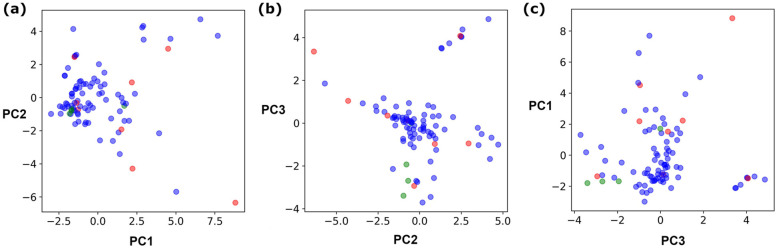
3D Principal component surfactant map showing the surfactants selected for the first screen (red dots) and second screen (green dots) and how they represent the surfactant space of all surfactants (blue dots); (a) PC1 *vs.* PC2; (b) PC2 *vs.* PC3; (c) PC1 *vs.* PC3.

Coupling of a DNA-tagged bromopyrazole (HP1) with boronic acid 1 was chosen for the initial Suzuki–Miyaura cross-coupling reaction (previous conversion 55%).^11^ Seven commercially available surfactants were applied to this reaction alongside TPGS-750-M (Table 1). This set of 8 surfactants was chosen to represent all areas of the surfactant map using the previously reported Python code to maximise the area covered by the selected surfactants within the 3D map (Fig. 4, with projection onto 3 × 2D planes to show the chemical space covered by the selection).^21^ The designer surfactant TPGS-750-M was used as the origin point for the algorithm, due to its known performance in our reactions. The principal component PC3 is mainly influenced by the nominal charge of the surfactant molecule. The most significant contributors to PC1 are the volume, surface area and flexibility of the hydrophobic and hydrophilic fragments of each surfactant. PC2 was mainly derived from a combination of contact angles, zeta potential and volume/area/flexibility properties of the hydrophobic fragment.

**Table 1:** Initial surfactant scope for Suzuki–Miyaura coupling of DNA-tagged bromopyrazole HP1 with boronic acid 1. Conditions: HP1 (1 nmol), 1 (500 mM), Pd(dtbpf)Cl_2_ (7.3 mM), K_3_PO_4_ (530 mM), 2% surfactant, 15% THF, 60 °C, 5 h

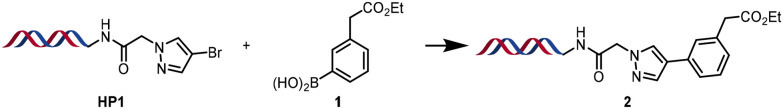		
Entry	Surfactant	Product^a^ (%)
1	None	56
2	TPGS-750-M	62
3	PEG_5_C_12_	40
4	Tween 65	29
5	Brij 700	41
6	Brij S20	26
7	Triton-X-405	59
8	TTAC	62
9	Sulfobetaine-16	70

a Desired product observed as the product with hydrolysis of the ester.

Firstly, replacing the surfactant from the reaction with just water resulted in the expected slight decrease in conversion to desired product compared with the use of TPGS-750-M (Table 1, entries 1 and 2). Conversions are determined by taking the integral of the peak areas in the total ion chromatogram, desired product conversion expressed as a percentage of all species present.

Several surfactants (PEG_5_C_12_, Tween 65, Brij 700 and Brij S20) led to a significant decrease in conversion (26–41%, Table 1, entries 3–6). The use of Triton-X-405 and TTAC yielded comparable conversions to TPGS-750-M (59% and 62% conversion observed for Triton-X-405 and TTAC respectively, Table 1, entries 7 and 8). Finally, utilising sulfobetaine-16 resulted in an improvement in conversion to 70% (Table 1, entry 9).

From these results, based on the surfactant map, a further subset of surfactants was assessed (Table 2). From this subset of four more surfactants, polysorbate 60 afforded a significantly reduced conversion (30%) compared with TPGS-750-M (Table 2, entry 1). Both (lauryldimethylammonio)acetate and dodecylamino-3-propane-1-sulfonate (DAPS) resulted in similar conversions to desired product (65% and 60%, respectively) compared with TPGS-750-M (Table 2, entries 2 and 3). Employing 4-dodecylbenzene sulfonic acid as the surfactant in this reaction led to the highest conversion across all surfactants tested (75%, Table 2, entry 4).

**Table 2:** Follow up surfactant scope of the Suzuki–Miyaura coupling reaction. Conditions: HP1 (1 nmol), 1 (500 mM), Pd(dtbpf)Cl_2_ (7.3 mM), K_3_PO_4_ (530 mM), 2% surfactant, 15% THF, 60 °C, 5 h

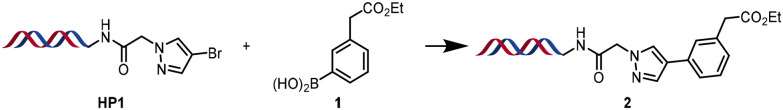		
Entry	Surfactant	Product (%)
1	Polysorbate 60	30
2	(Lauryldimethylammonio)acetate	65
3	DAPS	60
4	4-Dodecylbenzenesulfonic acid	75

The performance of the three best-performing surfactants (sulfobetaine-16, (lauryldimethylammonio)acetate and 4-dodecylbenzenesulfonic acid) was assessed against a wider range of substrates. DNA-tagged aryl iodide headpiece (HP4) was reacted with ten boronic acid/ester substrates (Table 3). All three surfactants afforded an overall improvement in reaction outcome across the set of substrates compared to TPGS-750-M. 4-Dodecylbenzenesulfonic acid offered the highest average conversion across all ten substrates. Therefore, 4-dodecylbenzenesulfonic acid was profiled across a wider range of boronic acid/esters (Table 4). 13 boronate substrates with varying chemical moieties and reactivities were chosen. Only one substrate gave rise to a reduced conversion with 4-dodecylbenzenesulfonic acid relative to TPGS-750-M (Table 4, entry 1). For 7 of the substrates, the conversion to product using TPGS-750-M and 4-dodecylbenzenesulfonic acid was comparable (within 5%, Table 4, entries 2–8), the remaining five substrates exhibited improved conversion (Table 4, entries 8–13). Hence, across all the substrates tested for the Suzuki–Miyaura reaction, 4-dodecylbenzenesulfonic acid as the surfactant offers significant improvement. Of the 24 substrates tested, 11 were found to be improved by the use of 4-dodecylbenzenesulfonic acid, 12 yielded the same conversion (within 5%) for both, whilst TPGS-750-M was preferred for only one substrate. The overall average conversion across all boronate substrates tested was improved from 72% with TPGS-750-M to 79% with 4-dodecylbenzenesulfonic acid.

**Table 3:** Substrate scope for the Suzuki–Miyaura reaction of DNA-tagged aryl iodide HP4 with various boronate substrates with TPGS-750-M, (lauryldimethylammonio)acetate, 4-dodecylbenzenesulfonic acid and sulfobetaine-16 as the surfactants. Conditions: HP4 (1 nmol), boronate (500 mM), Pd(dtbpf)Cl_2_ (7.3 mM), K_3_PO_4_ (530 mM), 2% surfactant, 15% THF, 60 °C, 5 h

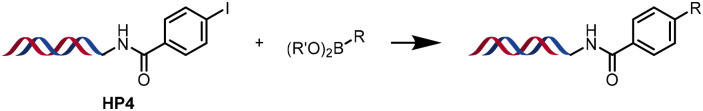					
Entry	Substrate	Product (%)			
		TPGS-750-M	(Lauryldimethyl-ammonio)acetate	4-Dodecylbenzene-sulfonic acid	Sulfobetaine-16
1	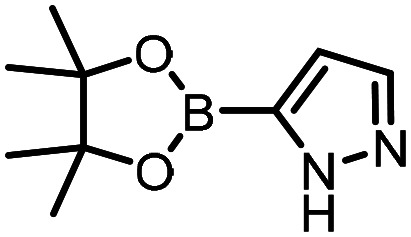	100	100	100	100
2	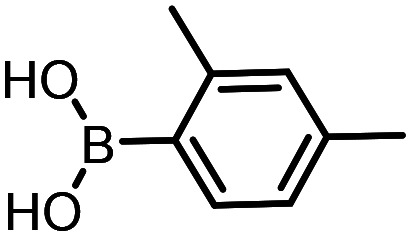	81	69	90	73
3	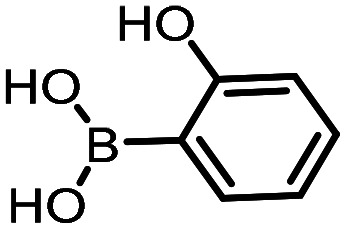	75	87	71	51
4	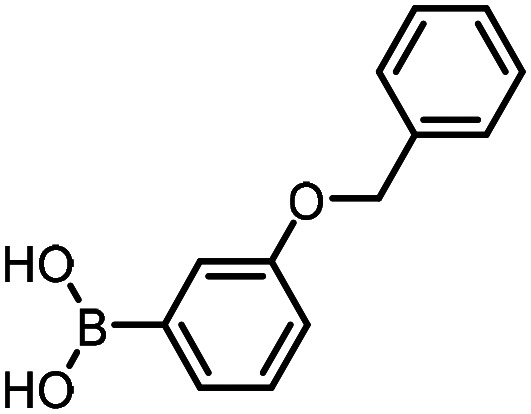	89	86	96	90
5	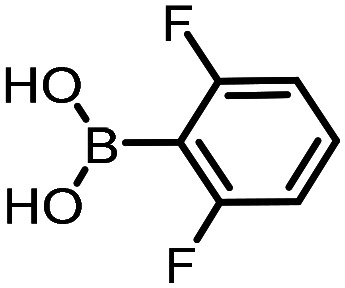	51	69	70	71
6	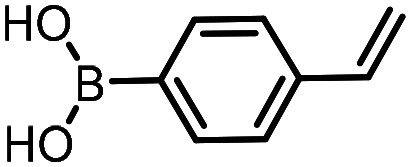	42	69	50	71
7	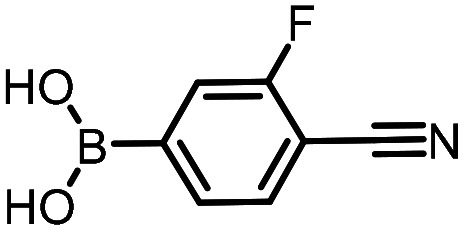	50	52	51	55
8	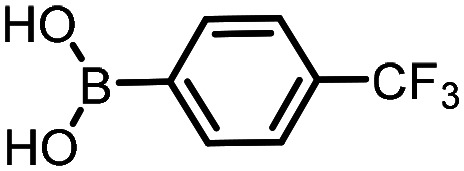	65	76	82	74
9	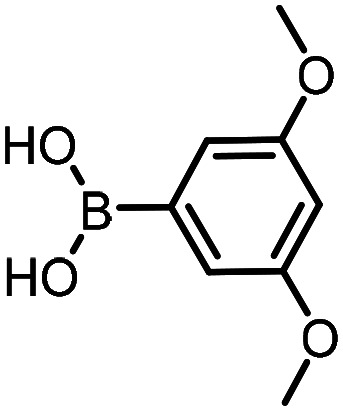	82	92	86	89
10	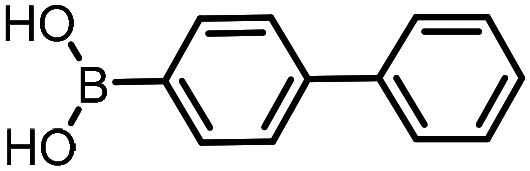	83	64	79	65
11	Average	72	76	78	74

**Table 4:** Extended substrate scope for the Suzuki–Miyaura reaction of DNA-tagged aryl iodide HP4 with a range of boronate substrate, comparing the use of 4-dodecylbenzenesulfonic acid as the surfactant with TPGS-750-M. Conditions: HP4 (1 nmol), boronate (500 mM), Pd(dtbpf)Cl_2_ (7.3 mM), K_3_PO_4_ (530 mM), 2% surfactant, 15% THF, 60 °C, 5 h

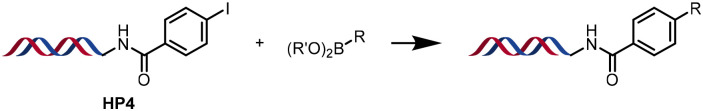			
Entry	Substrate	Product (%)	
		TPGS-750-M	4-Dodecylbenzenesulfonic acid
1	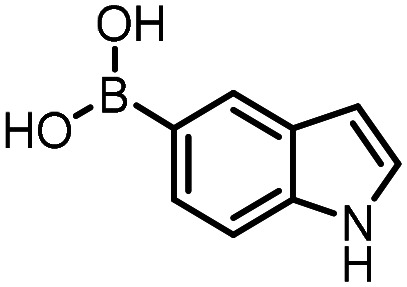	98	74
2	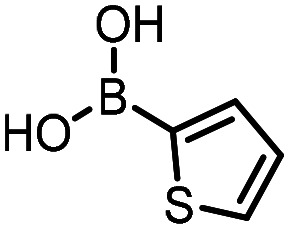	0	0
3	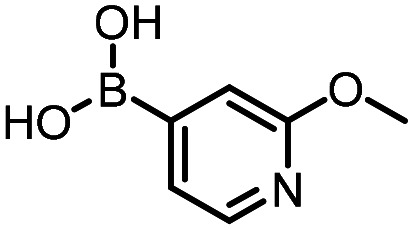	82	80
4	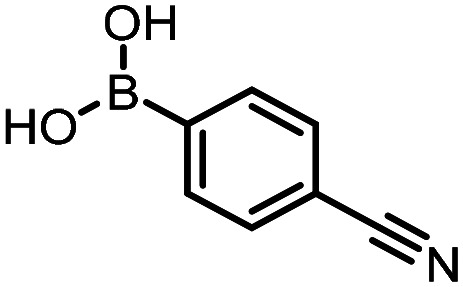	78^a^	80^a^
5	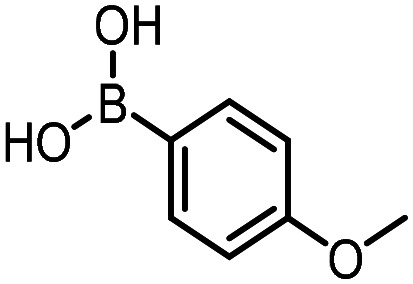	93	94
6	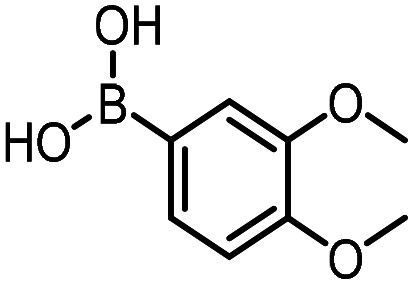	97	98
7	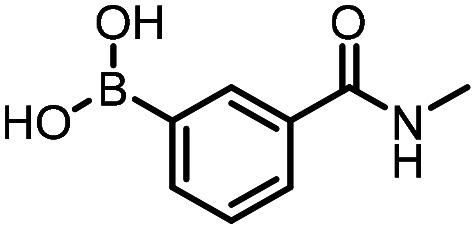	100	100
8	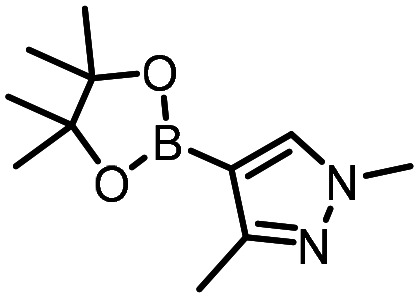	100	100
9	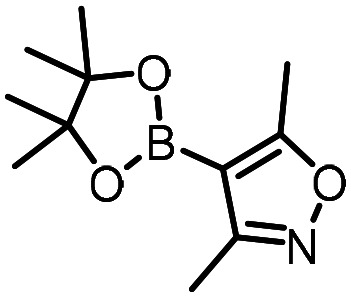	73	100
10	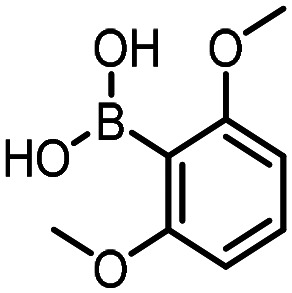	79	87
11	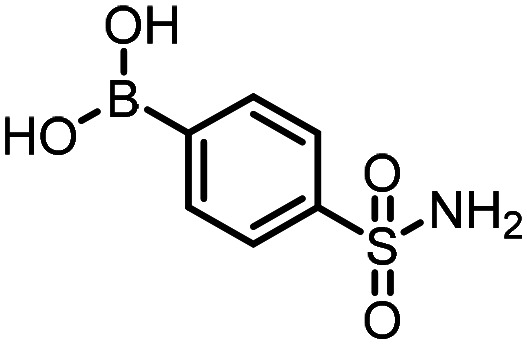	80	93
12	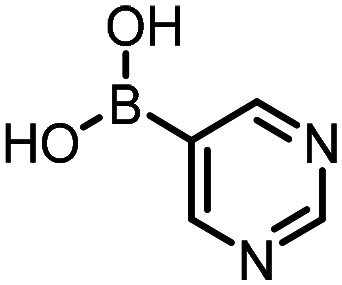	83	91
13	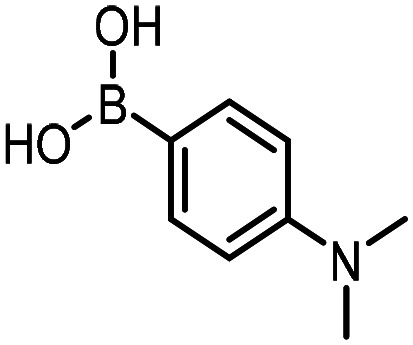	87	93
14	Average	81	84

a Indicates the sum of desired product and the product of nitrile hydrolysis.

### Reductive amination

The same approach was taken for the reductive amination reaction. The primary reaction tested was that of a DNA-tagged amine (HP2) with aldehyde 3. The same initial set of surfactants used previously were screened (Table 5). Previous work has proven the effectiveness of the use of surfactant in this reaction, which provides significant improvement upon conversions to desired product.^18^ The TPGS-750-M promoted reaction was somewhat better than anticipated (85%, Table 5, entry 1, compared to 77% observed previously). Two surfactants, PEG_5_C_12_ and Tween 65, gave reduced conversion (74% and 45% respectively, entries 2 and 3), Brij 700 and Brij S20, were comparable (86% and 91% respectively, Table 5, entries 4 and 5). The remainder of the surfactants all showed significantly improved results, with sulfobetaine-16 yielding 97% and Triton-X-405 and TTAC affording 100% conversion to desired product (Table 5, entries 6–8). Therefore, we determined that a follow up surfactant screen was not necessary. Instead, the three best-performing surfactants (sulfobetaine-16, Triton-X-405 and TTAC) were assessed for their wider limited substrate scope. Four diverse aldehydes were selected due to their previously observed moderate conversions to desired product (Table 6). Both Triton-X-405 and sulfobetaine-16 in these reactions were detrimental to conversion for all four aldehydes compared to TPGS-750-M. However, TTAC offered significant improvement in desired product conversion for every substrate, as well as a marked increase in the average conversion. Therefore, TTAC was selected as the optimal surfactant for the reductive amination and was hence applied to an extended selection of 20 aldehydes (Table 7). 4 aldehydes afforded reduced conversion to desired product with TTAC *versus* TPGS-750-M (Table 7, entries 1–4). A further 4 were comparable (within 5% conversion, Table 7, entries 5–8). However, the remaining 12 substrates provided increased conversions to desired product with TTAC, 9 of which gave an improvement of >10% conversion. In total, 16 of 25 total aldehydes explored exhibited an improved conversion to desired product upon changing the surfactant in the reaction from TPGS-750-M to TTAC. 5 of the aldehydes tested performed comparably (conversions within 5%), whilst only 4 aldehydes afforded reduced conversions to desired product following the implementation of TTAC. The overall average conversion across all aldehyde substrates was improved from 67% using TPGS-750-M to 75% using TTAC as the surfactant.

**Table 5:** Initial surfactant scope for the reductive amination reaction of DNA-tagged amine HP2 with 2,4-dimethoxybenzaldehyde 3. Conditions: HP2 (1 nmol), borate buffer (350 mM, pH = 10.8), 3 (400 mM), 5% surfactant, rt, 1.5 h, then NaBH_4_ (440 mM), rt, 16 h

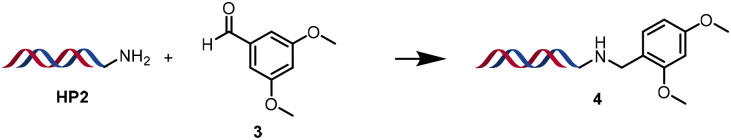		
Entry	Surfactant	Product (%)
1	TPGS-750-M	85
2	PEG_5_C_12_	74
3	Tween 65	45
4	Brij 700	86
5	Brij S20	91
6	Triton-X-405	100
7	TTAC	100
8	Sulfobetaine-16	97

**Table 6:** Substrate scope for the reductive amination of DNA-tagged amine HP2 with several aldehydes. Conditions: HP2 (1 nmol), borate buffer (350 mM, pH = 10.8), aldehyde (400 mM), 5% surfactant, rt, 1.5 h, then NaBH_4_ (440 mM), rt, 16 h

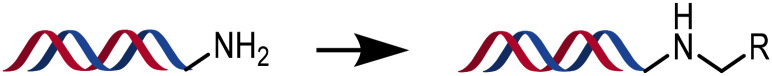					
Entry	Substrate	Product (%)			
		TPGS-750-M	Triton-X-405	TTAC	Sulfobetaine-16
1	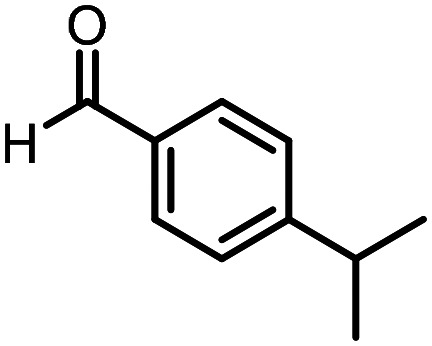	56	42	70	51
2	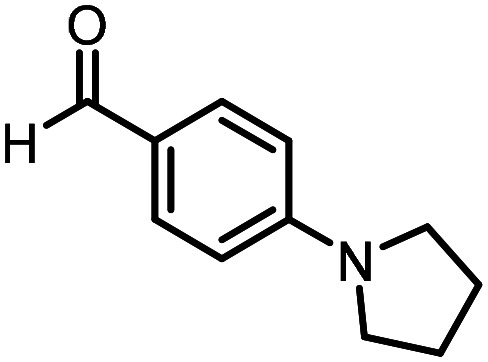	61	22	64	28
3	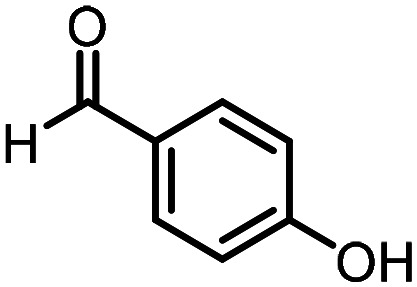	29	19	65	16
4	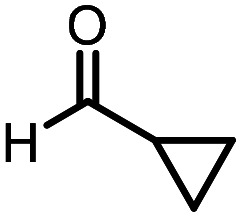	62	42	69	42
5	Average	52	31	67	34

**Table 7:** Extended substrate scope for the reductive amination of DNA-tagged amine HP2 with various aldehydes. Conditions: HP2 (1 nmol), borate buffer (350 mM, pH = 10.8), aldehyde (400 mM), 5% surfactant, rt, 1.5 h, then NaBH_4_ (440 mM), rt, 16 h

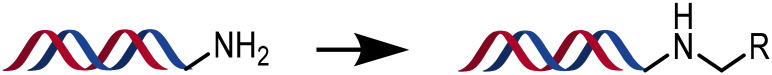			
Entry	Substrate	Product (%)	
		TPGS-750-M	TTAC
1	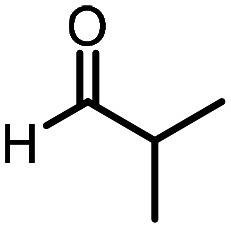	67	44
2	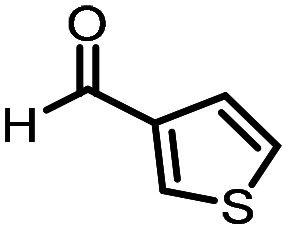	84	54
3	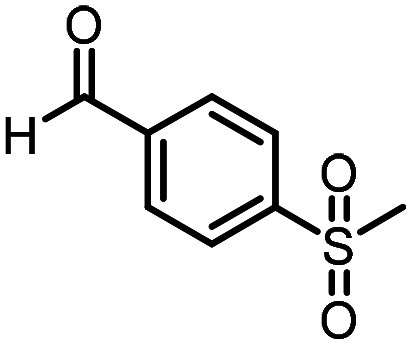	93	82
4	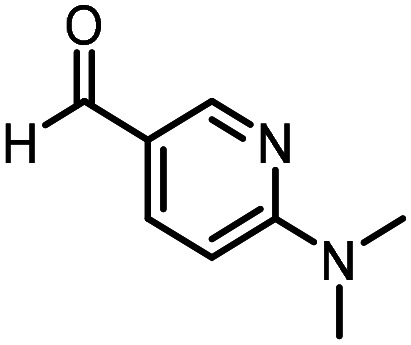	72	58
5	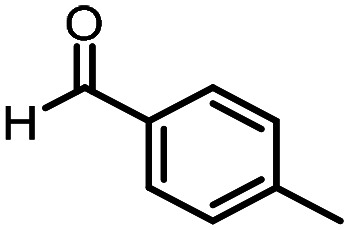	80	85
6	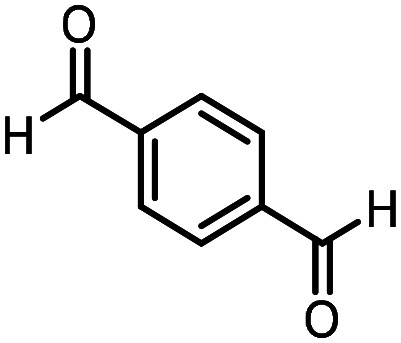	83^a^	83^a^
7	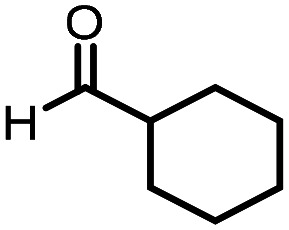	70	69
8	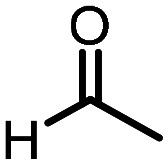	54	57
9	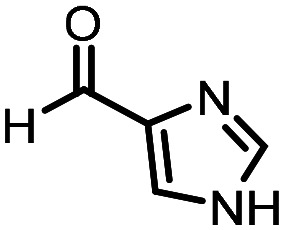	38	65
10	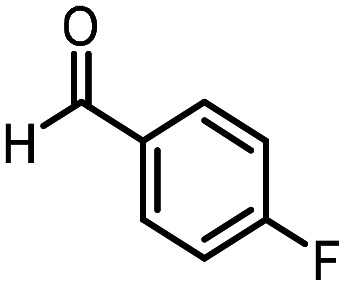	90	97
11	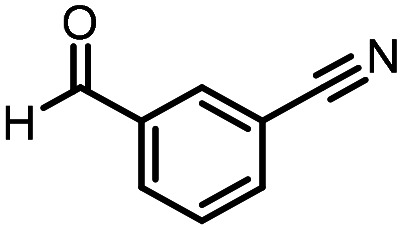	89	98
12	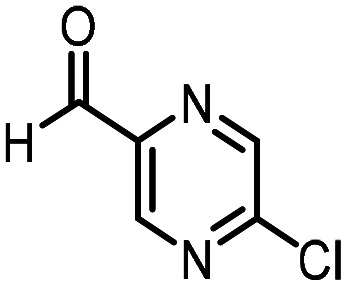	88	98
13	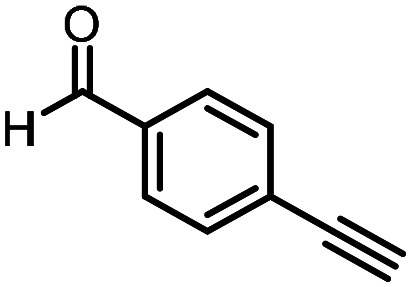	82	94
14	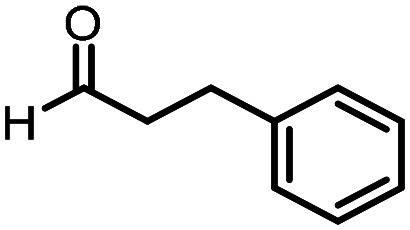	63	77
15	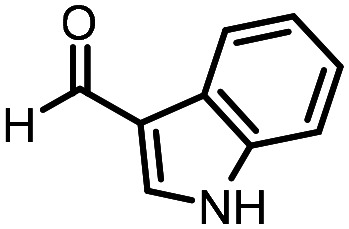	63	75
16	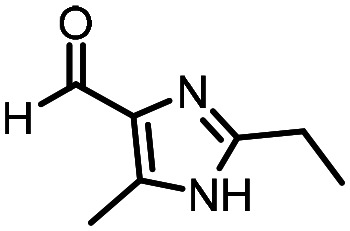	63	71
17	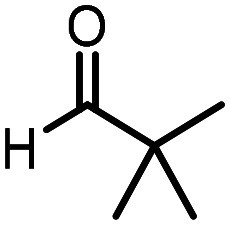	65	86
18	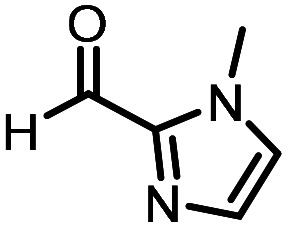	77	96
19	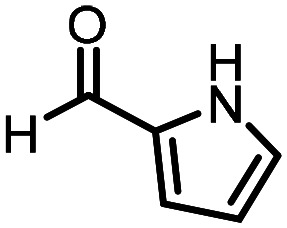	52	73
20	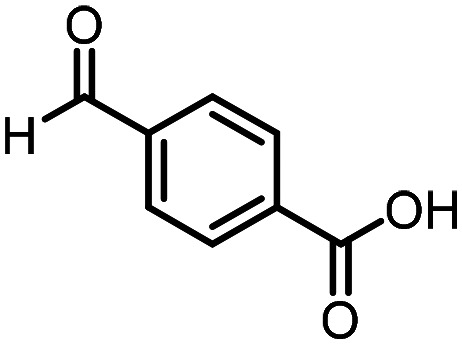	5	37
21	Average	69	75

a Indicates conversion to the product with the aldehyde reduced.

### Reverse amide coupling

Finally, the process was repeated for a “reverse” amide coupling reaction of DNA-tagged acid (HP3) with amine 5, with an expected conversion to desired product of 58%.^16^ The initial surfactant scope was performed using the same surfactants as previously (Table 8). The first thing to note is that the use of TPGS-750-M in this reaction afforded a much lower conversion to desired product (15%) than we had previously observed (58%), equivalent to no surfactant (Table 8, entries 1 and 2). Many of the initial surfactants (Tween 65, Brij 700, Brij S20 and Triton-X-405) offered some improvement in conversion compared with TPGS-750-M (20–27%, Table 8, entries 4–7). However, when both TTAC and sulfobetaine-16 were implemented in the reaction, a significant improvement in conversion was observed (44% and 36% respectively, Table 8, entries 8 and 9). From these initial results, a further subset of surfactants selected using the map was explored (Table 9). None of these surfactants offered any improvement on the reactions, with conversions to desired product ranging from 11–17%. Therefore, TTAC and sulfobetaine-16 were selected as the two best-performing surfactants to assess further. 6 amines occupying a range of chemical space with varying conversions were selected (Table 10). The use of TTAC as the surfactant in this reaction across this subset of substrates led to a significant reduction in the average conversion to desired product compared with using TPGS-750-M. However, sulfobetaine-16 gave a slight improvement and was profiled against further substrates (Table 11).

**Table 8:** Initial surfactant scope for the reverse amide coupling of DNA-tagged acid HP3 with amine 5. Conditions: amine (0.5 M), HOAt (0.5 M), lutidine (1.5 M), DIC (0.5 M), 4.5% surfactant, 45 °C, 3 h

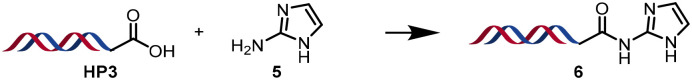		
Entry	Surfactant	Product (%)
1	None	14
2	TPGS-750-M	15
3	PEG_5_C_12_	N/A
4	Tween 65	20
5	Brij 700	26
6	Brij S20	27
7	Triton-X-405	26
8	TTAC	44
9	Sulfobetaine-16	36

**Table 9:** Follow up substrate scope for the reverse amide coupling of DNA-tagged acid HP3 with amine 5. Conditions: amine (0.5 M), HOAt (0.5 M), lutidine (1.5 M), DIC (0.5 M), 4.5% surfactant, 45 °C, 3 h

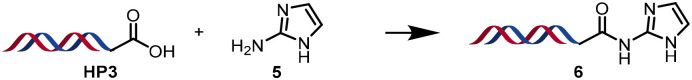		
Entry	Surfactant	Product (%)
1	Tween 85	17
2	Citrol 4DS	17
3	Span 65	17
4	Span 85	11

**Table 10:** Substrate scope for the reverse amide coupling of DNA-tagged acid HP3 with several amines. Conditions: amine (0.5 M), HOAt (0.5 M), lutidine (1.5 M), DIC (0.5 M),4.5% surfactant, 45 °C, 3 h

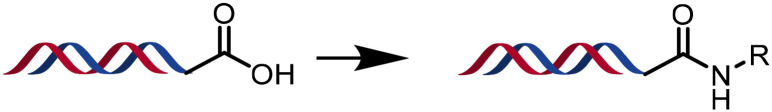				
Entry	Substrate	Product (%)		
		TPGS-750-M	TTAC	Sulfobetaine-16
1	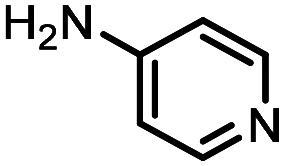	0	0	0
2	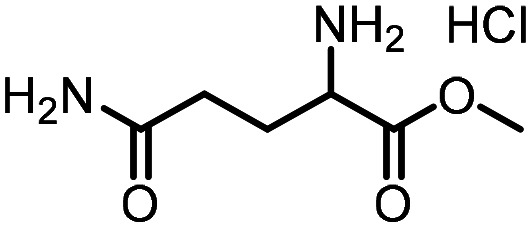	98	98	100
3	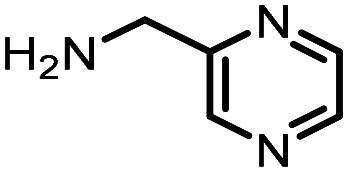	86	81	100
4	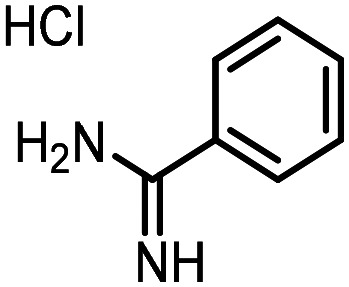	16^a^	11^a^	18^a^
5	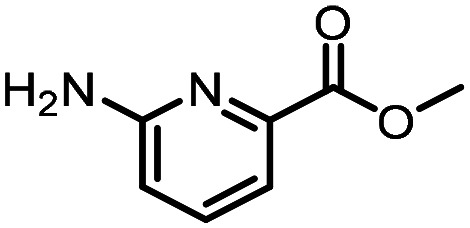	40^b^	15^b^	36^b^
6	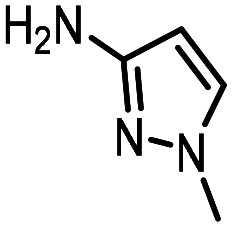	100	61	100
7	Average	57	44	59

a Indicates conversion to a mass corresponding to the hydrolysed imidine product.

b Indicates conversion to the product with the ester hydrolysed.

**Table 11:** Extended substrate scope for the reverse amide coupling of DNA-tagged acid HP3 with various amines. Conditions: amine (0.5 M), HOAt (0.5 M), lutidine (1.5 M), DIC (0.5 M), 4.5% surfactant, 45 °C, 3 h

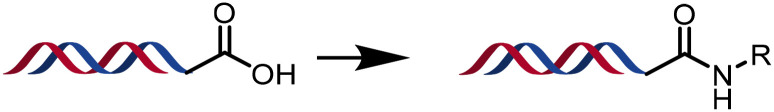			
Entry	Substrate	Product (%)	
		TPGS-750-M	Sulfobetaine-16
1	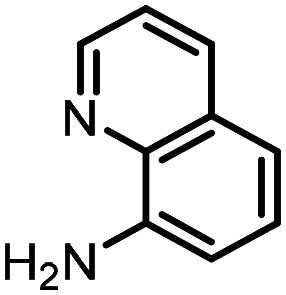	39	32
2	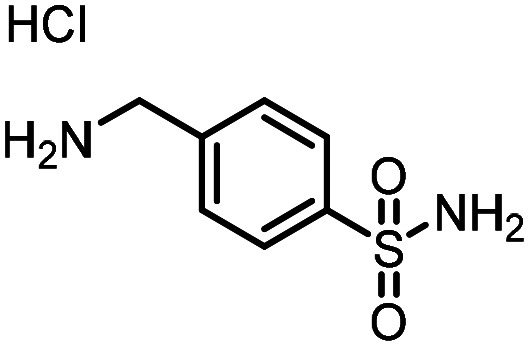	98	94
3	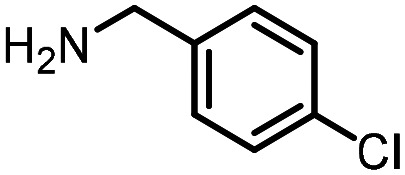	97	95
4	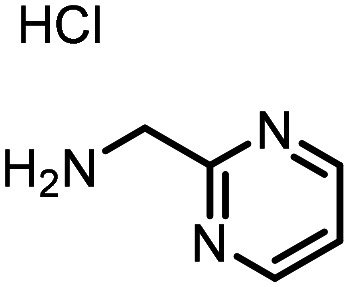	98	99
5	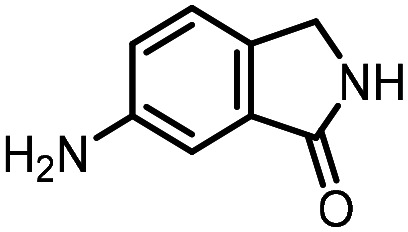	35	30
6	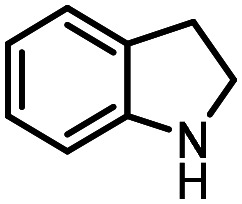	61	75
7	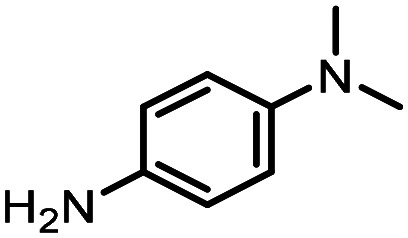	62	75
8	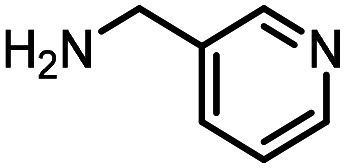	60	85
9	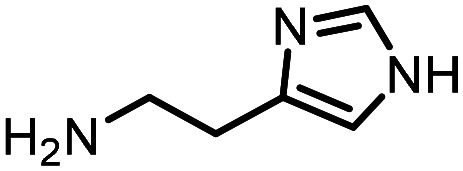	54	72
10	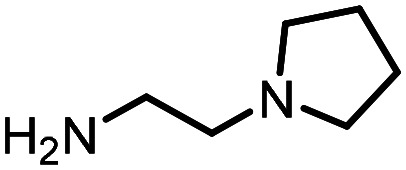	17	42
11	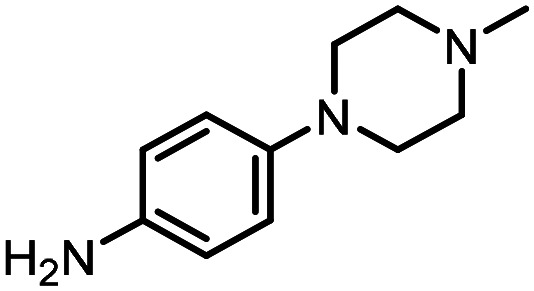	38	83
12	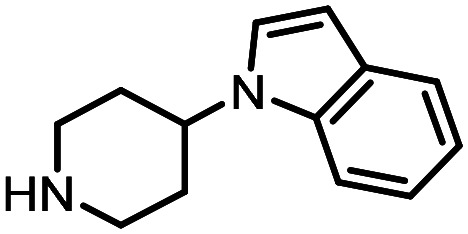	0	89
13	Average	55	73

Only one of the further substrates performed slightly worse when sulfobetaine-16 was utilised (Table 11, entry 1). 4 of the substrates performed similarly (within 5%, entries 2–5). The remaining 7 substrates all afforded improved conversions with sulfobetaine-16 (entries 6–12). Overall, across 19 substrates, conversions were improved for 9 amines when sulfobetaine-16 was used in place of TPGS-750-M. A further 9 substrates afforded the same conversion (within 5%) when either sulfobetaine-16 or TPGS-750-M was chosen as the surfactant, whilst only one substrate afforded a reduced conversion (>5%), albeit only 7% in magnitude. Average conversion across all amine substrates was improved from 53% using TPGS-750-M to 62% with sulfobetaine-16.

### Synthesis of a DNA-conjugated multicycle compound

**Scheme 1:**
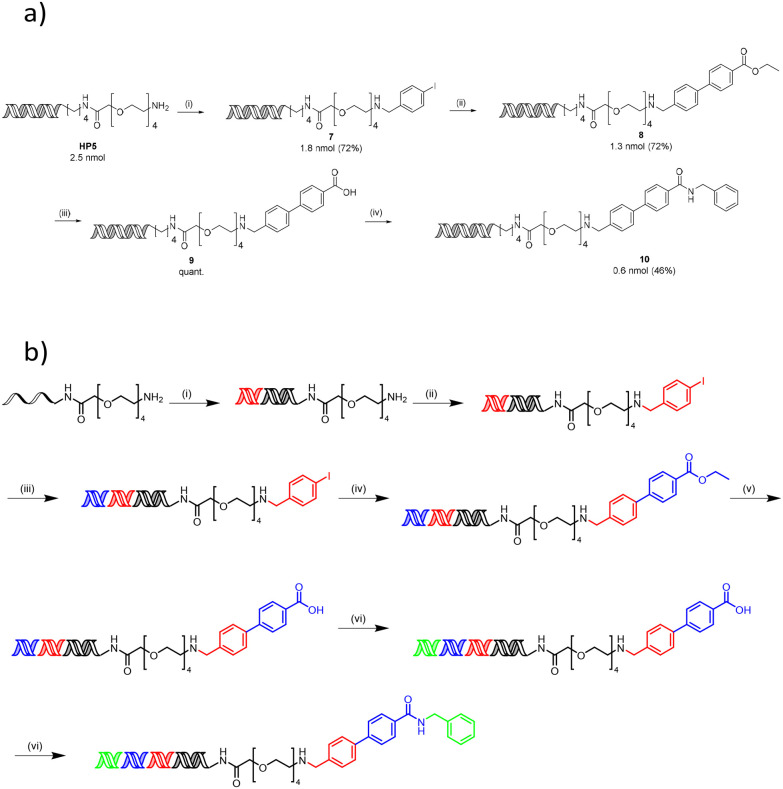
(a) Synthesis of a representative encoded compound using the three reactions optimised in this work. Conditions: (i) borate buffer (350 mM, pH = 10.8), 4-iodobenzaldehyde (400 mM), 5% TTAC, rt, 1.5 h then NaBH_4_ (440 mM), rt 16 h; (ii) (4-(ethoxycarbonyl)phenyl)boronic acid (500 mM), Pd(dtbpf)Cl_2_ (7.3 mM), K_3_PO_4_ (530 mM), 2% 4-dodecylbenzenesulfonic acid, 15% THF, 60 °C, 5 h; (iii) LiOH (250 mM), rt, 30 min; (iv) benzylamine (0.5 M), HOAt (0.5 M), lutidine (1.5 M), DIC (0.5 M), 4.5% sulfobetaine-16, 45 °C, 3 h. (b) Synthesis of a 1 × 1 × 1 library incorporating each of the three reactions optimised in this work. Conditions: (i) first ligation (DNA headpiece, primer, library codon and first DNA building block); (ii) borate buffer (350 mM, pH = 10.8), 4-iodobenzaldehyde (400 mM), 5% TTAC, rt, 1.5 h then NaBH_4_ (440 mM), rt 16 h; (iii) second ligation (second DNA building block); (iv) (4-(ethoxycarbonyl)phenyl)boronic acid (500 mM), Pd(dtbpf)Cl_2_ (7.3 mM), K_3_PO_4_ (530 mM), 2% 4-dodecylbenzenesulfonic acid, 15% THF, 60 °C, 5 h; (v) LiOH (250 mM), rt, 30 min; (vi) third ligation (third DNA building block); (vii) benzylamine (0.5 M), HOAt (0.5 M), lutidine (1.5 M), DIC (0.5 M), 4.5% sulfobetaine-16, 45 °C, 3 h.

To demonstrate the applicability of this work to DEL synthesis, a representative encoded compound was synthesised using a 3-cycle sequence of each reaction utilising the optimal surfactant (Scheme 1a). A reductive amination reaction of PEG-amine (HP5) with 4-iodobenzaldehyde using TTAC yielded compound 7. Suzuki–Miyaura cross-coupling of 7 with (4-(ethoxycarbonyl)phenyl)boronic acid in the presence of 4-dodecylbenzenesulfonic acid led to compound 8, and subsequent ester hydrolysis using aqueous LiOH produced acid 9. Finally, reverse amide coupling with benzylamine and sulfobetaine-16 formed the final compound 10. All reactions proceeded with excellent conversions to desired products and moderate recovery of DNA (comparable to the equivalent reactions using TPGS-750-M) to give final product 10 with a quantitative conversion (Fig. S201–204†) and an overall recovery of 18%.

This synthetic sequence was repeated, incorporating a DNA ligation prior to each chemical transformation to encode each chemical building block and form a representative 1 × 1 × 1 library (Scheme 1b). For each ligation, success was confirmed by the appearance of a major band at the expected number of base pairs upon gel electrophoresis (Fig. S206–208†). PCR amplification with NGS elongation primers also resulted in the expected major band at 161 base pairs (Fig. S209†), which suggests efficient amplification following the synthesis of the library. NGS of the library confirmed that the integrity of the DNA barcodes remained intact, with 78% of >80 000 reads corresponding to the expected sequence.

### Effect of surfactants

Overall, this study has shown that the published surfactant map can be employed for rational and efficient optimisation of surfactant for a wide range of reactions. In most cases, a readily available surfactant was found as a superior replacement for the commonly used TPGS-750-M. This effect could be attributed to the central location of designer surfactants in the map. In the past it has been shown that surfactants occupying this region are sufficiently good at many reactions, but not often the optimal surfactant for each of them.^21^

**Fig. 5:**
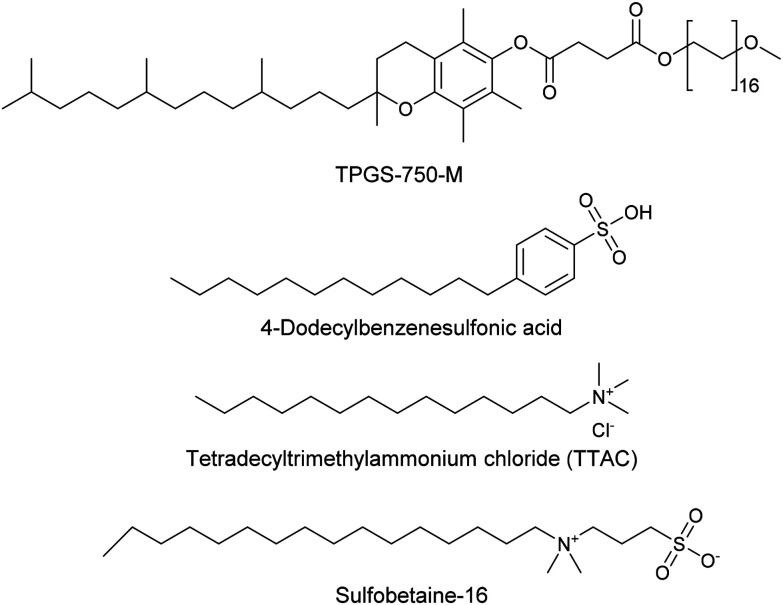
Structures of the TPGS-750-M and the preferred surfactants.

For the three reactions in this study, ionic surfactants were found to the best choices, *i.e.* 4-dodecylbenzenesulfonic acid, TTAC and sulfobetaine-16 (zwitterionic) (Fig. 5). Cationic and zwitterionic surfactants are known to interact with DNAs and can absorb them into micelles.^22,23^ In the case of zwitterionic surfactants, the interaction has been shown to change the shape of DNAs.^24–26^ These can have significant impact on the steric environment around the reaction centres. Given that our reactions were performed at much lower concentrations compared to typical surfactant-enabled organic reactions, the changes to DNAs through micellar interactions are particularly relevant.

It is also noteworthy that the improved surfactants do not contain hydrolytically labile linkers in contrast to TPGS-750-M. TPGS-750-M is known to degrade rapidly under hydrolytic conditions at high temperatures^27^ and this may lead to their reduced effectiveness with prolonged reactions times. This is less likely to be a consideration for the any of the improved surfactants.

## Conclusions

In summary, three established on-DNA micelle-mediated reactions have been improved to afford greater conversions to desired product across a broad substrate scope through the implementation of alternative surfactants to catalyse the reaction, compared to TPGS-750-M, the typical surfactant used in these reactions.

4-Dodecylbenzenesulfonic acid was employed to improve a Suzuki–Miyaura reaction, in which 11 of 24 boronate substrates were improved whilst only one substrate afforded a reduction in conversion. A reductive amination was improved through the use of TTAC as the surfactant in the reaction, in which 16 of the 25 aldehyde substrates gave rise to improved conversions when TTAC was employed, whilst only four substrates yielded reduced conversions compared with TPGS-750-M. Finally, a reverse amide coupling of a DNA-tagged acid with various amines was improved through the use of sulfobetaine-16 as the surfactant in the reaction, whereby conversion was improved for 9 amines and was only reduced for 1 substrate. No damage to the duplex DNA tags was detected in these reactions, even with the use of ionic surfactants. The surfactant map was demonstrated to be an effective tool for surfactant selection and optimisation in ‘micellar catalysis.^21^

Finally, a representative DNA-conjugated compound was successfully synthesised using the optimal surfactant for each of the investigated reactions, and PCR amplification followed by NGS confirmed that the conditions did not cause a detrimental effect to the DNA barcode integrity, highlighting applicability to library synthesis.

## Author contributions

JAO carried out experimental work and co-wrote the manuscript, MJA and TPC carried out experimental work, BN designed the surfactant screens, KF co-supervised JAO, MJW supervised JAO, MJA and TPC and co-wrote the manuscript. All authors reviewed the manuscript prior to publication.

## Conflicts of interest

There are no conflicts to declare.

## Supplementary Material

OB-023-D5OB00864F-s001

## Data Availability

Full supporting data and experimental details of this study are available within the article and the ESI.†
