# Supplementary material for: Improvements in micelle promoted DNA-encoded library synthesis by surfactant optimisation
Source: Org Biomol Chem. 2025 Jun 13;23(28):6745–54. doi: 10.1039/d5ob00864f (PMC12175057; doi:10.1039/d5ob00864f)
Supplement: OB-023-D5OB00864F-s001 [file OB-023-D5OB00864F-s001.pdf]

Supplementary Information (SI) for Organic & Biomolecular Chemistry.
This journal is © The Royal Society of Chemistry 2025

Table of Contents

Contents

Table of Contents 1
Materials and Methods 2
Solvents and Reagents 2
Analytical Techniques 2
Chromatography and Equipment 3
General Procedures 3
General DNA Headpiece Procedure 3
General Ethanol Precipitation Procedure for DNA Purification 4
General on-DNA EDC/Sulfo-NHS Amide Coupling Procedure 4
General on-DNA Suzuki-Miyaura Cross-Coupling Procedure 4
General on-DNA Reductive Amination Procedure 5
General on-DNA Reverse Amide Coupling Procedure 5
Surfactant Map Development 5
List of surfactants 7
Principal component analysis 18
Guide to using the *surfactant_map* 21
Python and R code 21
On-DNA Synthesis of HP1–5 25
On-DNA Suzuki-Miyaura Cross-Coupling Reaction 27
On-DNA Suzuki-Miyaura Cross-Coupling Reaction of DNA-Conjugated Bromopyrazole HP1 27
On-DNA Suzuki-Miyaura Cross-Coupling Reactions of DNA-Conjugated Iodo HP4 32
On-DNA Reductive Amination Reaction of DNA-Conjugated Amine HP2 66
On-DNA Reverse Amide Coupling Reaction of DNA-Conjugated Acid HP3 97
Representative Encoded Compound Synthesis 121
1x1x1 Library Synthesis 124

Materials and Methods

Solvents and Reagents

Reagents were purchased from Acros Organics, Alfa Aesar, Apollo Scientific, BLD Pharmatech, Enamine, Fluka, Fluorochem, Lancaster Synthesis, Manchester Organics, Maybridge, Novabiochem, Sigma-Aldrich; buffer components and reducing agents from Alfa Aesar, Fisher Scientific, and Sigma-Aldrich; and TPGS-750-M from Sigma-Aldrich. All chemicals were used without further purification. Bottles of anhydrous solvents using SureSeal™ were purchased from Sigma-Aldrich. All water used alongside DNA substrates was nuclease-free, DEPC-treated water purchased from ThermoFisher. Solid-supported 14mer DNA and corresponding complementary strand were custom synthesised by Sigma-Aldrich and supplied as solid-supported crude material and single-stranded DNA after desalting, respectively.

Analytical Techniques

DNA mass spectrometry was conducted on an Agilent 6550 QTOF in negative mode, using a standard 3200 m/z maximum and a 2 GHz extended dynamic range. Drying gas temperature was 260 °C at 12 L/min, sheath gas temperature was 400 °C at 12 L/min, nebulizer at 45 psig, VCap voltage of 4000 V, and nozzle voltage of 2000 V. The LC was carried out on an Agilent 1260 infinity 2 using an Agilent Advancedbio oligonucleotides column, 2.1x100 mm where the gradient was run at either:

A) 0.45 mL/min from 40–70% MeOH over 4.5 mins against a 25 mM HFIP:15 mM hexylamine buffer solution.

B) 0.8 mL/min from 20–70% MeOH over 3.5 mins against a 200 mM HFIP:8 mM triethylamine buffer solution.

C) 0.8 mL/min from 10–50% MeOH over 3.5 mins against a 200 mM HFIP:8 mM triethylamine buffer solution.

D) 0.4 mL/min from 10–40% MeOH over 10 mins against a 200 mM HFIP:8 mM triethylamine buffer solution.

All methods were followed by a 1.5 min flush at 95% MeOH.

Analysis of data was carried out by Agilent Qualitative Analysis version 7.

Calculated exact masses were quoted from ChemDraw Professional 18.1. Conversions were determined by integrating peaks in TIC chromatograms for starting material and all products. The proportion of desired product was determined by integrating all peaks, including starting material. Both conversion and desired product were reported as a percentage of all components detected. *Indicates the calculated observed mass based on the observed mass of the complementary strand.

DNA concentrations were calculated using a NanoDrop™ One/OneC Microvolume UV-Vis Spectrophotometer with a 1 µL sample loading.

Gel electrophoresis was conducted using prepacked E-Gel® EX 4% Agarose Gels on an Invitrogen™ E-Gel™ Power Snap Electrophoresis Device, using Invitrogen™ E-Gel™ Ultra Low Range DNA Ladders.

Chromatography and Equipment

Preparative HPLC purification was undertaken on an Agilent 1260 infinity system using a Phenomenex Clarity 5 µm Oligo-RP column, 10x150 mm. The gradient was run at 5 mL/min from 10–95% MeOH over 20 mins against a 50 mM HFIP : 15 mM DIPEA buffer solution. Fractions were analysed at 254 nm wavelength.

Ligations and phosphorylations were carried out using an Applied Biosystems™ ProFlex™ PCR System, 96-well. PCR was carried out using a Bio-Rad CFX Opus 96 Real-Time PCR System.

Unless otherwise specified, centrifugation was undertaken at 13,400 rpm using an Eppendorf™ Centrifuge MiniSpin®, non-refrigerated, except for ethanol precipitations, which were undertaken at 15,000 rpm using an Eppendorf™ Centrifuge 5424 R at 4 °C.

General Procedures

General DNA Headpiece Procedure

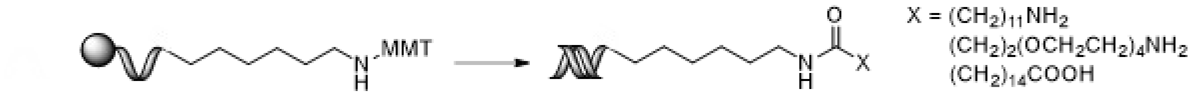

The average loading of single-stranded DNA attached to solid support was found by cleavage from the solid support using the below method and repeating 3 times. Nanodrop concentration of cleaved DNA showed that 103 mg resulted in 2 µmol of DNA. The single-stranded DNA used was a 14mer (GTCTTGCCGAATTC) with a 5′ MMT amino C6 linker bound to solid support at the 3′ end.

MMT Deprotection

The solid-supported DNA (103 mg, ca. 2 µmol) was washed with 5% TCA in DCM (20 x 400 µL). Successful removal of the protecting group was complete once the yellow colour subsided, and the solid-supported DNA was subsequently washed with DCM (14 x 400 µL). The beads were allowed to air dry (2 h) before being carried through to the next step without further analysis or purification.

Linker Coupling

To a 1.5 mL Eppendorf™ Polypropylene DNA LoBind Tube was added HATU (17 mg, 44 µmol), either 12-((((9*H*-fluoren-9-yl)methoxy)carbonyl)amino)dodecanoic acid (18 mg, 40 µmol), 1-(9*H*-fluoren-9-yl)-3-oxo-2,7,10,13,16-pentaoxa-4-azanonadecan-19-oic acid (23 mg, 40 µmol), or 16-(tert-butoxy)-16-oxohexadecanoic acid (14 mg, 40 µmol); and DIPEA (17 µL, 100 µmol) in DMF (1 mL). The reaction mixture was vortexed at room temperature for 15 mins before addition of the deprotected, solid-supported DNA (ca. 2 µmol) and the reaction mixture vortexed at room temperature for 20 h. The reaction mixture was filtered and washed with DMF (3 x 0.5 mL), MeCN (3 x 0.5 mL), MeOH (3 x 0.5 mL) and DCM (3 x 0.5 mL) and allowed to air dry (1 h). The product was carried through to the next step without further analysis or purification.

Cleavage from Solid Support

To a 1.5 mL Eppendorf™ Polypropylene DNA LoBind Tube was added 40 wt% aqueous methylamine solution (0.5 mL) and 33% aqueous ammonia solution (0.5 mL) and the mixture vortexed at room temperature for 15 mins. The solid-supported, linker-bound DNA (ca. 2 µmol) was added and the reaction mixture vortexed at room temperature for 5 h. The reaction mixture was filtered and washed

with DEPC-treated water (3 x 0.5 mL). The resulting mixture was concentrated under N_2 and purified by preparative HPLC (10–90% MeOH in 50 mM HFIP : 15 mM DIPEA buffer solution over 16 mins) to afford the desired compound as a white solid.

t-Butyl Ester Hydrolysis (HP3 only)

To the linker-bound DNA HP3 t-butyl ester (3 equiv.) was added LiOH (1 M in H_2O, 1 equiv.) and the reaction mixture stirred and heated to 80 ˚C for 100 mins. The reaction mixture was allowed to cool to room temperature, before being added to an Amicon Ultra-0.5 Centrifugal 3 kDa Cut-off Filter Unit and centrifuged at room temperature for 10 mins. DEPC-treated water (350 µL) was added to the filter and the mixture centrifuged at room temperature for 10 mins. This was repeated a further 2 times before the filter was inverted and centrifuged at room temperature for 2 mins to yield the aqueous DNA product. The product was purified via preparative HPLC (10–90% MeOH over 16 mins against a 50 mM HFIP : 15 mM DIPEA buffer solution) to afford the desired compound as a white solid.

Complementary Strand Annealing

The complementary 14mer (GAATTCGGCAAGAC), 3' and 5' hydroxylated (1.0 mM in H_2O, 1 equiv.), and linker-bound DNA (1 equiv.) were heated to 80 °C for 30 mins. The reaction mixture was allowed to cool to room temperature, and the double-stranded DNA concentrated under N_2 at rt to form a 1–4 mM solution of PEG–amine HP5, HP2 or HP3. Mass spectrum analysis confirmed product formation.

General Ethanol Precipitation Procedure for DNA Purification

To the DNA product was added aq. NaCl (5 M in H_2O, 0.1 v/v) and ice-cold EtOH (3 v/v) and the mixture vortexed and incubated at -20 °C overnight. The mixture was centrifuged at 4 °C for 15 mins and the supernatant decanted. 70% v/v aq. ice-cold EtOH (200 µL) was added and the mixture centrifuged at 4 °C for 10 mins. The supernatant was decanted to afford the DNA product as a pellet, which was allowed to air dry for 1 h before being redissolved in H_2O.

General on-DNA EDC/Sulfo-NHS Amide Coupling Procedure

To a 1.5 mL Eppendorf™ Polypropylene DNA LoBind Tube was added carboxylic acid (200 mM in DMSO, 12 µL), DMSO (50 µL), EDC.HCl (100 mM in DMSO, 12 µL), and *N*-Hydroxysulfosuccinimide sodium salt (100 mM in 2:1 DMSO:H_2O, 12 µL) and the reaction mixture was vortexed at room temperature for 30 mins. DNA HP (1–50 nmol) in MOPS buffer (100 mM in H_2O, pH=8.0, 50 µL) was added and the reaction mixture heated in a sand bath at 37 °C overnight. The reaction mixture was purified via the general ethanol precipitation procedure and analysed by mass spectrometry.

General on-DNA Suzuki-Miyaura Cross-Coupling Procedure

To a 50 µL glass insert for a Para-dox™ 96-well micro photoredox/optimisation plate was added a solution of boronic acid/ester (750 mM in DMF, 20 µL) and double-stranded DNA (1.5 µL, 1–2 nmol) and the solvents removed at 55 °C in a centrifugal evaporator for 30 mins. To this mixture was added surfactant (5 wt% in H_2O, 24 µL) and potassium phosphate (2.67 M in H_2O, 6 µL) and the vials vortexed for 10 s to enhance mixing. Pd(dtbpf)Cl_2 (50 mM in THF, 4.5 µL) was added and the reaction mixture vortexed for 10 s and heated in a Para-dox™ 96-well micro photoredox/optimisation plate at 60 °C for 5 h. The mixture was diluted to 200 µL H_2O and washed with DCM (200 µL). The organic layer was removed and the aqueous layer filtered through a hydrophobic PTFE filter, purified via the general ethanol precipitation procedure. The precipitated product was added to an Amicon® Ultra-0.5 Centrifugal 3 kDa Cut-off Filter Unit, DEPC-treated water (350 µL was added and the solution centrifuged at room temperature for 10 mins. DEPC-treated water (200 µL) was added to the filter and the mixture centrifuged at room temperature for 10 mins. This was repeated 2 more times, then

the filter was inverted and centrifuged at 4,300 rpm at room temperature for 2 mins to yield the aqueous DNA product which was analysed by mass spectrometry.

General on-DNA Reductive Amination Procedure

To a 0.5 mL Eppendorf™ Polypropylene DNA LoBind Tube was added DNA (5 µL, 0.5–5 nmol), borate buffer (5 µL, 350 mM, pH 10.8), surfactant (5 µL, 5 wt%) and aldehyde (6 µL, 400 mM in MeCN) and the mixture vortexed at room temperature for 10 s to enhance mixing. The mixture was then shaken using a STARLAB® Thermomixer-Mixer HC at 800 rpm at room temperature for 1.5 h. $NaBH_4$ (5 µL, 440 mM in 1:1 v/v MeCN/H_2O) was added and the reaction mixture shaken at room temperature for a further 16 h. The reaction was then precipitated according to the general ethanol precipitation procedure and analysed by mass spectrometry.

General on-DNA Reverse Amide Coupling Procedure

To a 50 µL glass insert for a Para-dox™ 96-well micro photoredox/optimisation plate was added HOAt (2 mg, 15 µmol), amine (15 µmol), surfactant (5 wt% in H_2O, 26 µL), DNA (4 µL, 1 nmol), 2,6-lutidine (5.2 µL, 45 µmol) and DIC (2.2 µL, 15 µmol) and the reaction mixture vortexed to enhance mixing an heated in a Para-dox™ 96-well micro photoredox/optimisation plate at 45 °C for 3 h. The mixture was diluted to 200 µL H_2O and washed with DCM (200 µL). The organic layer was removed and the aqueous layer filtered through a hydrophobic PTFE filter, purified via the general ethanol precipitation procedure and analysed by mass spectrometry.

Surfactant Map Development

Table S1. Descriptors for ***surfactant_map***

Descriptor	Classification	Representing	Source
Critical micelle concentration (CMC)	Micellar property	Surfactant-surfactant interactions	Literature
Aggregation number range	Micellar property	Surfactant-surfactant interactions	Literature
Micelle size range	Micellar property	Surfactant-surfactant interactions, homogeneity	Literature
Contact angles	Emulsion property	Surface tension and wettability	Experimenta l
Zeta potential	Emulsion/micellar property	Charge environment around micelles, emulsions	Experimenta l
Hydrophilic Lipophilic Balance (HLB)	Emulsion property	Emulsion stability	Literature
Hydrophilic	Molecular	Flexibility of surfactant molecules and	*rdkit*

fragment rotatable bonds	property	emulsion flexibility/stability	
Hydrophobic fragment rotatable bonds	Molecular property	Flexibility of surfactant molecules and emulsion flexibility/stability	*rdkit*
Hydrophilic fragment longest chain length	Molecular property	Size of the interface layer between organic and aqueous phases	*rdkit*
Hydrophobic fragment longest chain length	Molecular property	Capability for stabilising organic phase inside emulsions	*rdkit*
Hydrophilic fragment volume	Molecular property	Packing of surfactant molecules and stability of emulsion	*rdkit*
Hydrophobic fragment volume	Molecular property	Packing of surfactant molecules and stability of emulsion	*rdkit*
Hydrophilic fragment surface area	Molecular property	Packing of surfactant molecules and stability of emulsion	*rdkit*
Hydrophobic fragment surface area	Molecular property	Packing of surfactant molecules and stability of emulsion	*rdkit*
Hydrophobic fragment number of C=C bonds	Molecular property	Flexibility of surfactant molecules and emulsion flexibility/stability	*rdkit*
Hydrophilic fragment number of OH groups	Molecular property	Capability of H-bonding at the interface of organic and aqueous phases	*rdkit*
Hydrophilic fragment DG_{solv}	Molecular property	Stability of emulsion	Gaussian, PM6
Hydrophilic fragment dipole moment	Molecular property	Stability of emulsion	Gaussian, PM6
Hydrophilic fragment HOMO energy	Molecular property	H-bonding capability and interactions with transition states	Gaussian, PM6
Hydrophilic fragment LUMO energy	Molecular property	H-bonding capability and interactions with transition states	Gaussian, PM6
Hydrophobic	Molecular	Stability of emulsion	Gaussian,

fragment dipole moment	property		PM6
Hirshfeld charge for most negative heteroatom	Molecular property	Interactions with transition states	Gaussian, PM6, multiwfn

List of surfactants

Table S2. Full list of surfactants in this study

**No.**	**Name**	**Structure**
1	Polysorbate 20	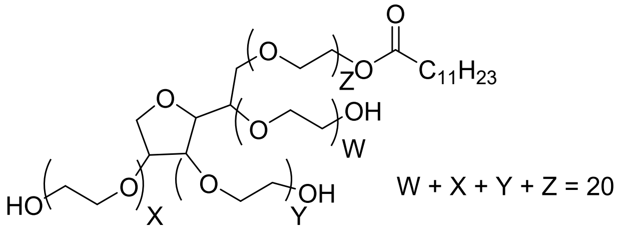
2	Polysorbate 40	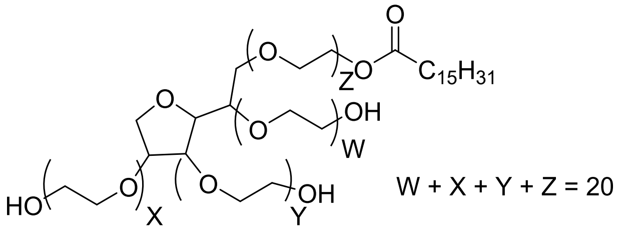
3	Polysorbate 60	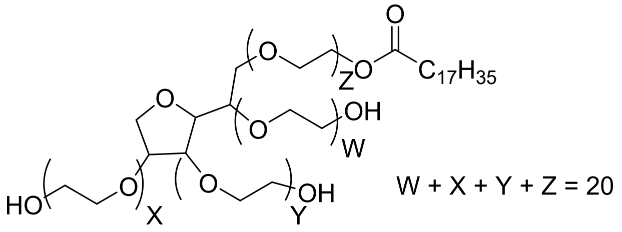
4	Polysorbate 80	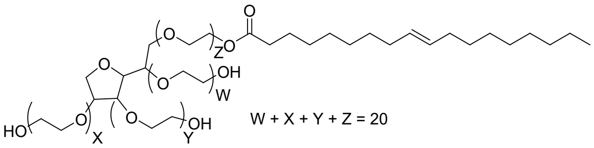
5	Tween 65	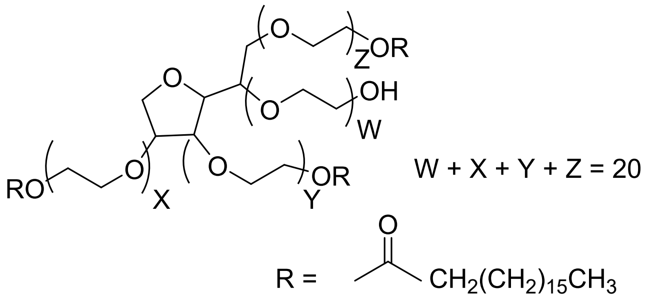

6	Tween 85	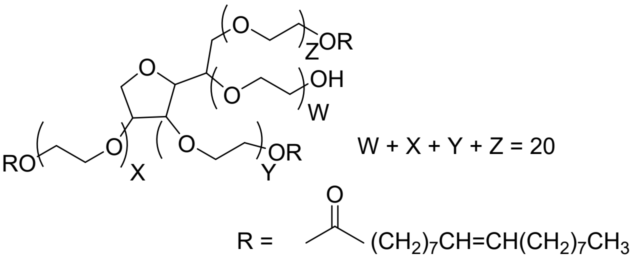
7	Span 20	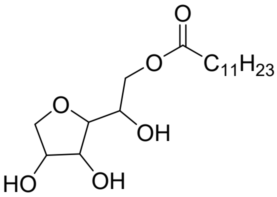
8	Span 40	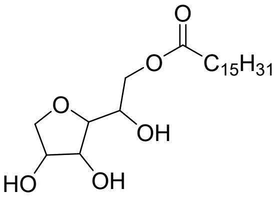
9	Span 60	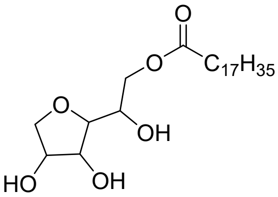
10	Span 80	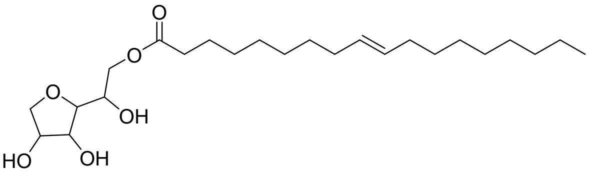
11	Span 65	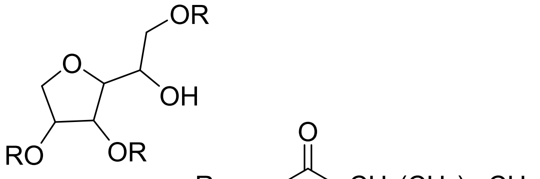
12	Span 85	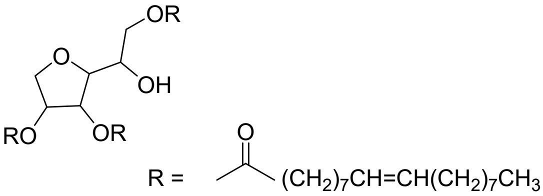
13	C8E6	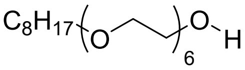
14	Octaethylene glycol monodecyl	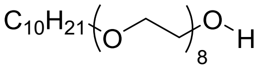

15	Brij 30	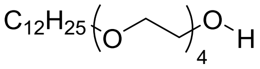
16	C12E5	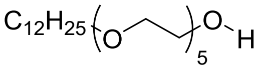
17	C12E6	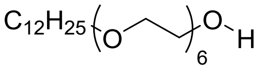
18	Brij 35	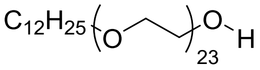
19	Octaethylene glycol monotetradecyl ether	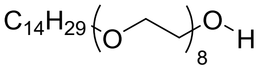
20	SP Brij C2 MBAL/Brij 52	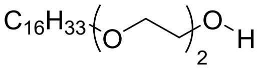
21	C16E8	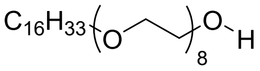
22	Brij 56	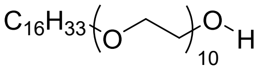
23	Brij 58	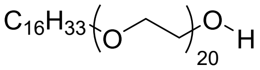
24	Brij 72	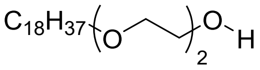
25	C18E8	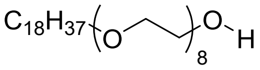
26	Brij S-10	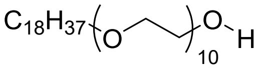
27	Brij 93	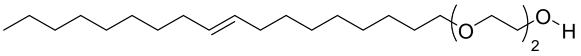
28	Brij O10	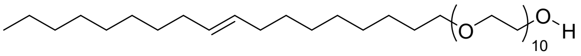
29	Brij 99	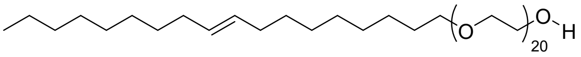
30	Brij S20	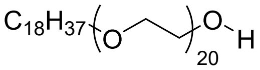
31	Brij 721	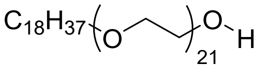
32	Brij 700	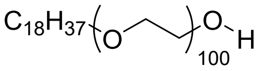

33	Triton-X-15	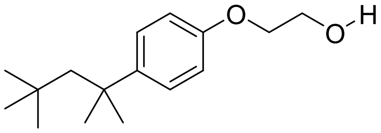
34	Triton-X-35	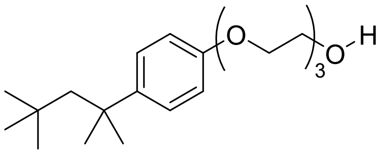
35	Triton-X-45	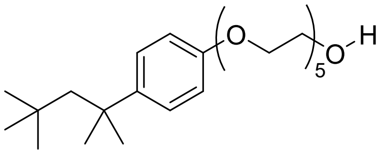
36	Triton-X-100	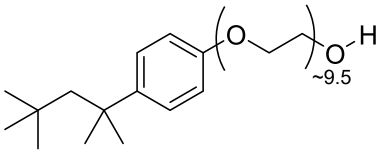
37	Triton-X-102	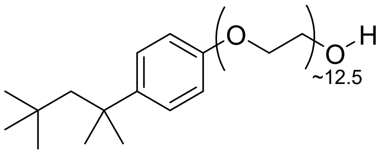
38	Triton-X-114	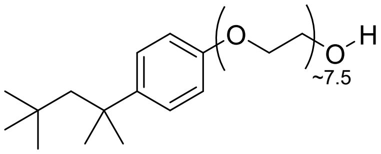
39	Triton-X-165	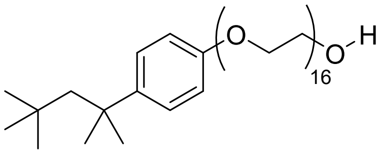
40	Triton-X-305 (	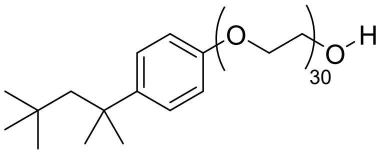
41	Triton-X-405	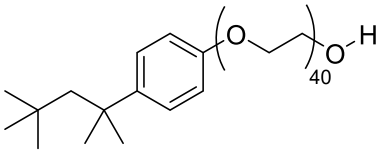
42	Triton-X-705	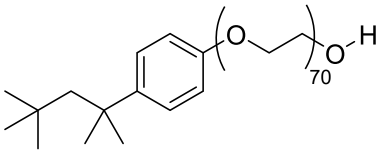

43	IGEPAL CA720	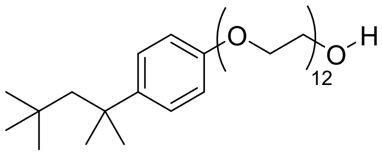
44	IGEPAL CO520	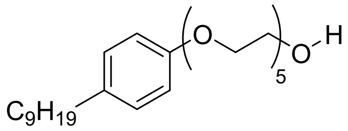
45	IGEPAL CO630	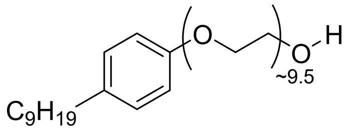
46	IGEPAL CO720	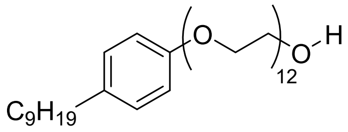
47	Myrj S8	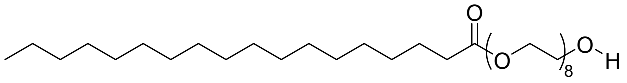
48	Myrj S20	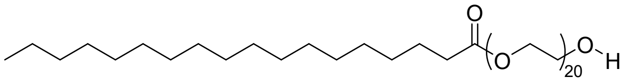
49	Myrj 52	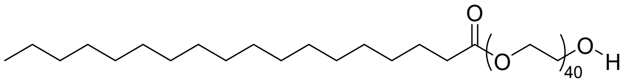
50	Myrj 53	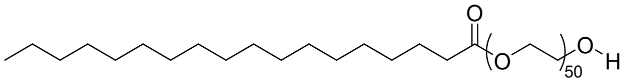
51	Myrj 59	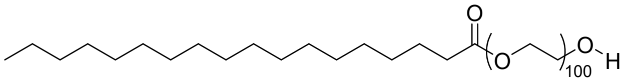
52	DDM (N-Dodecyl b-D-maltoside)	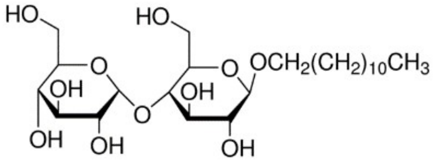
53	Nonyl b-D-glucopyranoside	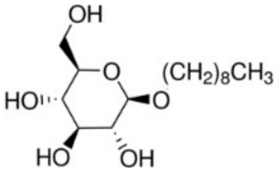

54	Sucrose monolaurate	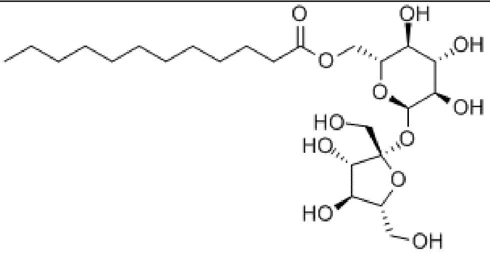
55	Sucrose Distearate	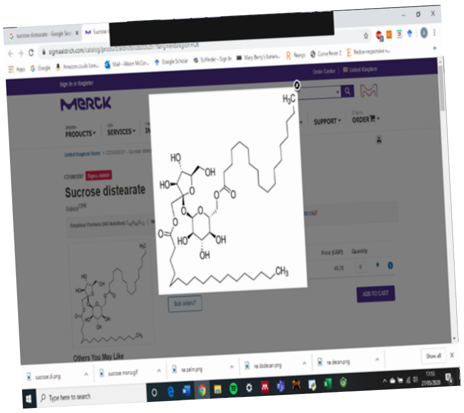
56	CTAB	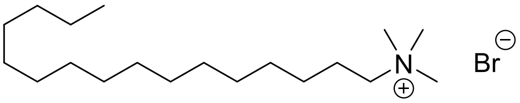
57	CTAC	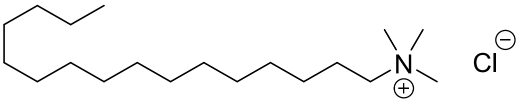
58	DTAB	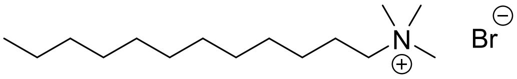
59	DTAC	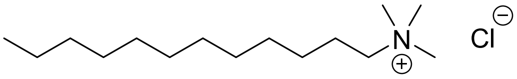
60	TTAC	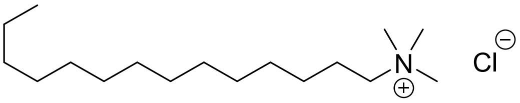
61	TTAB	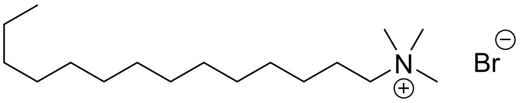
62	CPC ((1-Hexdecyl)pyridinium chloride monohydrate)	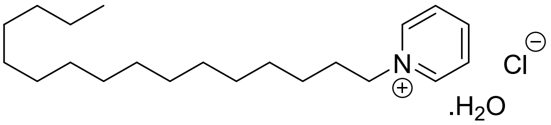
63	DDAB (Di-n-dodecyl)dimethyl ammonium bromide)	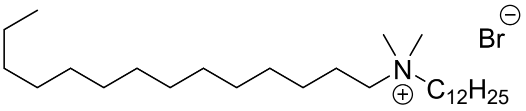

64	CHAPS (3-((3-cholamidopropyl) dimethylammonio)-1-propanesulfonate)	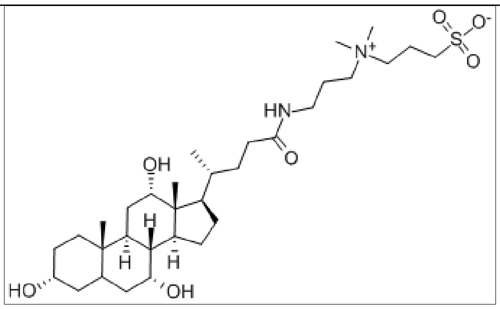
65	DAPS (N-dodecyl-N,N-dimethyl-3-ammonium-1-propane sulfonate)	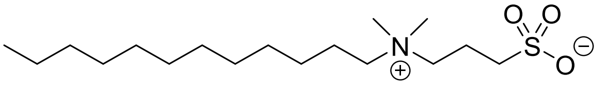
66	3-(N,N-Dimethylmyristylammonio)propanesulfonate	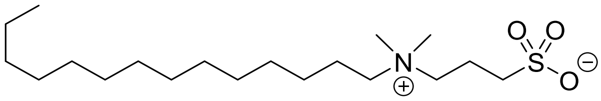
67	Sulfobetaine-16	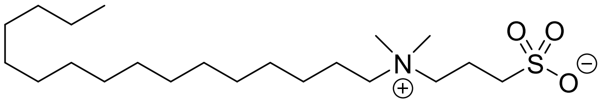
68	Lauryldimethylammonio)acetate	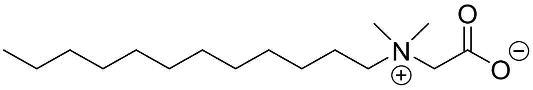
69	TPGS-750-M	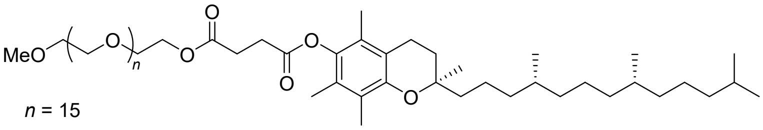
70	TPGS-1000 (DL-alpha-Tocopherolmethoxypolyethyleneglycol 1000 succinate)	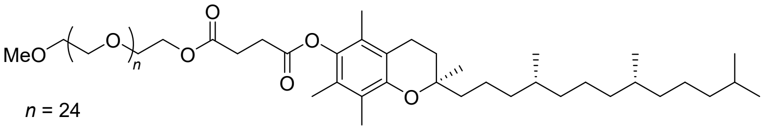
71	SPGS-550-M	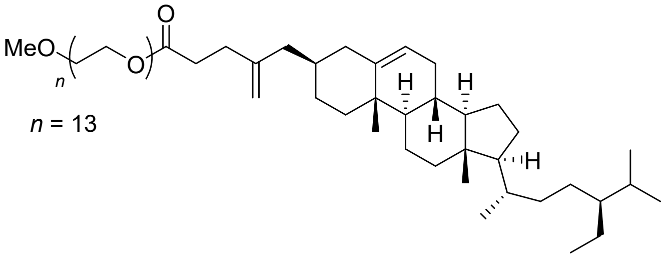
72	PSS	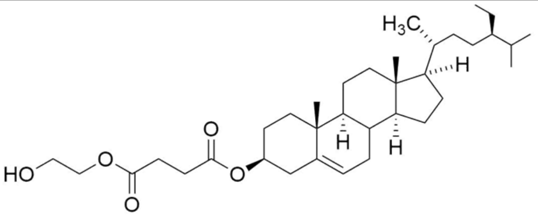

73	PTS	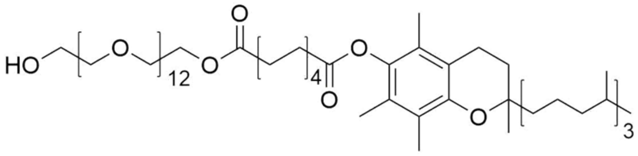
74	Coolade	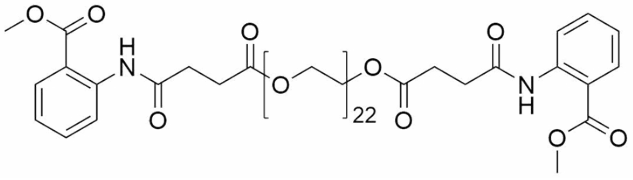
75	PS-750-M	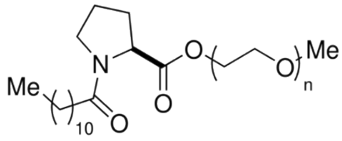
76	Sodium Octanoate/Sodium caprylate	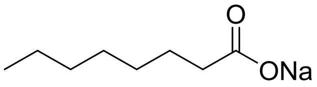
77	Sodium decanoate	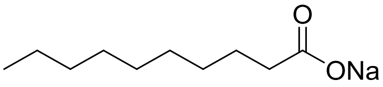
78	Sodium Laurate	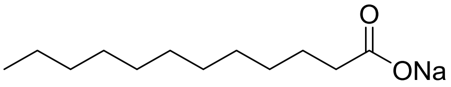
79	Palmitic acid sodium salt	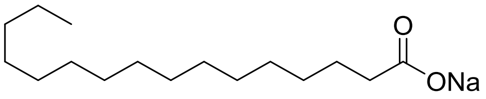
80	Sodium Stearate	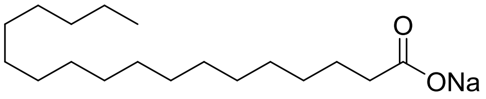
81	Sodium Oleate	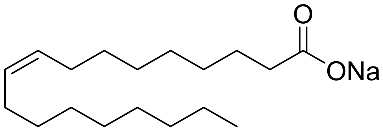
82	1-Decanesulfonic acid, sodium salt	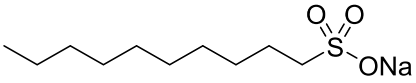
83	1-Dodecanesulfonic acid, sodium salt	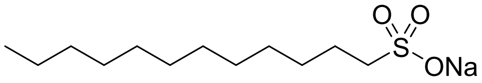
84	Sodium 1-tetradecanesulfonate	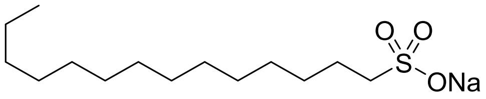
85	Sodium 1-hexadecanesulfonate	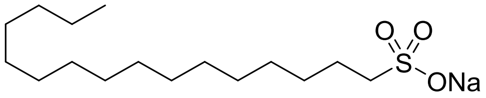

86	n-Decyl sodium sulfate	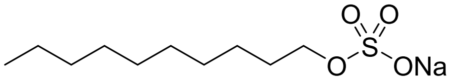
87	SDS	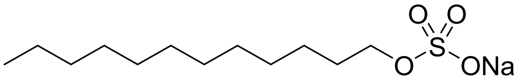
88	Sodium-1-tetradecyl sulfate	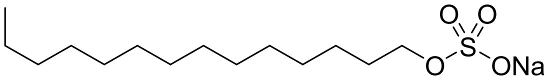
89	Sodium Hexadecyl sulfate	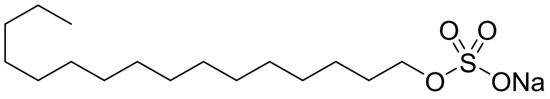
90	Sodium n-octadecy sulfate	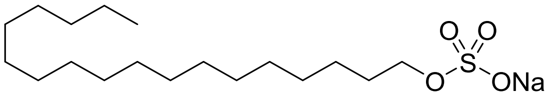
91	Sodium 4-n-octylbenzenesulfonate	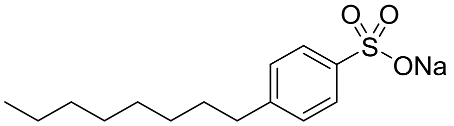
92	SDBS	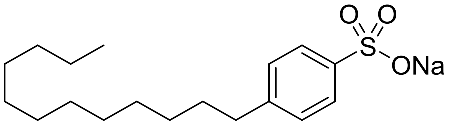
93	4-Dodecylbenzene sulfonic acid	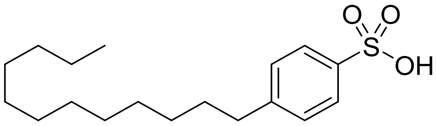
94	Dioctyl sulfosuccinate sodium salt	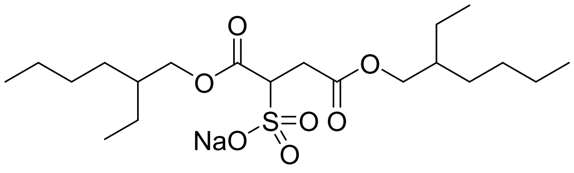
95	Dihexyl sodium sulfosuccinate	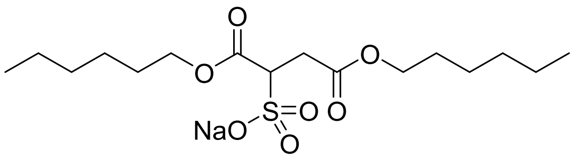
96	Cithrol 10MS	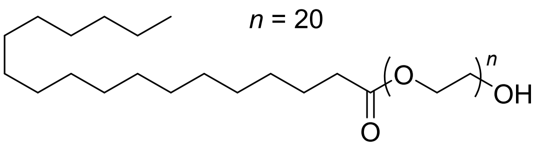
97	Citrhol 4DS	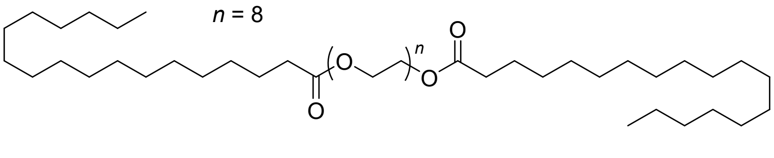

98	Croduret 25-LQ	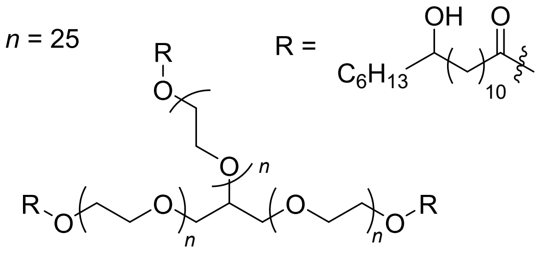
99	Croduret 50-SS	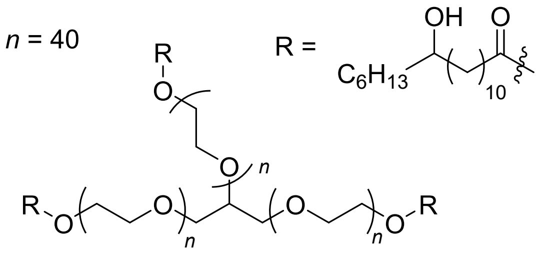
10<br>0	Crodasinic LS95	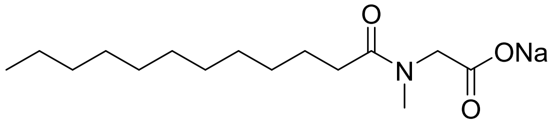

Table S3. Trimmed list of surfactants to reduce uncertainty due to missing data

No.	Name	No.	Name
1	Polysorbate 20	47	Nonyl b-D-glucopyranoside
2	Polysorbate 40	48	Sucrose monolaurate
3	Polysorbate 60	49	CTAB
4	Polysorbate 80	50	CTAC
5	Tween 65	51	DTAB
6	Tween 85	52	DTAC
7	Span 20	53	TTAC
8	Span 40	54	TTAB
9	Span 60	55	CPC ((1-Hexdecyl)pyridinium chloride monohydrate)
10	Span 80	56	DDAB (Di-n-dodecyl)dimethyl ammonium bromide)
11	Span 65	57	CHAPS (3-((3-cholamidopropyl) dimethylammonio)-1-propanesulfonate)
12	Span 85	58	DAPS (N-dodecyl-N,N-dimethyl-3-ammonium-1-propane sulfonate)

13	Octaethylene glycol monodecyl	59	3-(N,N-Dimethylmyristylammonio)propanesulfonate
14	Brij 30	60	Sulfobetaine-16
15	C12E5	61	Lauryldimethylammonio)acetate
16	C12E6	62	TPGS-750-M
17	Brij 35	63	TPGS-1000 (DL-alpha-Tocopherolmethoxypolyethyleneglycol 1000 succinate)
18	Octaethylene glycol monotetradecyl ether	64	SPGS-550-M
19	SP Brij C2 MBAL/Brij 52	65	PTS
20	C16E8	66	PS-750-M
21	Brij 56	67	Sodium Octanoate/Sodium caprylate
22	Brij 58	68	Sodium decanoate
23	Brij S-10	69	Sodium Laurate
24	Brij 93	70	Palmitic acid sodium salt
25	Brij O10	71	Sodium Stearate
26	Brij S20	72	Sodium Oleate
27	Brij 721	73	1-Decanesulfonic acid, sodium salt
28	Brij 700	74	1-Dodecanesulfonic acid, sodium salt
29	Triton-X-15	75	Sodium 1-tetradecanesulfonate
30	Triton-X-35	76	Sodium 1-hexadecanesulfonate
31	Triton-X-45	77	n-Decyl sodium sulfate
32	Triton-X-100	78	SDS
33	Triton-X-102	79	Sodium-1-tetradecyl sulfate
34	Triton-X-114	80	Sodium Hexadecyl sulfate
35	Triton-X-165	81	Sodium n-octadecy sulfate
36	Triton-X-305	82	Sodium 4-n-octylbenzenesulfonate
37	Triton-X-405	83	SDBS
38	Triton-X-705	84	4-Dodecylbenzene sulfonic acid
39	IGEPAL CA720	85	Dioctyl sulfosuccinate sodium salt

40	IGEPAL CO520	86	Dihexyl sodium sulfosuccinate
41	IGEPAL CO630	87	Cithrol 10MS
42	IGEPAL CO720	88	Citrhol 4DS
43	Myrj S8	89	Croduret 25-LQ
44	Myrj S20	90	Croduret 50-SS
45	Myrj 52	91	Crodasinic LS95
46	DDM (N-Dodecyl b-D-maltoside)		

Principal component analysis

Algorithms

PPCA: Probabilistic Principal Component Analysis is a method to estimate the principal axes when any data vector has one or more missing values.[29,30]

PPCA is based on an isotropic error model. It seeks to relate a *p*-dimensional observation vector *y* to a corresponding *k*-dimensional vector of latent (or unobserved) variable *x*, which is normal with mean zero and covariance *I*(*k*). The relationship is

$yT= W*xT+\mu+\varepsilon$,

where *y* is the row vector of observed variable, *x* is the row vector of latent variables, and *ε* is the isotropic error term. *ε* is Gaussian with mean zero and covariance of *v*I(k)*, where *v* is the residual variance. Here, *k* needs to be smaller than the rank for the residual variance to be greater than 0 (*v*>0). Standard principal component analysis, where the residual variance is zero, is the limiting case of PPCA. The observed variables, *y*, are conditionally independent given the values of the latent variables, *x*. So, the latent variables explain the correlations between the observation variables and the error explains the variability unique to a particular y_i. The *p*-by-*k* matrix *W* relates the latent and observation variables, and the vector *μ* permits the model to have a nonzero mean. PPCA assumes that the values are missing at random through the data set. This means that whether a data value is missing or not does not depend on the latent variable given the observed data values.

Under this model,

$y \sim N(\mu,W*WT+v*I(k))$.

There is no closed-form analytical solution for *W* and *v*, so their estimates are determined by iterative maximization of the corresponding loglikelihood using an expectation-maximization (EM) algorithm. This EM algorithm handles missing values by treating them as additional latent variables. At convergence, the columns of *W* spans the subspace, but they are not orthonormal. PPCA obtains the orthonormal coefficients, for the components by orthogonalization of *W*.

BPCA: Bayesian Principal Component Analysis combines an EM approach for PCA with a Bayesian model. In standard PCA data far from the training set but close to the principal subspace may have

the same reconstruction error. BPCA defines a likelihood function such that the likelihood for data far from the training set is much lower, even if they are close to the principal subspace.[31]

BPCA works iteratively, the complexity is growing with $O(n^3)$ because several matrix inversions are required. The size of the matrices to invert depends on the number of components used for re-estimation.

Finding the optimal number of components for estimation is not a trivial task; the best choice depends on the internal structure of the data. A method called kEstimate is provided to estimate the optimal number of components via cross validation. In general few components are sufficient for reasonable estimation accuracy. See also the package documentation for further discussion about on what data PCA-based missing value estimation makes sense.

Details about the probabilistic model underlying BPCA are found in Oba et. al 2003.[32] The algorithm uses an expectation maximation approach together with a Bayesian model to approximate the principal axes (eigenvectors of the covariance matrix in PCA). The estimation is done iteratively, the algorithm terminates if either the maximum number of iterations was reached or if the estimated increase in precision falls below $1e^-4$.

Complexity: The relatively high complexity of the method is a result of several matrix inversions required in each step. Considering the case that the maximum number of iteration steps is needed, the approximate complexity is given by the term

Where row_miss is the number of rows containing missing values and $O(n^3)$ is the complexity for inverting a matrix of size $components$. Components is the number of components used for re-estimation.

NIPALS: Nonlinear Iterative Partial Least Squares method is a method presented by Wold to allow principal component analysis with missing values.[33] The NIPALS algorithm is applied on the dataset and the obtained PCA model is used to predict the missing values.[34]

The NIPALS algorithm can be modified to accommodate missing values using the method of Martens and Martens (p. 381).[35]

If, for a certain variable *k* [column of *X*], a missing value is encountered in X for a certain object *i* [row of *X*], then the corresponding elements in t_{ih} must also be skipped in the calculation of the loadings, which for *X*-variable *k* is

$$p_{hk} = X_{k,h-1} t_h' / (t_h' t_h)$$

Likewise, if, for a certain sample *i* [row of *X*], a missing value is encountered in *X* for a certain variable *k* [column of *X*], then the corresponding elements in p_{kh} must also be skipped in calculating the scores, which for sample *i* is

$$t_{ih} = X_{i,h-1} p_h / (p_h' p_h)$$

This method may have convergence problems if there are many missing values.

NLPCA: Nonlinear Principal Component Analysis is generally seen as a non-linear generalisation of standard linear principal component analysis. The principal components are generalised from straight lines to curves.[36]

The algorithm, proposed by Kramer,[37] is based on a multi-layer perceptron (deep neural network) with an auto-associative topology, also known as an autoencoder, replicator network, bottleneck or sand glass type network.

The network can be divided into two parts: the first part represents the extraction function $\Phi_{extr}: X \rightarrow Z$, whereas the second part represents the inverse function, the generation or reconstruction function $\Phi_{gen}: Z \rightarrow \hat{X}$. A hidden layer in each part enables the network to perform non-linear mapping functions.

The inverse NLPCA model can be easily extended to be applicable to incomplete datasets. If the *i*th element x_i^n of the *n*th sample vector x^n is missing, the partial error σ_i^n is set to zero before back-propagating; hence this error is ignored, and it has no contribution to the gradients. Thus, the non-linear components are extracted from all the available observations. With these components the original data can be reconstructed, including the missing values. The network output x_i^n gives the estimation of the missing element x_i^n.

PCA results

Due to missing data in the dataset for some experimental values a “traditional” PCA approach could not be used on the dataset. Instead, four PCA approaches which can be employed on incomplete datasets were trialled: Bayesian PCA (BPCA), probabilistic PCA (PPCA), non-linear PCA (NLPCA) and on-linear iterative partial least squares (NIPALS). The relevant code for data normalisation, PCA and plotting are in Section 4.

Table S4. NIPALS loadings for descriptors and PCs

**Descriptor**	**PC1**	**PC2**	**PC3**
Contact_angle_left	0.119849	0.188936	0.146323
Contact_angle_right	0.100006	0.197218	0.134578
Zeta_potential	-0.04506	0.156862	0.317523
Size_low	-0.0092	0.00593	-0.0531
Size_high	-1.61×10^{-5}	0.00063	-0.05109
CMC	-0.07685	-0.03592	-0.02751
Aggregation_number_low	-0.06601	0.102619	0.10661
Aggregation_number_high	-0.04075	0.095504	0.12053
HLB	-0.0652	-0.11232	0.01585
Area_hydrophilic	0.375622	-0.23636	0.082023
Area_hydrophobic	0.2979	0.333079	-0.09485
Volume_hydrophobic	0.30495	0.325878	-0.11382
Volume_hydrophilic	0.36434	-0.25371	0.114468

Rotatable_bonds_hydrophilic	0.364517	-0.25523	0.11818
Rotatable_bonds_hydrophobic	0.285553	0.350459	-0.08946
Longest_chain_length_hydrophilic	0.335862	-0.29298	0.129539
Longest_chain_length_hydrophobic	0.27726	0.28896	-0.0477
Philic_DeltaG_sol	0.189123	-0.26703	0.193799
Philic_Solv_dip	0.00501	0.033201	-0.25541
Philic_HOMO	0.077073	-0.14427	-0.52856
Philic_LUMO	0.074148	-0.17595	-0.49362
Philic_Most_neg	0.053238	0.069546	0.298744
Phobic_Solv_dip	0.202227	0.126911	-0.14908
Double_bonds_hydrophobic	0.069898	0.137099	-0.07263
OH_groups_hydrophilic	0.042828	-0.09282	-0.04409

*Contributors with coefficients above 0.15 are in green

Guide to using the *surfactant_map*

The map can be utilised for rational and rapid surfactant screening/optimisation for any given reactions. The step-by-step guide is provided below as guidelines for researchers less familiar with Principal Component Analysis maps. The process is summarised in Figure 5 of the manuscript.

Step 1: Select one surfactant as the initial surfactant. This can be a surfactant which enables the reaction in water from previous experiments, but the results need improvement. If no prior surfactant is known for the reaction, TPGS-750-M is generally a good surfactant to start with, which occupies a central position in the ***surfactant_map***.

Step 2: Select another 7-8 surfactants using the surfactant_map to maximise the space covered by these surfactants. These surfactants will form ***screen1***, and help guide the optimisation in ***screen2*** to the right area of the map. This can be done manually with the ***surfactant_map***, plotted in 3D, or automatically using the Python code in section 5.7. This code randomises the choices of surfactants to maximise the distance between them in the ***surfactant_map*** with only the first selected surfactant being constant as a benchmark.

Step 3: Carry out screen1 using the surfactants selected in Step 2. The best performing surfactants should be compared to identify the area in the surfactant_map where the best results are obtained.

Step 4: Based on the results of screen1, 4-5 surfactants are manually selected around the best performing surfactants for ***screen2***.

Step 5: Perform ***screen2*** and identify the best surfactant for the reaction, enabling further process optimisation through reaction conditions (*e.g.* temperature, concentration, etc.) and stoichiometry.

Python and R code

Python code for surfactant selection

```
import pandas as pd
import math
import random

df = pd.read_csv("surfactant_map.csv")

# the numbering of the first surfactant hit for the reaction
hit_index = 26
# the total number of cycles for optimisation of covering surfactant space
max_cycles = 1000
# the number of surfactants to be selected in the screen
number_of_surfactants = 8

# function to calculate the distance in 3D space between two surfactants in PC1-3
def distance(index1,index2):
    pc1_1 = float(df['PC1'][index1-1])
    pc2_1 = float(df['PC2'][index1-1])
```

```
    pc3_1 = float(df['PC3'][index1-1])
    pc1_2 = float(df['PC1'][index2-1])
    pc2_2 = float(df['PC2'][index2-1])
    pc3_2 = float(df['PC3'][index2-1])
    calc_distance = math.sqrt((pc1_1 - pc1_2)**2 + (pc2_1 - pc2_2)**2 + (pc3_1 -
pc3_2)**2)
    return calc_distance

# Calculate sum of distances to index 1
def distance_sum(hit_indexes):
    sum_of_distances = 0
    for ind in df['Number']:
        distance_from_hit = 0
        if ind not in hit_indexes :
            distance_from_hit = 100
            for hit_ind in hit_indexes:
                individual_distance = distance(hit_ind, ind)
                # print (individual_distance)
                if individual_distance < distance_from_hit:
                    distance_from_hit = individual_distance
        sum_of_distances = sum_of_distances + distance_from_hit
    return sum_of_distances

def all_hit_indexes():
    gen_hit_indexes = list(combinations(df['Number'], number_of_surfactants-1))
    all_hit_indexes = [list(elem) for elem in gen_hit_indexes] # convert into list of
lists
    return all_hit_indexes

# need a faster way of generating the first index_combinations = []
index_list = list(df['Number'])
index_list.remove(hit_index)
hit_indexes = []
```

```
for i in range (1, number_of_surfactants):
    random_item = random.choice(index_list)
    index_list.remove(random_item)
    hit_indexes.append(random_item)

# create a copy and add the first hit index
full_hit_indexes = hit_indexes
full_hit_indexes.append(hit_index)
print(full_hit_indexes)

# store data of the first position
current_hit_indexes = full_hit_indexes
current_distance = distance_sum(current_hit_indexes)
print (current_hit_indexes, current_distance)

# optimisation code to minimise distance by swapping one element in the list at a time
temp_hit_indexes = current_hit_indexes.copy()
for i in range(1, max_cycles):
    remove_index = random.randint(0, len(temp_hit_indexes)-1)
    new_index = random.randint(1, len(df['Number']))
    if temp_hit_indexes[remove_index] != hit_index:
        if new_index not in current_hit_indexes:
            temp_hit_indexes.remove(temp_hit_indexes[remove_index])
            temp_hit_indexes.append(new_index)
            temp_distance = distance_sum(temp_hit_indexes)
            if temp_distance < current_distance:
                current_hit_indexes = temp_hit_indexes.copy()
                current_distance = distance_sum(current_hit_indexes)
                print (current_hit_indexes, current_distance)
        temp_hit_indexes = current_hit_indexes.copy()
print ('Final results: ',current_hit_indexes, current_distance)

final_data = []
final_datum = [current_hit_indexes, current_distance]
```

On-DNA Synthesis of HP1–5

PEG amine HP5 was synthesised using 1-(9*H*-fluoren-9-yl)-3-oxo-2,7,10,13,16-pentaoxa-4-azanonadecan-19-oic acid according to the general DNA headpiece procedure.

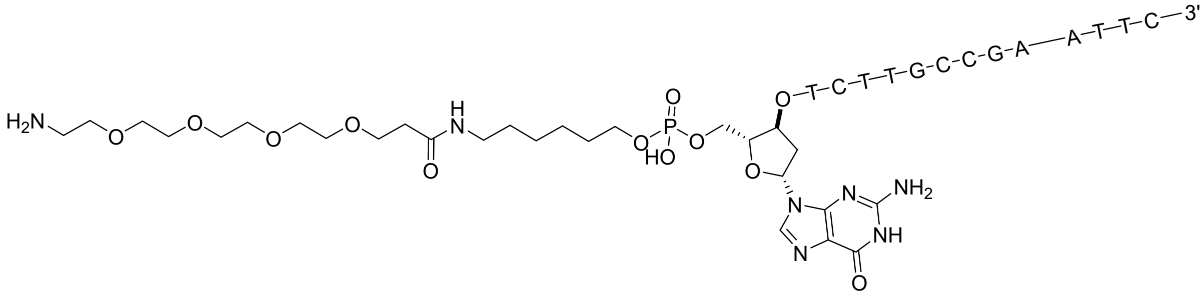

Figure S1: Mass spectrum of double-stranded PEG amine HP5, comprising complementary strand and PEG HP analysed by DNA mass spectrometry gradient A.

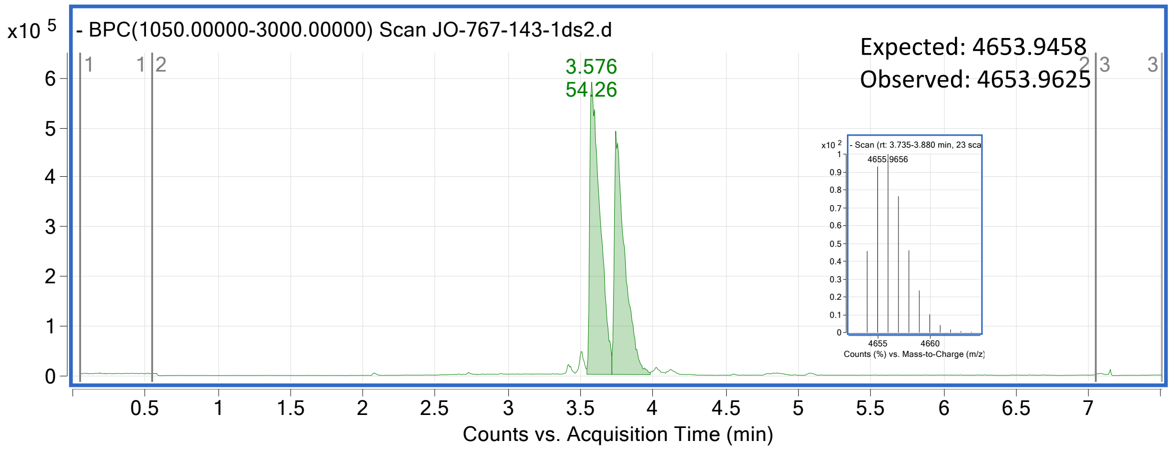

HP1 was synthesised using PEG amine HP and 2-(4-bromo-1*H*-pyrazol-1-yl)acetic acid according to the general on-DNA EDC/Sulfo-NHS forward amide coupling procedure.

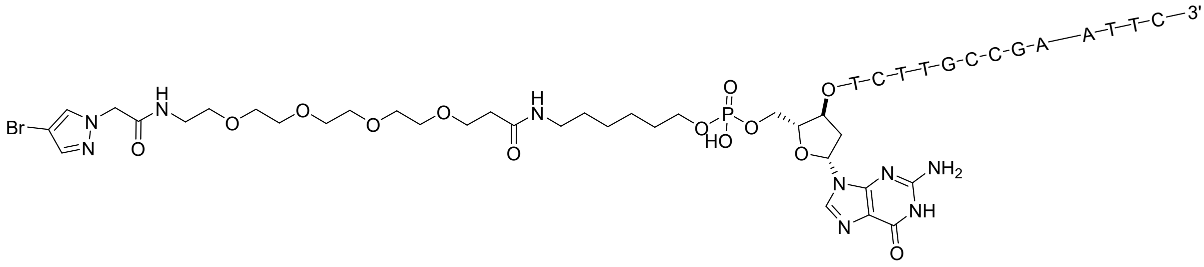

Figure S2: Mass spectrum of HP1 analysed by DNA mass spectrometry gradient A.

Expected: 4839.8887
Observed: 4839.9775*

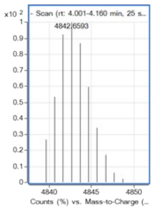

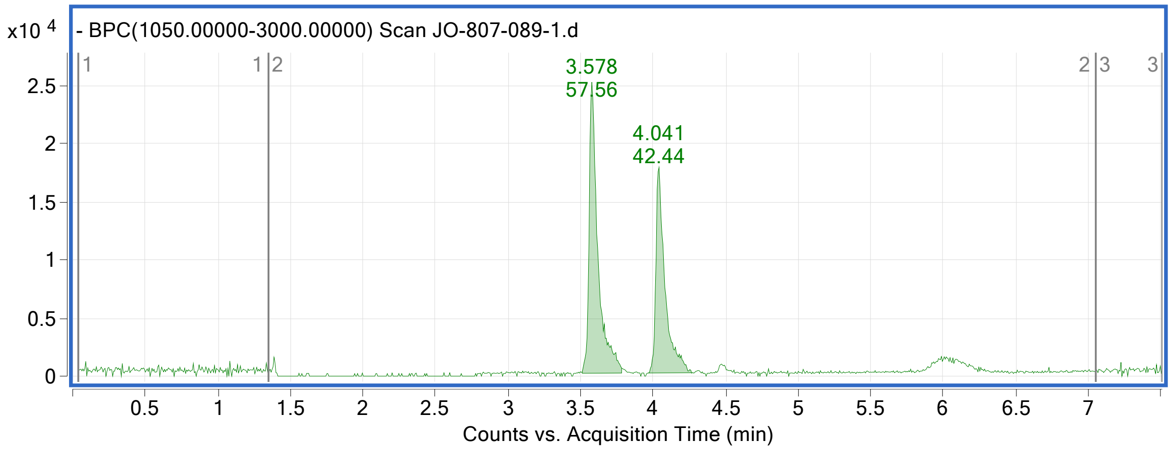

HP2 was synthesised using 12-((((9*H*-fluoren-9-yl)methoxy)carbonyl)amino)dodecanoic acid according to the general DNA headpiece procedure.

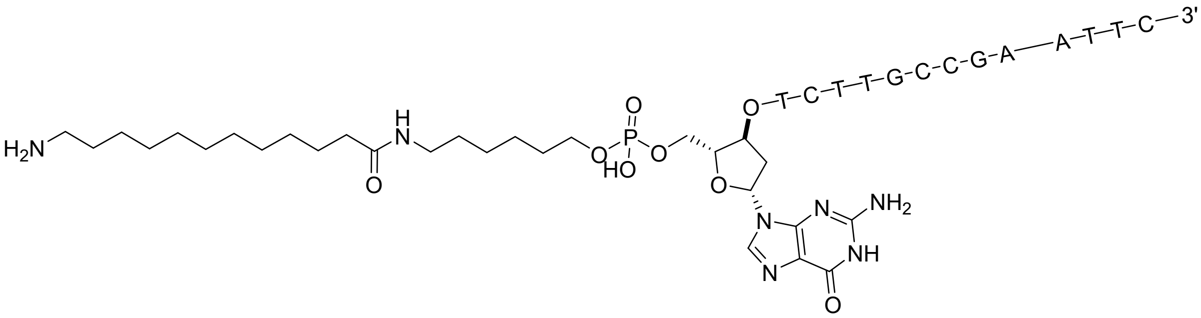

Figure S3: Mass spectrum of double-stranded HP2 analysed by DNA mass spectrometry gradient A.

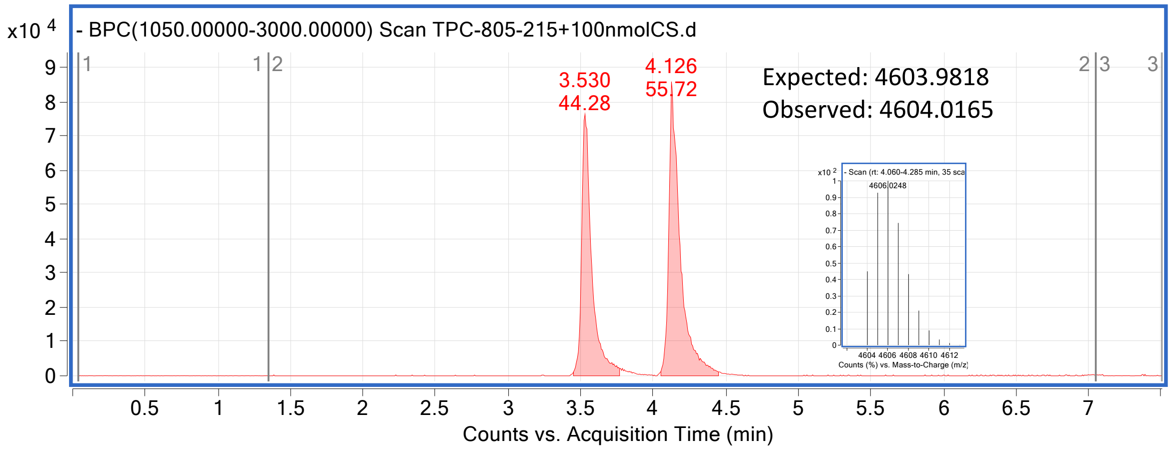

HP3 was synthesised using 16-(*tert*-butoxy)-16-oxohexadecanoic acid according to the general DNA headpiece procedure.

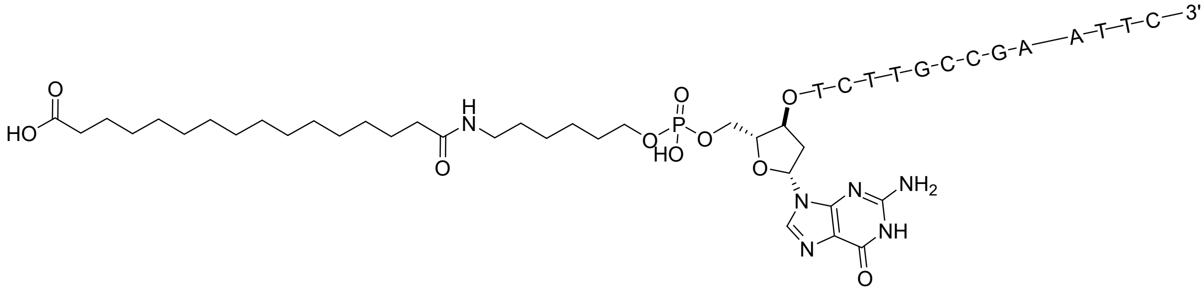

Figure S4: Mass spectrum of double-stranded HP3 analysed by DNA mass spectrometry gradient A.

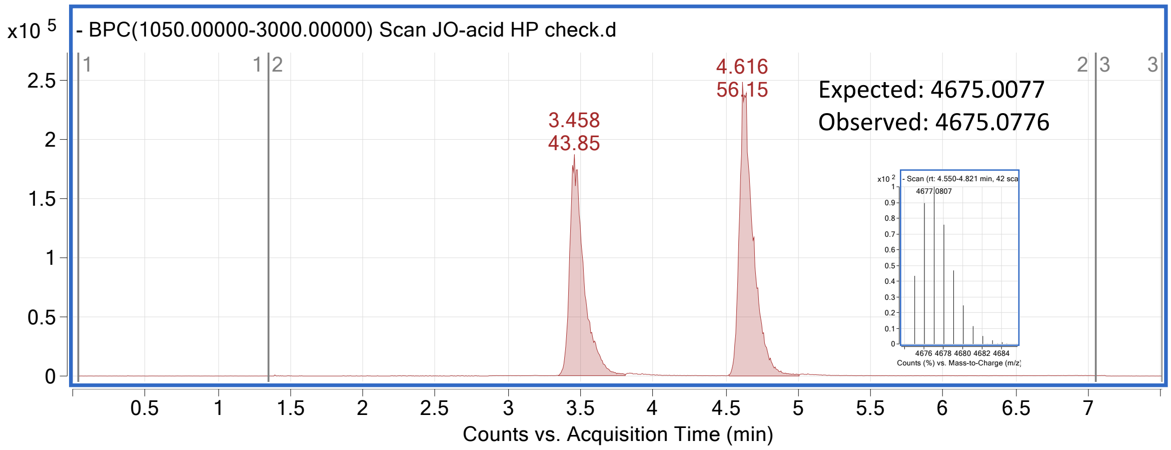

HP4 was synthesised using PEG amine HP (1–50 nmol) and 4-iodobenzoic acid according to the general on-DNA EDC/Sulfo-NHS forward amide coupling procedure.

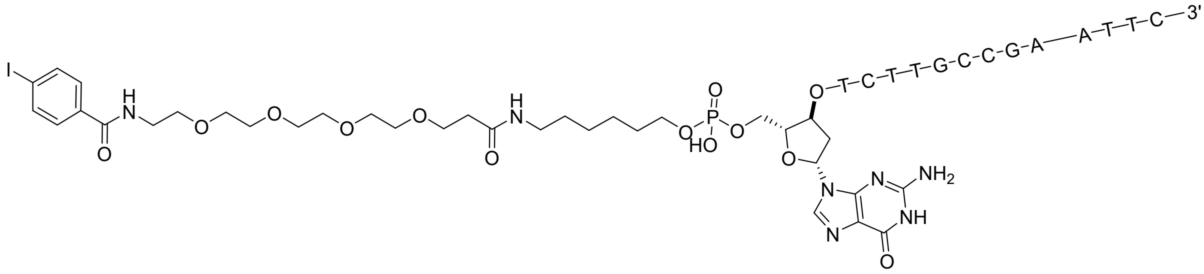

Figure S5: Mass spectrum of HP4 analysed by DNA mass spectrometry gradient A.

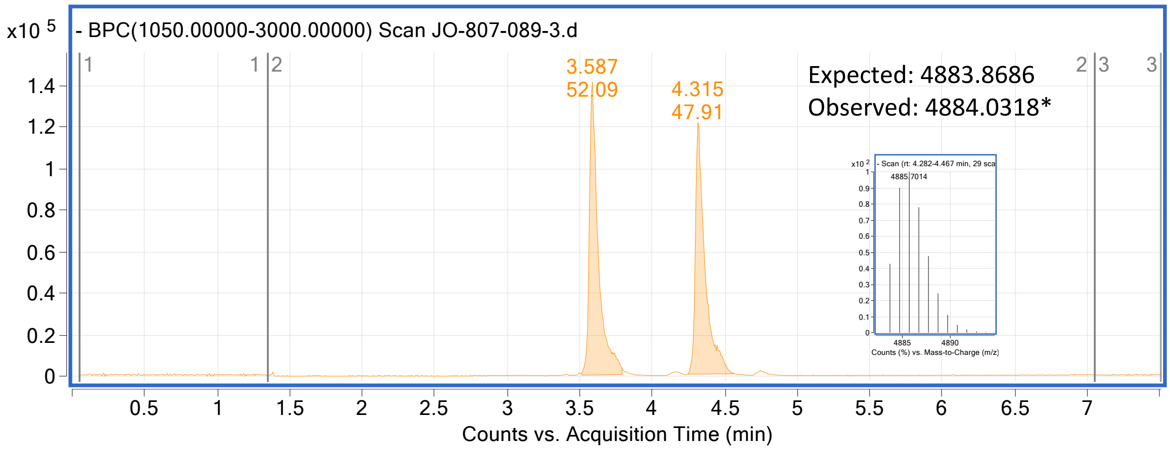

On-DNA Suzuki-Miyaura Cross-Coupling Reaction

On-DNA Suzuki-Miyaura Cross-Coupling Reaction of DNA-Conjugated Bromopyrazole HP1

Tables 1–2 Chromatograms

Compound 2 was synthesised according to general Suzuki procedure.

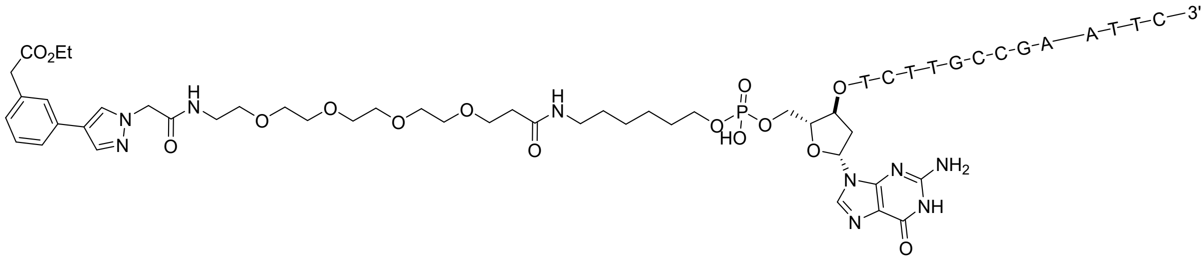

Figure S6: Mass spectrum of product 2 synthesised from HP1 and boronic acid 1 with no surfactant analysed by DNA mass spectrometry gradient A.

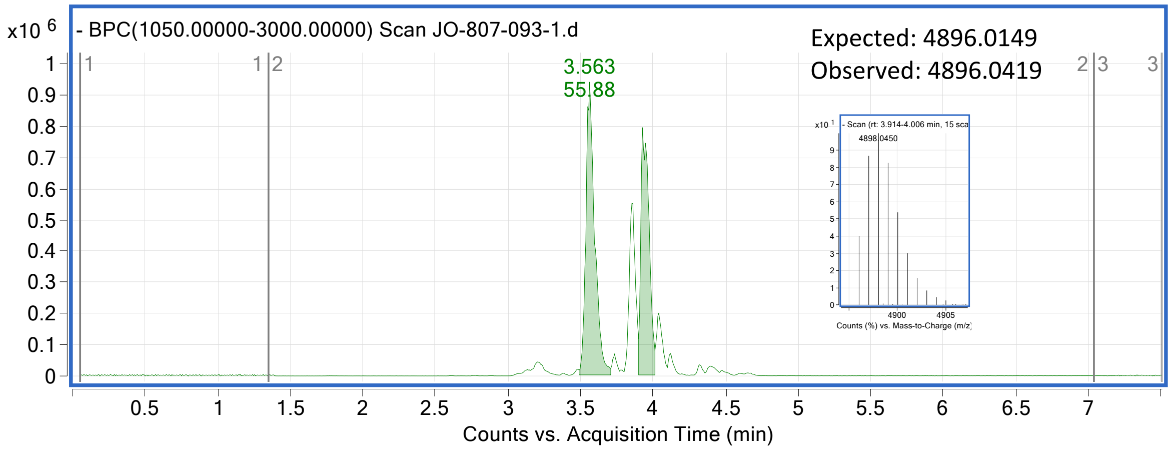

Figure S7: Mass spectrum of product 2 synthesised from HP1 and boronic acid 1 using TPGS-750-M as the surfactant analysed by DNA mass spectrometry gradient A.

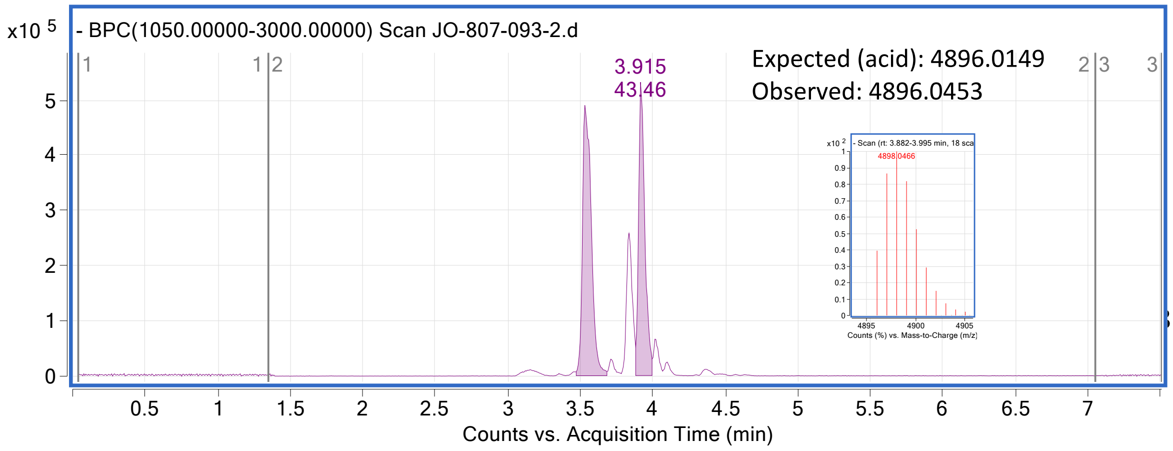

Figure S8: Mass spectrum of product 2 synthesised from HP1 and boronic acid 1 using PEG_5C_{12} as the surfactant analysed by DNA mass spectrometry gradient A.

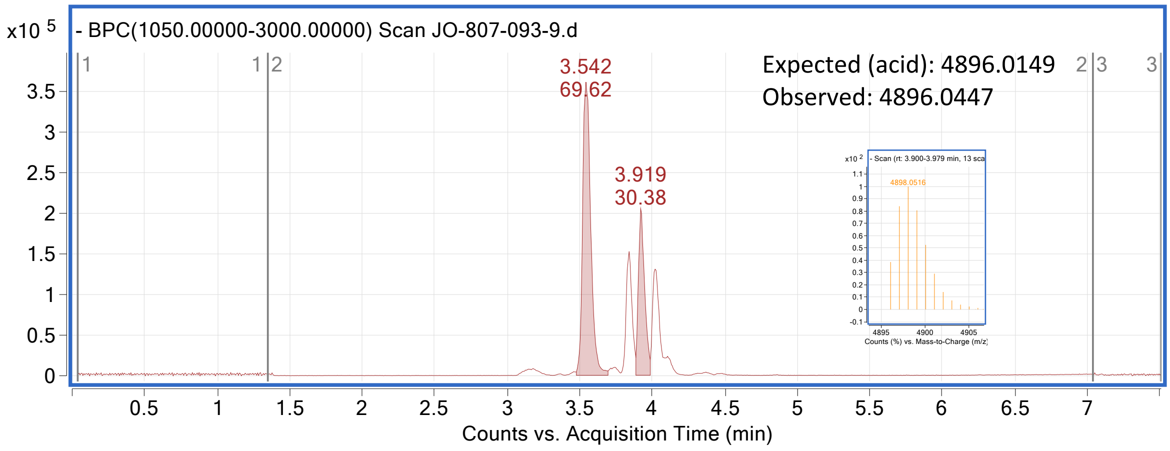

Figure S9: Mass spectrum of product 2 synthesised from HP1 and boronic acid 1 using Tween 65 as the surfactant analysed by DNA mass spectrometry gradient A.

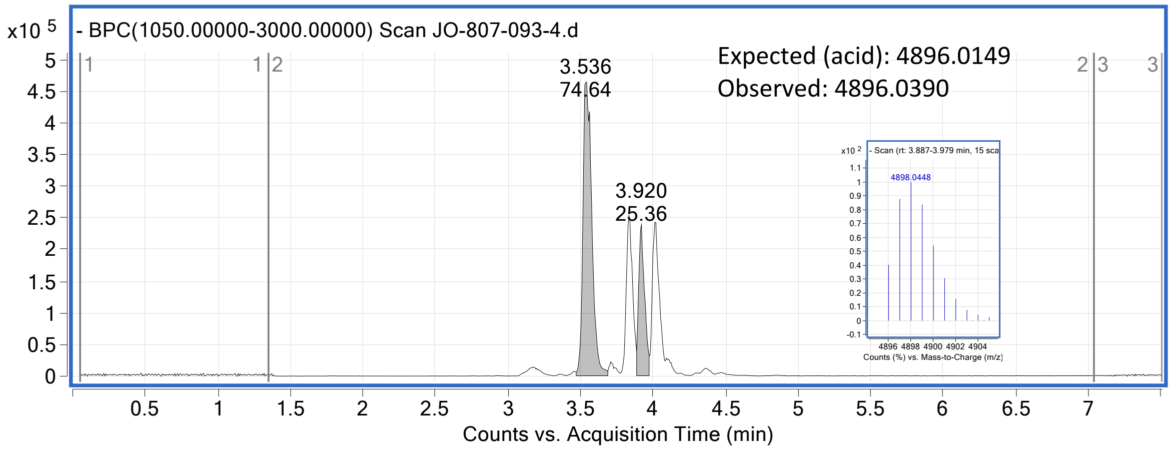

Figure S10: Mass spectrum of product 2 synthesised from HP1 and boronic acid 1 using Brij 700 as the surfactant analysed by DNA mass spectrometry gradient A.

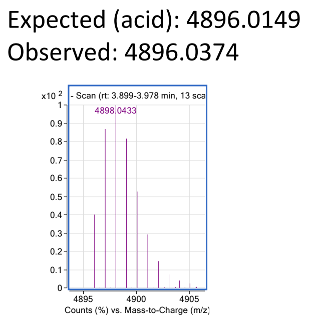

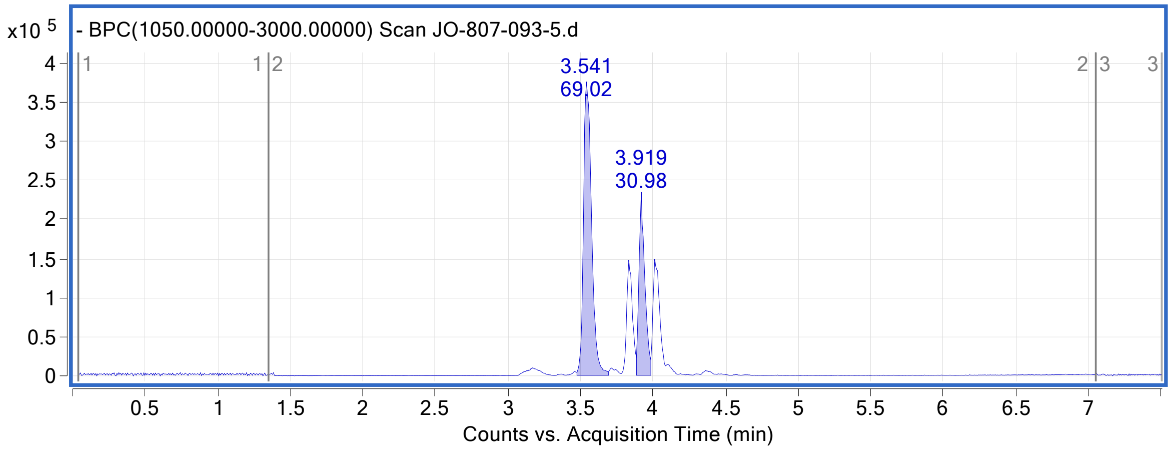

Figure S11: Mass spectrum of product 2 synthesised from HP1 and boronic acid 1 using Brij S20 as the surfactant analysed by DNA mass spectrometry gradient A.

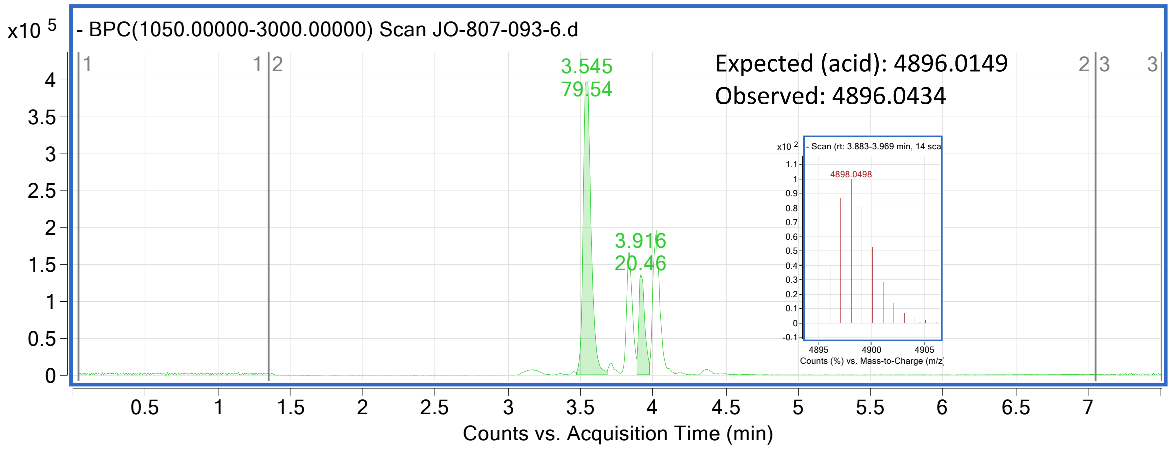

Figure S12: Mass spectrum of product 2 synthesised from HP1 and boronic acid 1 using Triton-X-405 as the surfactant analysed by DNA mass spectrometry gradient A.

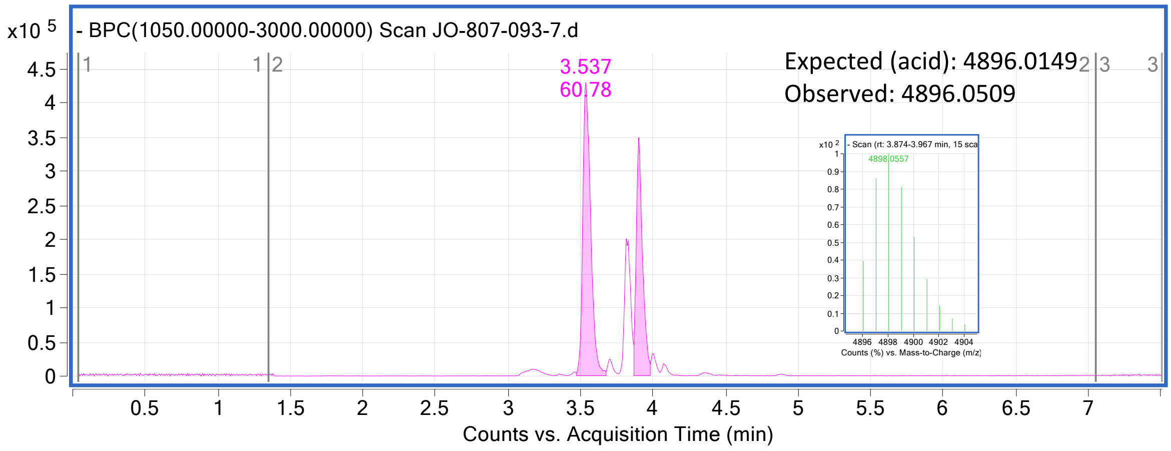

Figure S13: Mass spectrum of product 2 synthesised from HP1 and boronic acid 1 using TTAC as the surfactant analysed by DNA mass spectrometry gradient A.

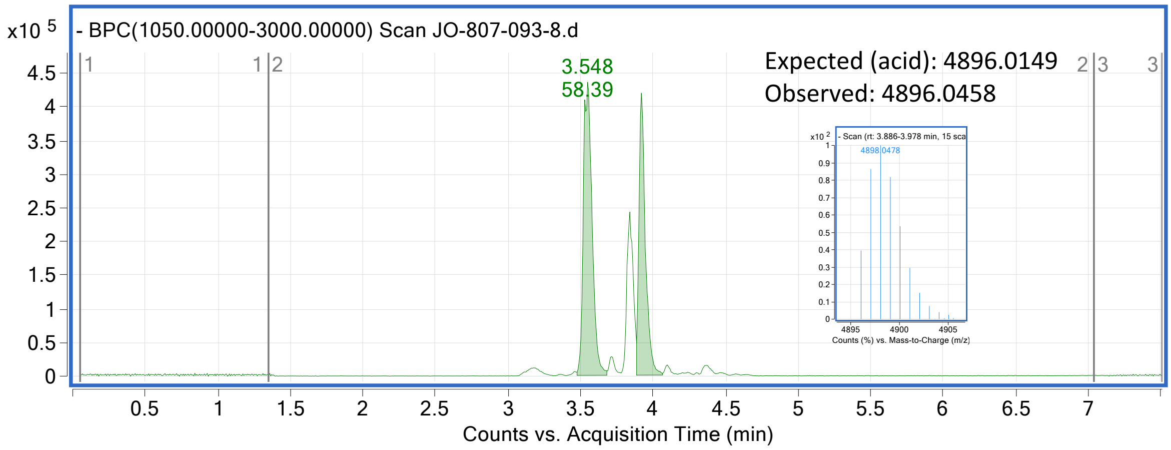

Figure S14: Mass spectrum of product 2 synthesised from HP1 and boronic acid 1 using Sulfobetaine-16 as the surfactant analysed by DNA mass spectrometry gradient A.

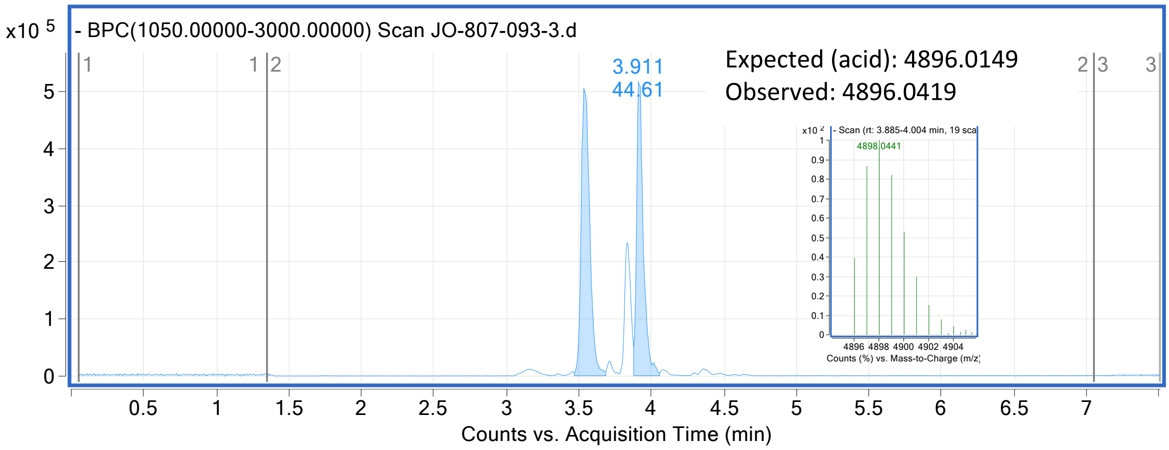

Figure S15: Mass spectrum of product 2 synthesised from HP1 and boronic acid 1 using Polysorbate 60 as the surfactant analysed by DNA mass spectrometry gradient A.

Expected (acid): 4896.0149
Observed: 4896.0477

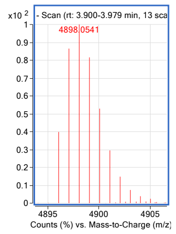

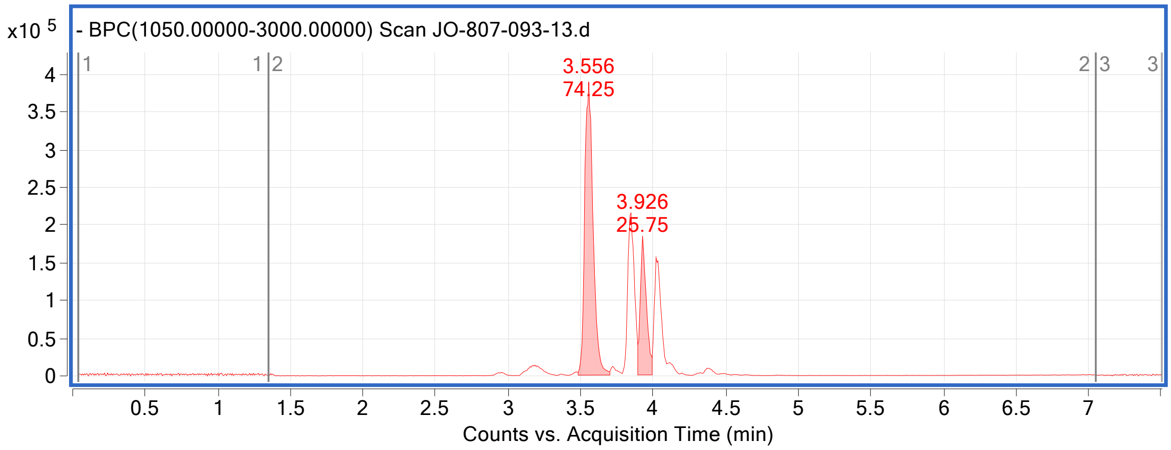

Figure S16: Mass spectrum of product 2 synthesised from HP1 and boronic acid 1 using (Lauryldimethylammonio) acetate as the surfactant analysed by DNA mass spectrometry gradient A.

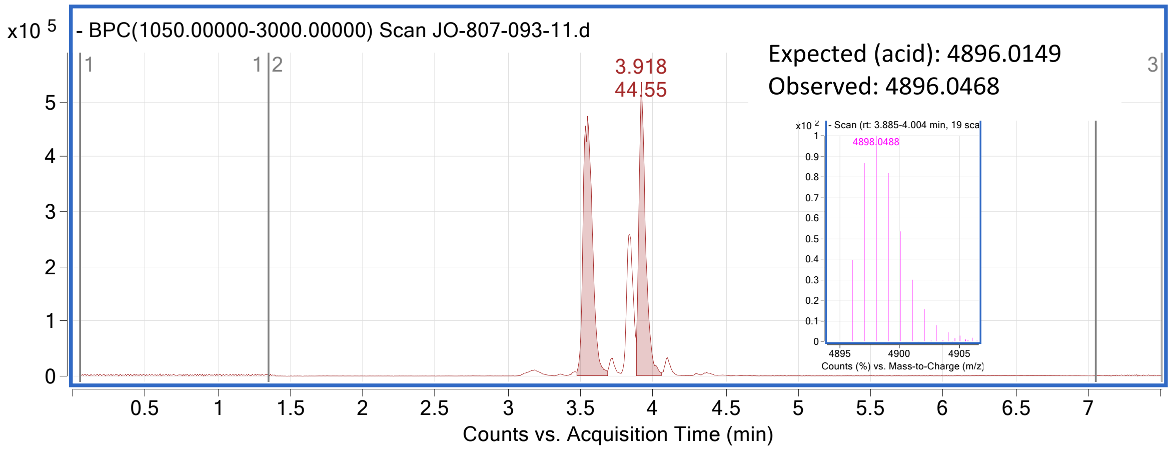

Figure S17: Mass spectrum of product 2 synthesised from HP1 and boronic acid 1 using DAPS as the surfactant analysed by DNA mass spectrometry gradient A.

Expected (acid): 4896.0149
Observed: 4896.0759

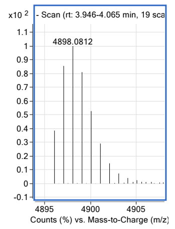

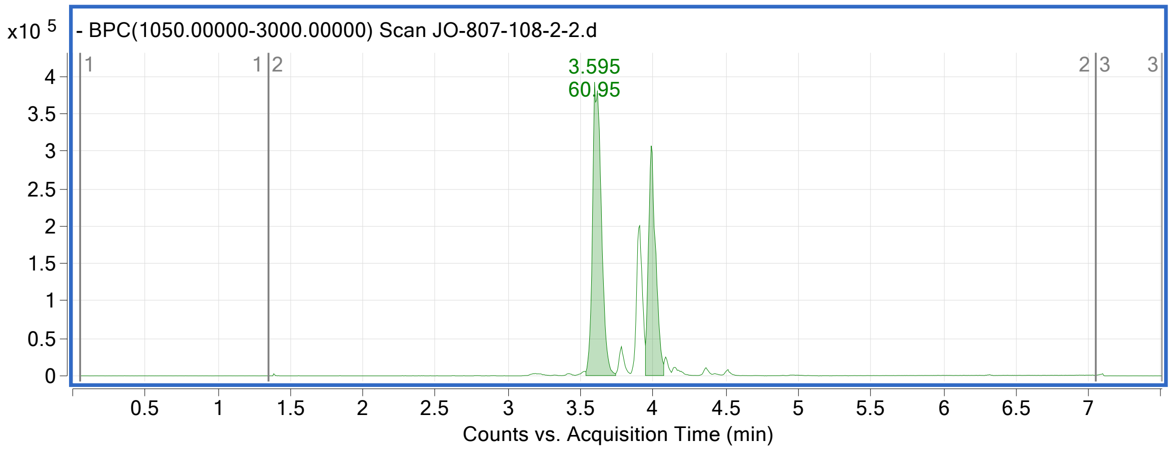

Figure S18: Mass spectrum of product 2 synthesised from HP1 and boronic acid 1 using 4-dodecylbenzenesulfonic acid as the surfactant analysed by DNA mass spectrometry gradient A.

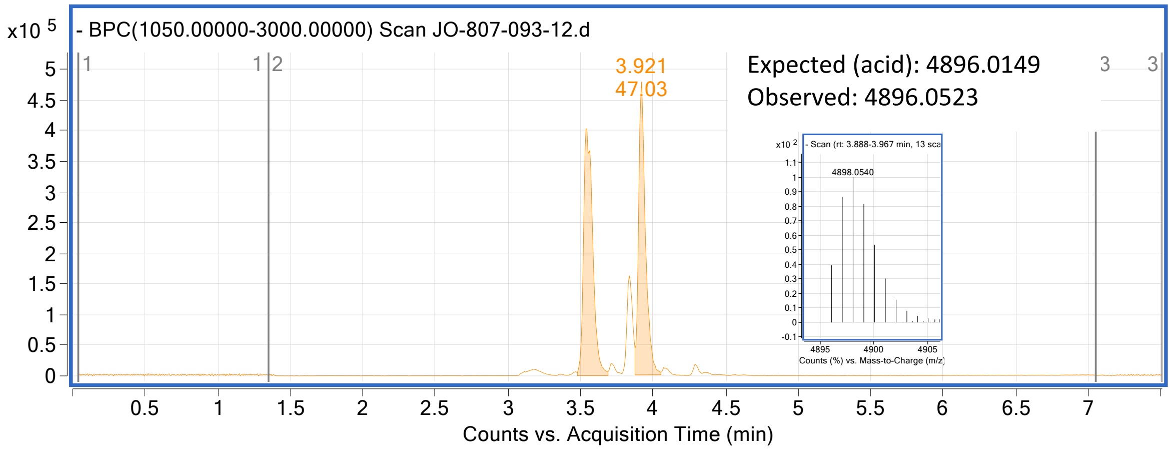

On-DNA Suzuki-Miyaura Cross-Coupling Reactions of DNA-Conjugated Iodo HP4

Table 3 Chromatograms

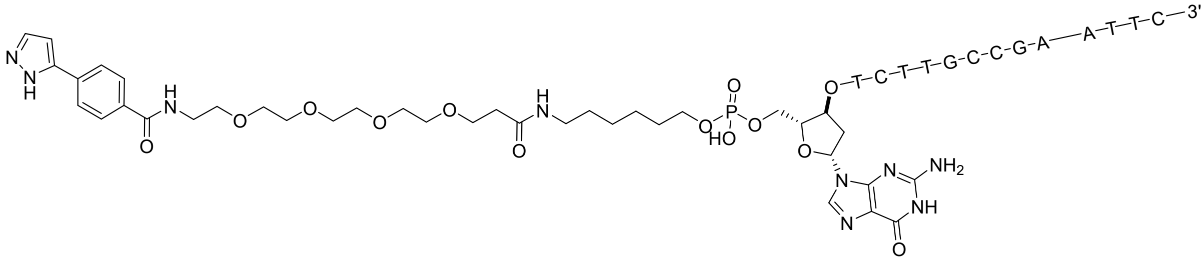

Figure S19: Mass spectrum of DNA-tagged product of Suzuki-Miyaura cross-coupling of HP4 and 5-(4,4,5,5-tetramethyl-1,3,2-dioxaborolan-2-yl)-1*H*-pyrazole using TPGS-750-M as the surfactant analysed by DNA mass spectrometry gradient A.

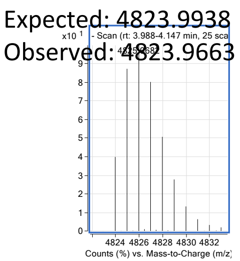

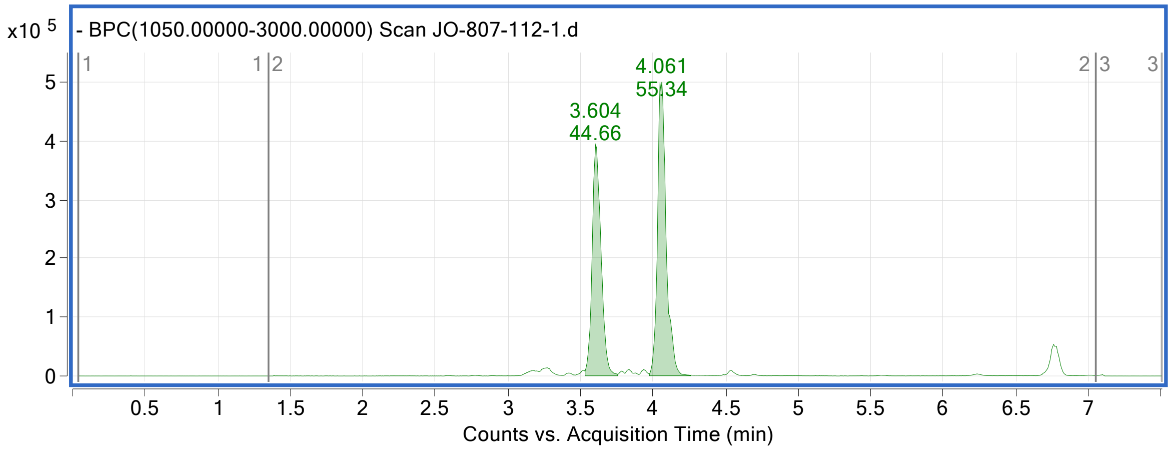

Figure S20: Mass spectrum of DNA-tagged product of Suzuki-Miyaura cross-coupling of HP4 and 5-(4,4,5,5-tetramethyl-1,3,2-dioxaborolan-2-yl)-1*H*-pyrazole using (lauryldimethylammonio)acetate as the surfactant analysed by DNA mass spectrometry gradient A.

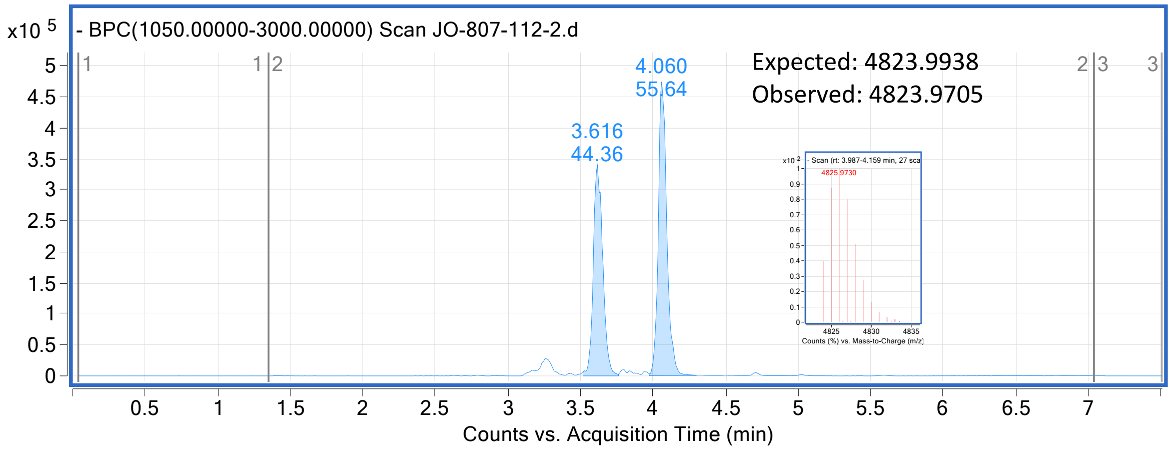

Figure S21: Mass spectrum of DNA-tagged product of Suzuki-Miyaura cross-coupling of HP4 and 5-(4,4,5,5-tetramethyl-1,3,2-dioxaborolan-2-yl)-1*H*-pyrazole using 4-dodecylbenzenesulfonic acid as the surfactant analysed by DNA mass spectrometry gradient A.

Expected: 4823.9938
Observed: 4823.9728

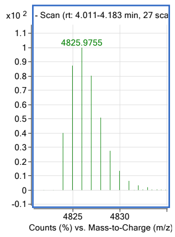

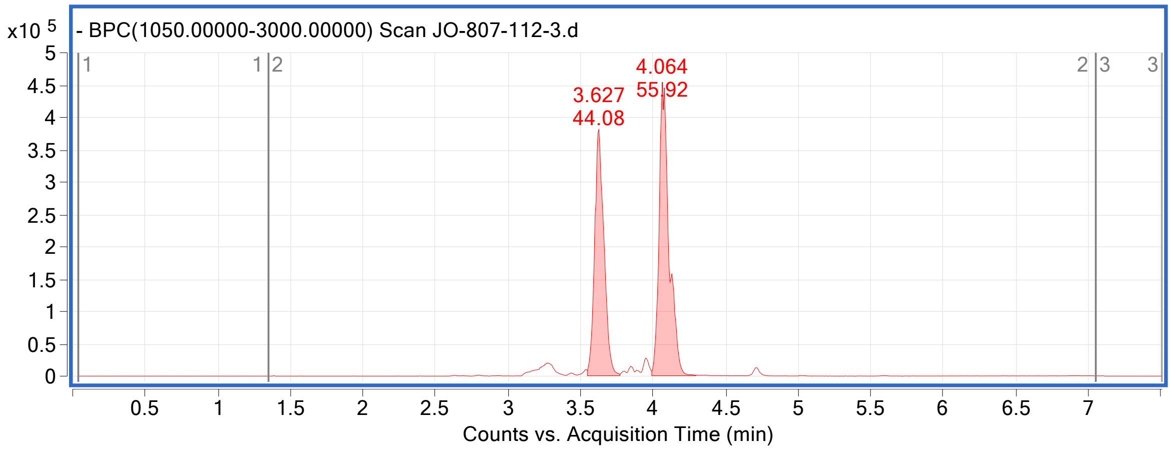

Figure S22: Mass spectrum of DNA-tagged product of Suzuki-Miyaura cross-coupling of HP4 and 5-(4,4,5,5-tetramethyl-1,3,2-dioxaborolan-2-yl)-1*H*-pyrazole using sulfobetaine-16 as the surfactant analysed by DNA mass spectrometry gradient A.

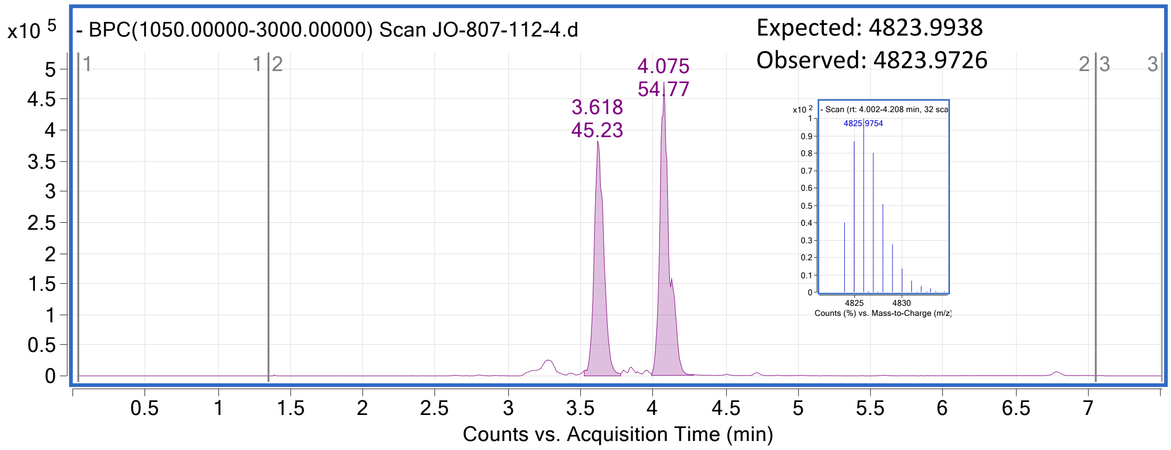

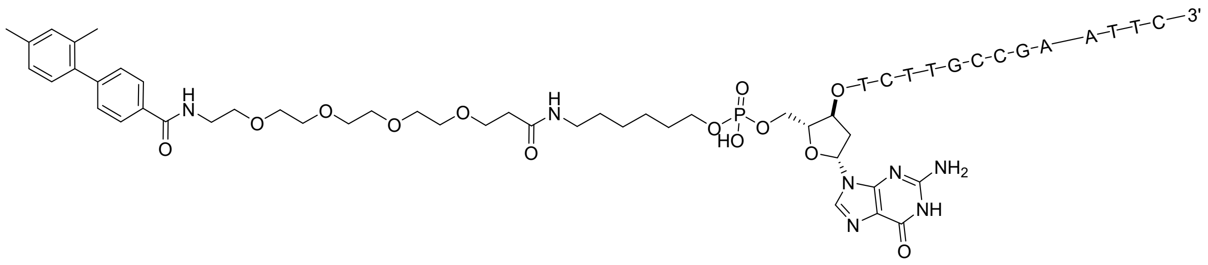

Figure S23: Mass spectrum of DNA-tagged product of Suzuki-Miyaura cross-coupling of HP4 and (2,4-dimethylphenyl)boronic acid using TPGS-750-M as the surfactant analysed by DNA mass spectrometry gradient A.

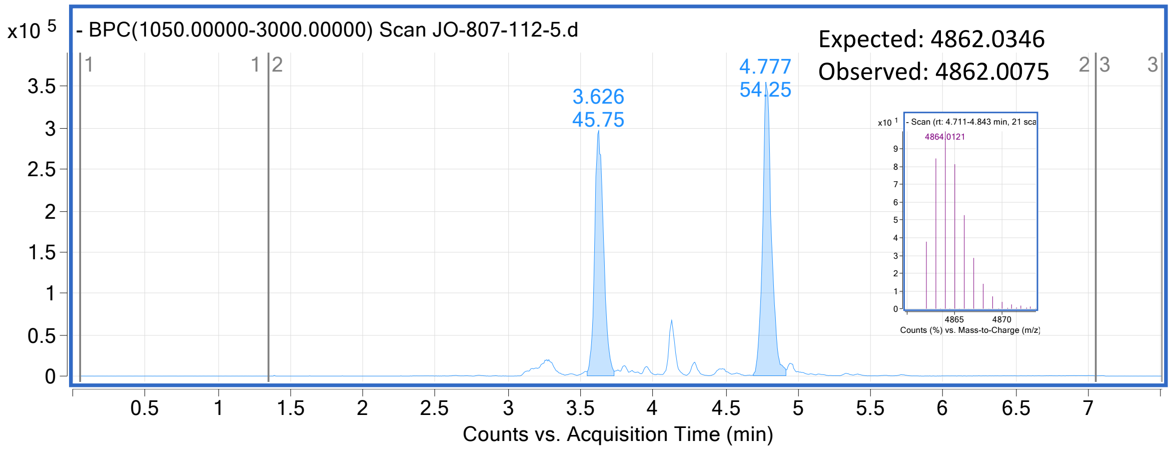

Figure S24: Mass spectrum of DNA-tagged product of Suzuki-Miyaura cross-coupling of HP4 and (2,4-dimethylphenyl)boronic acid using (lauryldimethylammonio)acetate as the surfactant analysed by DNA mass spectrometry gradient A.

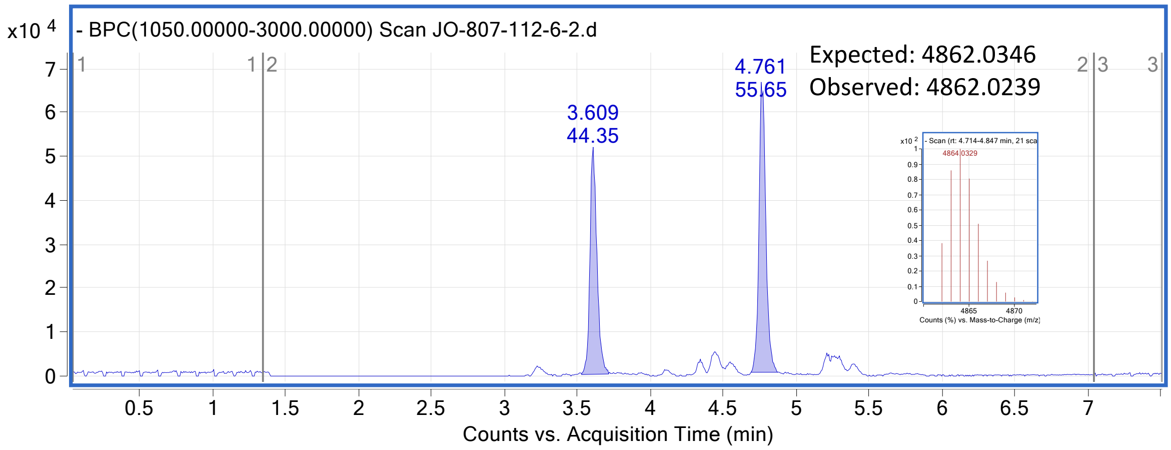

Figure S25: Mass spectrum of DNA-tagged product of Suzuki-Miyaura cross-coupling of HP4 and (2,4-dimethylphenyl)boronic acid using 4-dodecylbenzenesulfonic acid as the surfactant analysed by DNA mass spectrometry gradient A.

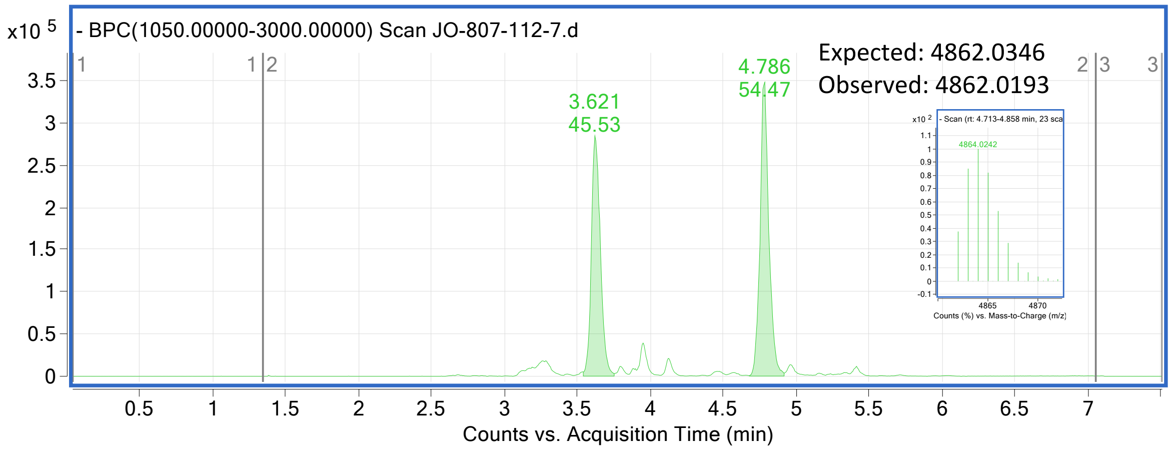

Figure S26: Mass spectrum of DNA-tagged product of Suzuki-Miyaura cross-coupling of HP4 and (2,4-dimethylphenyl)boronic acid using sulfobetaine-16 as the surfactant analysed by DNA mass spectrometry gradient A.

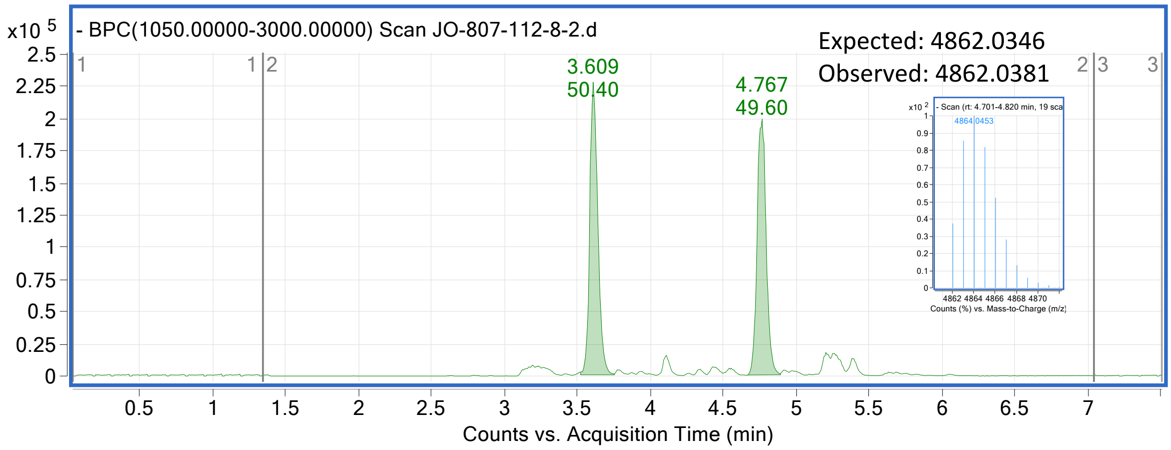

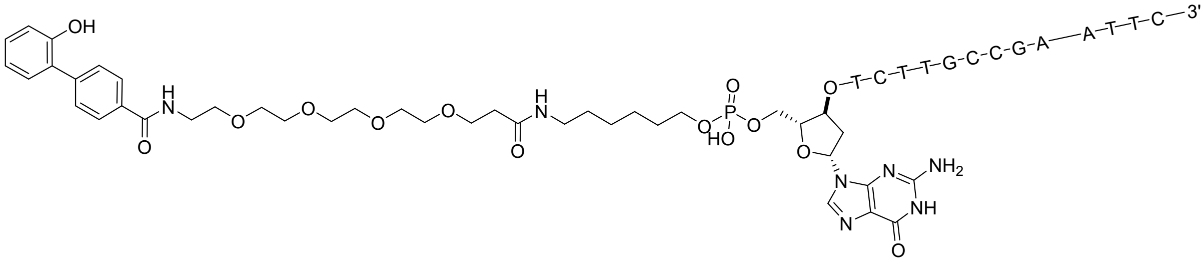

Figure S27: Mass spectrum of DNA-tagged product of Suzuki-Miyaura cross-coupling of HP4 and (2-hydroxyphenyl)boronic acid using TPGS-750-M as the surfactant analysed by DNA mass spectrometry gradient A.

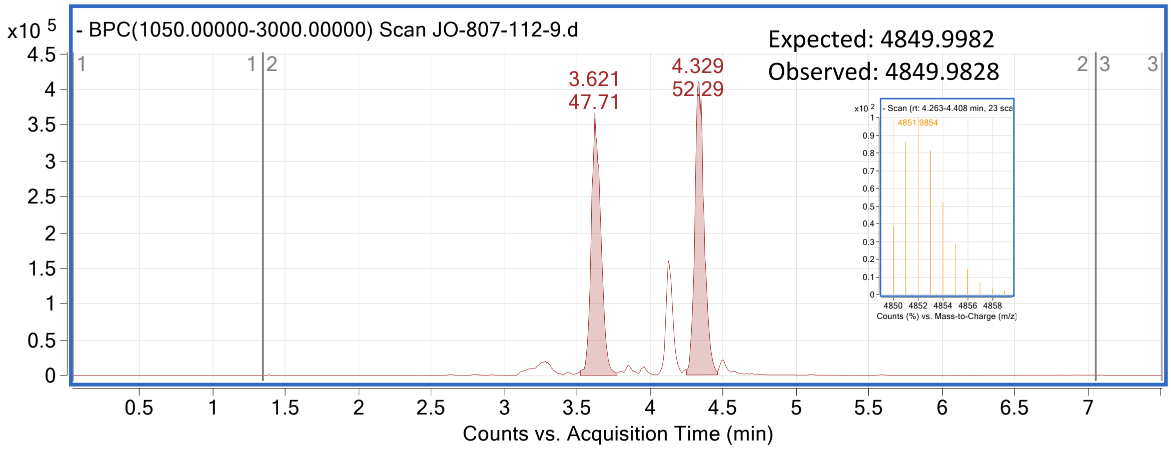

Figure S28: Mass spectrum of DNA-tagged product of Suzuki-Miyaura cross-coupling of HP4 and (2-hydroxyphenyl)boronic acid using (lauryldimethylammonio)acetate as the surfactant analysed by DNA mass spectrometry gradient A.

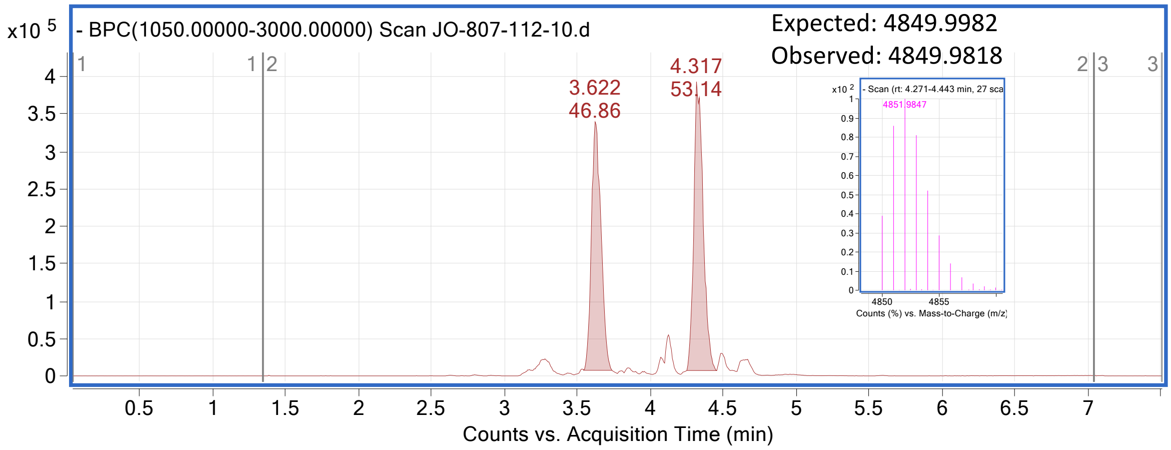

Figure S29: Mass spectrum of DNA-tagged product of Suzuki-Miyaura cross-coupling of HP4 and (2-hydroxyphenyl)boronic acid using 4-dodecylbenzenesulfonic acid as the surfactant analysed by DNA mass spectrometry gradient A.

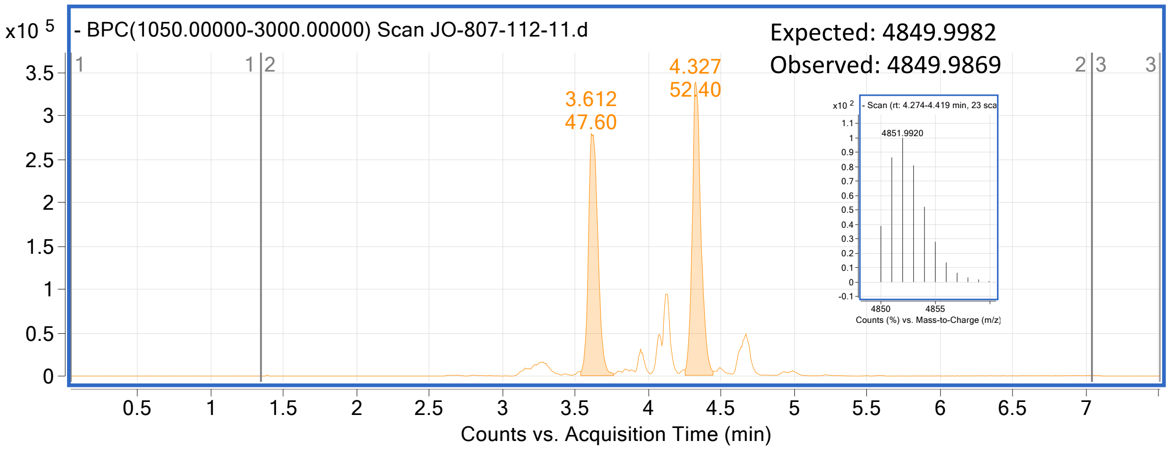

Figure S30: Mass spectrum of DNA-tagged product of Suzuki-Miyaura cross-coupling of HP4 and (2-hydroxyphenyl)boronic acid using sulfobetaine-16 as the surfactant analysed by DNA mass spectrometry gradient A.

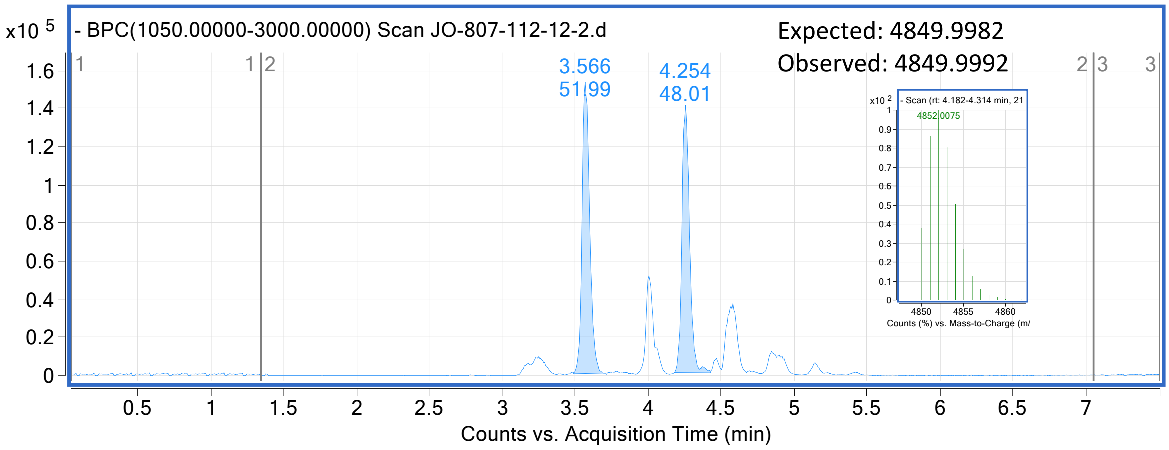

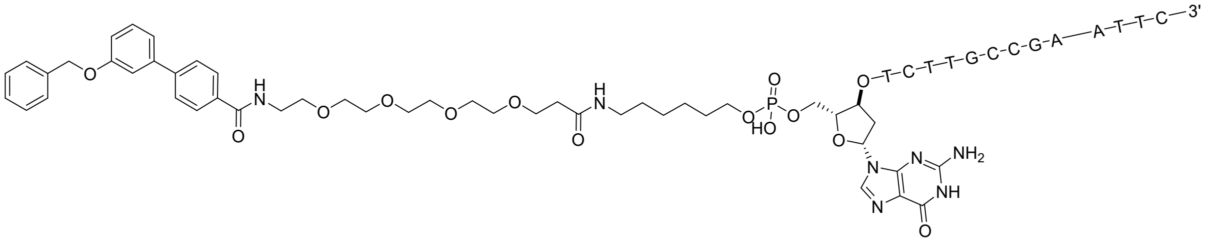

Figure S31: Mass spectrum of DNA-tagged product of Suzuki-Miyaura cross-coupling of HP4 and (3-(benzyloxy)phenyl)boronic acid using TPGS-750-M as the surfactant analysed by DNA mass spectrometry gradient A.

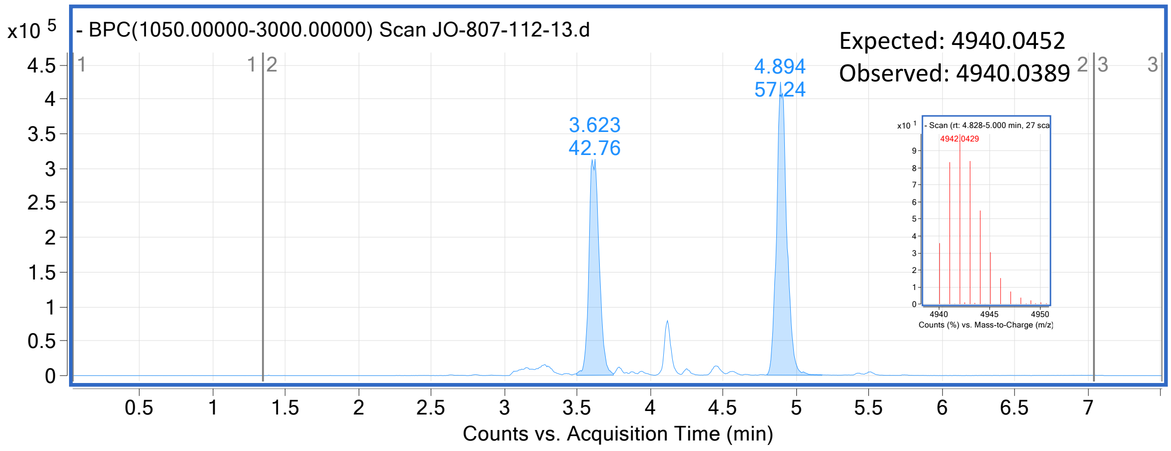

Figure S32: Mass spectrum of DNA-tagged product of Suzuki-Miyaura cross-coupling of HP4 and (3-(benzyloxy)phenyl)boronic acid using (lauryldimethylammonio)acetate as the surfactant analysed by DNA mass spectrometry gradient A.

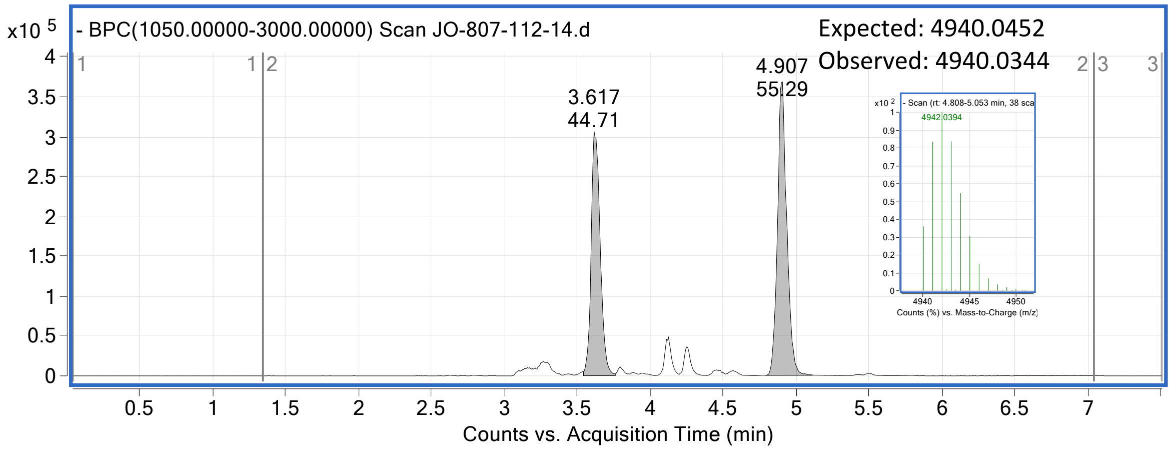

Figure S33: Mass spectrum of DNA-tagged product of Suzuki-Miyaura cross-coupling of HP4 and (3-(benzyloxy)phenyl)boronic acid using 4-dodecylbenzenesulfonic acid as the surfactant analysed by DNA mass spectrometry gradient A.

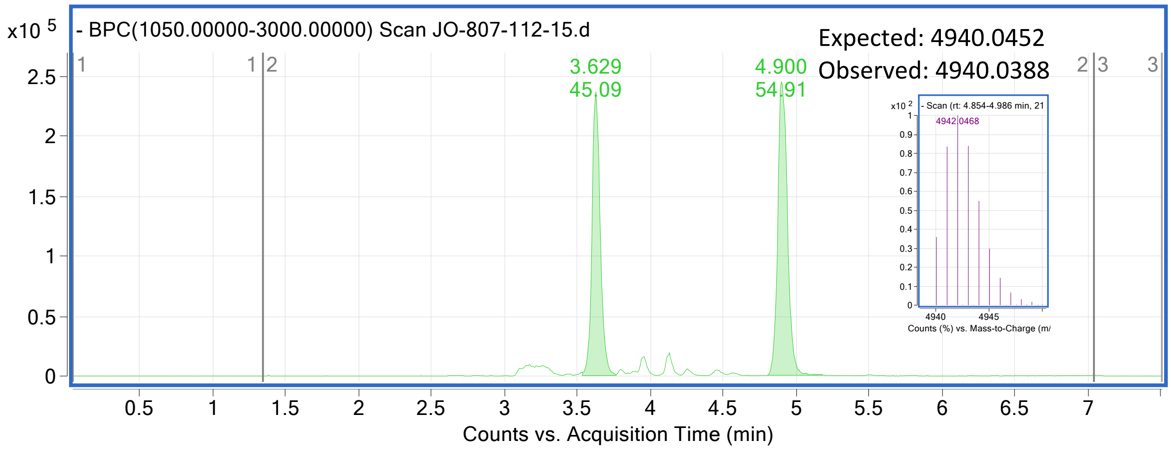

Figure S34: Mass spectrum of DNA-tagged product of Suzuki-Miyaura cross-coupling of HP4 and (3-(benzyloxy)phenyl)boronic acid using sulfobetaine-16 as the surfactant analysed by DNA mass spectrometry gradient A.

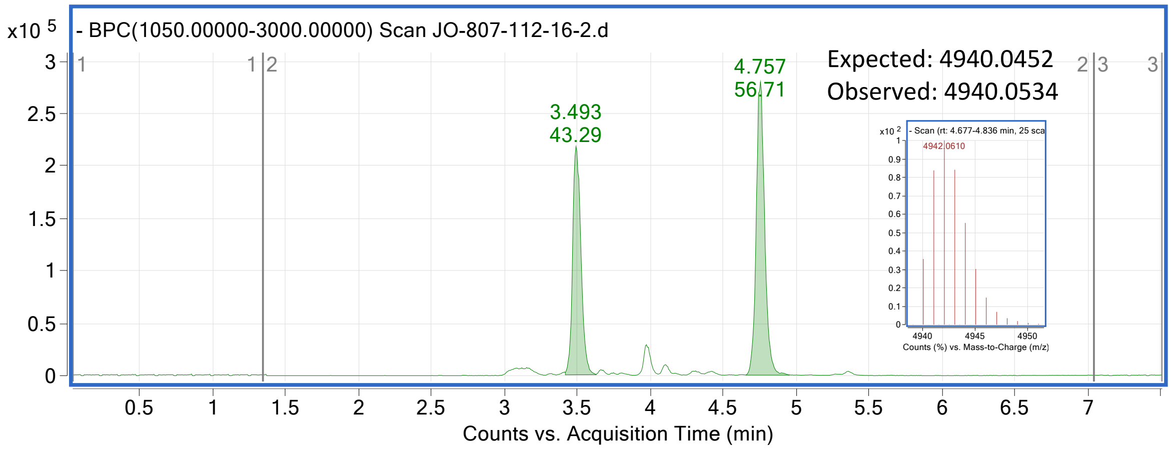

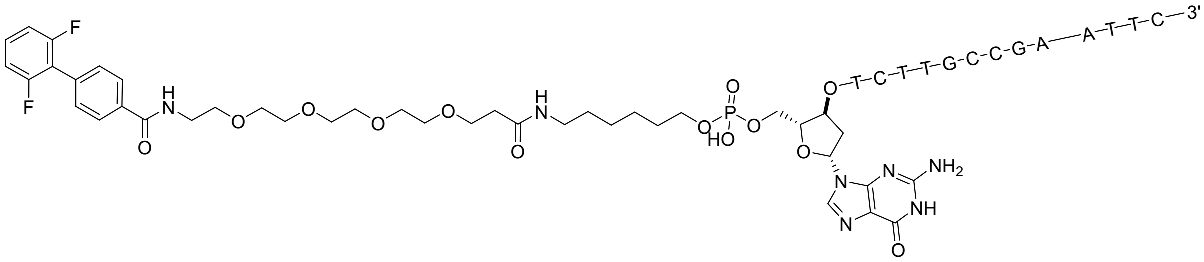

Figure S35: Mass spectrum of DNA-tagged product of Suzuki-Miyaura cross-coupling of HP4 and (2,6-difluorophenyl)boronic acid using TPGS-750-M as the surfactant analysed by DNA mass spectrometry gradient A.

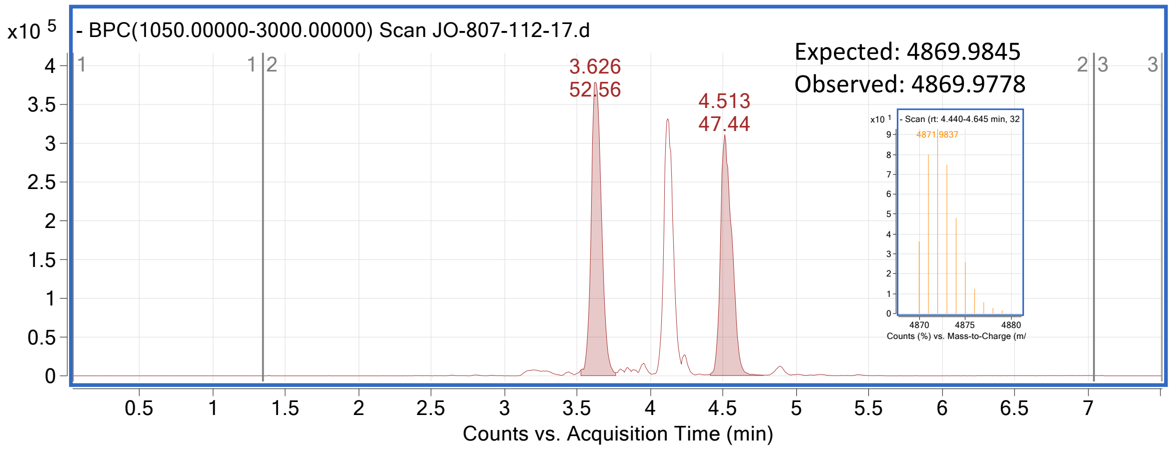

Figure S36: Mass spectrum of DNA-tagged product of Suzuki-Miyaura cross-coupling of HP4 and (2,6-difluorophenyl)boronic acid using (lauryldimethylammonio)acetate as the surfactant analysed by DNA mass spectrometry gradient A.

Expected: 4869.9845
Observed: 4869.9685

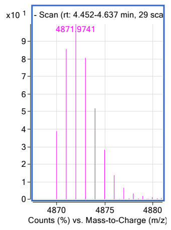

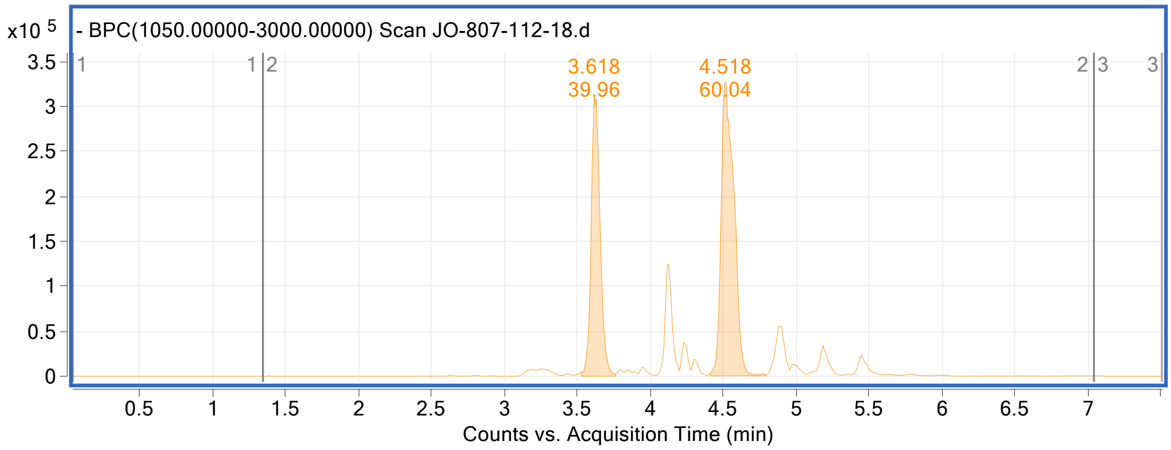

Figure S37: Mass spectrum of DNA-tagged product of Suzuki-Miyaura cross-coupling of HP4 and (2,6-difluorophenyl)boronic acid using 4-dodecylbenzenesulfonic acid as the surfactant analysed by DNA mass spectrometry gradient A.

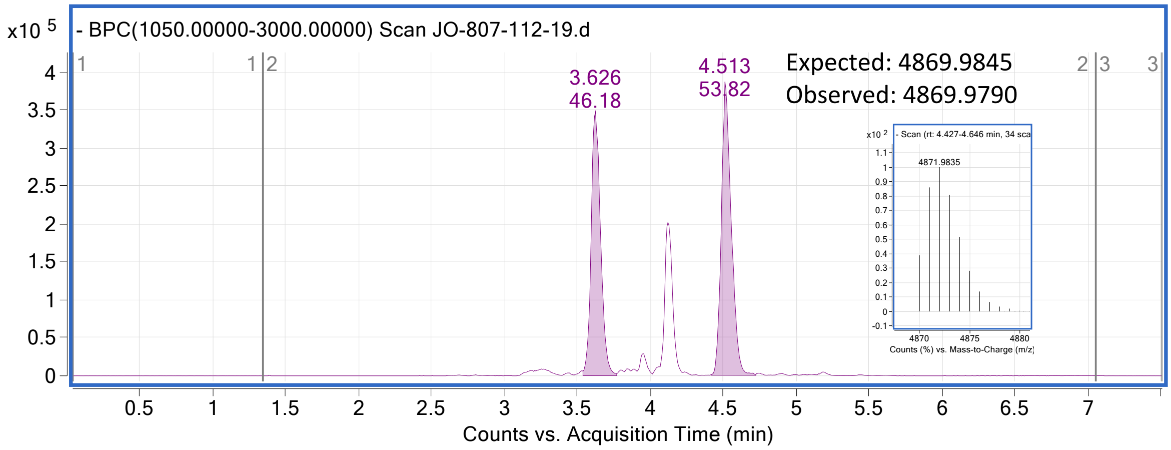

Figure S38: Mass spectrum of DNA-tagged product of Suzuki-Miyaura cross-coupling of HP4 and (2,6-difluorophenyl)boronic acid using sulfobetaine-16 as the surfactant analysed by DNA mass spectrometry gradient A.

Expected: 4869.9845
Observed: 4869.9912

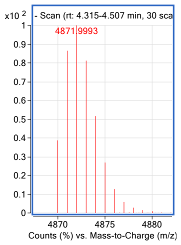

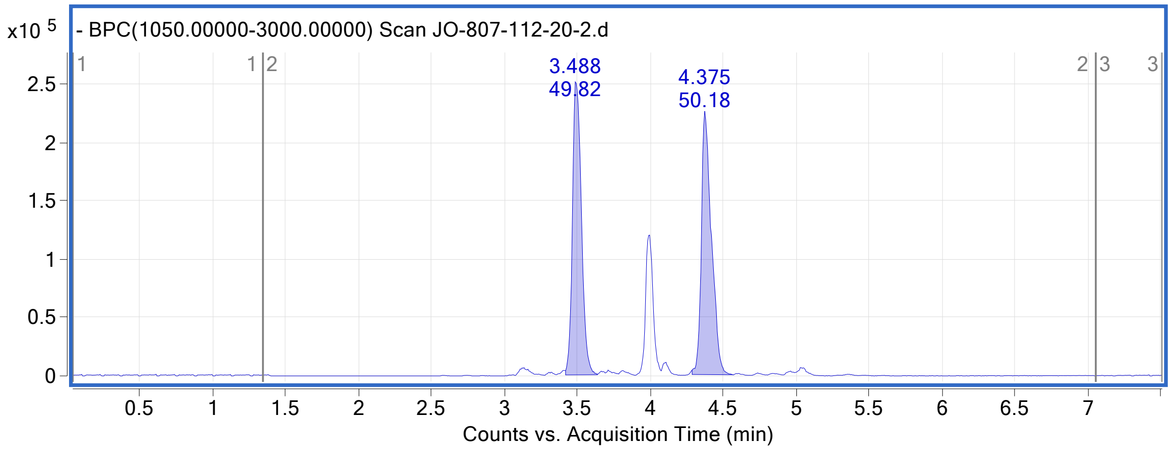

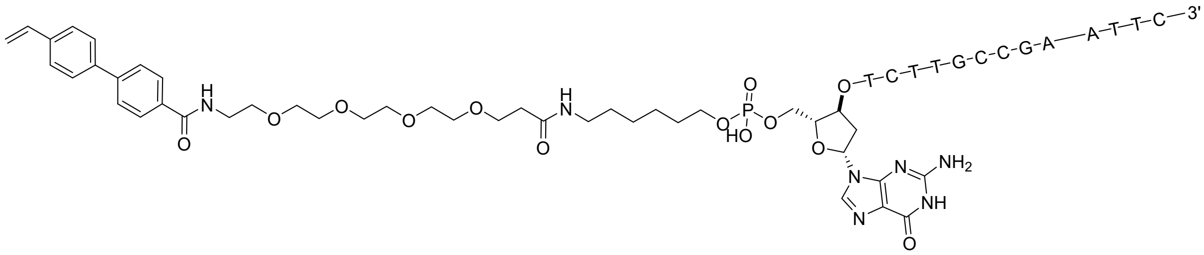

Figure S39: Mass spectrum of DNA-tagged product of Suzuki-Miyaura cross-coupling of HP4 and (4-vinylphenyl)boronic acid using TPGS-750-M as the surfactant analysed by DNA mass spectrometry gradient A.

Expected: 4860.0190
Observed: 4860.0176

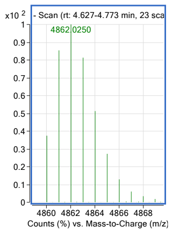

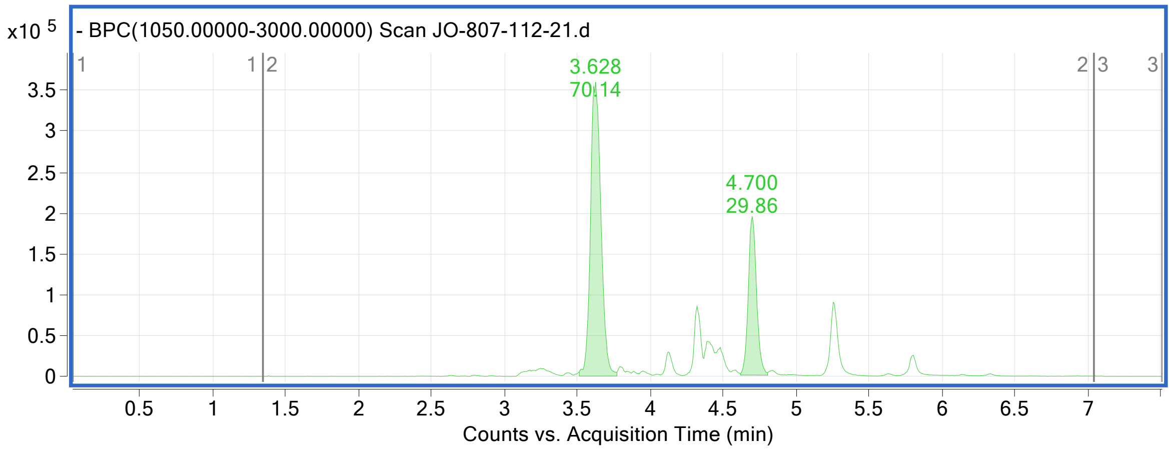

Figure S40: Mass spectrum of DNA-tagged product of Suzuki-Miyaura cross-coupling of HP4 and (4-vinylphenyl)boronic acid using (lauryldimethylammonio)acetate as the surfactant analysed by DNA mass spectrometry gradient A.

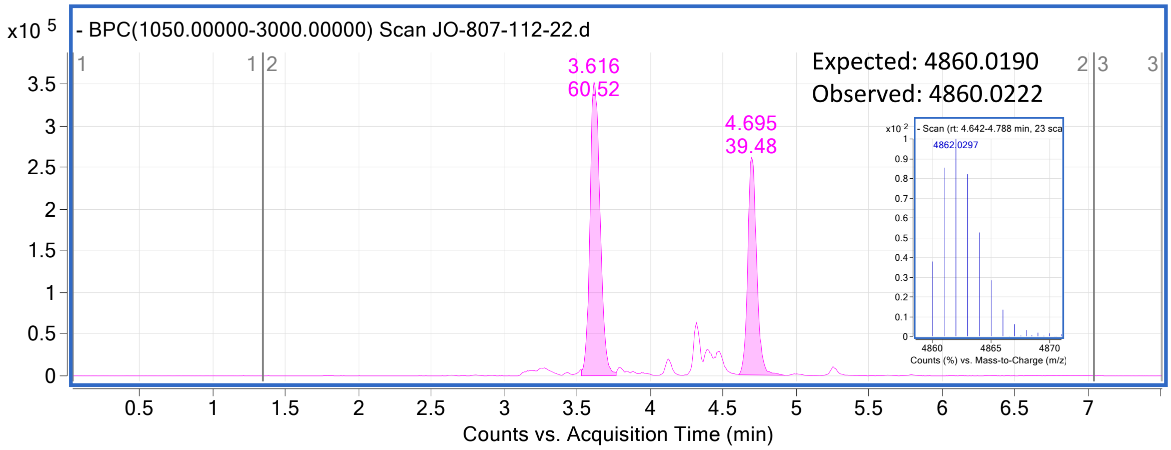

Figure S41: Mass spectrum of DNA-tagged product of Suzuki-Miyaura cross-coupling of HP4 and (4-vinylphenyl)boronic acid using 4-dodecylbenzenesulfonic acid as the surfactant analysed by DNA mass spectrometry gradient A.

Expected: 4860.0190
Observed: 4860.0351

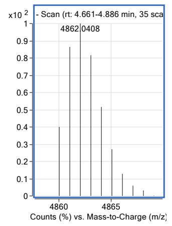

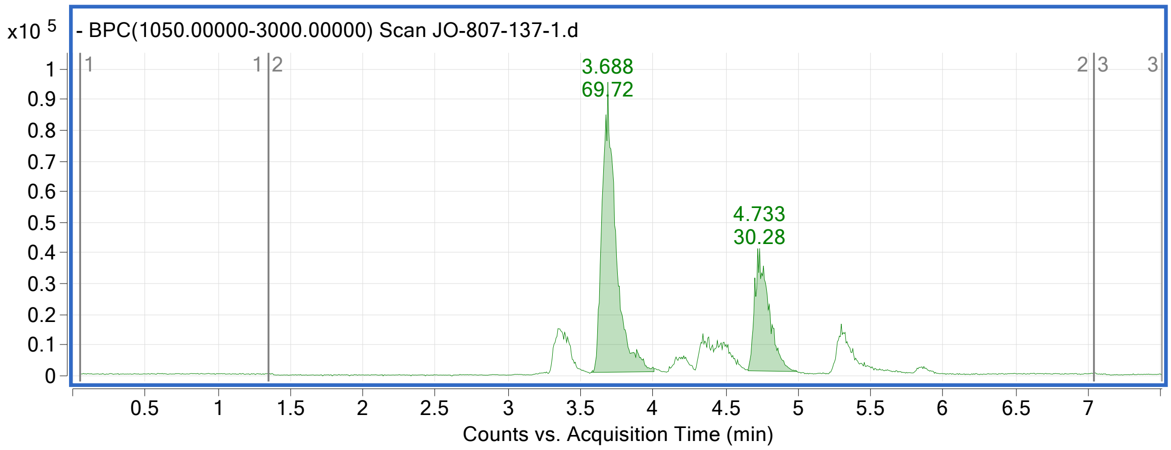

Figure S42: Mass spectrum of DNA-tagged product of Suzuki-Miyaura cross-coupling of HP4 and (4-vinylphenyl)boronic acid using sulfobetaine-16 as the surfactant analysed by DNA mass spectrometry gradient A.

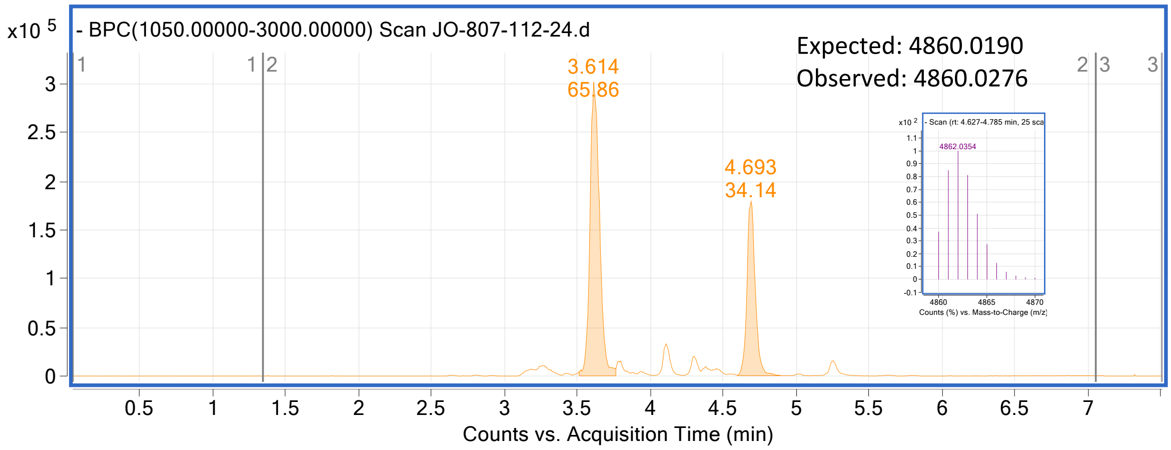

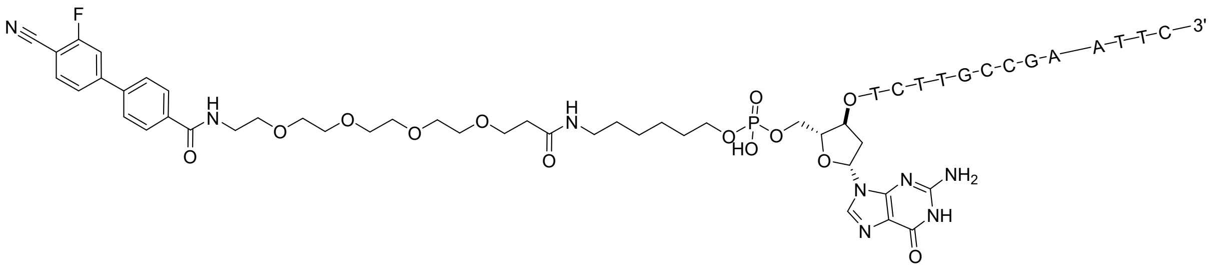

Figure S43: Mass spectrum of DNA-tagged product of Suzuki-Miyaura cross-coupling of HP4 and (4-cyano-3-fluorophenyl)boronic acid using TPGS-750-M as the surfactant analysed by DNA mass spectrometry gradient A.

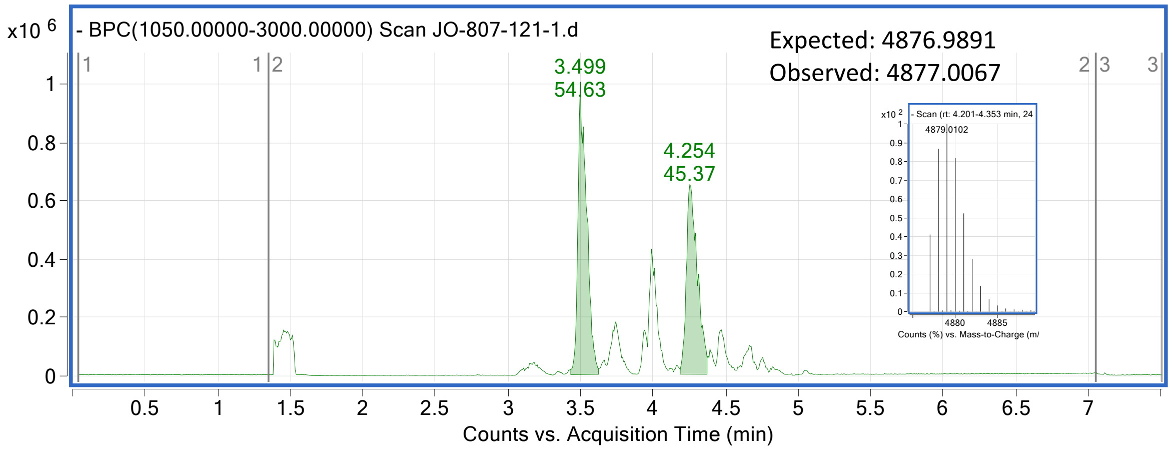

Figure S44: Mass spectrum of DNA-tagged product of Suzuki-Miyaura cross-coupling of HP4 and (4-cyano-3-fluorophenyl)boronic acid using (lauryldimethylammonio)acetate as the surfactant analysed by DNA mass spectrometry gradient A.

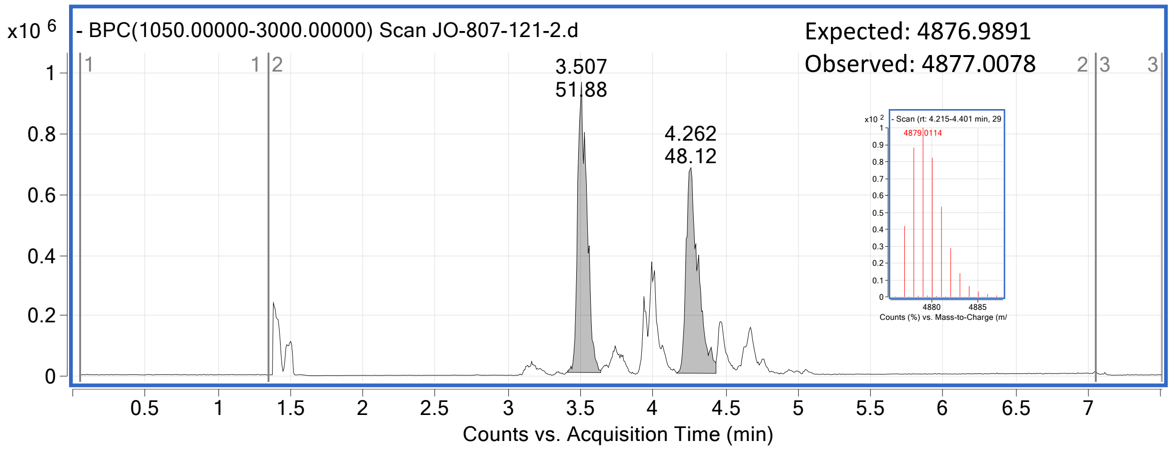

Figure S45: Mass spectrum of DNA-tagged product of Suzuki-Miyaura cross-coupling of HP4 and (4-cyano-3-fluorophenyl)boronic acid using 4-dodecylbenzenesulfonic acid as the surfactant analysed by DNA mass spectrometry gradient A.

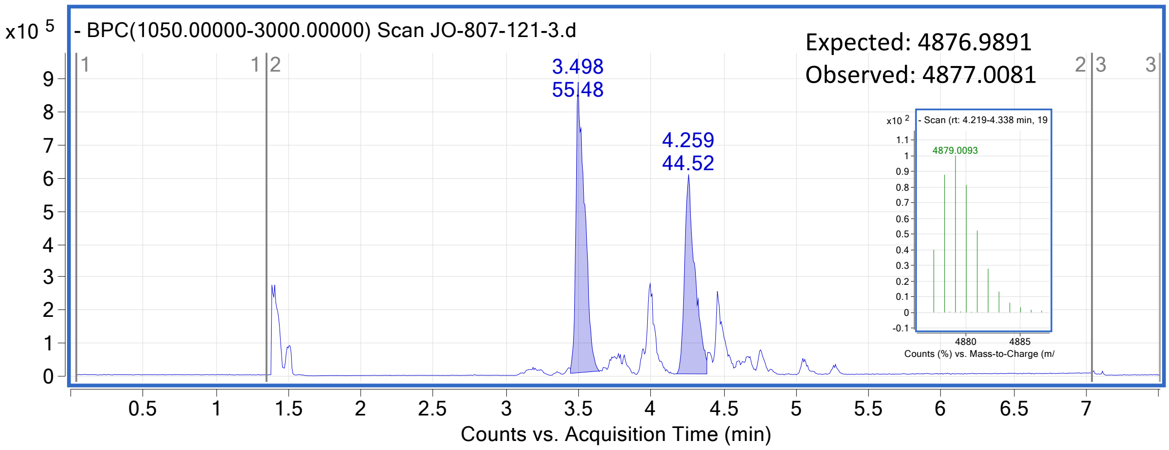

Figure S46: Mass spectrum of DNA-tagged product of Suzuki-Miyaura cross-coupling of HP4 and (4-cyano-3-fluorophenyl)boronic acid using sulfobetaine-16 as the surfactant analysed by DNA mass spectrometry gradient A.

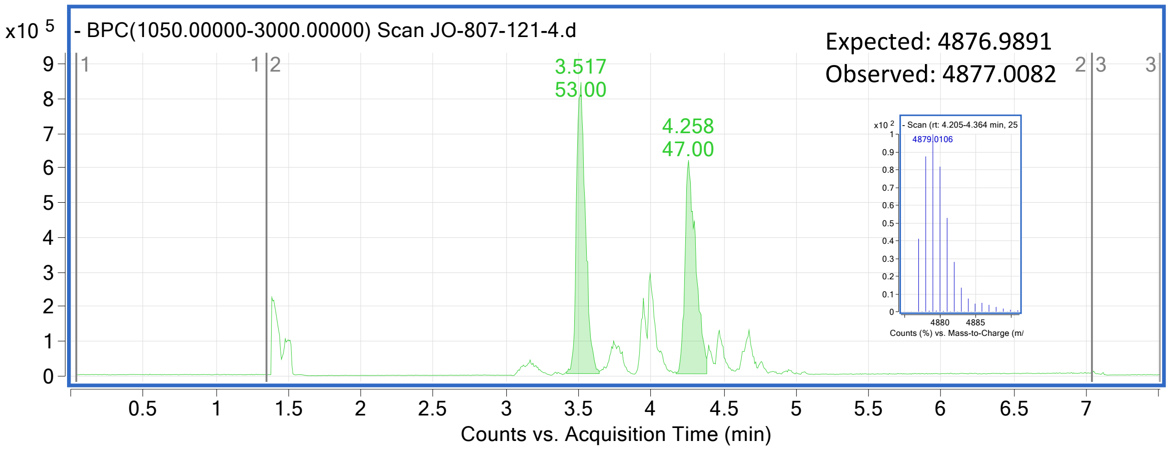

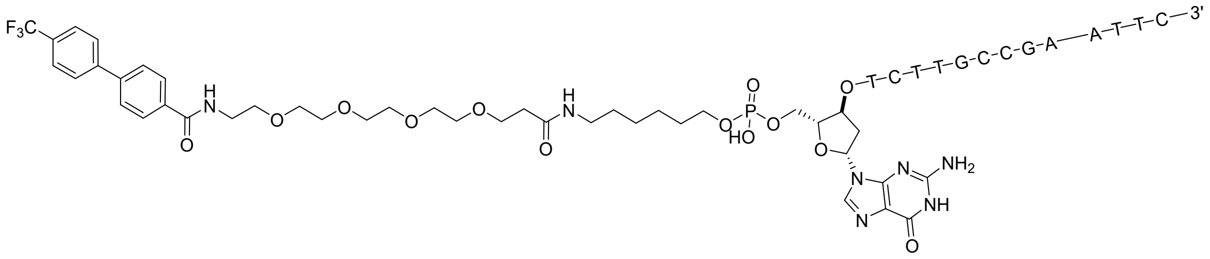

Figure S47: Mass spectrum of DNA-tagged product of Suzuki-Miyaura cross-coupling of HP4 and (4-(trifluoromethyl)phenyl)boronic acid using TPGS-750-M as the surfactant analysed by DNA mass spectrometry gradient A.

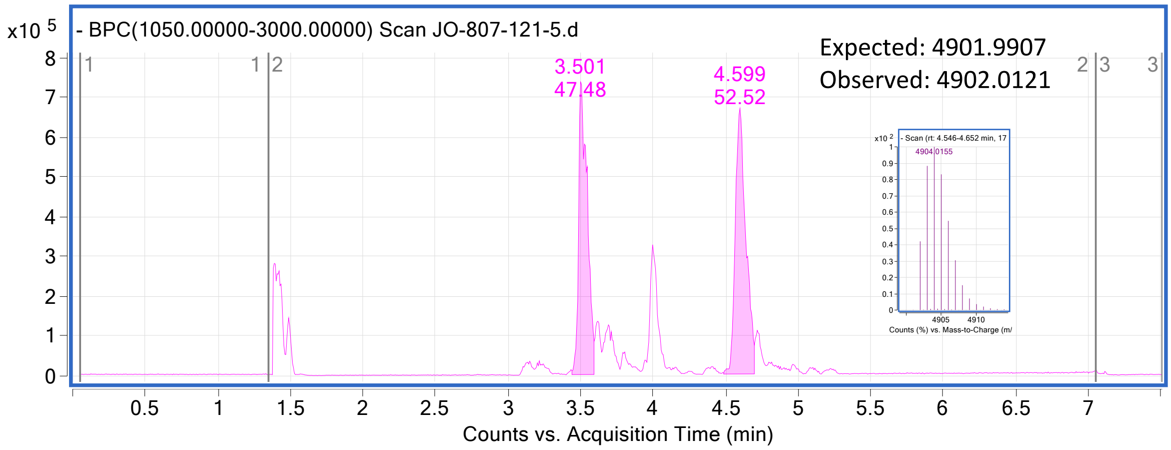

Figure S48: Mass spectrum of DNA-tagged product of Suzuki-Miyaura cross-coupling of HP4 and (4-(trifluoromethyl)phenyl)boronic acid using (lauryldimethylammonio)acetate as the surfactant analysed by DNA mass spectrometry gradient A.

Expected: 4901.9907
Observed: 4902.0109

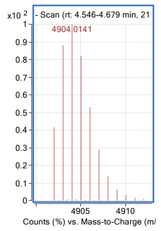

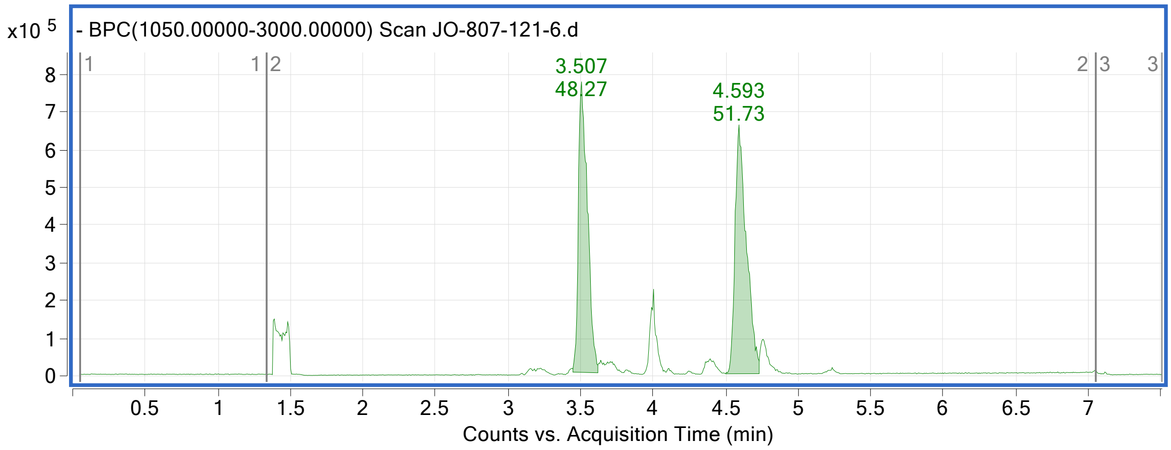

Figure S49: Mass spectrum of DNA-tagged product of Suzuki-Miyaura cross-coupling of HP4 and (4-(trifluoromethyl)phenyl)boronic acid using 4-dodecylbenzenesulfonic acid as the surfactant analysed by DNA mass spectrometry gradient A.

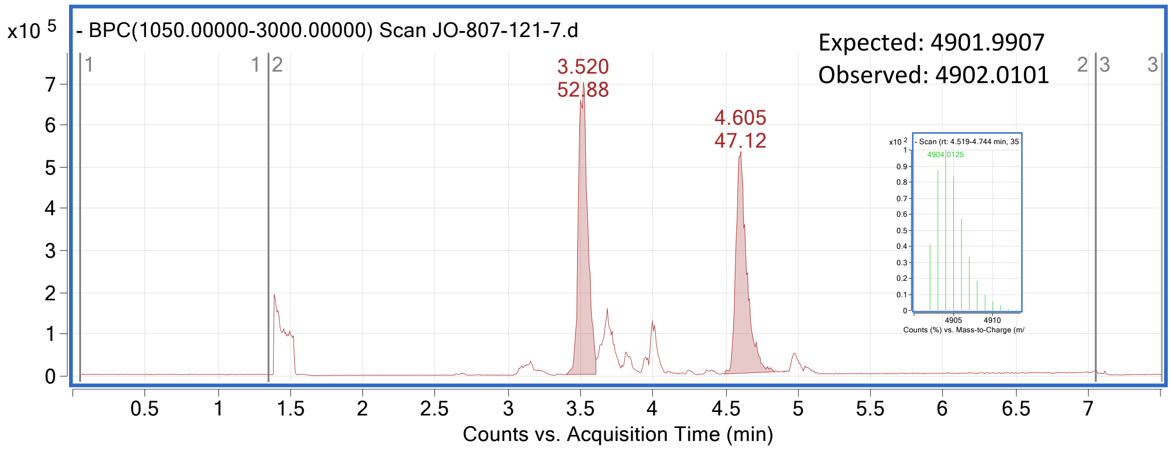

Figure S50: Mass spectrum of DNA-tagged product of Suzuki-Miyaura cross-coupling of HP4 and (4-(trifluoromethyl)phenyl)boronic acid using sulfobetaine-16 as the surfactant analysed by DNA mass spectrometry gradient A.

Expected: 4901.9907
Observed: 4902.0089

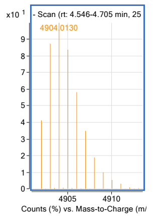

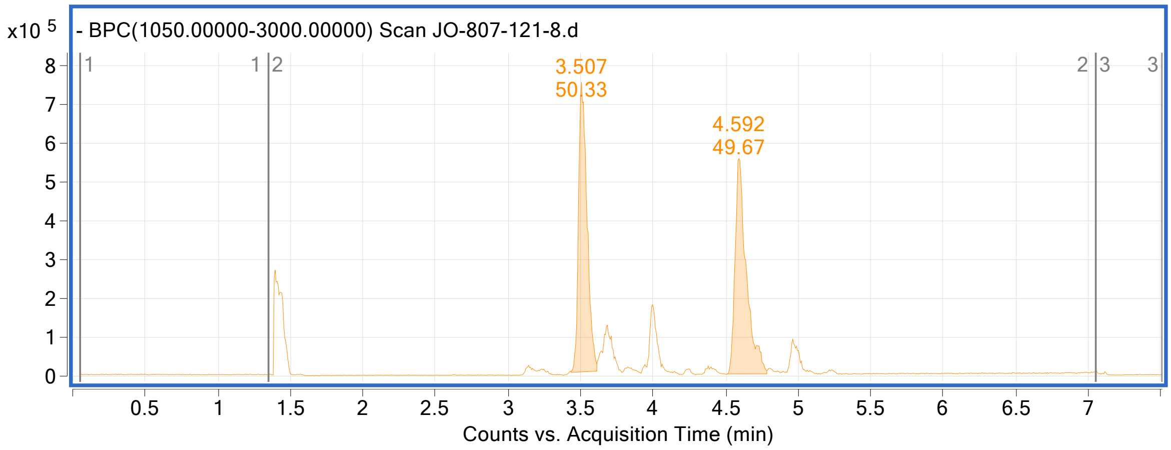

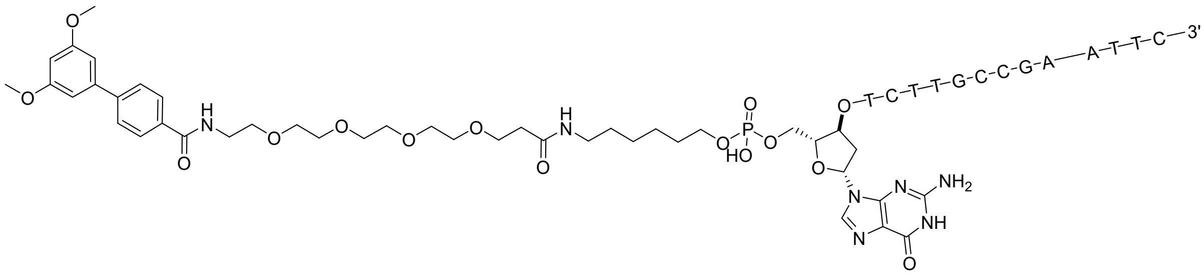

Figure S51: Mass spectrum of DNA-tagged product of Suzuki-Miyaura cross-coupling of HP4 and (3,5-dimethoxyphenyl)boronic acid using TPGS-750-M as the surfactant analysed by DNA mass spectrometry gradient A.

Expected: 4894.0244
Observed: 4894.0442

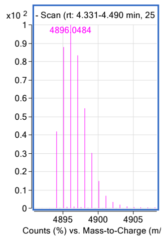

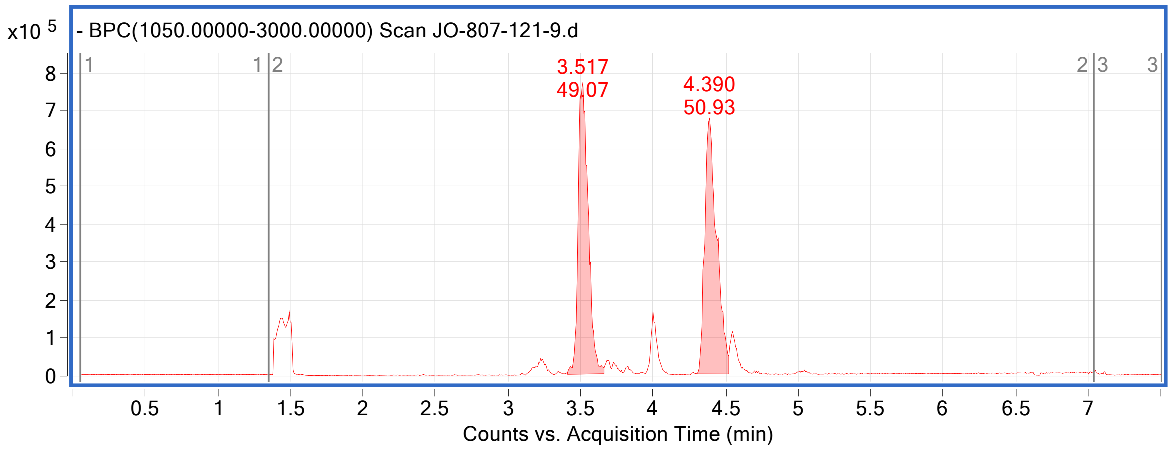

Figure S52: Mass spectrum of DNA-tagged product of Suzuki-Miyaura cross-coupling of HP4 and (3,5-dimethoxyphenyl)boronic acid using (lauryldimethylammonio)acetate as the surfactant analysed by DNA mass spectrometry gradient A.

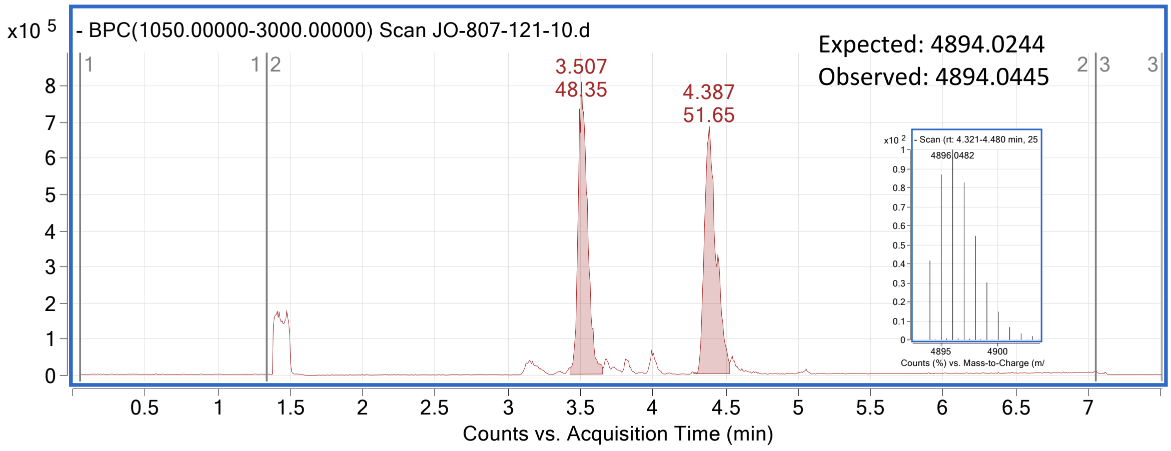

Figure S53: Mass spectrum of DNA-tagged product of Suzuki-Miyaura cross-coupling of HP4 and (3,5-dimethoxyphenyl)boronic acid using 4-dodecylbenzenesulfonic acid as the surfactant analysed by DNA mass spectrometry gradient A.

Expected: 4894.0244
Observed: 4894.0440

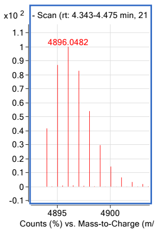

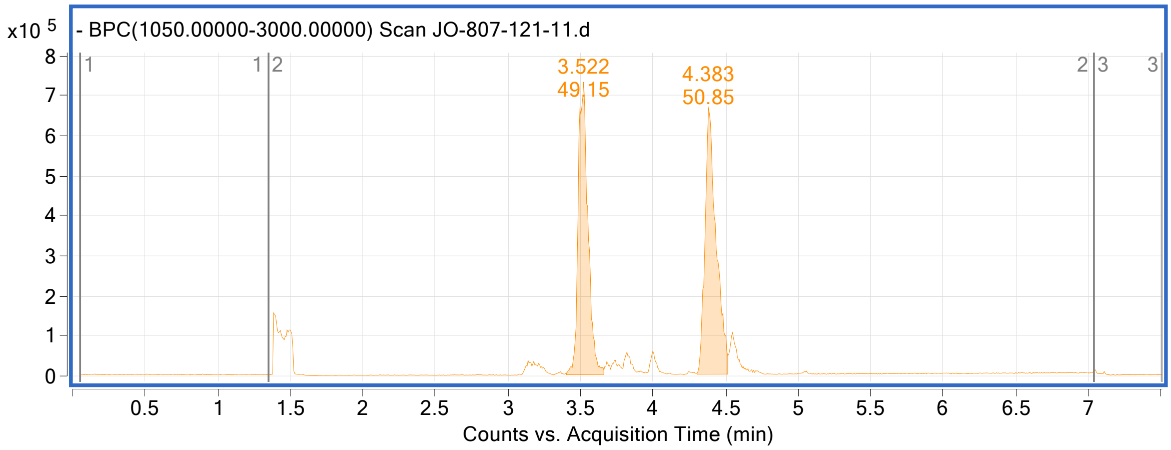

Figure S54: Mass spectrum of DNA-tagged product of Suzuki-Miyaura cross-coupling of HP4 and (3,5-dimethoxyphenyl)boronic acid using sulfobetaine-16 as the surfactant analysed by DNA mass spectrometry gradient A.

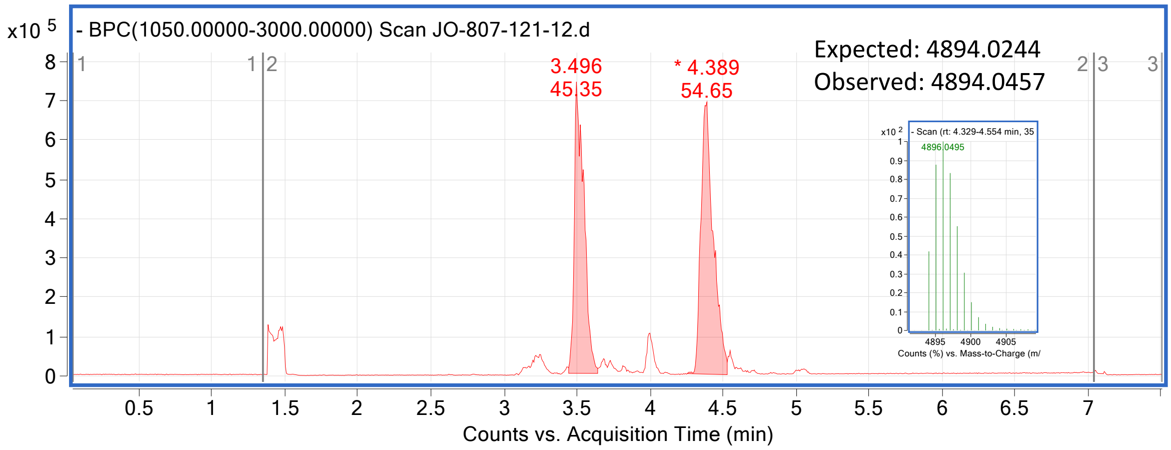

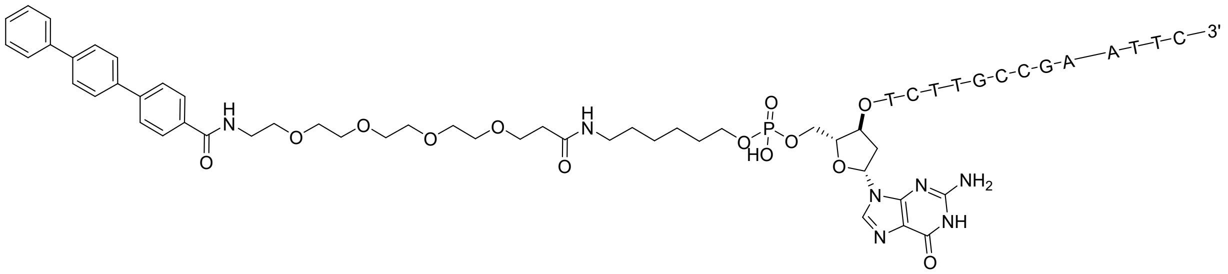

Figure S55: Mass spectrum of DNA-tagged product of Suzuki-Miyaura cross-coupling of HP4 and [1,1'-biphenyl]-3-ylboronic acid using TPGS-750-M as the surfactant analysed by DNA mass spectrometry gradient A.

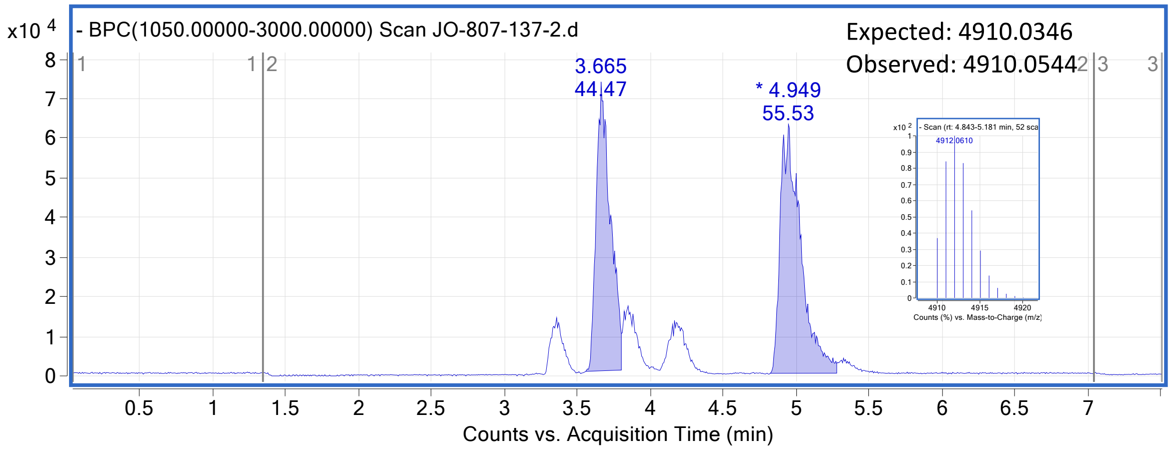

Figure S56: Mass spectrum of DNA-tagged product of Suzuki-Miyaura cross-coupling of HP4 and [1,1'-biphenyl]-3-ylboronic acid using (lauryldimethylammonio)acetate as the surfactant analysed by DNA mass spectrometry gradient A.

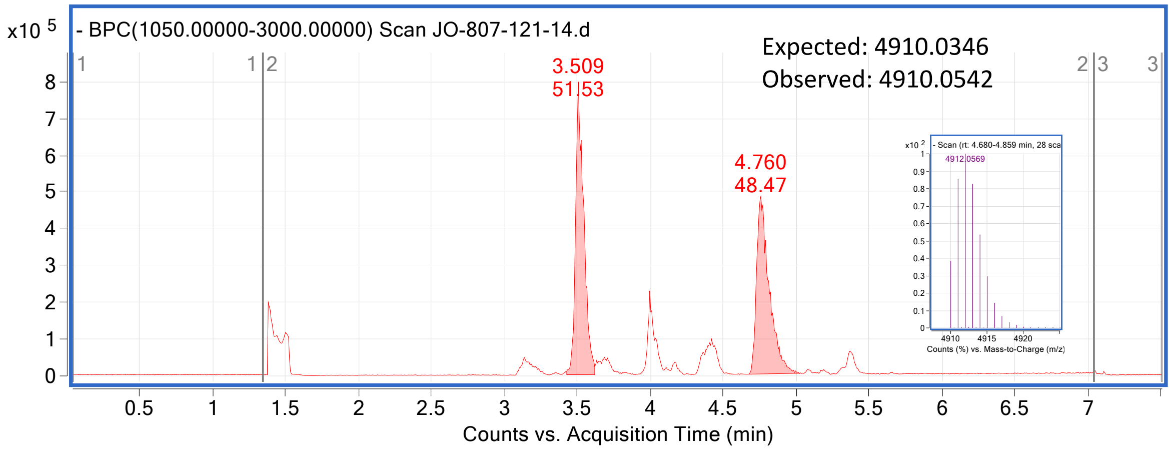

Figure S57: Mass spectrum of DNA-tagged product of Suzuki-Miyaura cross-coupling of HP4 and [1,1'-biphenyl]-3-ylboronic acid using 4-dodecylbenzenesulfonic acid as the surfactant analysed by DNA mass spectrometry gradient A.

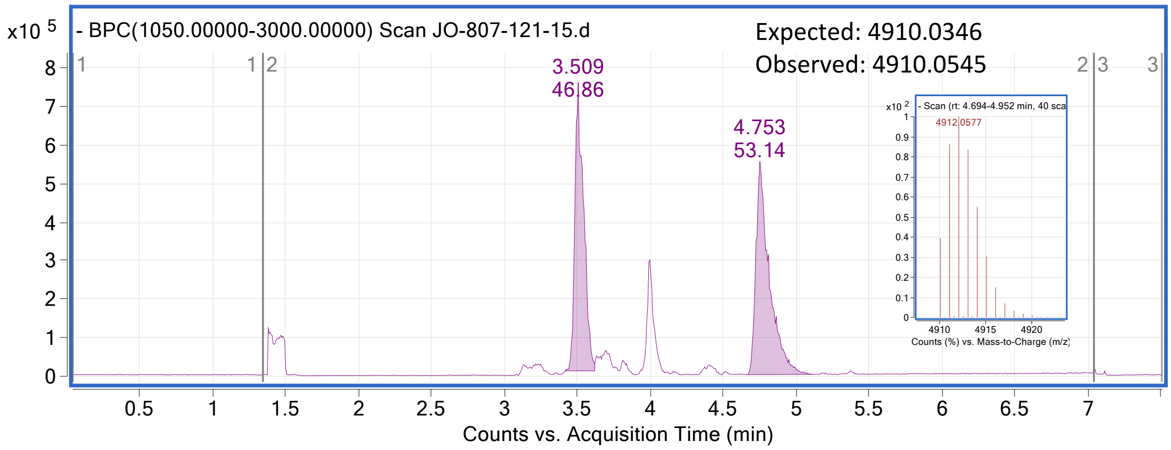

Figure S58: Mass spectrum of DNA-tagged product of Suzuki-Miyaura cross-coupling of HP4 and [1,1'-biphenyl]-3-ylboronic acid using sulfobetaine-16 as the surfactant analysed by DNA mass spectrometry gradient A.

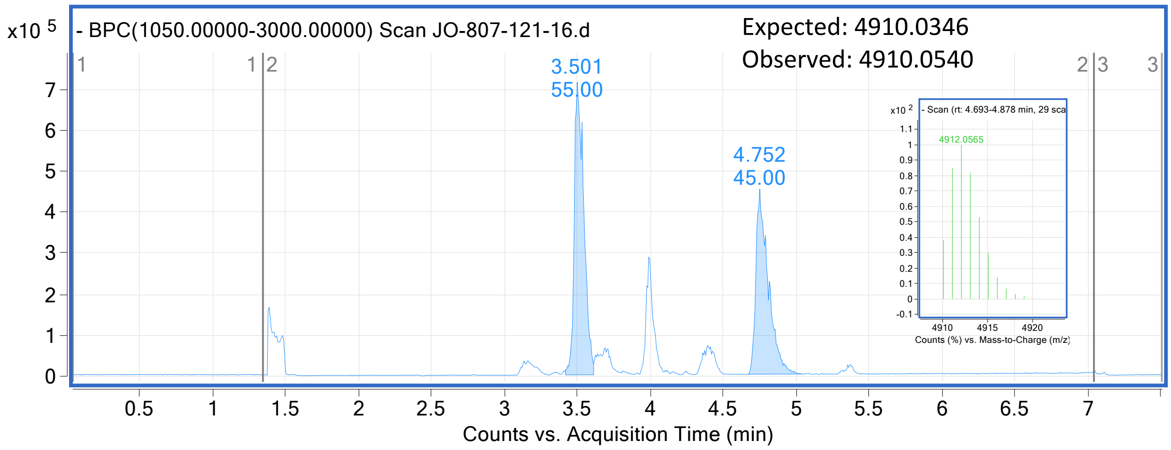

Table 4 Chromatograms

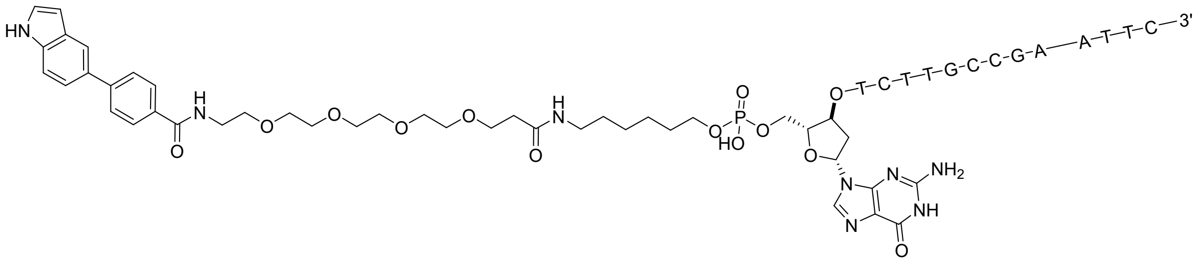

Figure S59: Mass spectrum of DNA-tagged product of Suzuki-Miyaura cross-coupling of HP4 and (1*H*-indol-5-yl)boronic acid using TPGS-750-M as the surfactant analysed by DNA mass spectrometry gradient A.

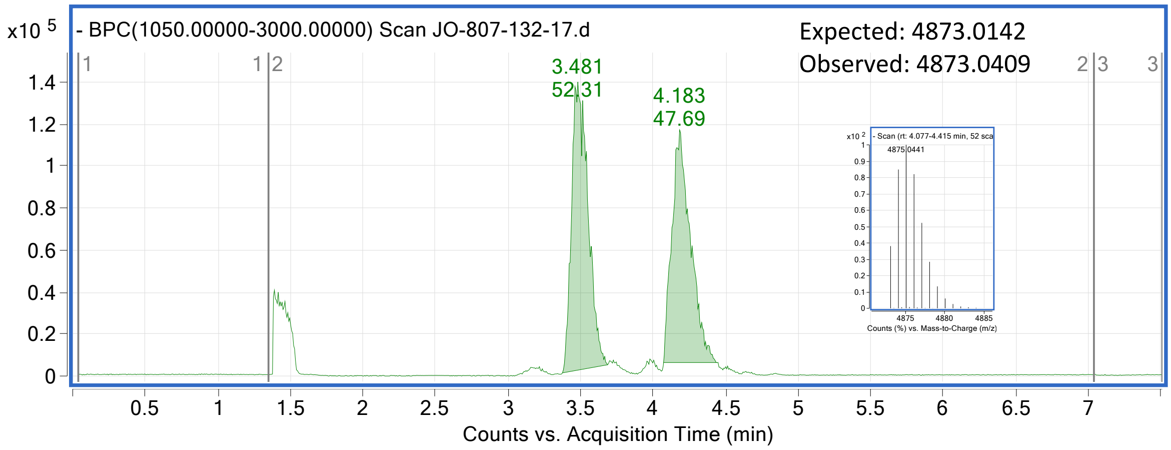

Figure S60: Mass spectrum of DNA-tagged product of Suzuki-Miyaura cross-coupling of HP4 and (1*H*-indol-5-yl)boronic acid using 4-dodecylbenzenesulfonic acid as the surfactant analysed by DNA mass spectrometry gradient A.

Expected: 4873.0142
Observed: 4873.0529

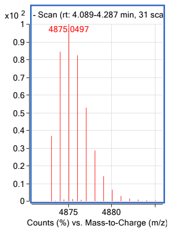

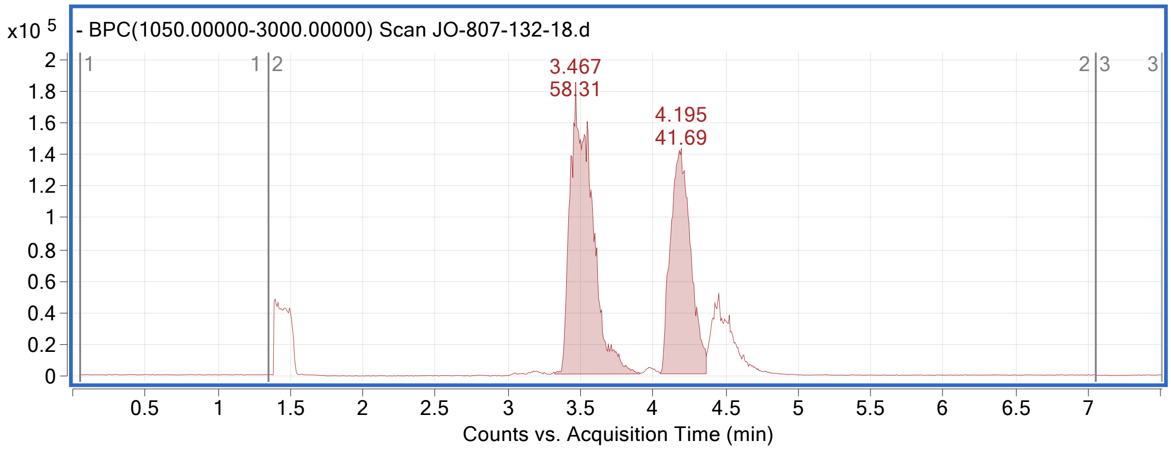

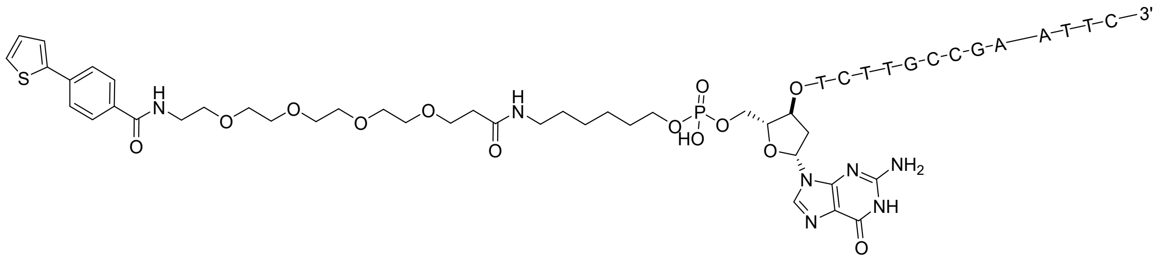

Figure S61: Mass spectrum of DNA-tagged product of Suzuki-Miyaura cross-coupling of HP4 and thiophen-2-ylboronic acid using TPGS-750-M as the surfactant analysed by DNA mass spectrometry gradient A.

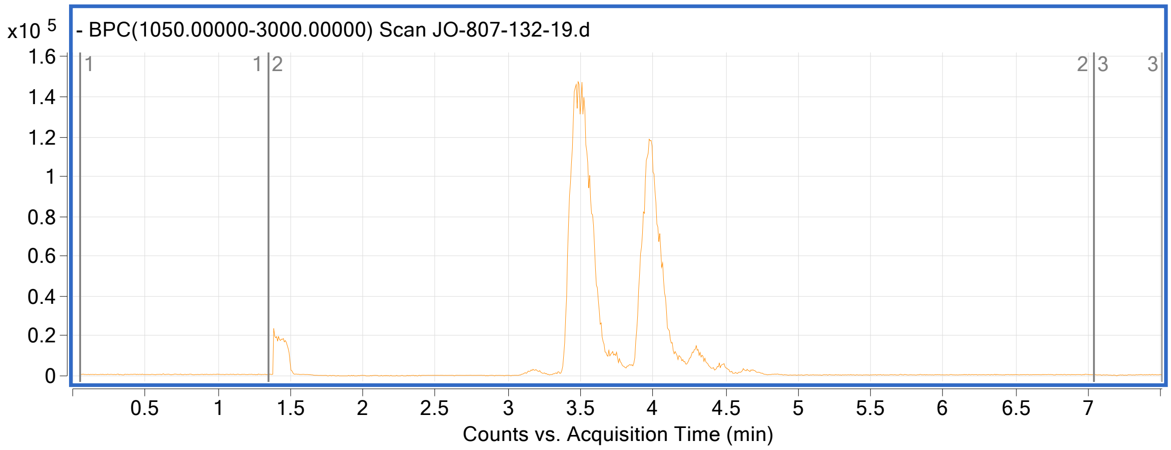

Figure S62: Mass spectrum of DNA-tagged product of Suzuki-Miyaura cross-coupling of HP4 and thiophen-2-ylboronic acid using 4-dodecylbenzenesulfonic acid as the surfactant analysed by DNA mass spectrometry gradient A.

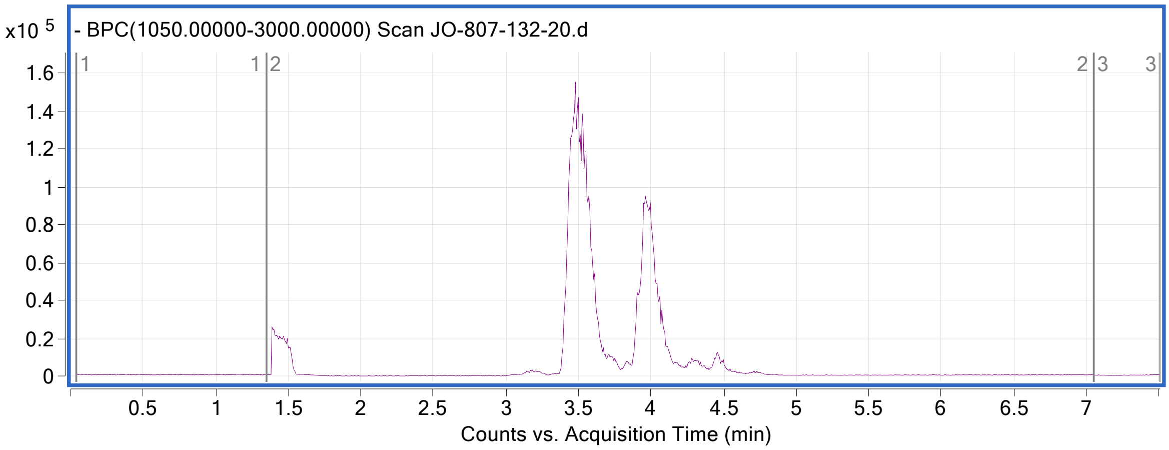

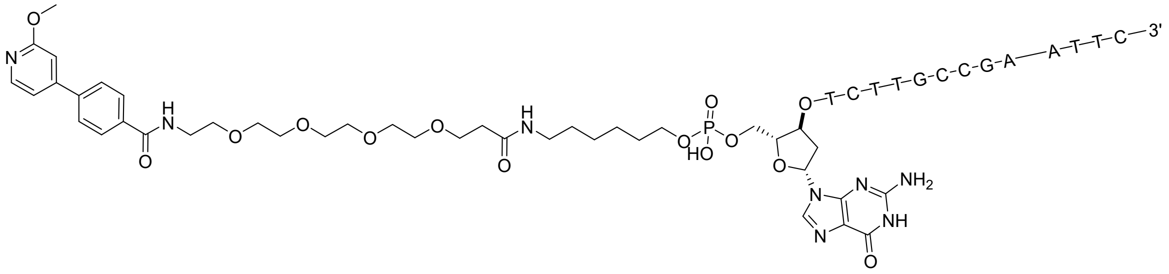

Figure S63: Mass spectrum of DNA-tagged product of Suzuki-Miyaura cross-coupling of HP4 and (2-methoxypyridin-4-yl)boronic acid using TPGS-750-M as the surfactant analysed by DNA mass spectrometry gradient A.

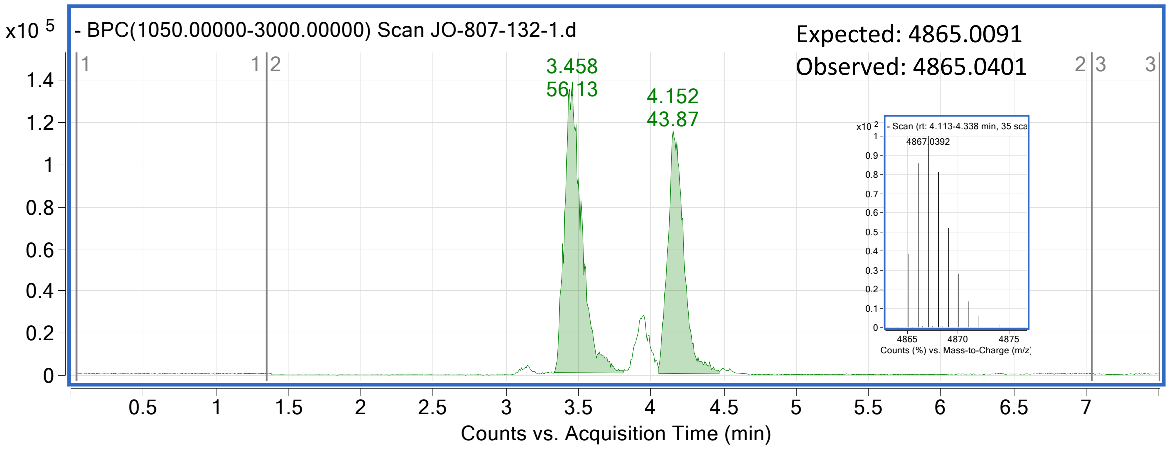

Figure S64: Mass spectrum of DNA-tagged product of Suzuki-Miyaura cross-coupling of HP4 and (2-methoxypyridin-4-yl)boronic acid using 4-dodecylbenzenesulfonic acid as the surfactant analysed by DNA mass spectrometry gradient A.

Expected: 4865.0091
Observed: 4865.0402

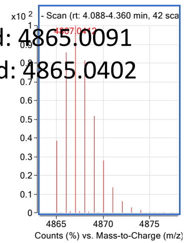

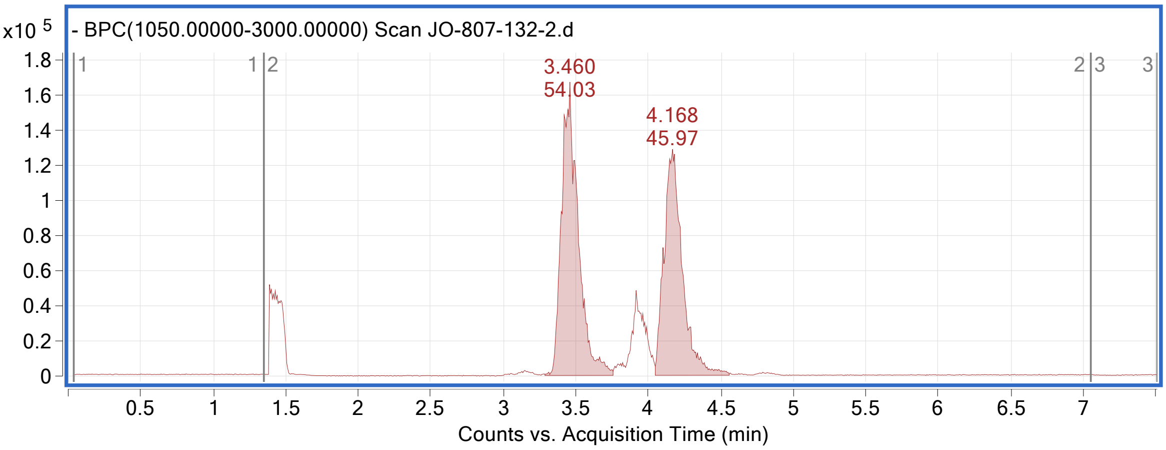

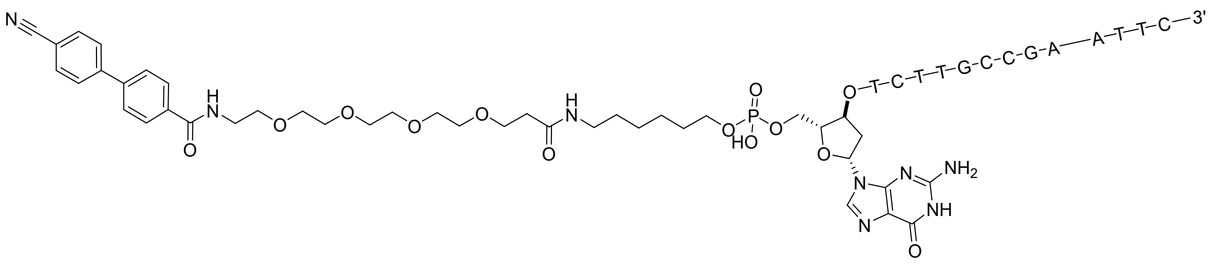

Figure S65: Mass spectrum of DNA-tagged product of Suzuki-Miyaura cross-coupling of HP4 and (4-cyanophenyl)boronic acid using TPGS-750-M as the surfactant analysed by DNA mass spectrometry gradient A.

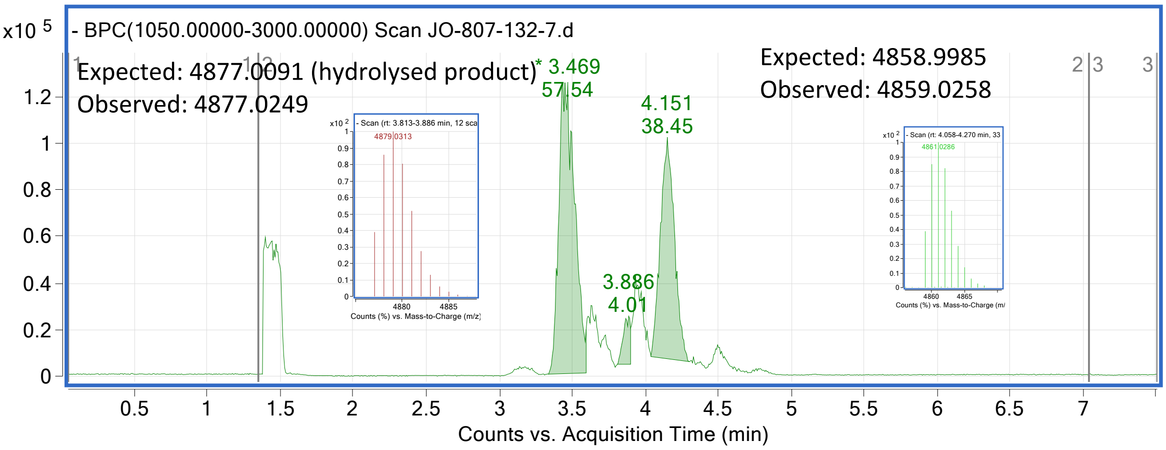

Figure S66: Mass spectrum

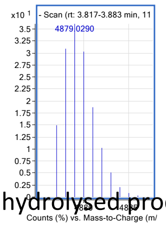

of DNA-tagged product of

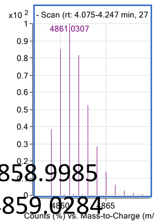

Suzuki-

Expected: 4877.0091 (hydrolysed product)

Expected: 4858.9985

Observed: 4859.0284

Miyaura cross-coupling of HP4 and (4-cyanophenyl)boronic acid using 4-dodecylbenzenesulfonic acid as the surfactant analysed by DNA mass spectrometry gradient A.

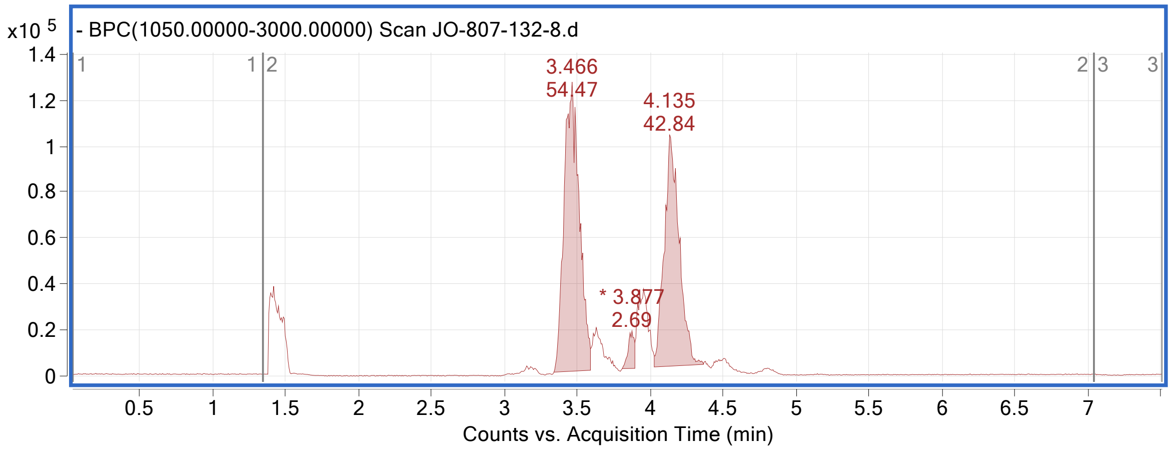

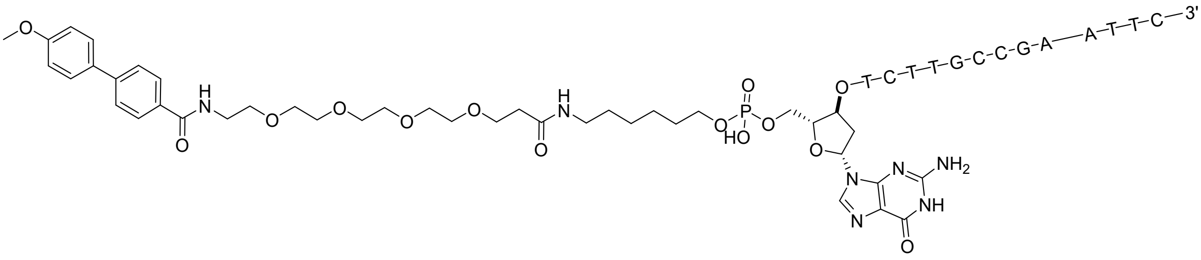

Figure S67: Mass spectrum of DNA-tagged product of Suzuki-Miyaura cross-coupling of HP4 and (4-methoxyphenyl)boronic acid using TPGS-750-M as the surfactant analysed by DNA mass spectrometry gradient A.

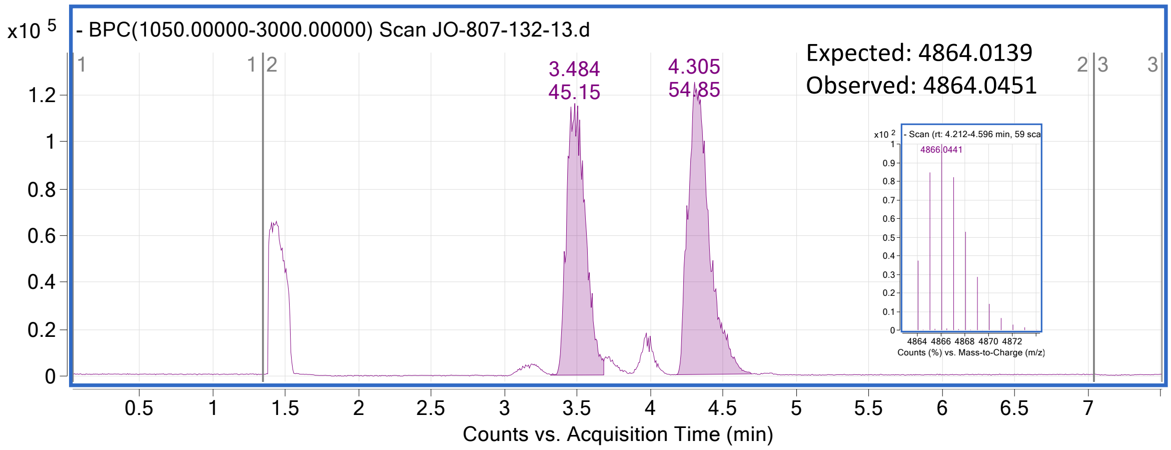

Figure S68: Mass spectrum of DNA-tagged product of Suzuki-Miyaura cross-coupling of HP4 and (4-methoxyphenyl)boronic acid using 4-dodecylbenzenesulfonic acid as the surfactant analysed by DNA mass spectrometry gradient A.

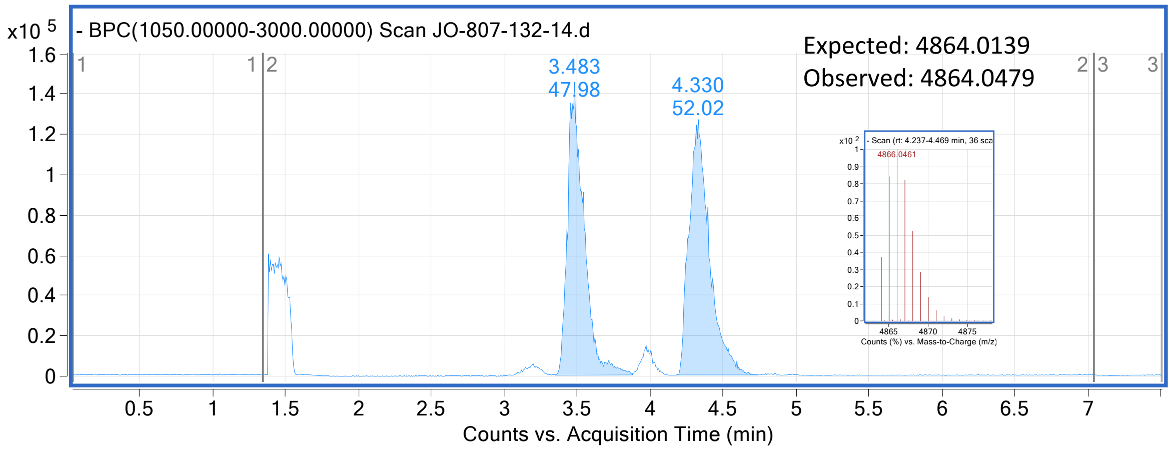

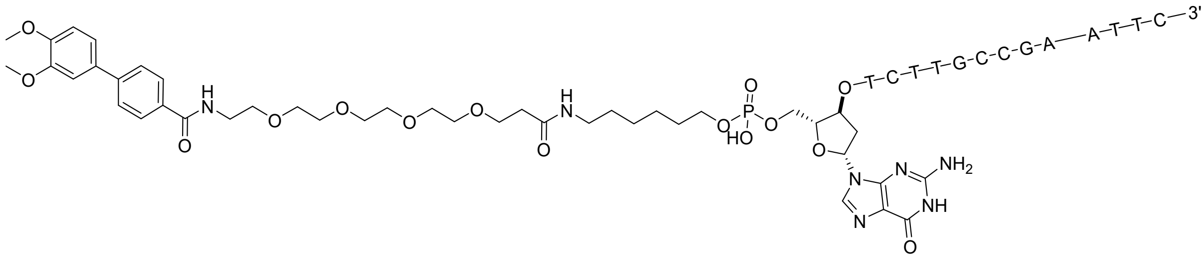

Figure S69: Mass spectrum of DNA-tagged product of Suzuki-Miyaura cross-coupling of HP4 and (3,4-dimethoxyphenyl)boronic acid using TPGS-750-M as the surfactant analysed by DNA mass spectrometry gradient A.

Expected: 4894.0244
Observed: 4894.0592

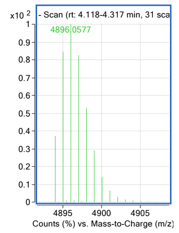

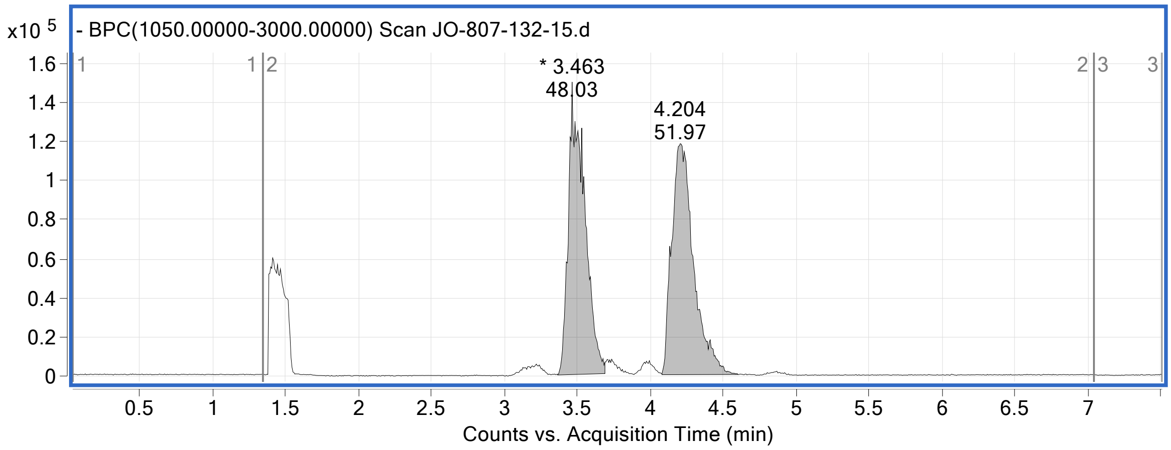

Figure S70: Mass spectrum of DNA-tagged product of Suzuki-Miyaura cross-coupling of HP4 and (3,4-dimethoxyphenyl)boronic acid using 4-dodecylbenzenesulfonic acid as the surfactant analysed by DNA mass spectrometry gradient A.

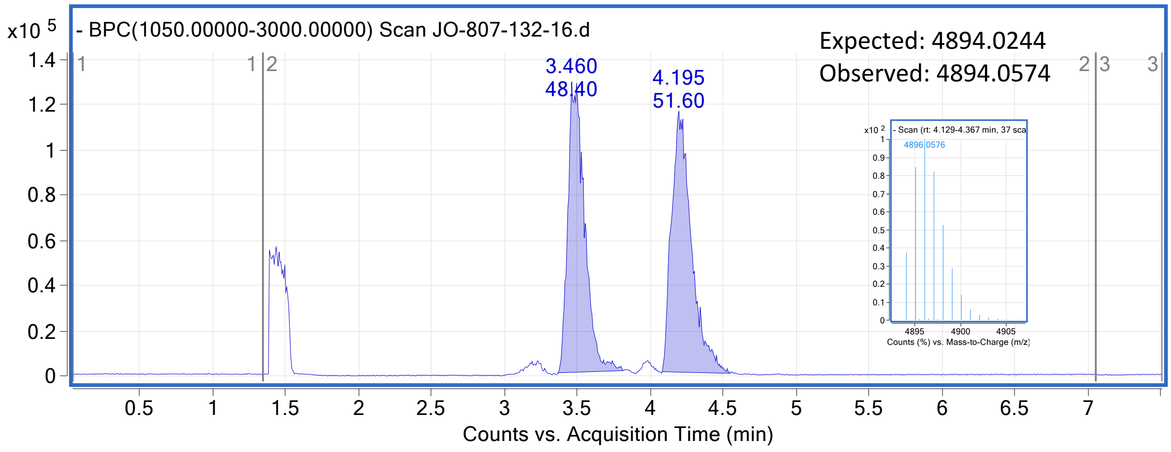

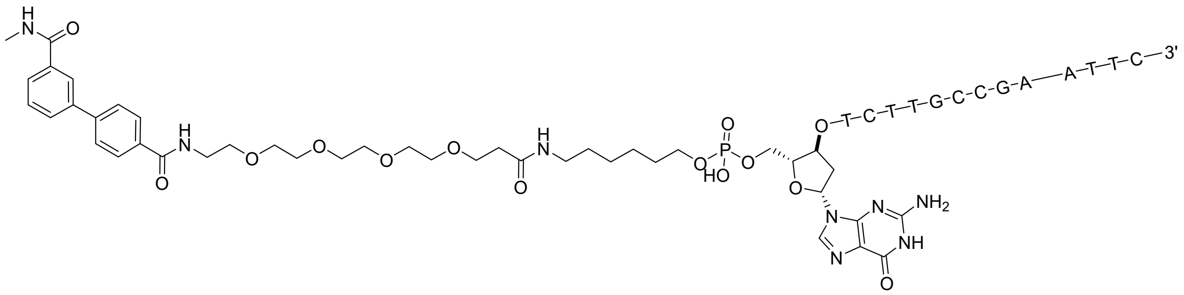

Figure S71: Mass spectrum of DNA-tagged product of Suzuki-Miyaura cross-coupling of HP4 and (3-(methylcarbamoyl)phenyl)boronic acid using TPGS-750-M as the surfactant analysed by DNA mass spectrometry gradient A.

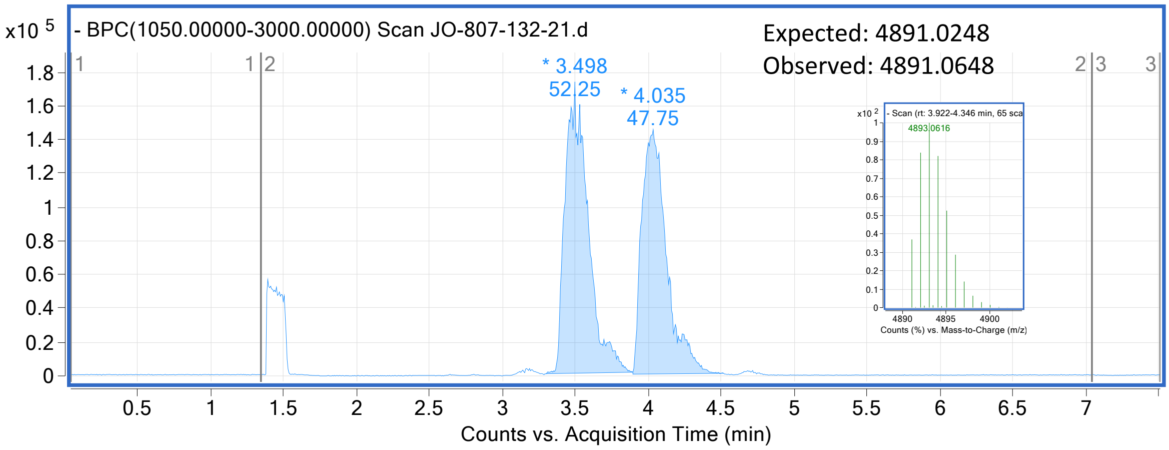

Figure S72: Mass spectrum of DNA-tagged product of Suzuki-Miyaura cross-coupling of HP4 and (3-(methylcarbamoyl)phenyl)boronic acid using 4-dodecylbenzenesulfonic acid as the surfactant analysed by DNA mass spectrometry gradient A.

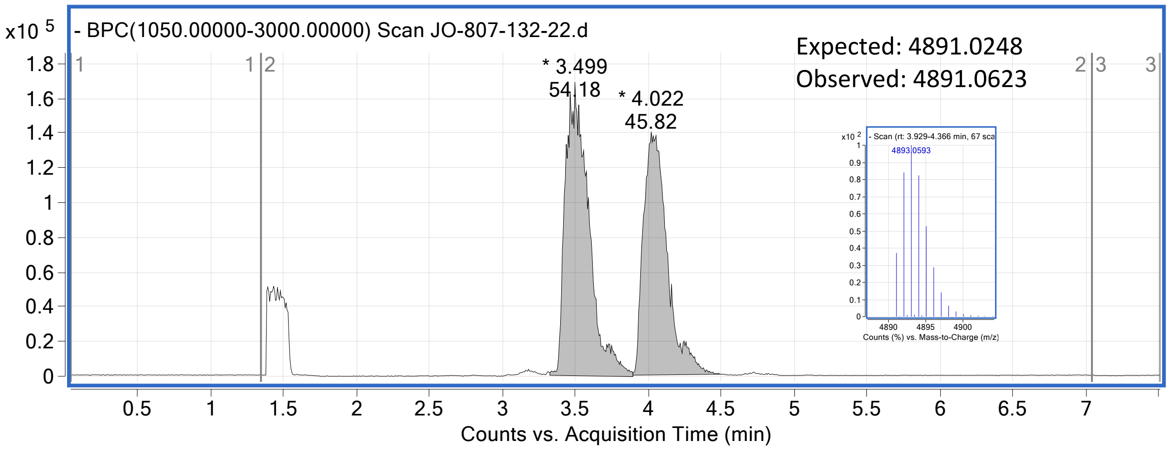

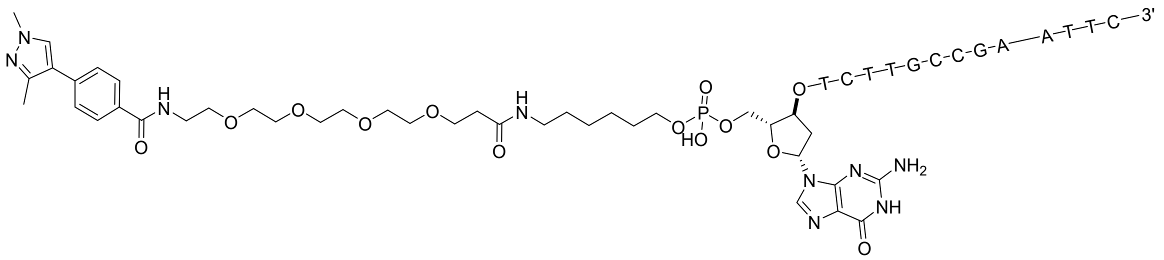

Figure S73: Mass spectrum of DNA-tagged product of Suzuki-Miyaura cross-coupling of HP4 and 1,3-dimethyl-4-(4,4,5,5-tetramethyl-1,3,2-dioxaborolan-2-yl)-1*H*-pyrazole using TPGS-750-M as the surfactant analysed by DNA mass spectrometry gradient A.

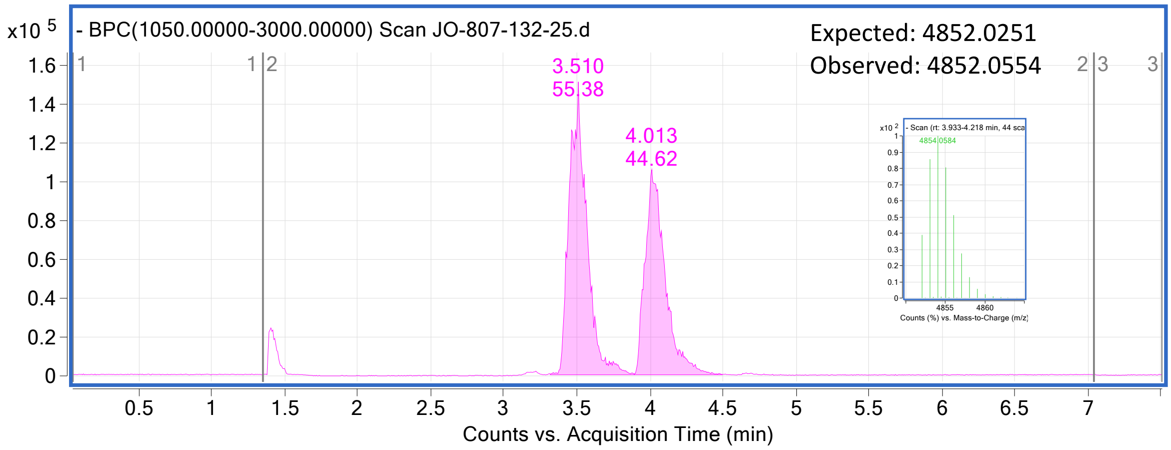

Figure S74: Mass spectrum of DNA-tagged product of Suzuki-Miyaura cross-coupling of HP4 and 1,3-dimethyl-4-(4,4,5,5-tetramethyl-1,3,2-dioxaborolan-2-yl)-1*H*-pyrazole using 4-dodecylbenzenesulfonic acid as the surfactant analysed by DNA mass spectrometry gradient A.

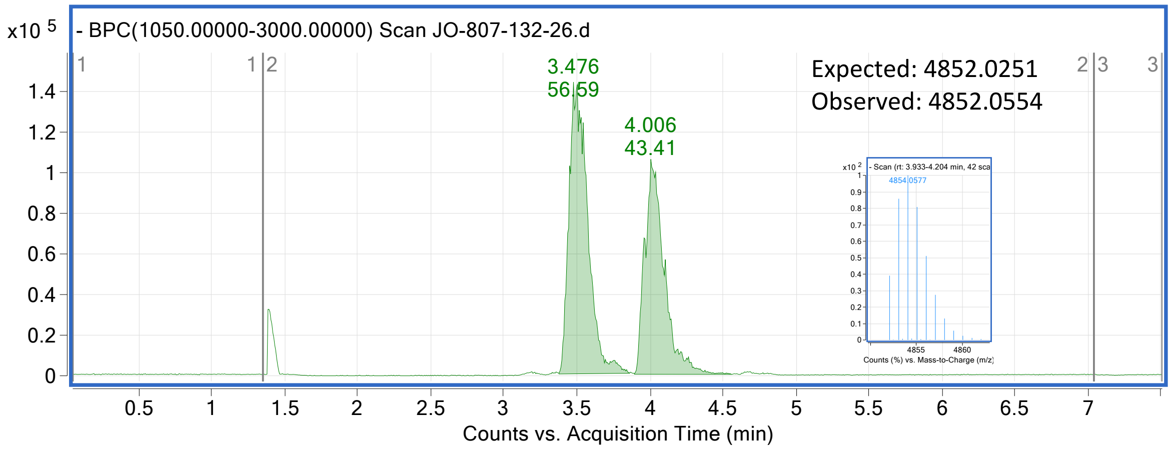

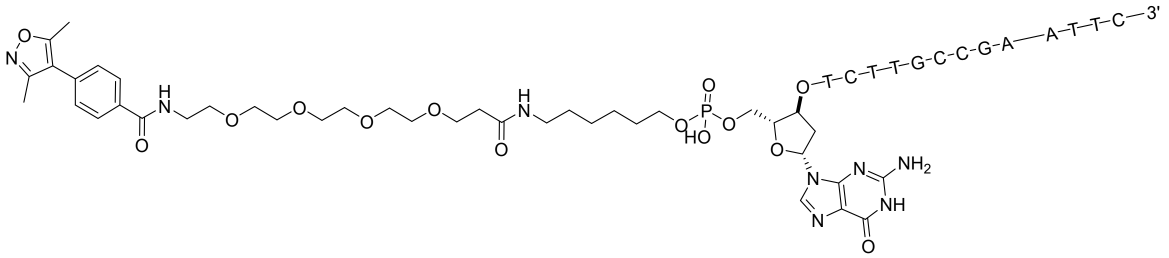

Figure S75: Mass spectrum of DNA-tagged product of Suzuki-Miyaura cross-coupling of HP4 and 3,5-dimethyl-4-(4,4,5,5-tetramethyl-1,3,2-dioxaborolan-2-yl)isoxazole using TPGS-750-M as the surfactant analysed by DNA mass spectrometry gradient A.

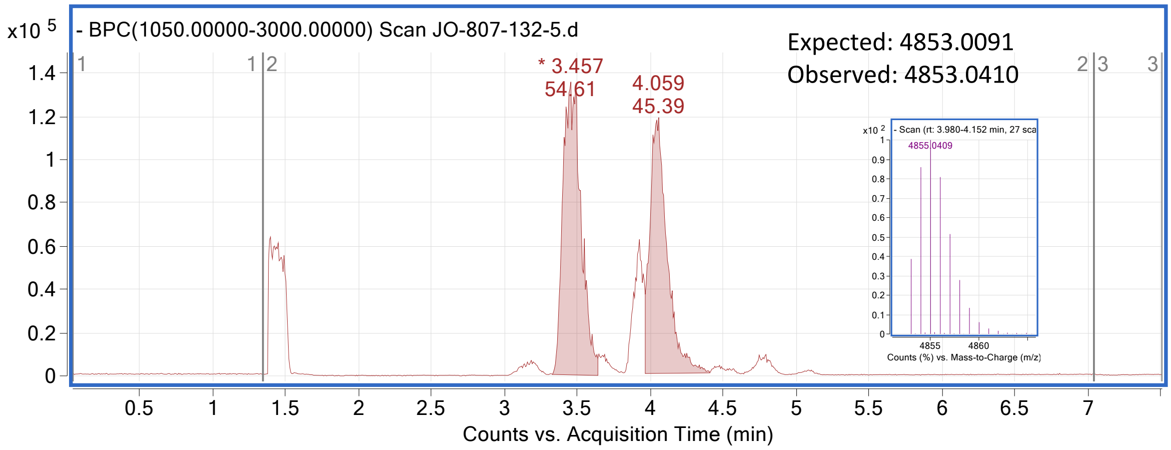

Figure S76: Mass spectrum of DNA-tagged product of Suzuki-Miyaura cross-coupling of HP4 and 3,5-dimethyl-4-(4,4,5,5-tetramethyl-1,3,2-dioxaborolan-2-yl)isoxazole using 4-dodecylbenzenesulfonic acid as the surfactant analysed by DNA mass spectrometry gradient A.

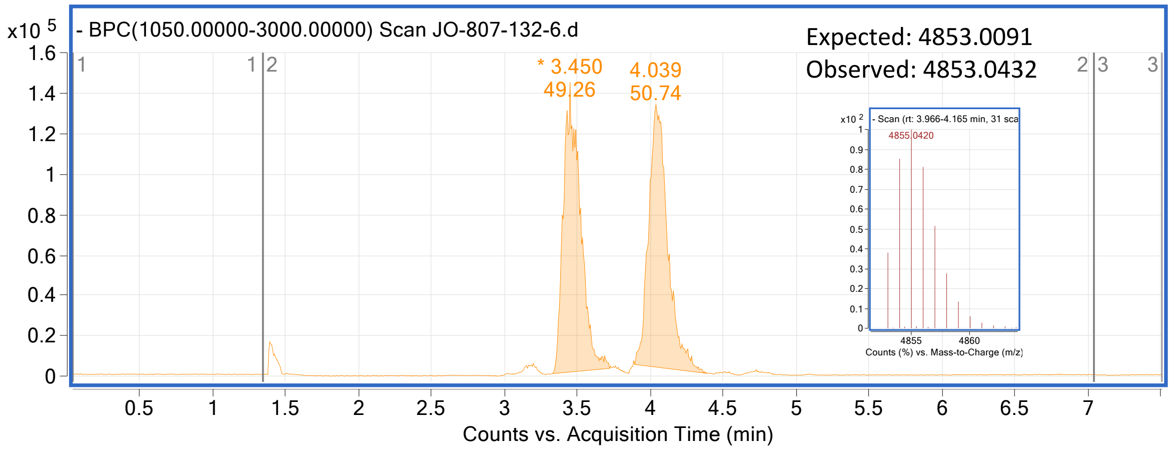

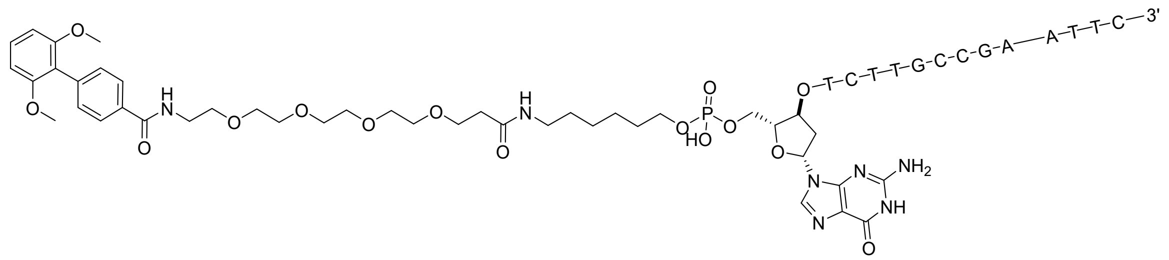

Figure S77: Mass spectrum of DNA-tagged product of Suzuki-Miyaura cross-coupling of HP4 and (2,6-dimethoxyphenyl)boronic acid using TPGS-750-M as the surfactant analysed by DNA mass spectrometry gradient A.

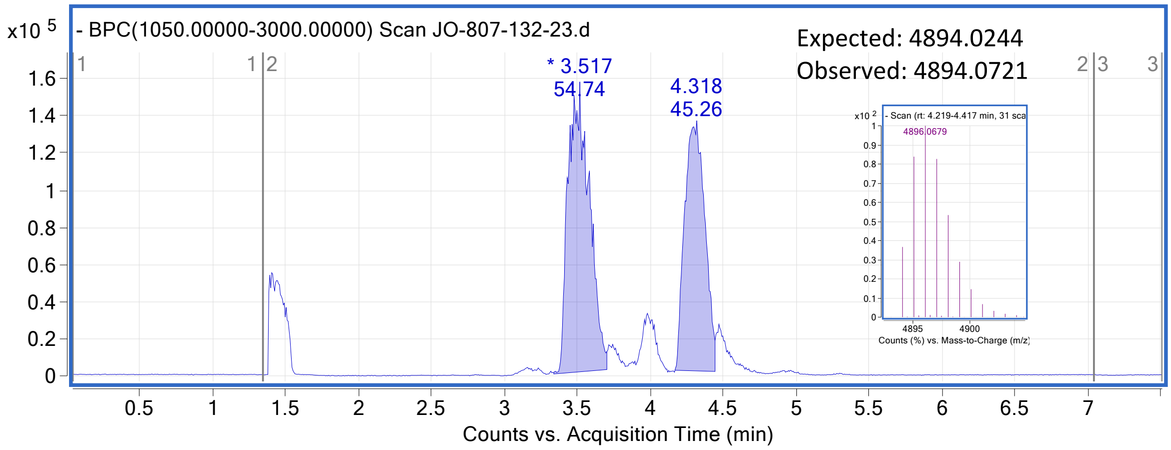

Figure S78: Mass spectrum of DNA-tagged product of Suzuki-Miyaura cross-coupling of HP4 and (2,6-dimethoxyphenyl)boronic acid using 4-dodecylbenzenesulfonic acid as the surfactant analysed by DNA mass spectrometry gradient A.

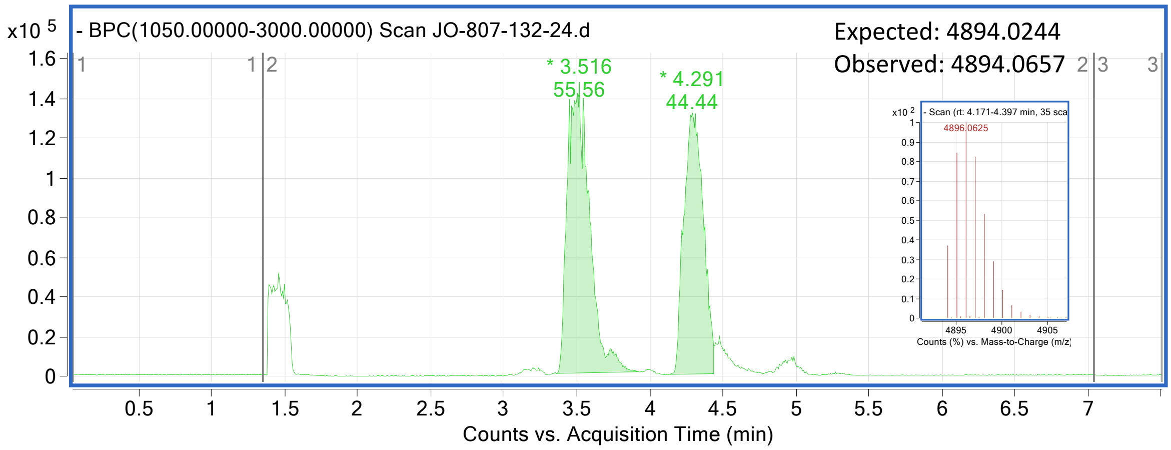

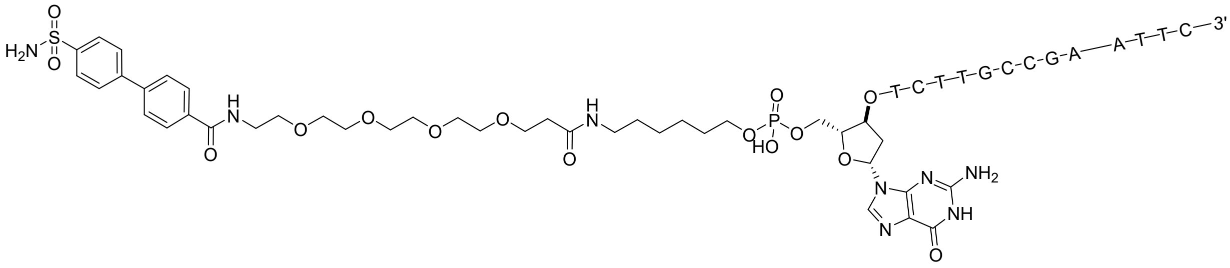

Figure S79: Mass spectrum of DNA-tagged product of Suzuki-Miyaura cross-coupling of HP4 and (4-sulfamoylphenyl)boronic acid using TPGS-750-M as the surfactant analysed by DNA mass spectrometry gradient A.

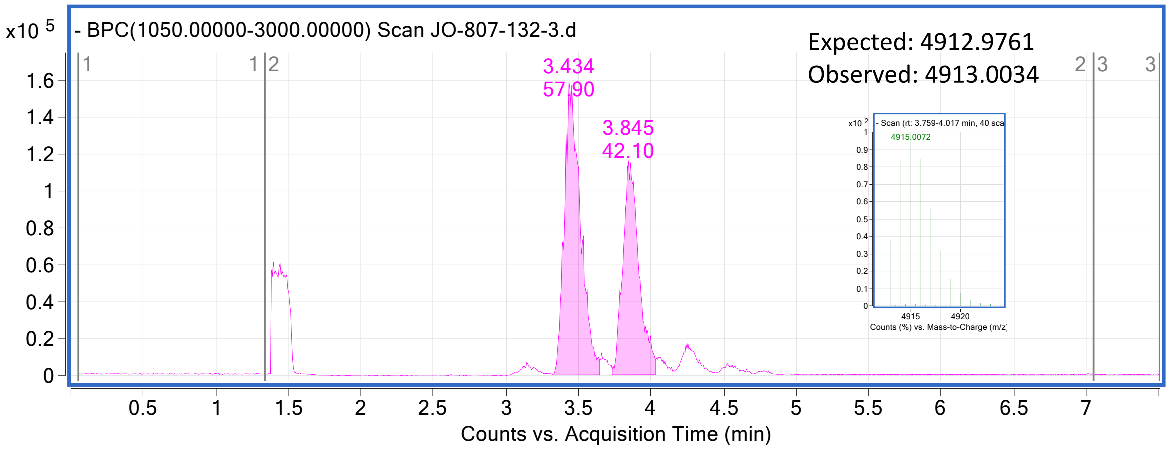

Figure S80: Mass spectrum of DNA-tagged product of Suzuki-Miyaura cross-coupling of HP4 and (4-sulfamoylphenyl)boronic acid using 4-dodecylbenzenesulfonic acid as the surfactant analysed by DNA mass spectrometry gradient A.

Expected: 4912.9761
Observed: 4913.0056

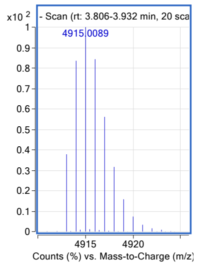

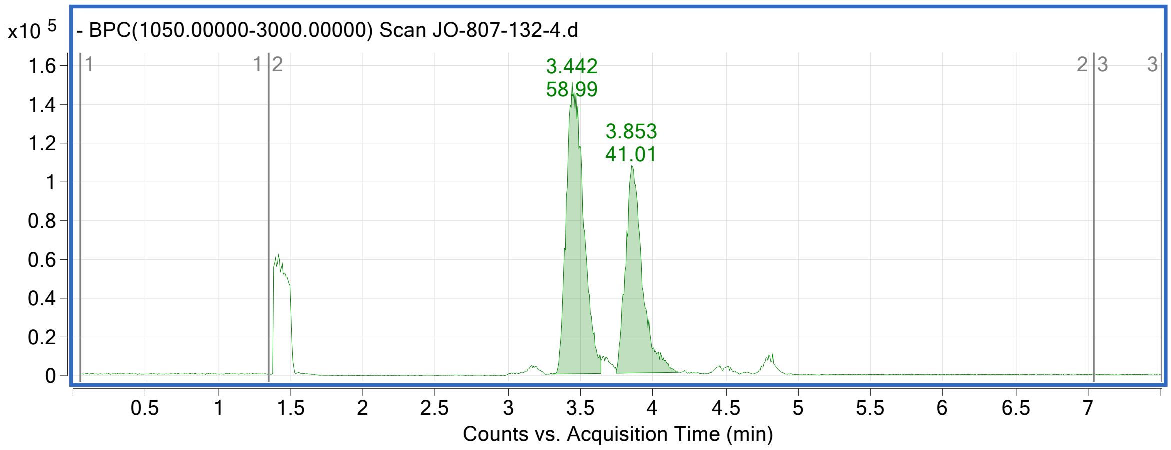

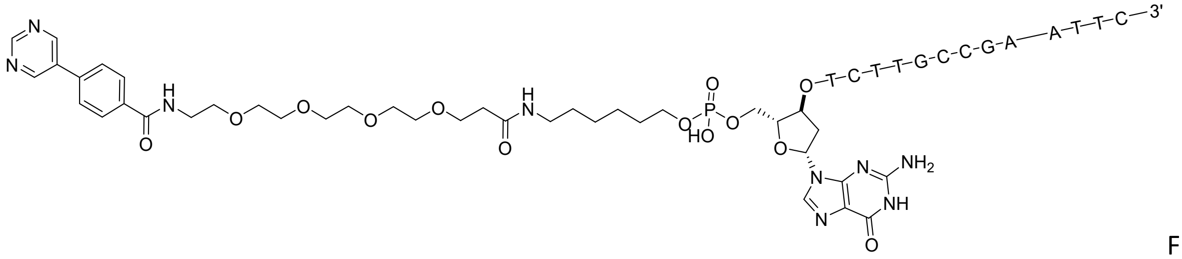

Figure S81: Mass spectrum of DNA-tagged product of Suzuki-Miyaura cross-coupling of HP4 and pyrimidin-5-ylboronic acid using TPGS-750-M as the surfactant analysed by DNA mass spectrometry gradient A.

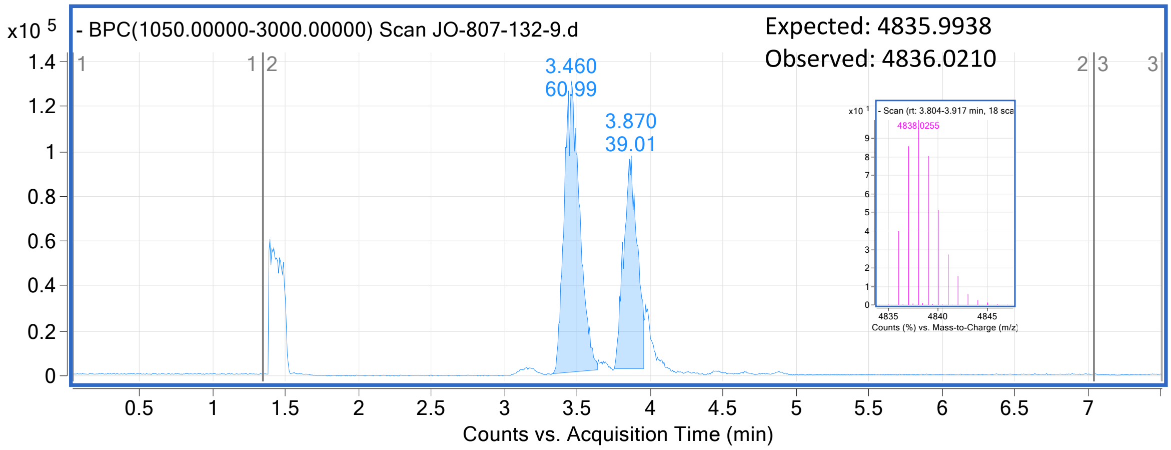

Figure S82: Mass spectrum of DNA-tagged product of Suzuki-Miyaura cross-coupling of HP4 and pyrimidin-5-ylboronic acid using 4-dodecylbenzenesulfonic acid as the

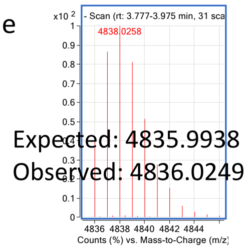

surfactant analysed by DNA mass spectrometry gradient A.

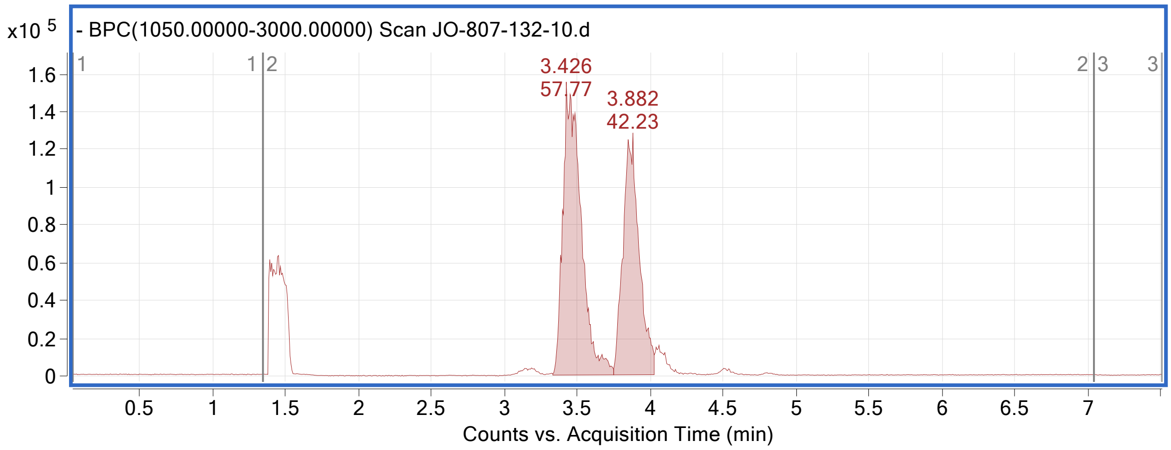

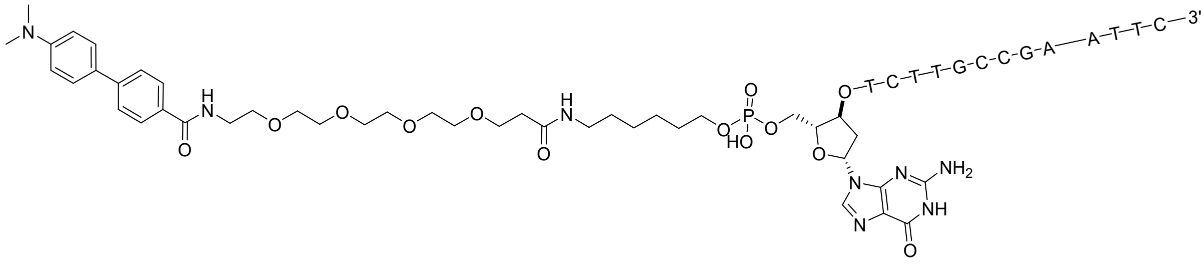

Figure S83: Mass spectrum of DNA-tagged product of Suzuki-Miyaura cross-coupling of HP4 and (4-(dimethylamino)phenyl)boronic acid using TPGS-750-M as the surfactant analysed by DNA mass spectrometry gradient A.

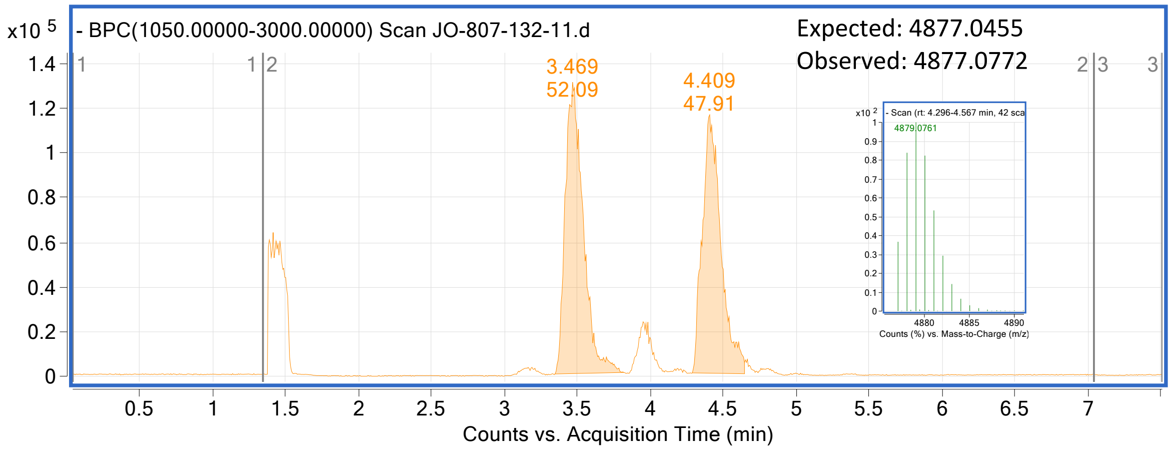

Figure S84: Mass spectrum of DNA-tagged product of Suzuki-Miyaura cross-coupling of HP4 and (4-(dimethylamino)phenyl)boronic acid using 4-dodecylbenzenesulfonic acid as the surfactant analysed by DNA mass spectrometry gradient A.

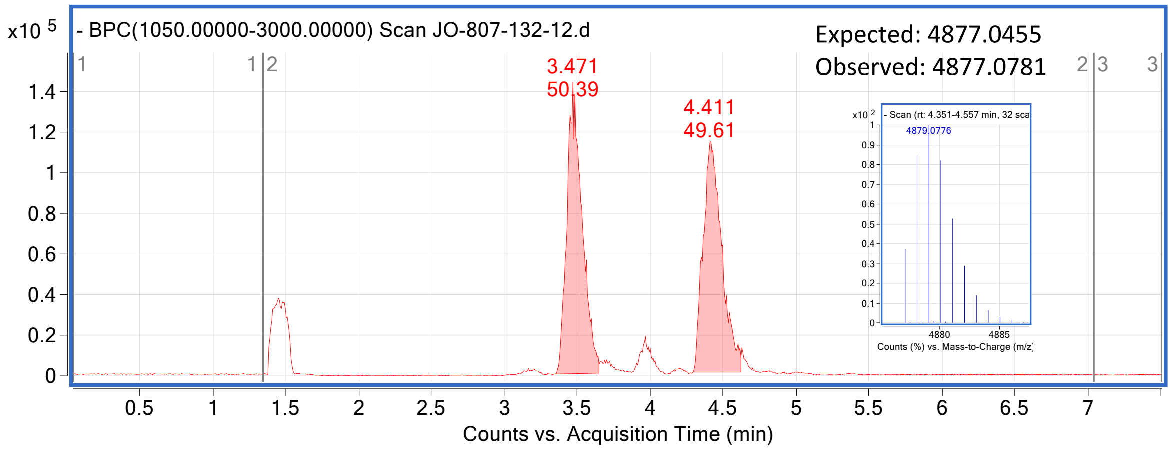

On-DNA Reductive Amination Reaction of DNA-Conjugated Amine HP2

Table 5 Chromatograms

Compound 4 was synthesised according to general reductive amination procedure.

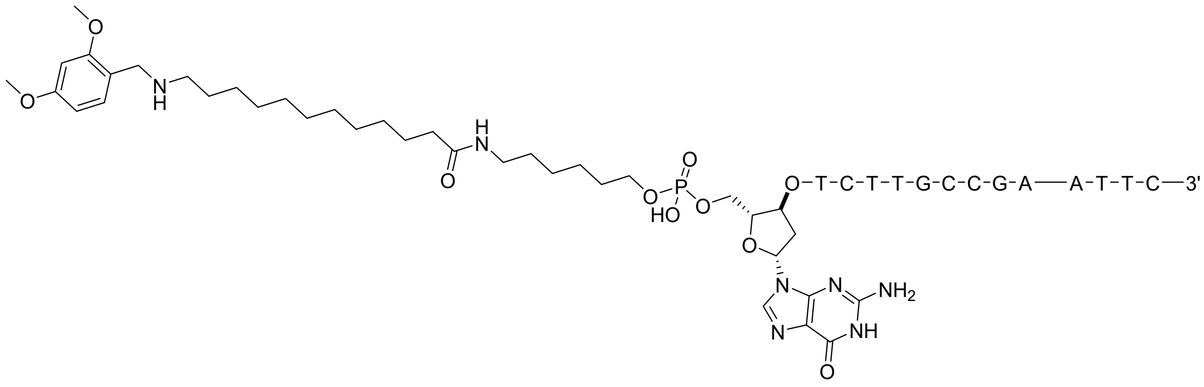

Figure S85: Mass spectrum of product 4 synthesised from HP2 and aldehyde 3 with TPGS-750-M as the surfactant analysed by DNA mass spectrometry gradient B.

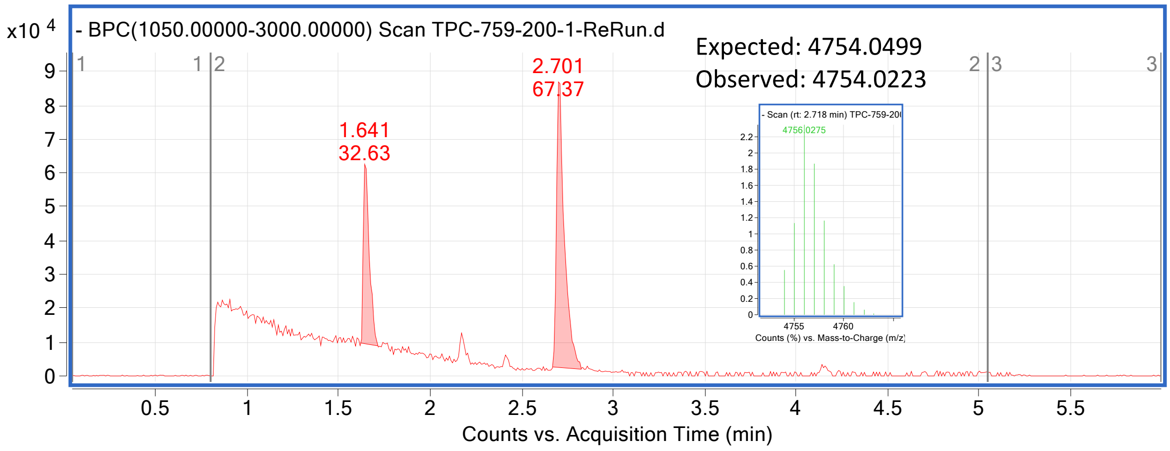

Figure S86: Mass spectrum of product 4 synthesised from HP2 and aldehyde 3 with PEG_5C_{12} as the surfactant analysed by DNA mass spectrometry gradient B.

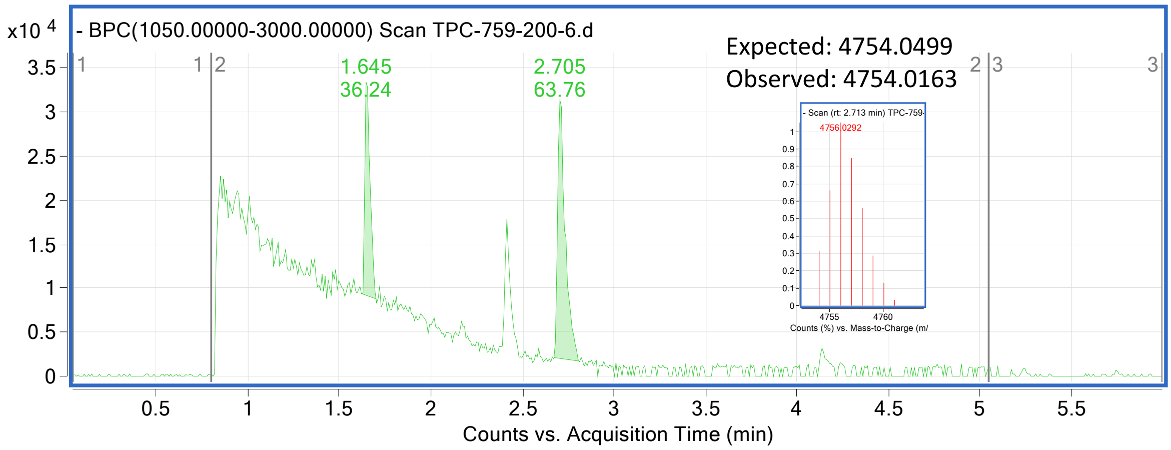

Figure S87: Mass spectrum of product 4 synthesised from HP2 and aldehyde 3 with Tween 65 as the surfactant analysed by DNA mass spectrometry gradient B.

Expected: 4754.0499
Observed: 4754.0180

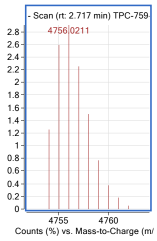

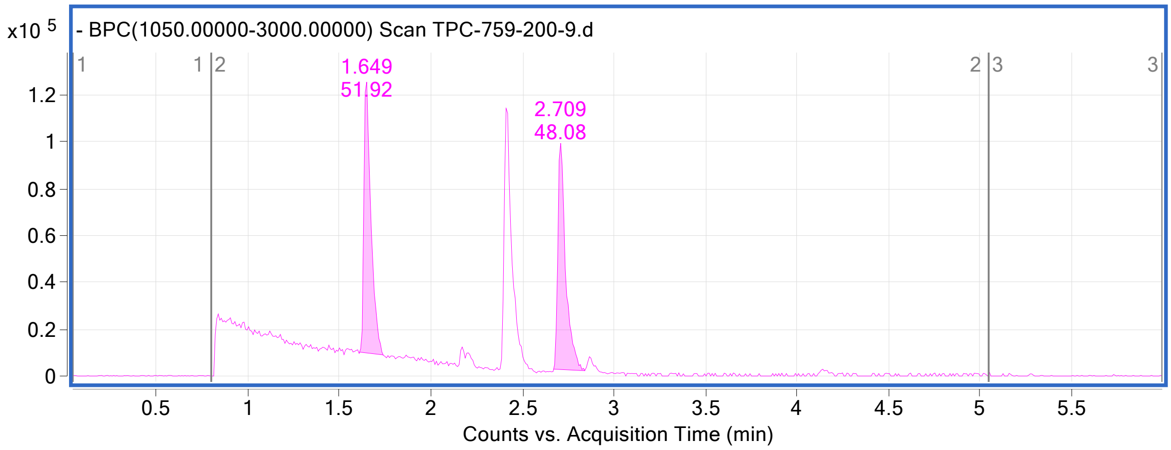

Figure S88: Mass spectrum of product 4 synthesised from HP2 and aldehyde 3 with Brij 700 as the surfactant analysed by DNA mass spectrometry gradient B.

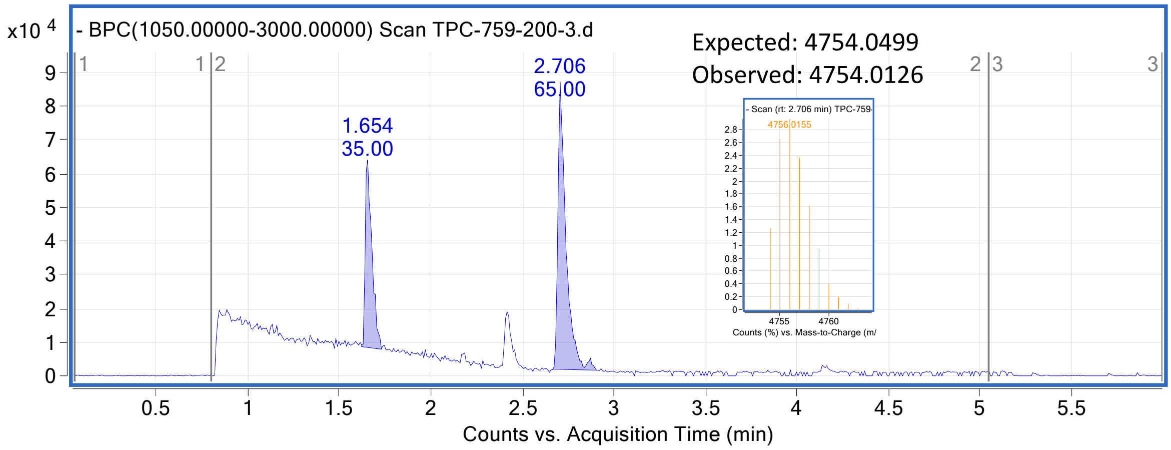

Figure S89: Mass spectrum of product 4 synthesised from HP2 and aldehyde 3 with Brij S20 as the surfactant analysed by DNA mass spectrometry gradient B.

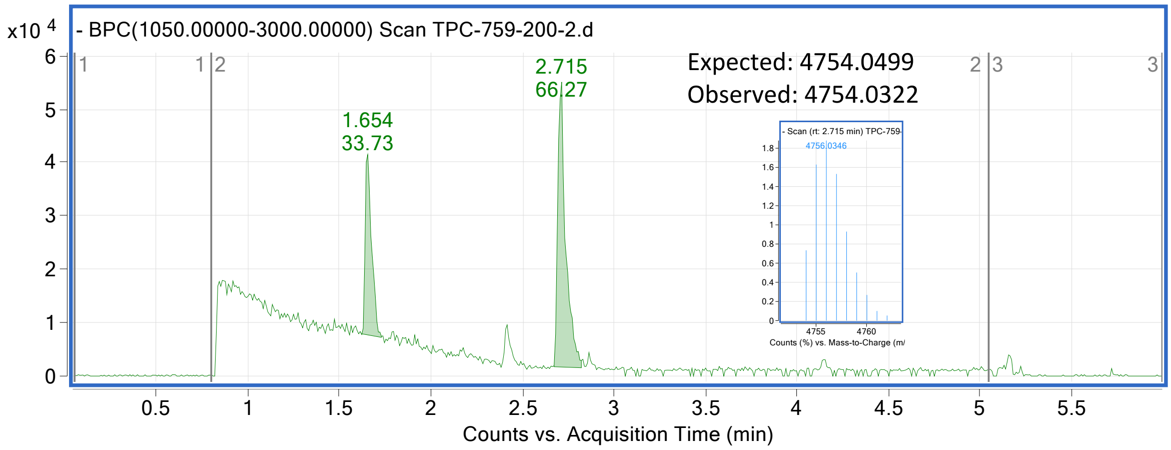

Figure S90: Mass spectrum of product 4 synthesised from HP2 and aldehyde 3 with Triton-X-405 as the surfactant analysed by DNA mass spectrometry gradient B.

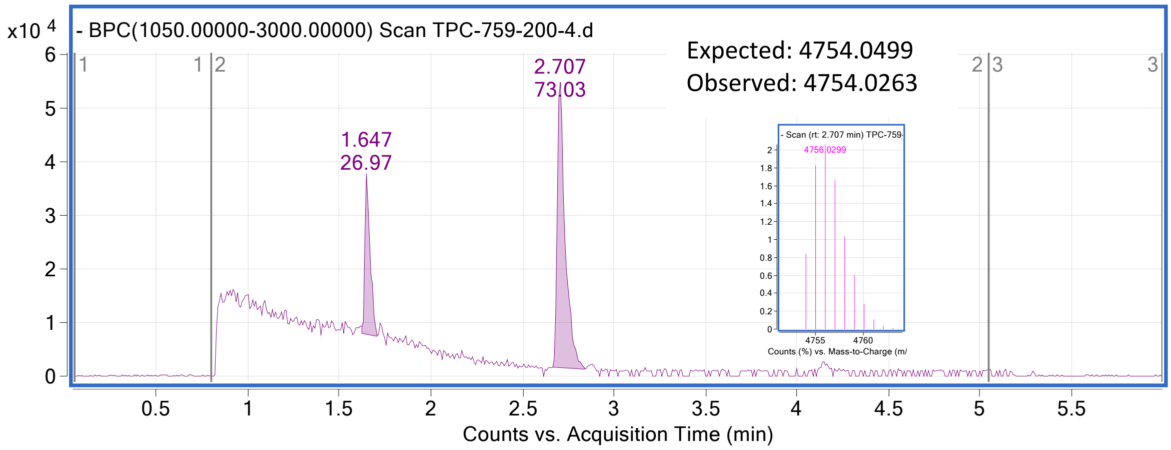

Figure S91: Mass spectrum of product 4 synthesised from HP2 and aldehyde 3 with TTAC as the surfactant analysed by DNA mass spectrometry gradient B.

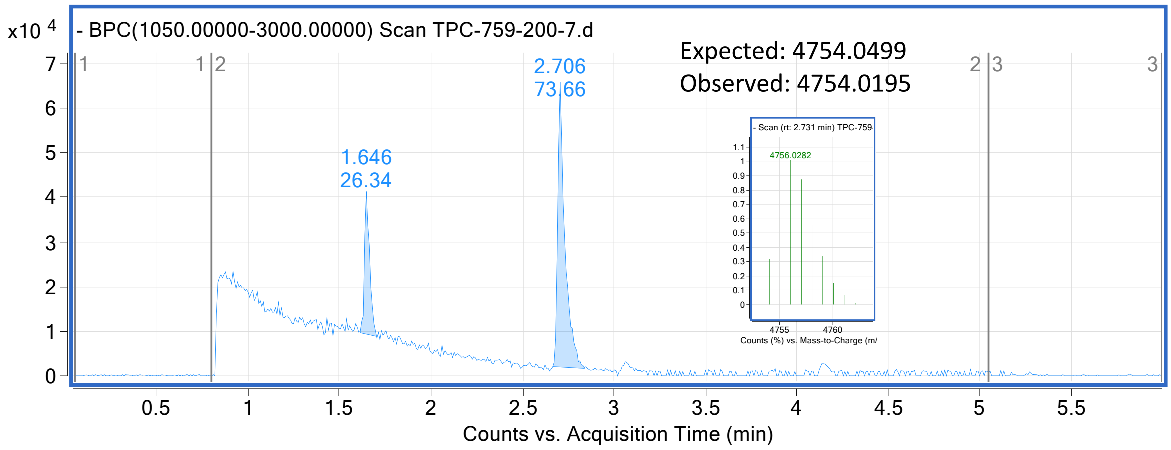

Figure S92: Mass spectrum of product 4 synthesised from HP2 and aldehyde 3 with sulfobetaine-16 as the surfactant analysed by DNA mass spectrometry gradient B.

Expected: 4754.0499
Observed: 4754.0073

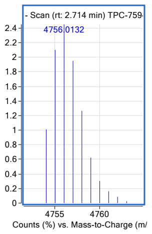

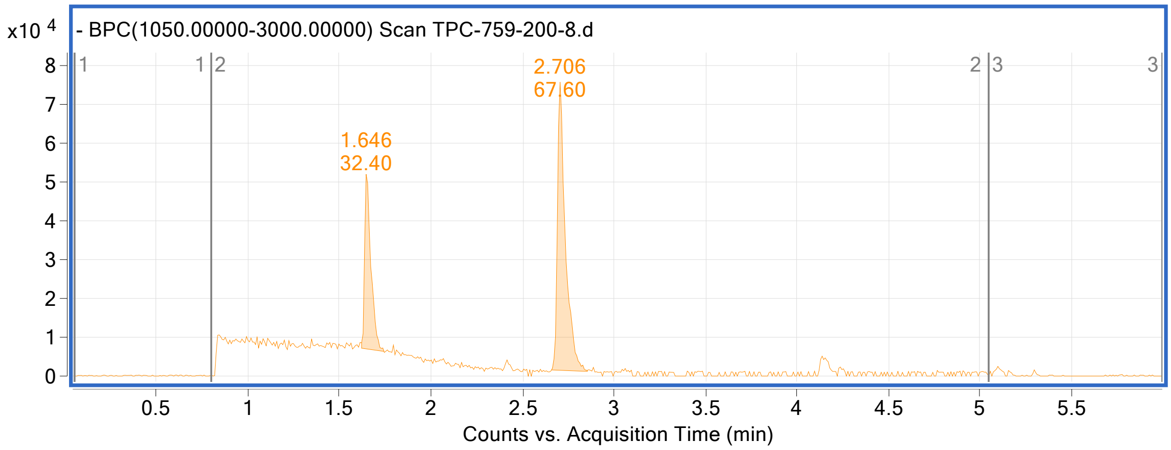

Table 6 Chromatograms

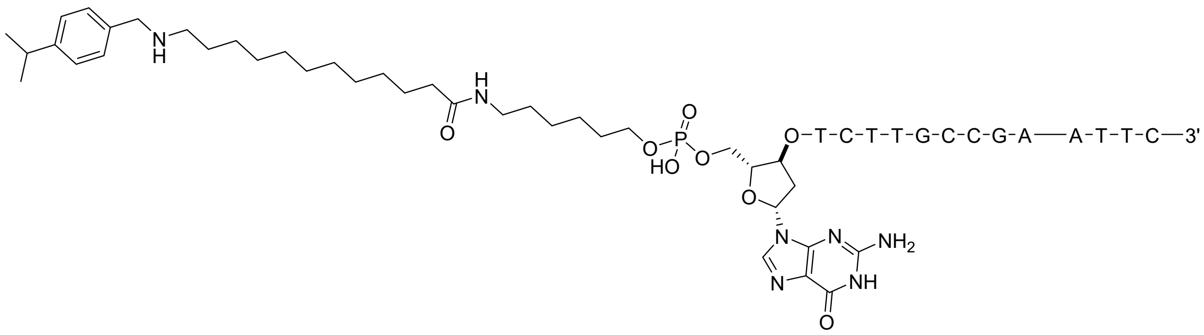

Figure S93: Mass spectrum of DNA-tagged product of reductive amination of HP2 and 4-isopropylbenzaldehyde using TPGS-750-M as the surfactant analysed by DNA mass spectrometry gradient C.

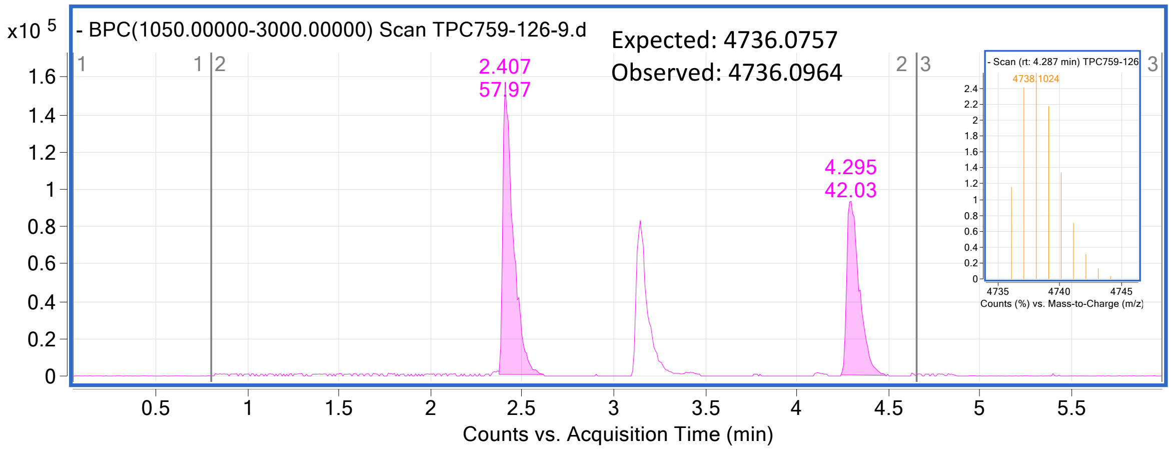

Figure S94: Mass spectrum of DNA-tagged product of reductive amination of HP2 and 4-isopropylbenzaldehyde using Triton-X-405 as the surfactant analysed by DNA mass spectrometry gradient A.

Expected: 4736.0757
Observed: 4736.0608

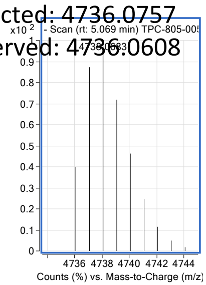

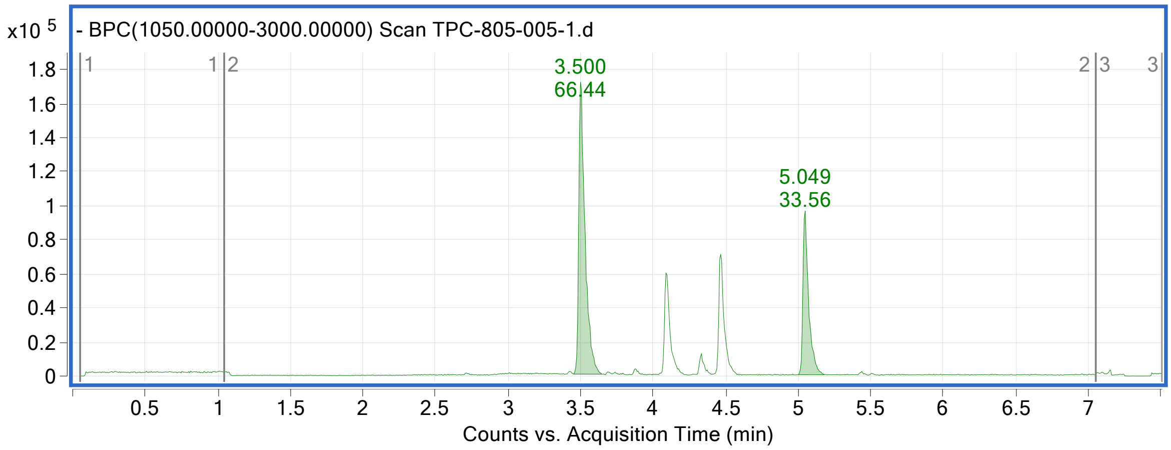

Figure S95: Mass spectrum of DNA-tagged product of reductive amination of HP2 and 4-isopropylbenzaldehyde using TTAC as the surfactant analysed by DNA mass spectrometry gradient A.

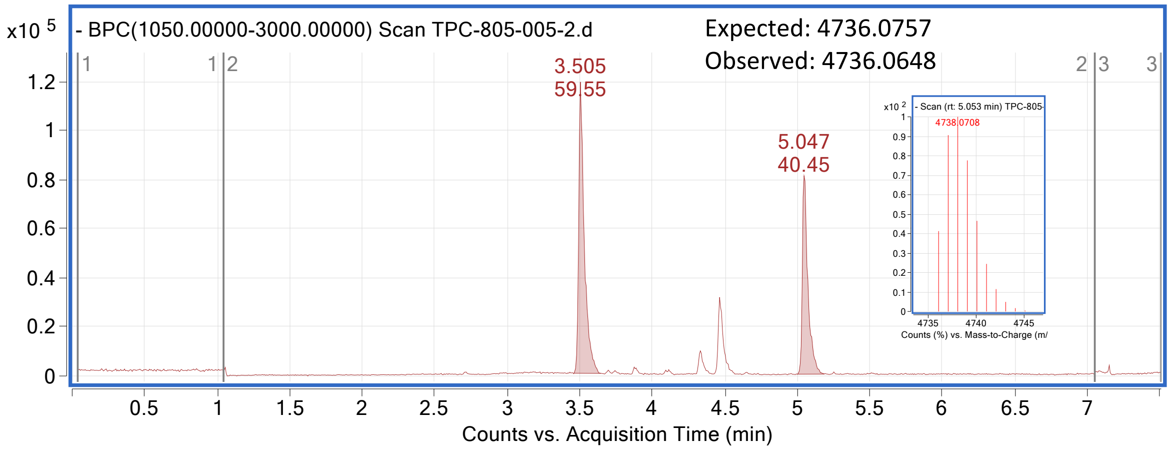

Figure S96: Mass spectrum of DNA-tagged product of reductive amination of HP2 and 4-isopropylbenzaldehyde using sulfobetaine-16 as the surfactant analysed by DNA mass spectrometry gradient A.

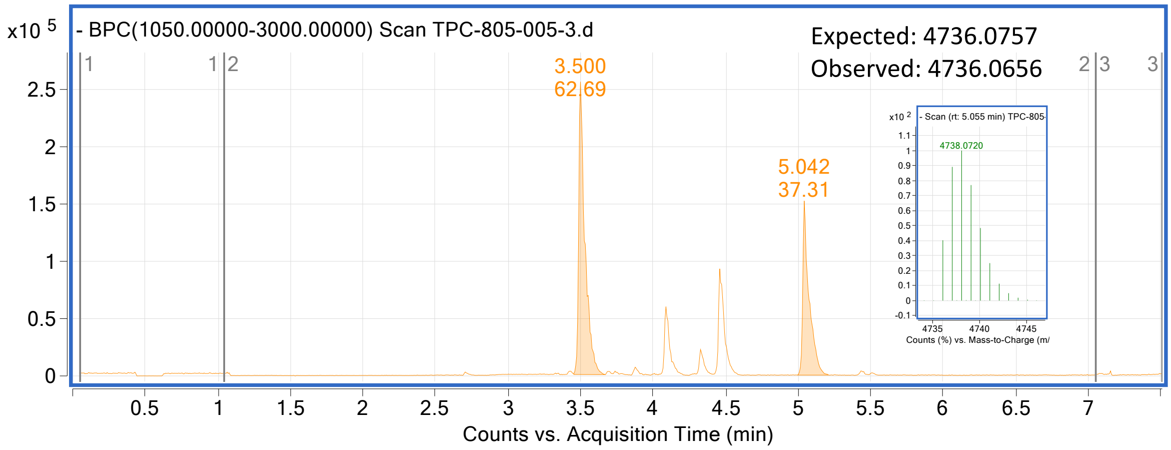

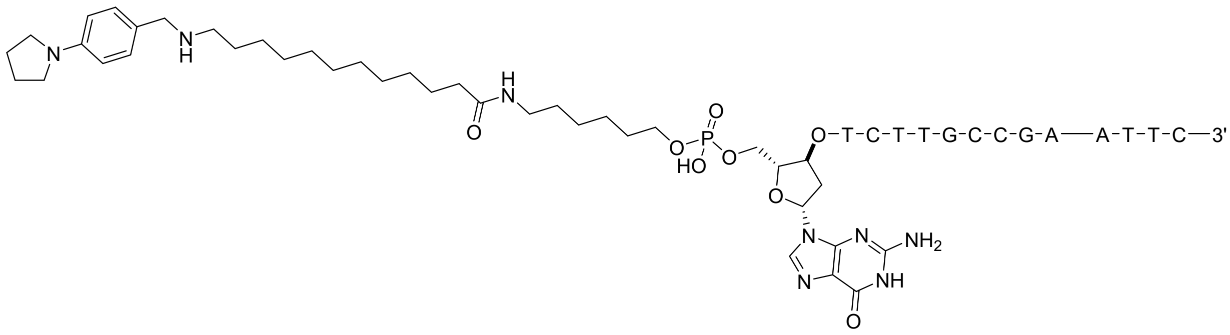

Figure S97: Mass spectrum of DNA-tagged product of reductive amination of HP2 and 4-(pyrrolidin-1-yl)benzaldehyde using TPGS-750-M as the surfactant analysed by DNA mass spectrometry gradient D.

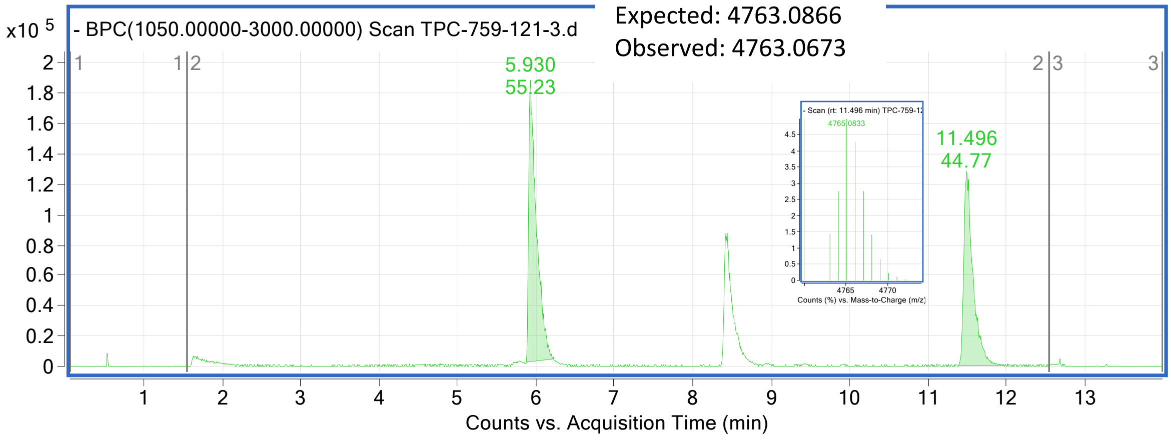

Figure S98: Mass spectrum of DNA-tagged product of reductive amination of HP2 and 4-(pyrrolidin-1-yl)benzaldehyde using Triton-X-405 as the surfactant analysed by DNA mass spectrometry gradient A.

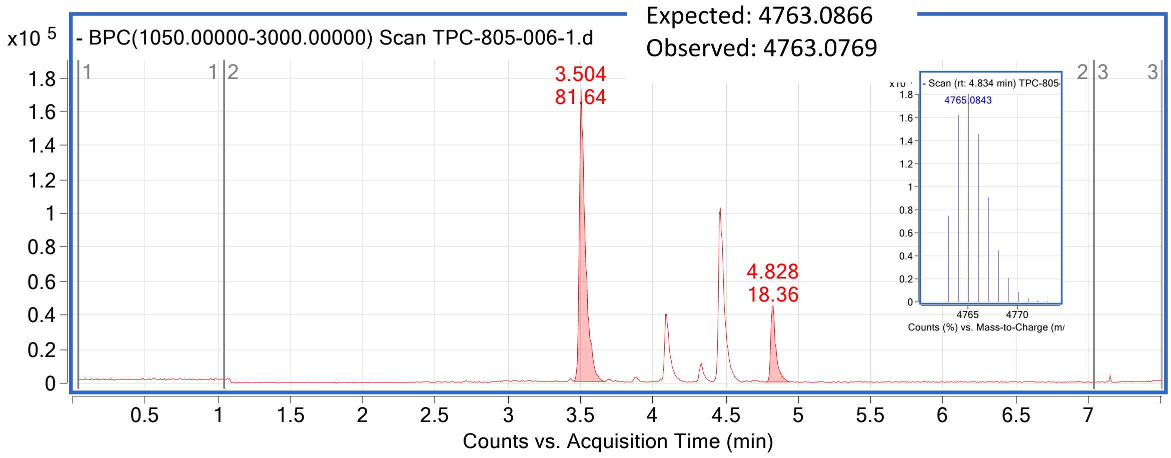

Figure S99: Mass spectrum of DNA-tagged product of reductive amination of HP2 and 4-(pyrrolidin-1-yl)benzaldehyde using TTAC as the surfactant analysed by DNA mass spectrometry gradient A.

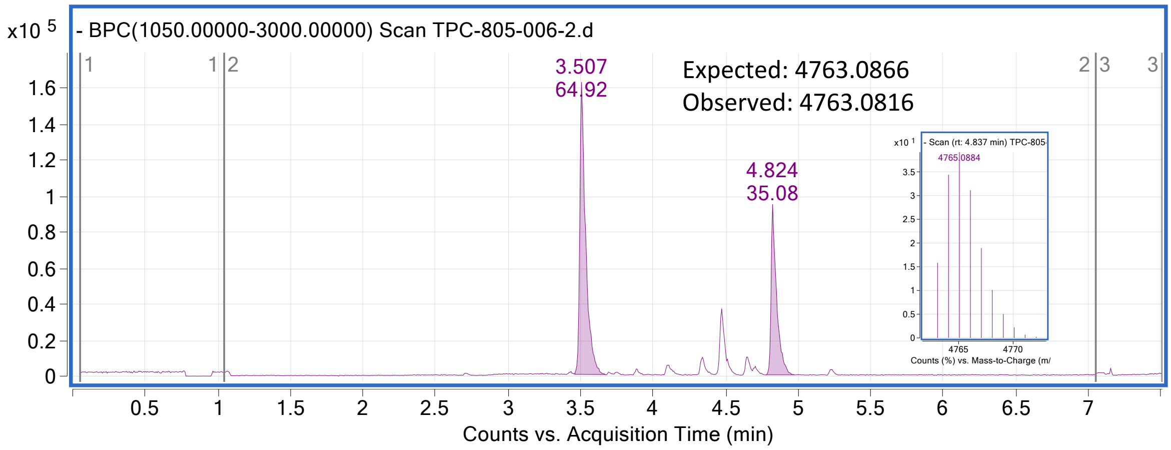

Figure S100: Mass spectrum of DNA-tagged product of reductive amination of HP2 and 4-(pyrrolidin-1-yl)benzaldehyde using sulfobetaine-16 as the surfactant analysed by DNA mass spectrometry gradient A.

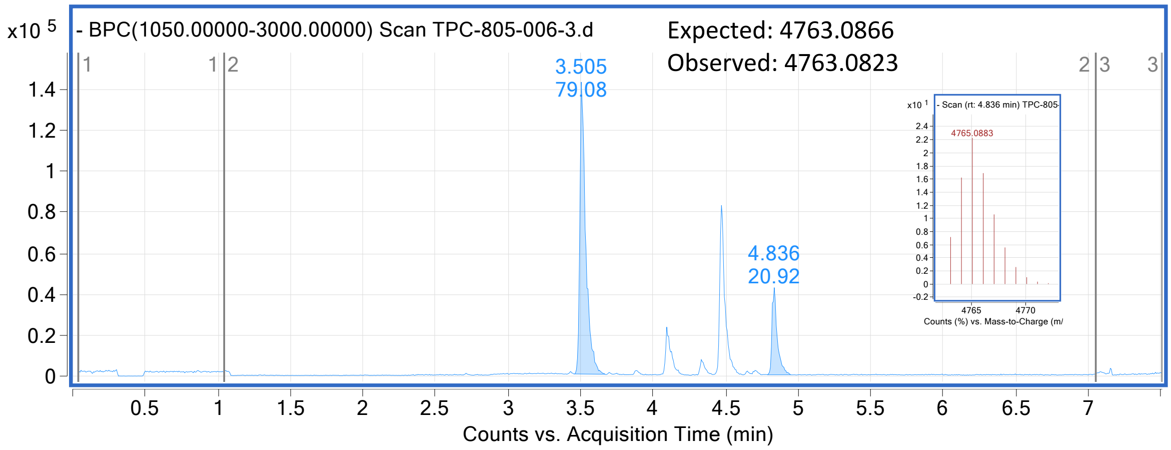

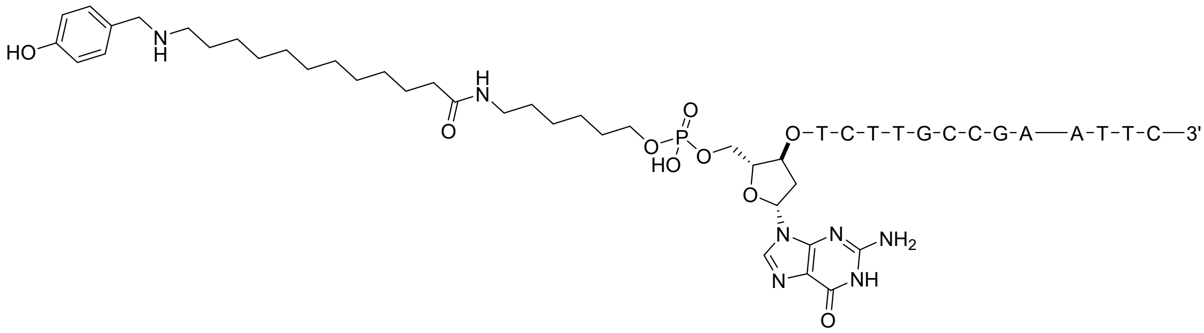

Figure S101: Mass spectrum of DNA-tagged product of reductive amination of HP2 and 4-hydroxybenzaldehyde using TPGS-750-M as the surfactant analysed by DNA mass spectrometry gradient C.

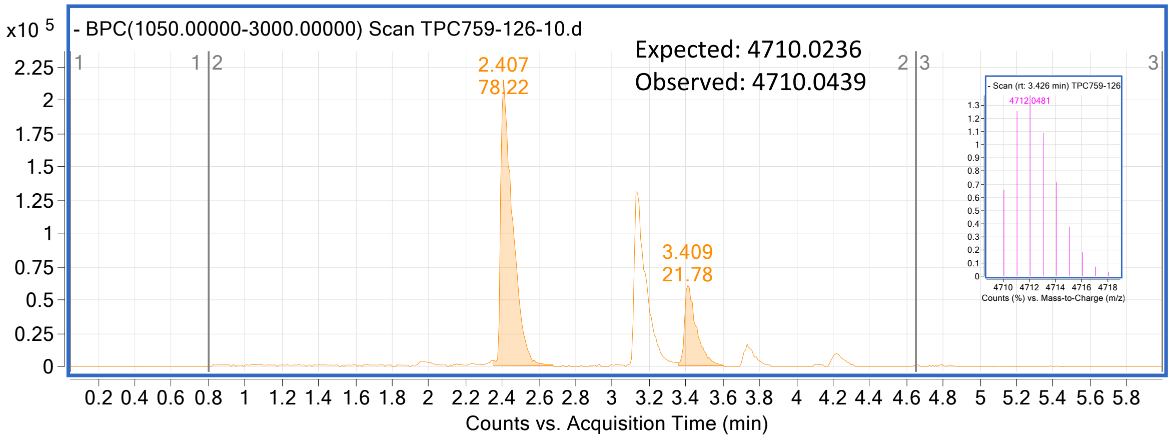

Figure S102: Mass spectrum of DNA-tagged product of reductive amination of HP2 and 4-hydroxybenzaldehyde using Triton-X-405 as the surfactant analysed by DNA mass spectrometry gradient A.

Expected: 4710.0236
Observed: 4710.0043

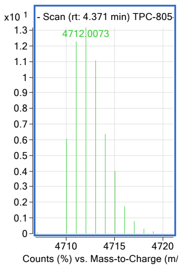

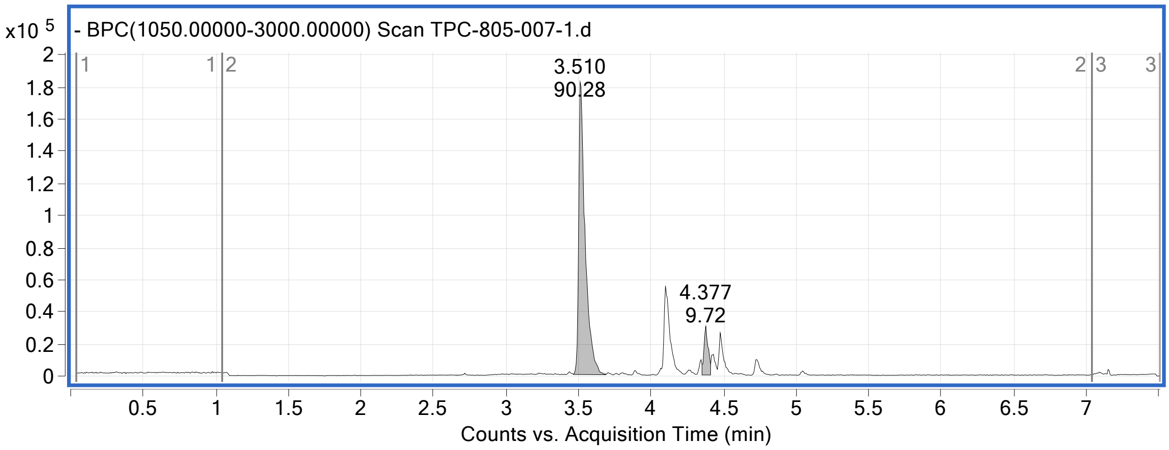

Figure S103: Mass spectrum of DNA-tagged product of reductive amination of HP2 and 4-hydroxybenzaldehyde using TTAC as the surfactant analysed by DNA mass spectrometry gradient A.

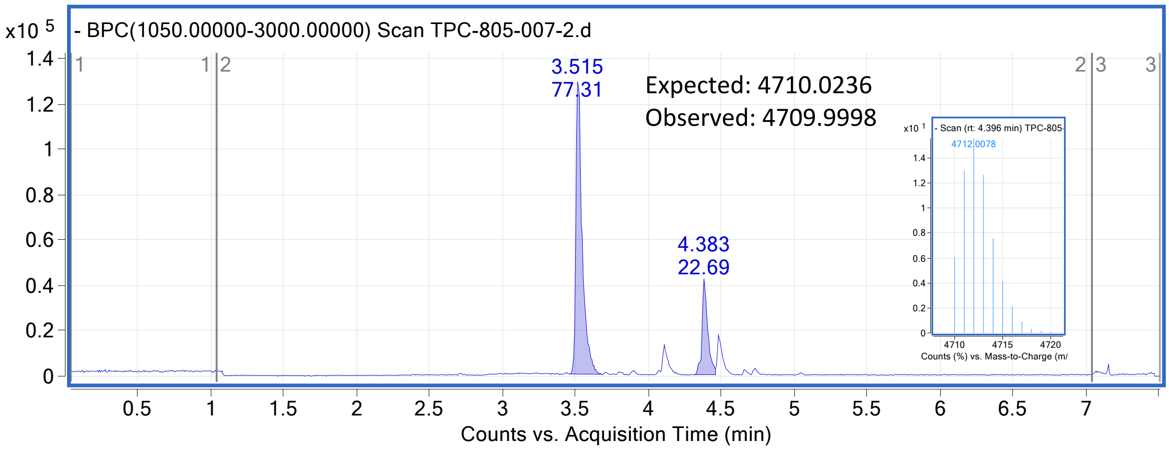

Figure S104: Mass spectrum of DNA-tagged product of reductive amination of HP2 and 4-hydroxybenzaldehyde using sulfobetaine-16 as the surfactant analysed by DNA mass spectrometry gradient A.

Expected: 4710.0236
Observed: 4710.0000

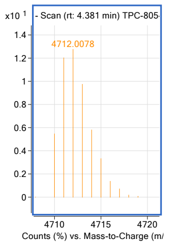

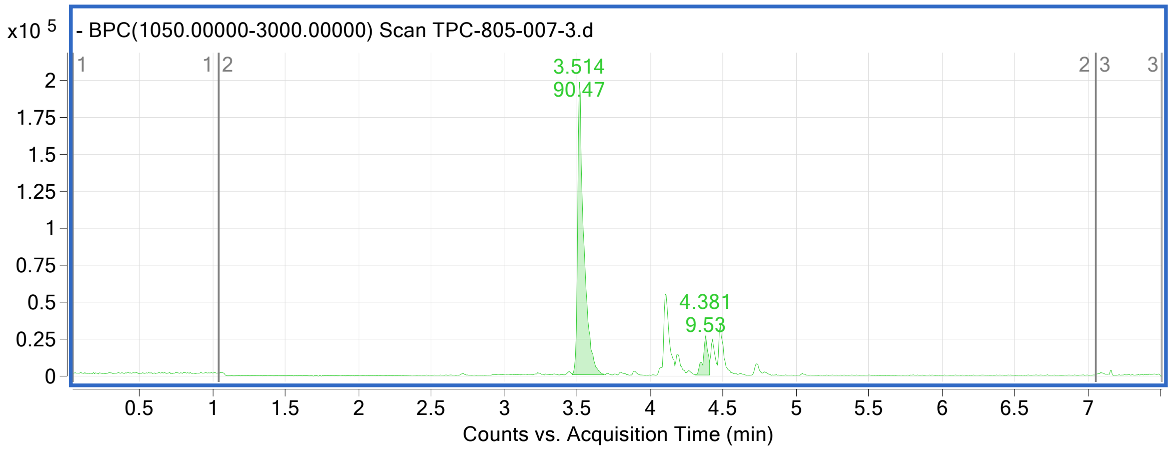

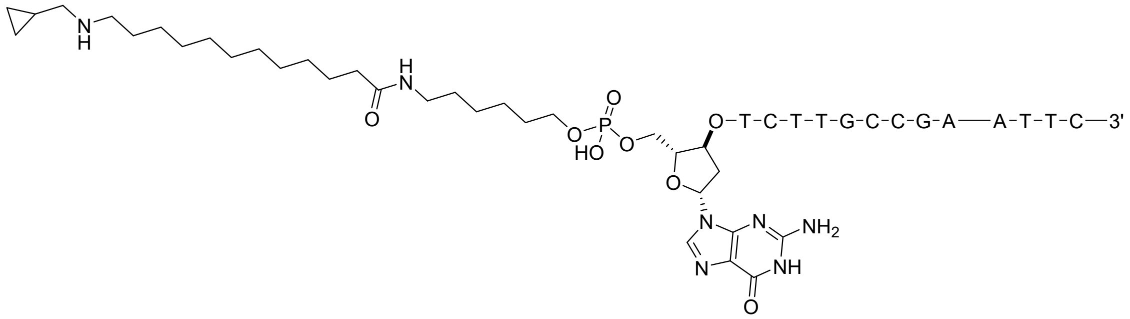

Figure S105: Mass spectrum of DNA-tagged product of reductive amination of HP2 and cyclopropanecarbaldehyde using TPGS-750-M as the surfactant analysed by DNA mass spectrometry gradient C.

Expected: 4658.0287
Observed: 4658.0315

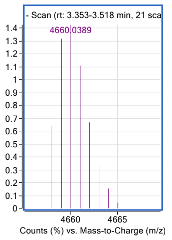

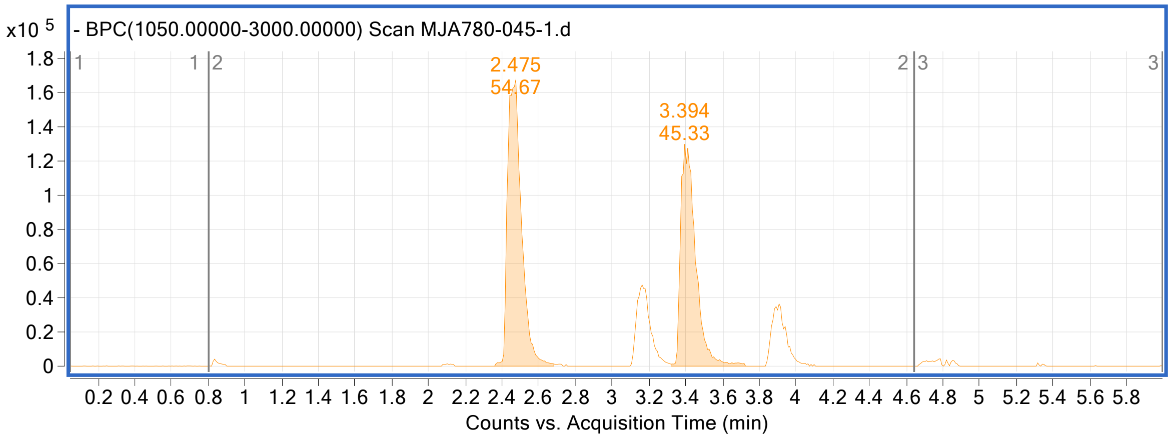

Figure S106: Mass spectrum of DNA-tagged product of reductive amination of HP2 and cyclopropanecarbaldehyde using Triton-X-405 as the surfactant analysed by DNA mass spectrometry gradient A.

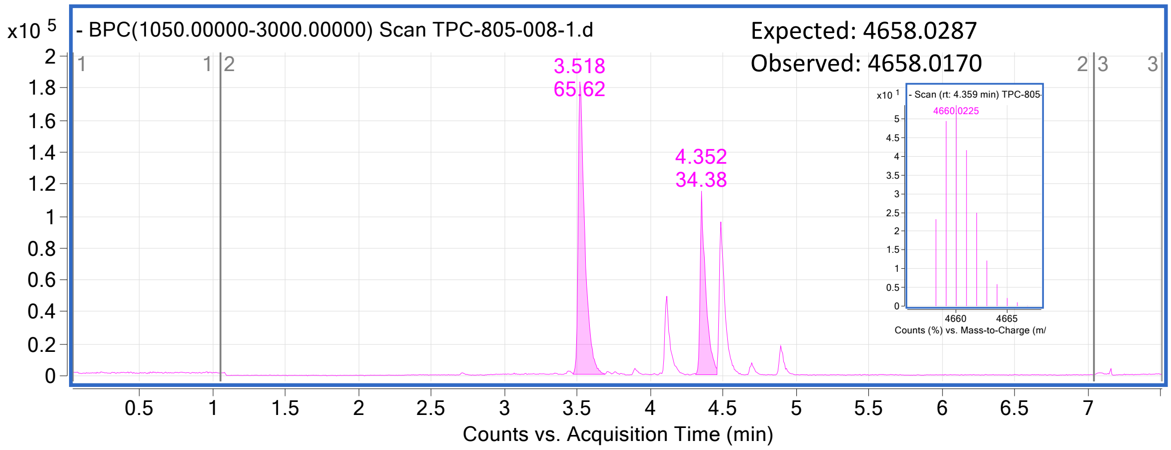

Figure S107: Mass spectrum of DNA-tagged product of reductive amination of HP2 and cyclopropanecarbaldehyde using TTAC as the surfactant analysed by DNA mass spectrometry gradient A.

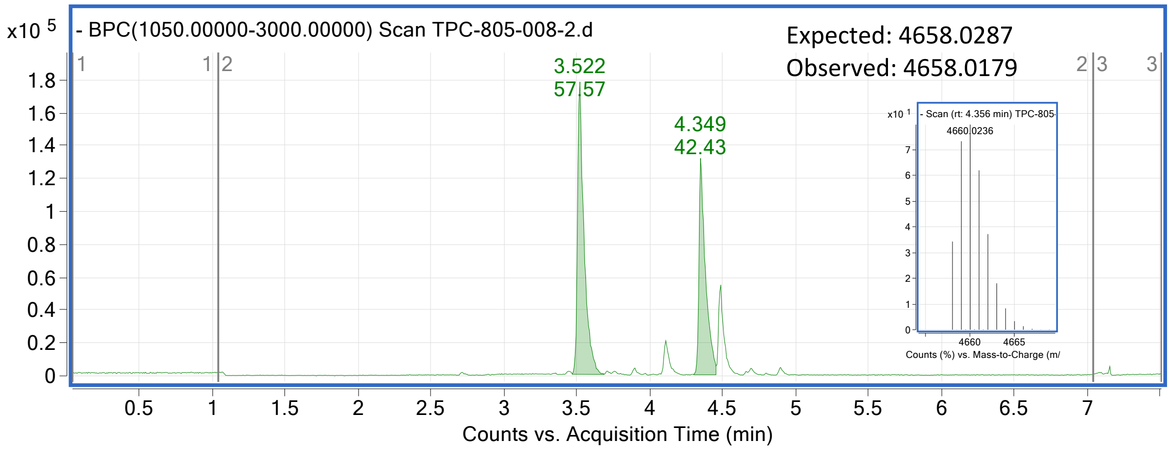

Figure S108: Mass spectrum of DNA-tagged product of reductive amination of HP2 and cyclopropanecarbaldehyde using Sulfobetaine-16 as the surfactant analysed by DNA mass spectrometry gradient A.

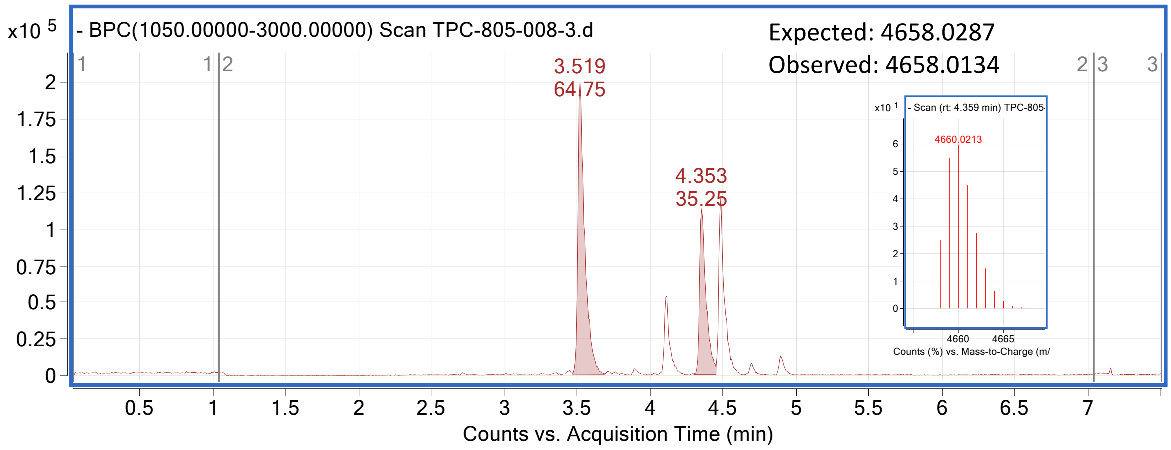

Table 7 Chromatograms

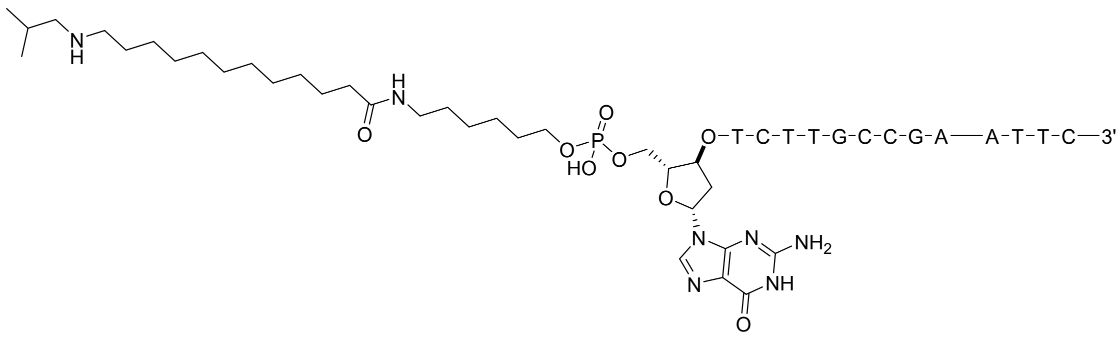

Figure S109: Mass spectrum of DNA-tagged product of reductive amination of HP2 and isobutyraldehyde using TPGS-750-M as the surfactant analysed by DNA mass spectrometry gradient A.

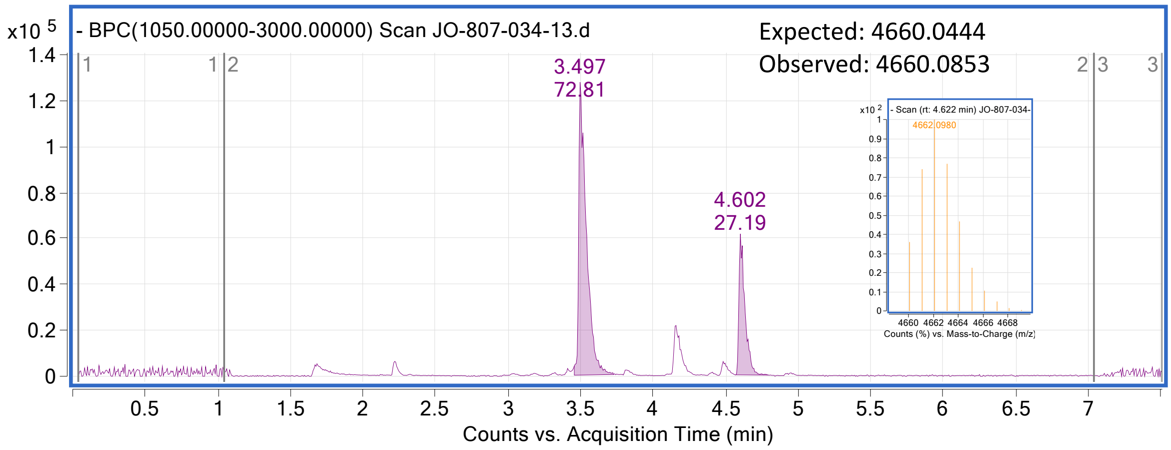

Figure S110: Mass spectrum of DNA-tagged product of reductive amination of HP2 and isobutyraldehyde using TTAC as the surfactant analysed by DNA mass spectrometry gradient A.

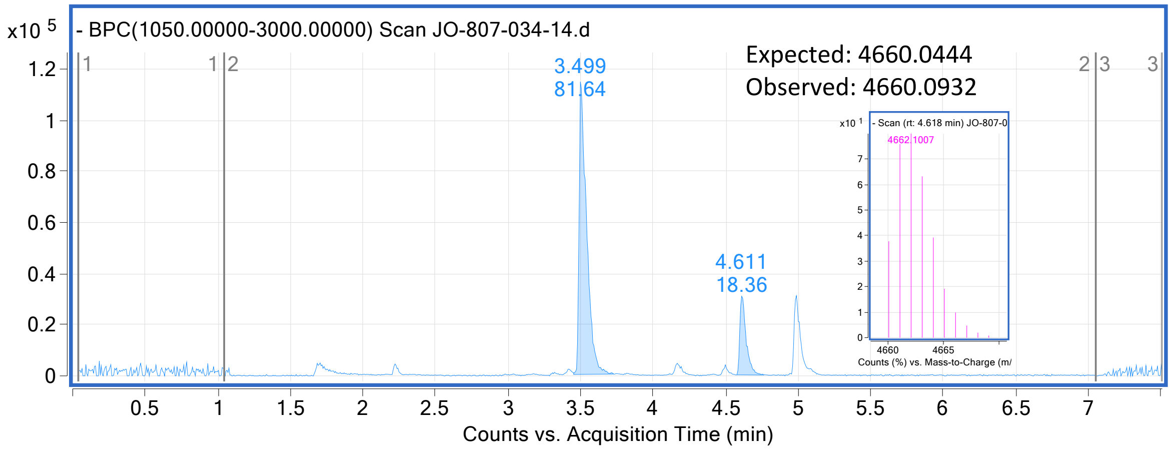

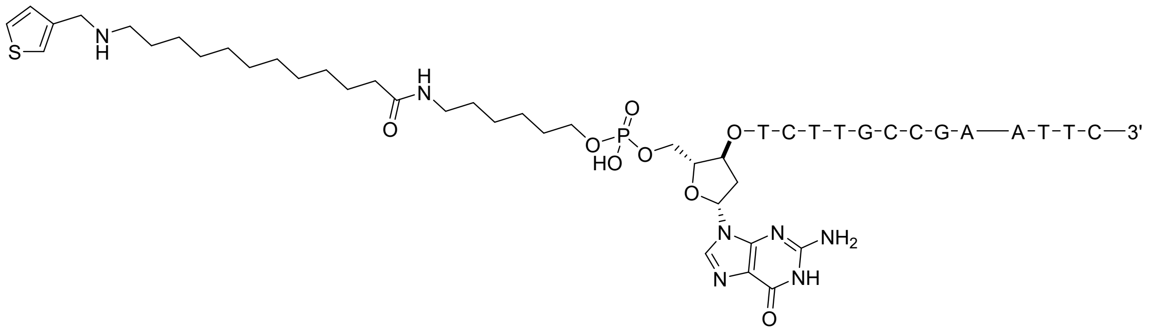

Figure S111: Mass spectrum of DNA-tagged product of reductive amination of HP2 and thiophene-3-carbaldehyde using TPGS-750-M as the surfactant analysed by DNA mass spectrometry gradient A.

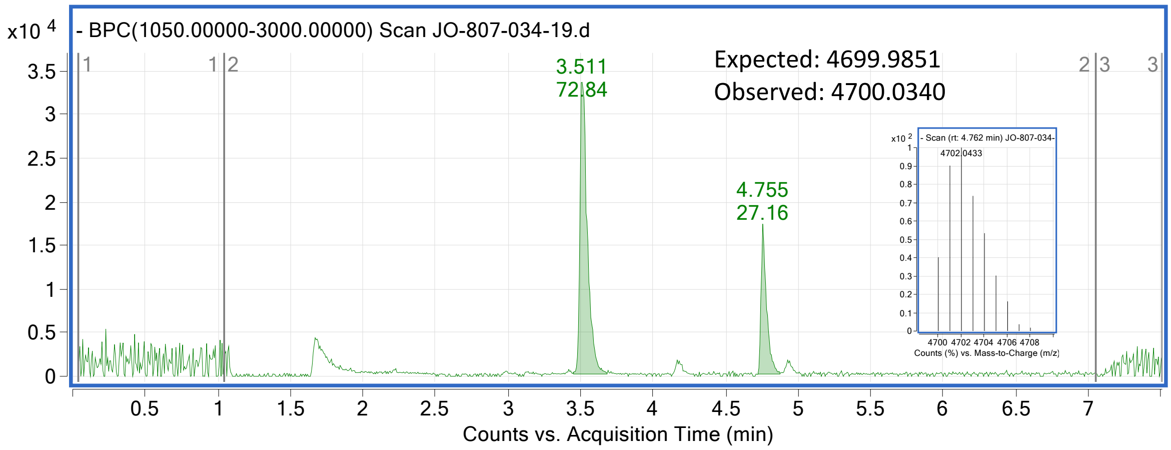

Figure S112: Mass spectrum of DNA-tagged product of reductive amination of HP2 and thiophene-3-carbaldehyde using TTAC as the surfactant analysed by DNA mass spectrometry gradient A.

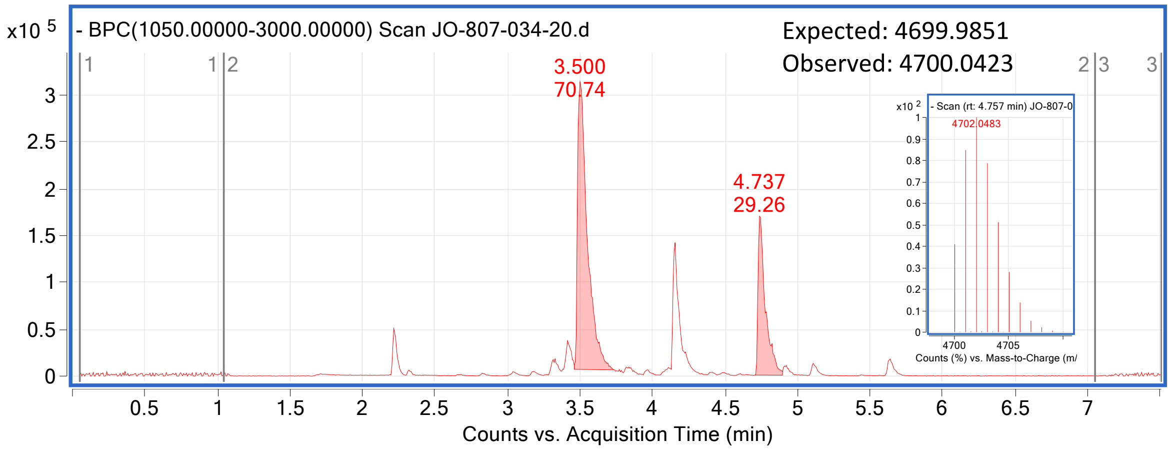

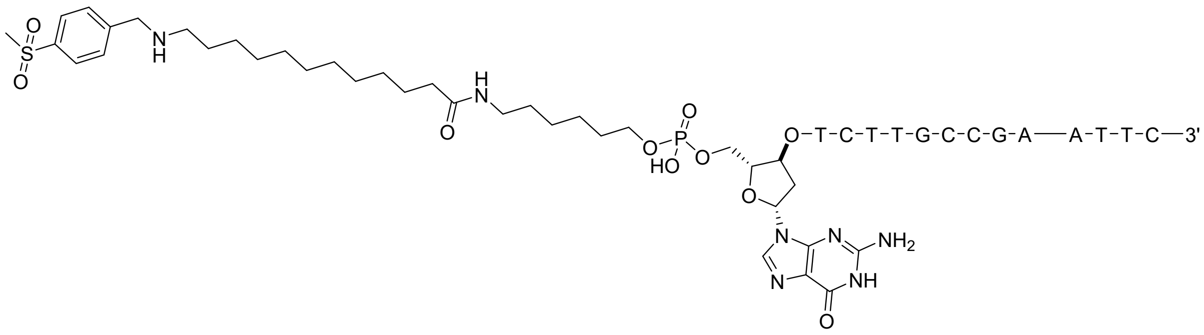

Figure S113: Mass spectrum of DNA-tagged product of reductive amination of HP2 and 4-(methylsulfonyl)benzaldehyde using TPGS-750-M as the surfactant analysed by DNA mass spectrometry gradient A.

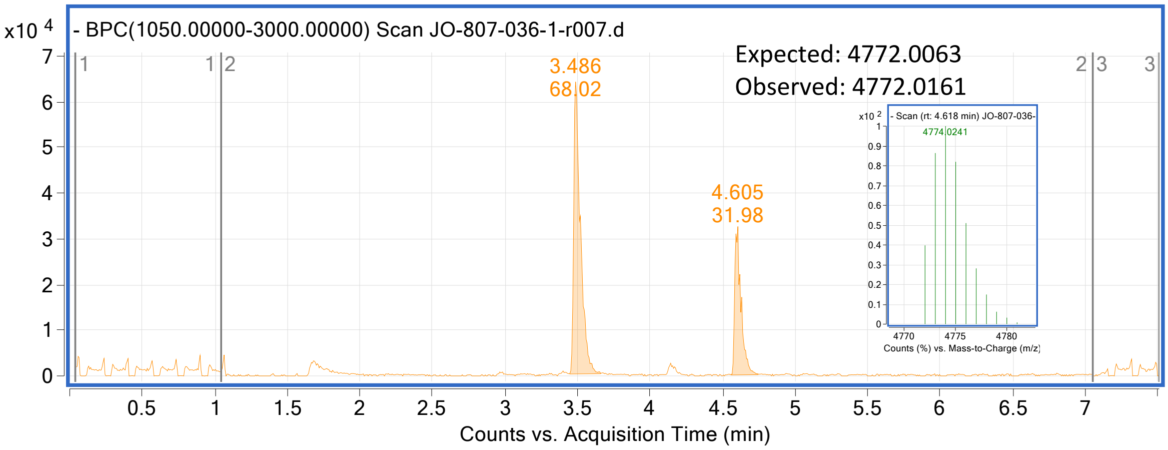

Figure S114: Mass spectrum of DNA-tagged product of reductive amination of HP2 and 4-(methylsulfonyl)benzaldehyde using TTAC as the surfactant analysed by DNA mass spectrometry gradient A.

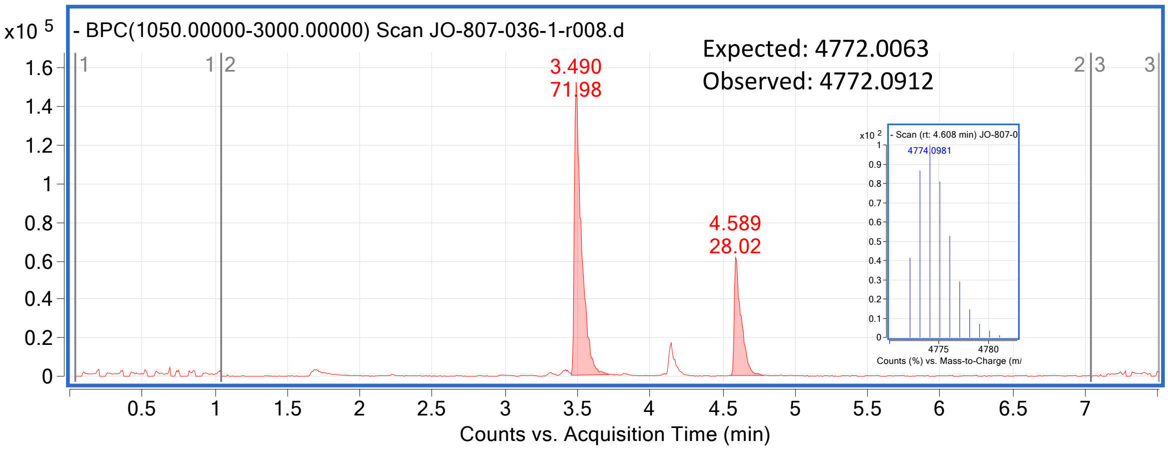

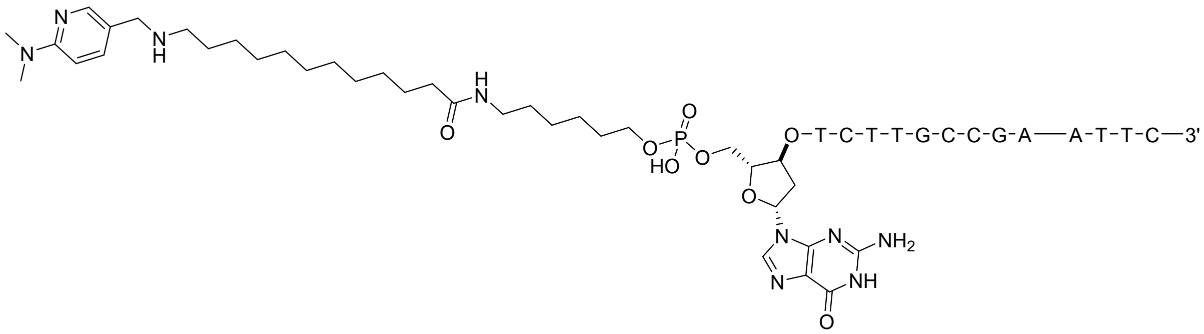

Figure S115: Mass spectrum of DNA-tagged product of reductive amination of HP2 and 6-(dimethylamino)nicotinaldehyde using TPGS-750-M as the surfactant analysed by DNA mass spectrometry gradient A.

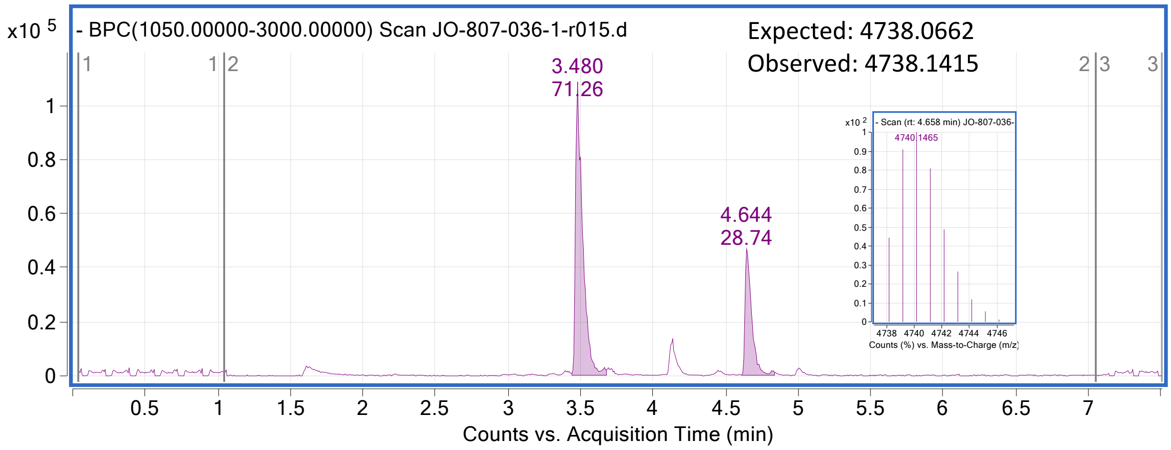

Figure S116: Mass spectrum of DNA-tagged product of reductive amination of HP2 and 6-(dimethylamino)nicotinaldehyde using TTAC as the surfactant analysed by DNA mass spectrometry gradient A.

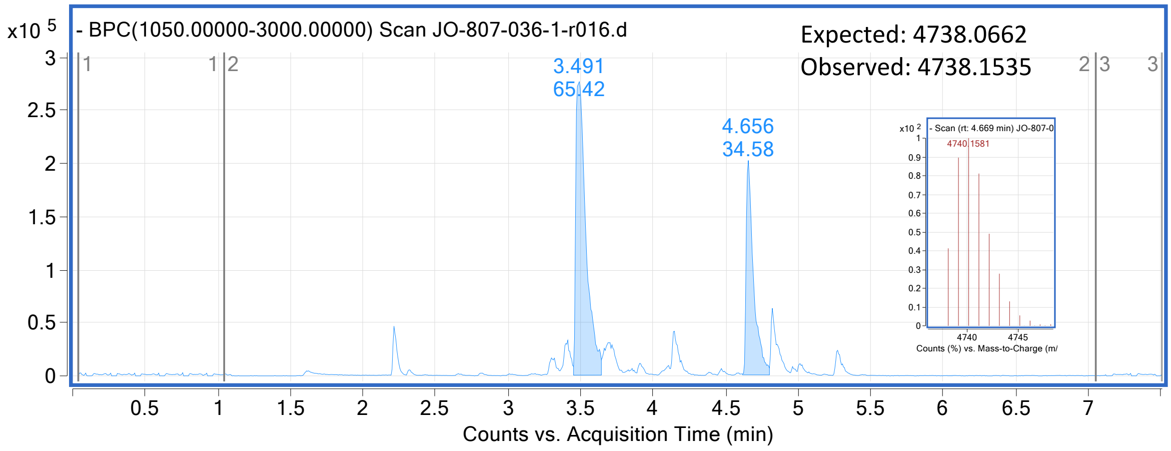

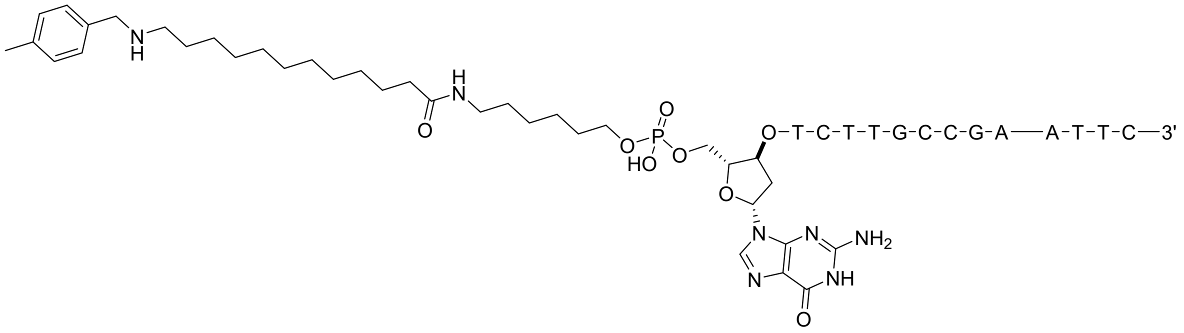

Figure S117: Mass spectrum of DNA-tagged product of reductive amination of HP2 and 4-methylbenzaldehyde using TPGS-750-M as the surfactant analysed by DNA mass spectrometry gradient A.

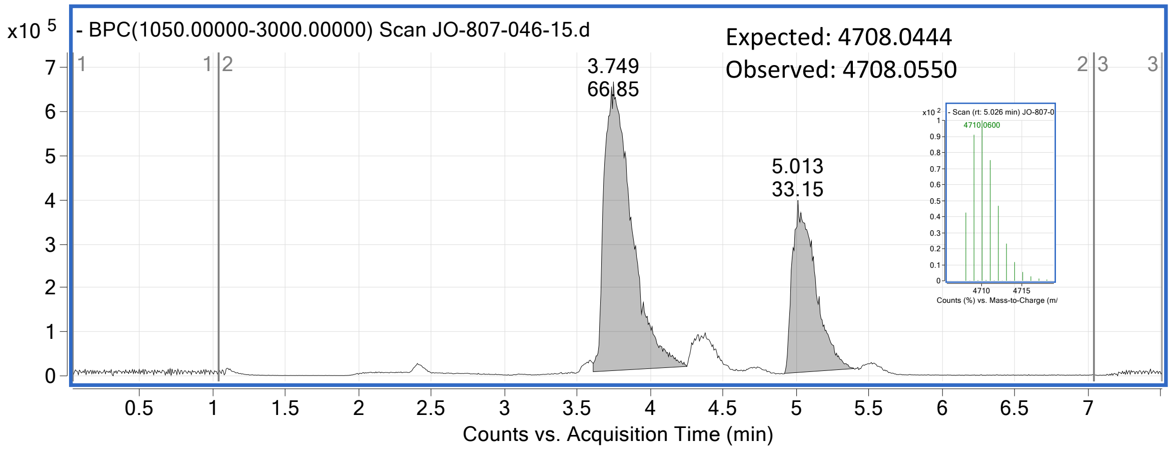

Figure S118: Mass spectrum of DNA-tagged product of reductive amination of HP2 and 4-methylbenzaldehyde using TTAC as the surfactant analysed by DNA mass spectrometry gradient A.

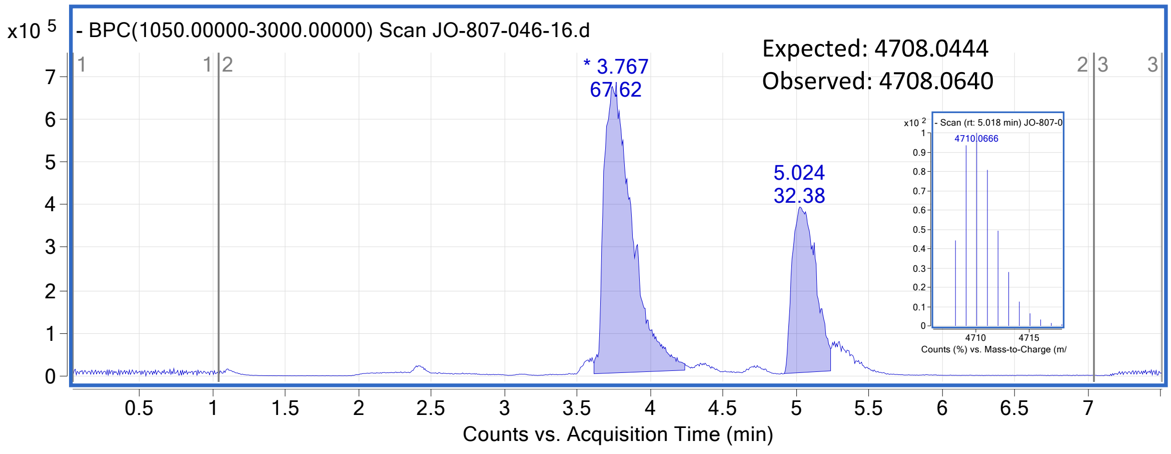

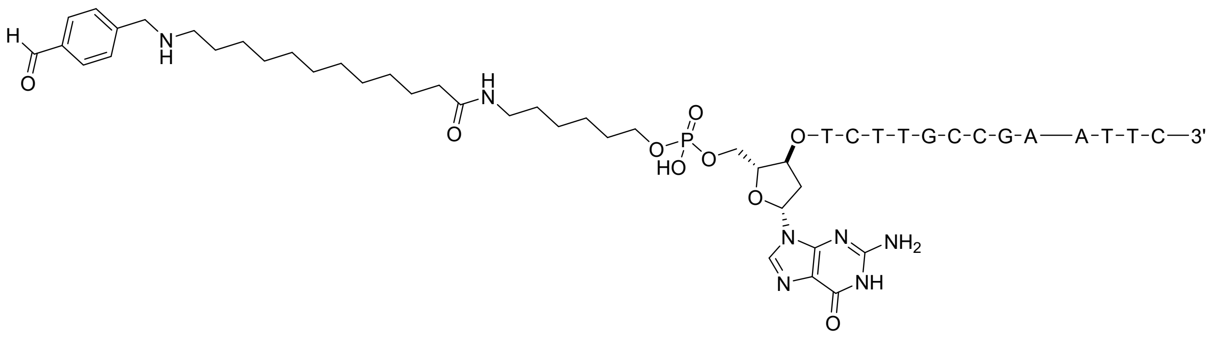

Figure S119: Mass spectrum of DNA-tagged product of reductive amination of HP2 and terephthalaldehyde using TPGS-750-M as the surfactant analysed by DNA mass spectrometry gradient A.

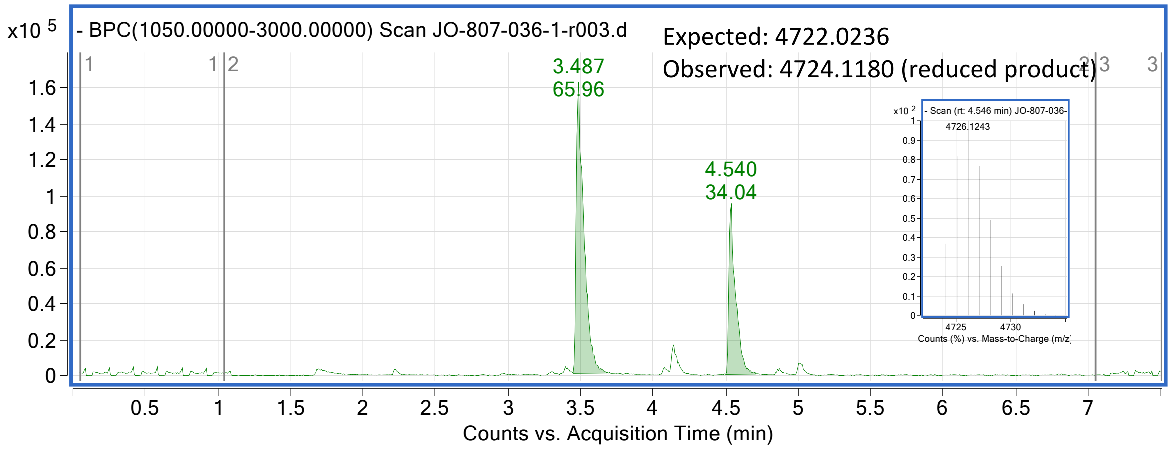

Figure S120: Mass spectrum of DNA-tagged product of reductive amination of HP2 and terephthalaldehyde using TTAC as the surfactant analysed by DNA mass spectrometry gradient A.

Expected: 4722.0236
Observed: 4724.1141 (reduced product)

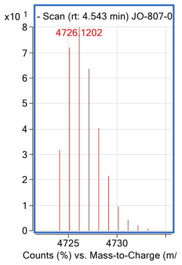

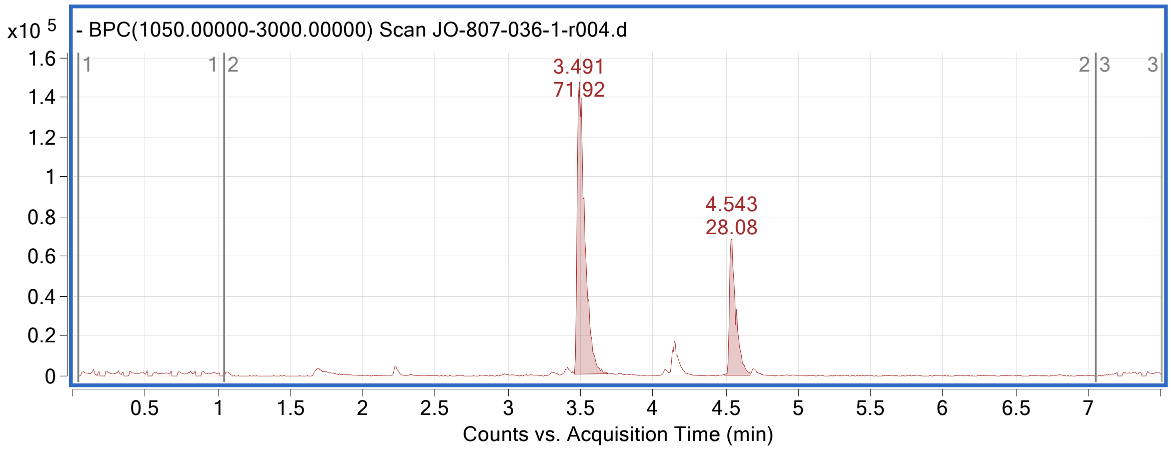

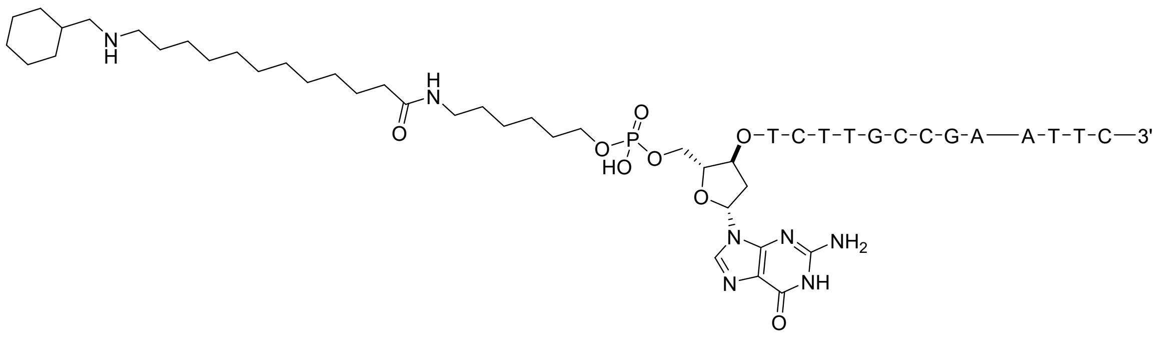

Figure S121: Mass spectrum of DNA-tagged product of reductive amination of HP2 and cyclohexanecarbaldehyde using TPGS-750-M as the surfactant analysed by DNA mass spectrometry gradient A.

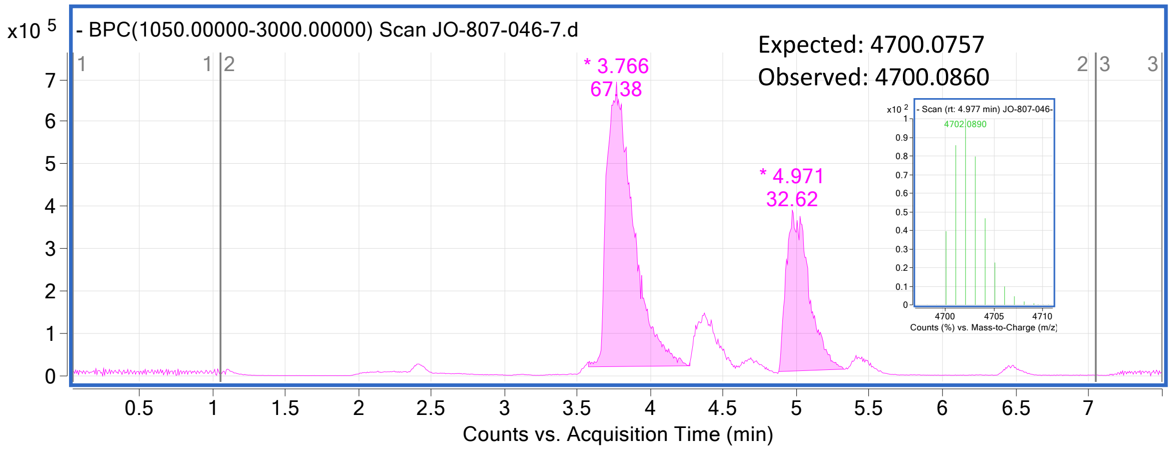

Figure S122: Mass spectrum of DNA-tagged product of reductive amination of HP2 and cyclohexanecarbaldehyde using TTAC as the surfactant analysed by DNA mass spectrometry gradient A.

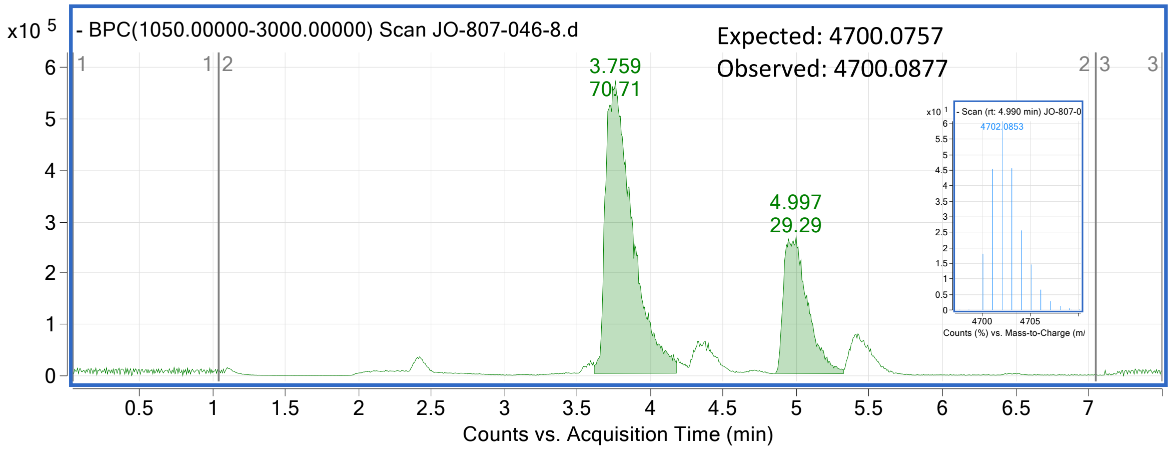

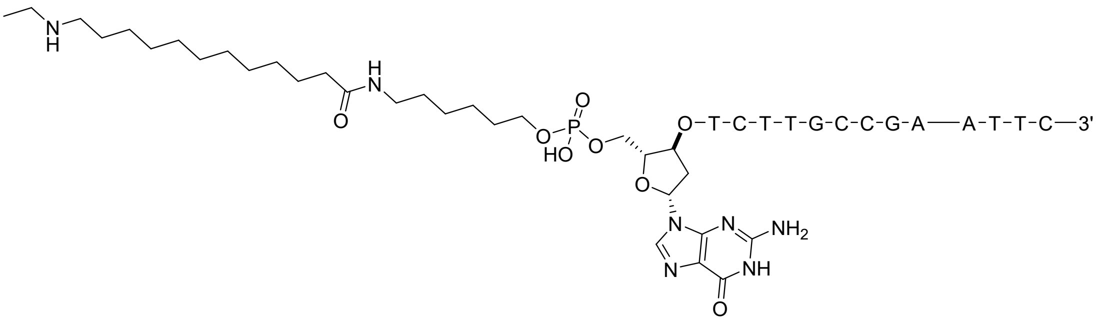

Figure S123: Mass spectrum of DNA-tagged product of reductive amination of HP2 and acetaldehyde using TPGS-750-M as the surfactant analysed by DNA mass spectrometry gradient A.

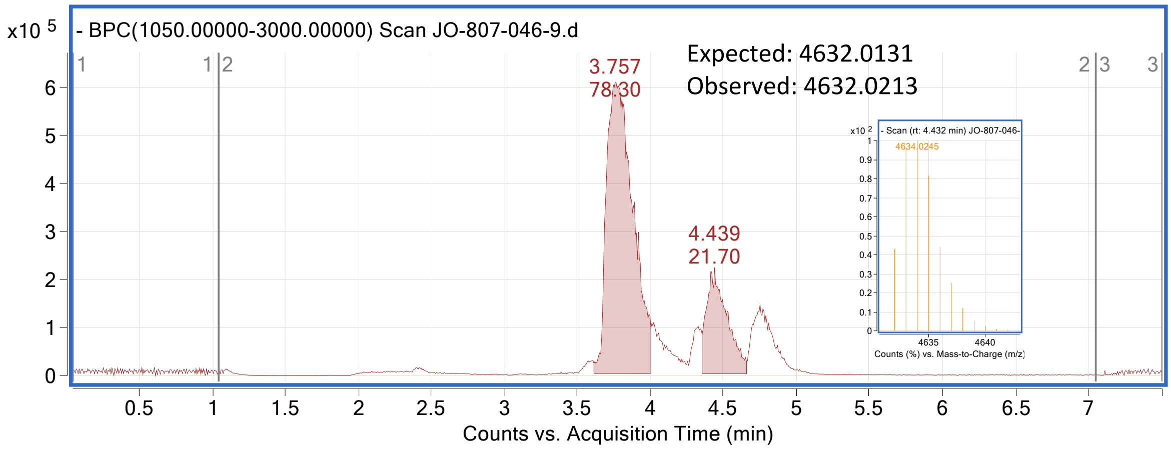

Figure S124: Mass spectrum of DNA-tagged product of reductive amination of HP2 and acetaldehyde using TTAC as the surfactant analysed by DNA mass spectrometry gradient A.

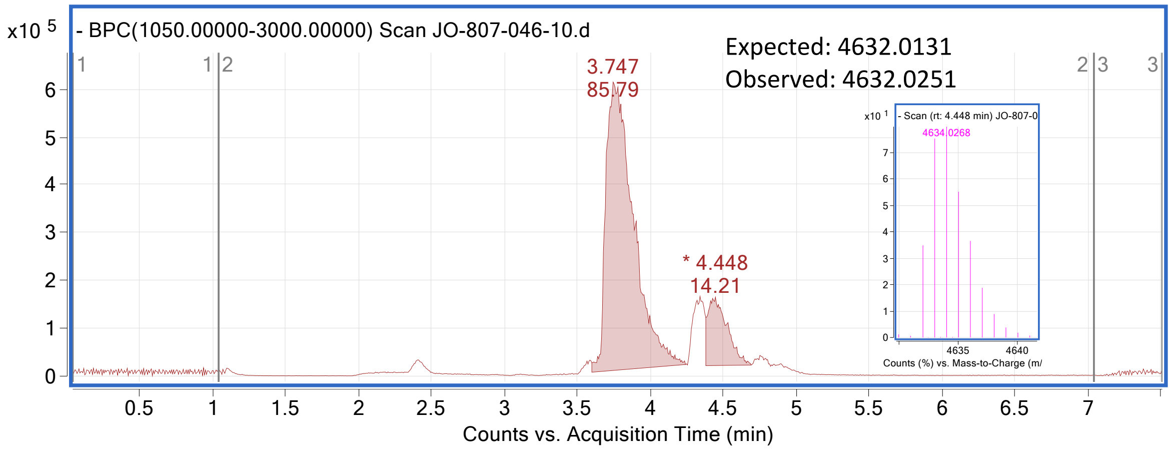

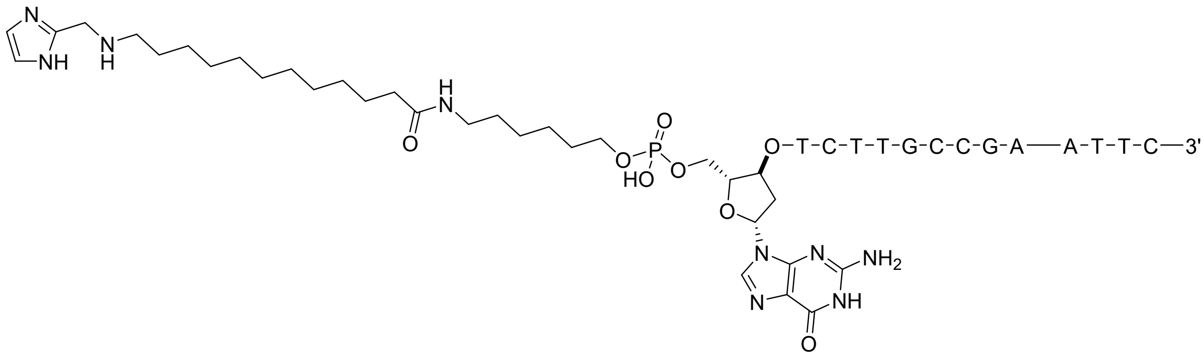

Figure S125: Mass spectrum of DNA-tagged product of reductive amination of HP2 and 1*H*-pyrrole-2-carbaldehyde using TPGS-750-M as the surfactant analysed by DNA mass spectrometry gradient A.

Expected: 4684.0192
Observed: 4684.0877

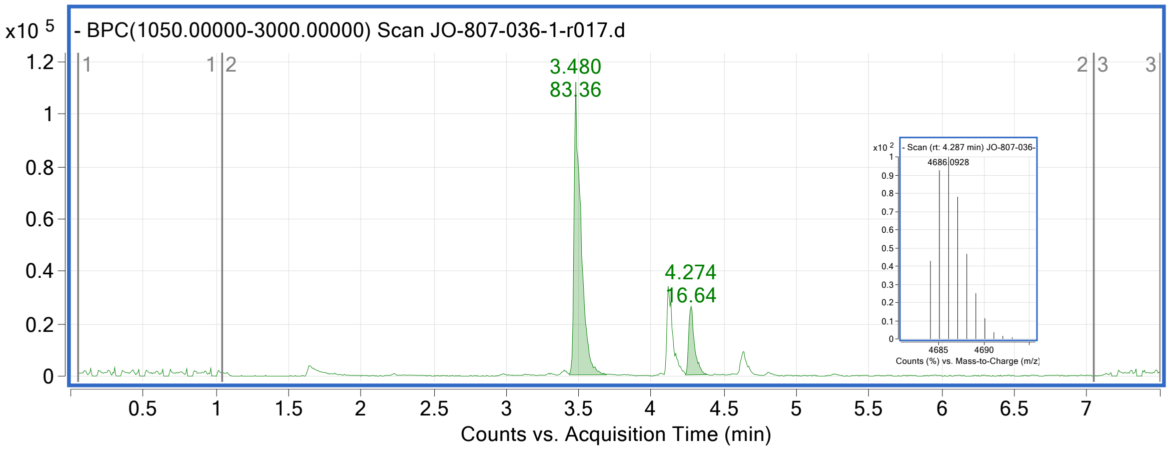

Figure S126: Mass spectrum of DNA-tagged product of reductive amination of HP2 and 1*H*-pyrrole-2-carbaldehyde using TTAC as the surfactant analysed by DNA mass spectrometry gradient A.

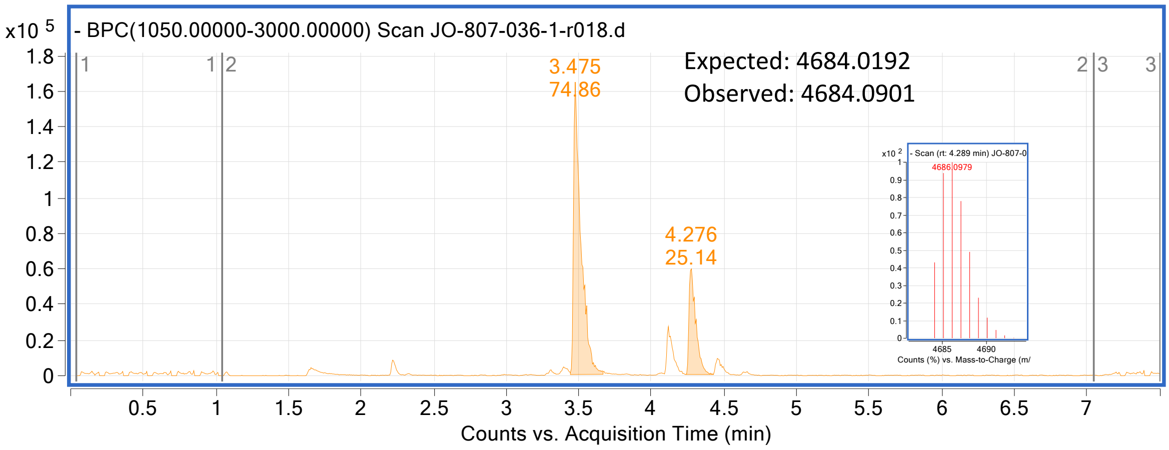

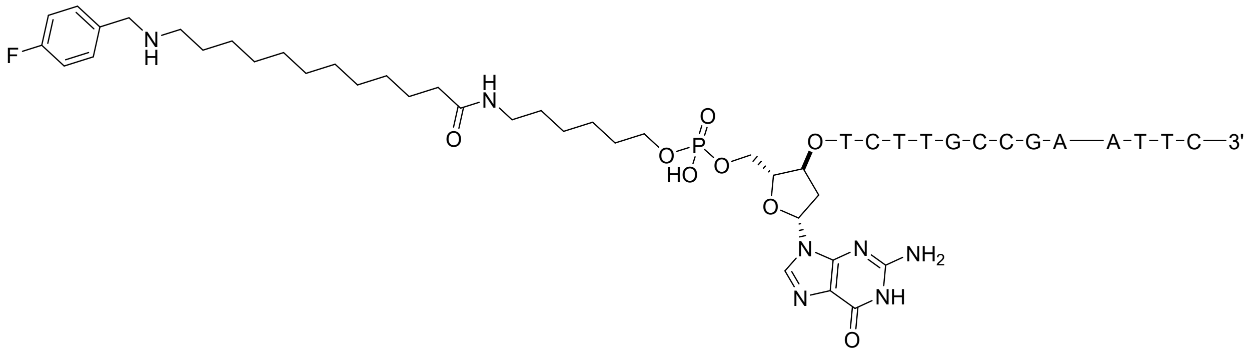

Figure S127: Mass spectrum of DNA-tagged product of reductive amination of HP2 and 4-fluorobenzaldehyde using TPGS-750-M as the surfactant analysed by DNA mass spectrometry gradient A.

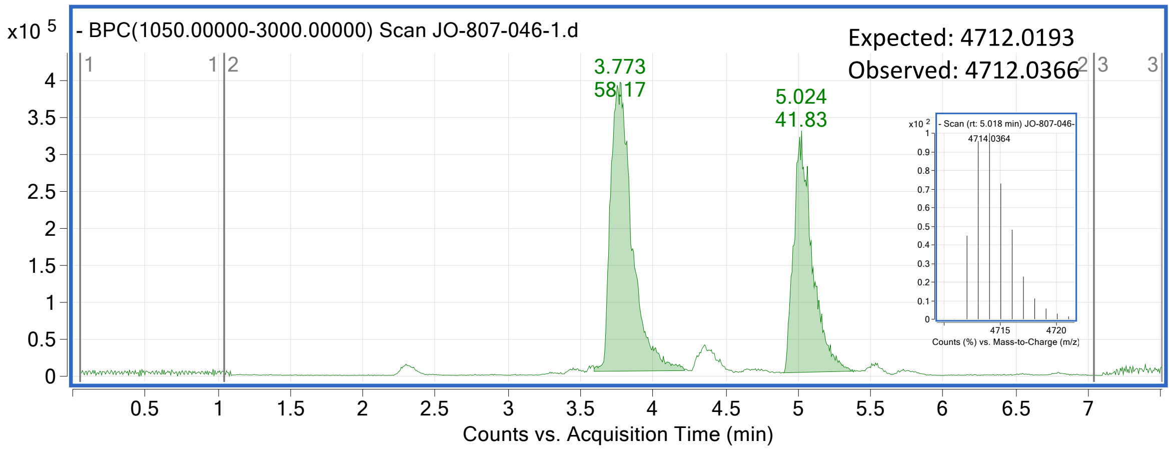

Figure S128: Mass spectrum of DNA-tagged product of reductive amination of HP2 and 4-fluorobenzaldehyde using TTAC as the surfactant analysed by DNA mass spectrometry gradient A.

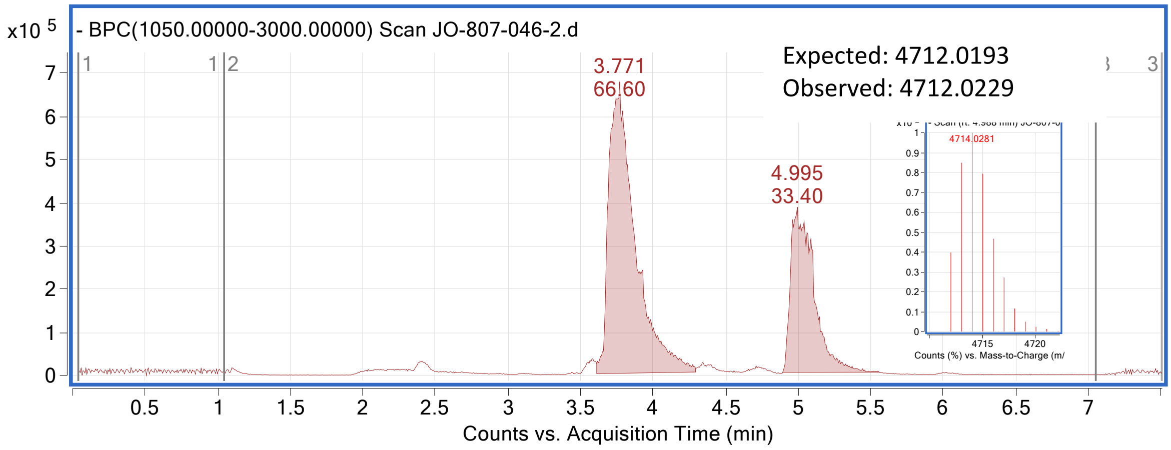

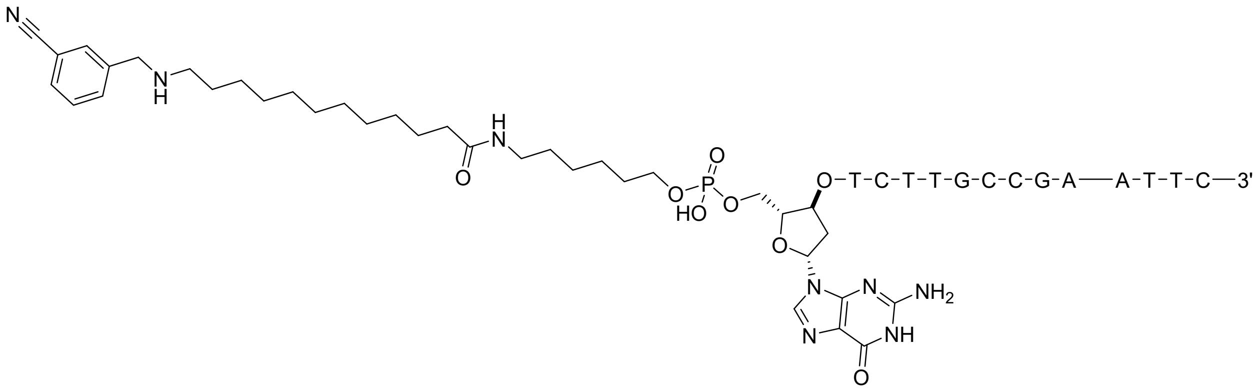

Figure S129: Mass spectrum of DNA-tagged product of reductive amination of HP2 and 3-formylbenzonitrile using TPGS-750-M as the surfactant analysed by DNA mass spectrometry gradient A.

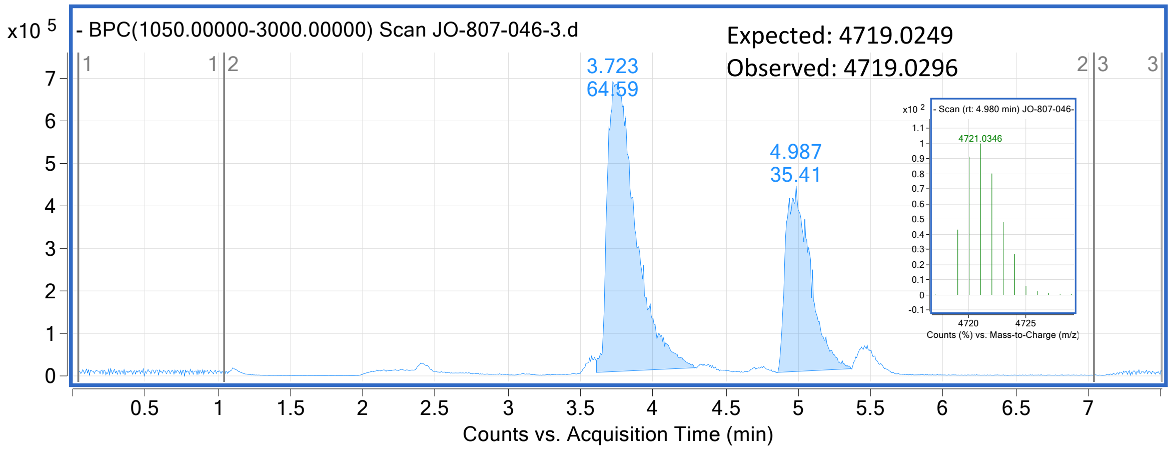

Figure S130: Mass spectrum of DNA-tagged product of reductive amination of HP2 and 3-formylbenzonitrile using TTAC as the surfactant analysed by DNA mass spectrometry gradient A.

Expected: 4719.0249
Observed: 4719.0286

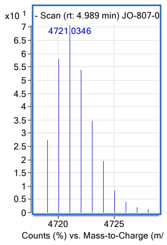

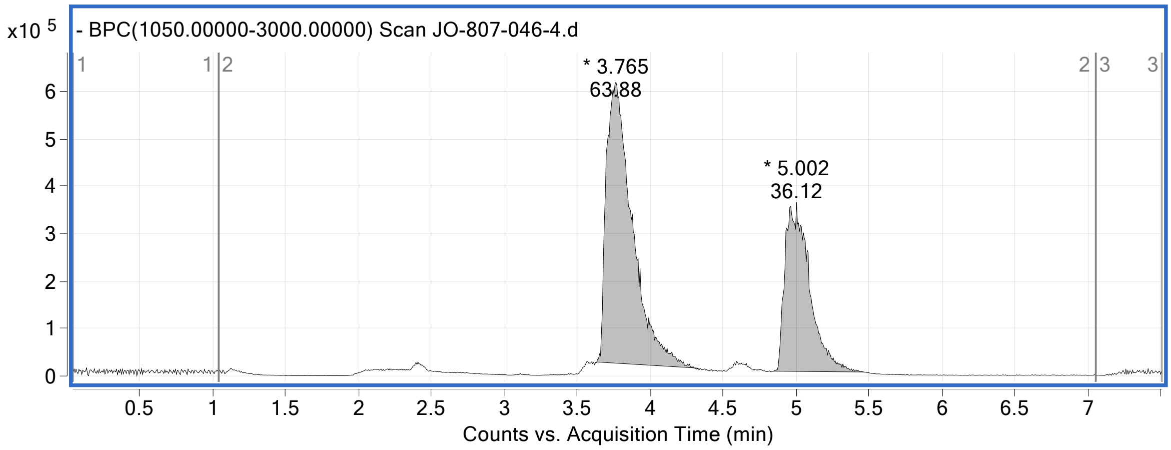

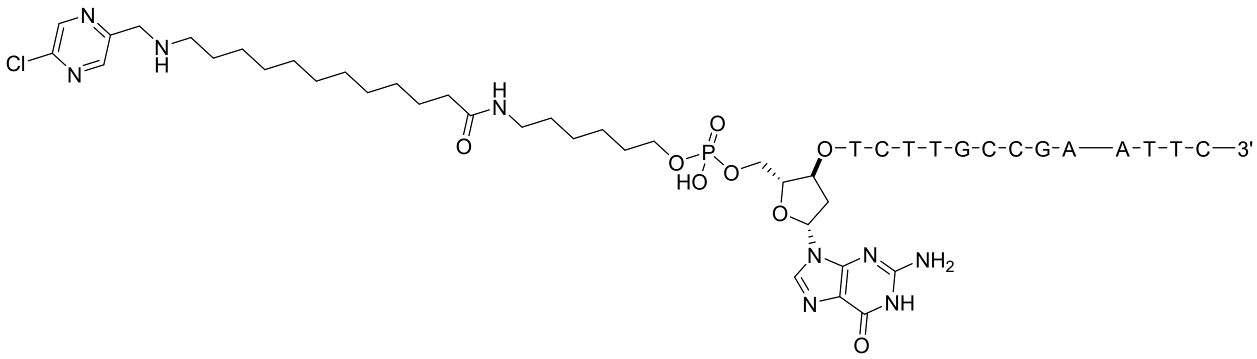

Figure S131: Mass spectrum of DNA-tagged product of reductive amination of HP2 and 5-chloropyrazine-2-carbaldehyde using TPGS-750-M as the surfactant analysed by DNA mass spectrometry gradient A.

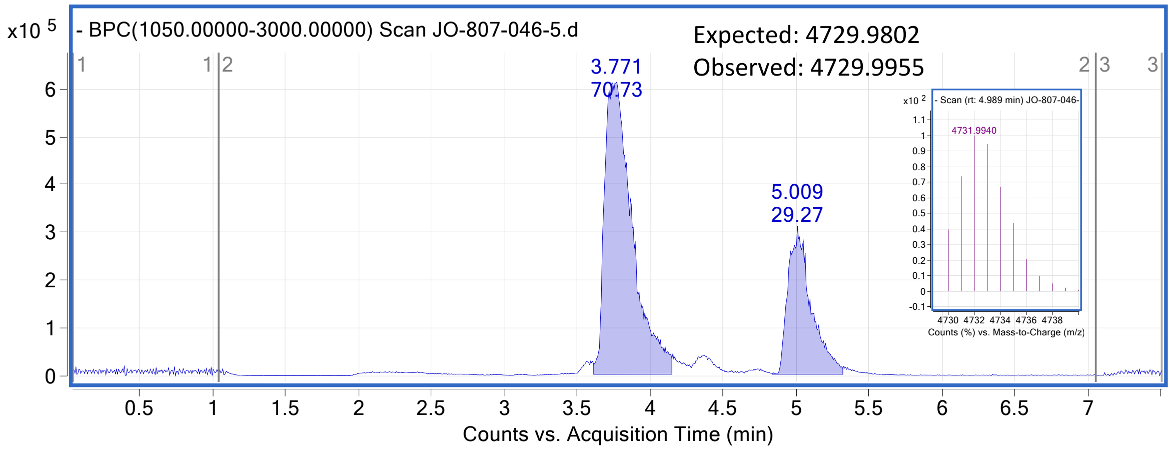

Figure S132: Mass spectrum of DNA-tagged product of reductive amination of HP2 and 5-chloropyrazine-2-carbaldehyde using TTAC as the surfactant analysed by DNA mass spectrometry gradient A.

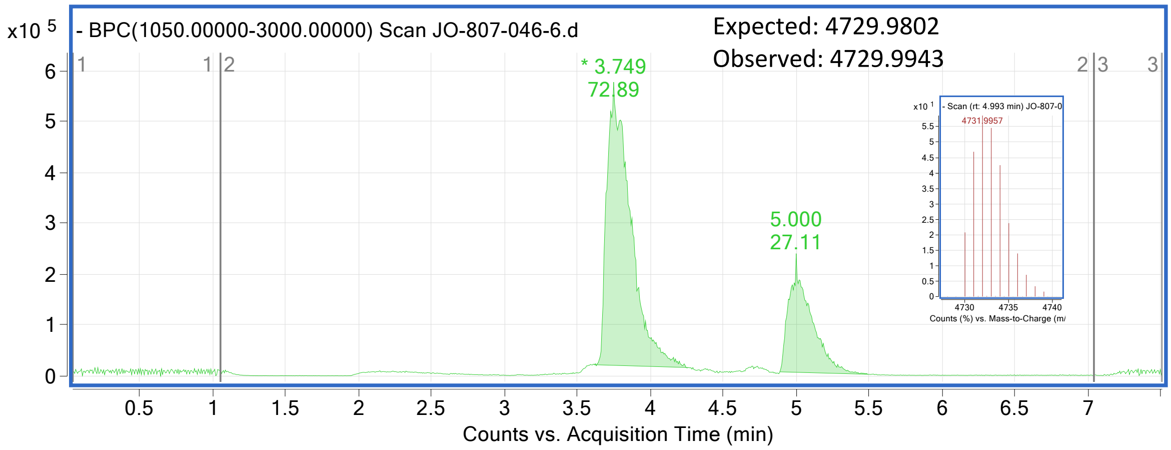

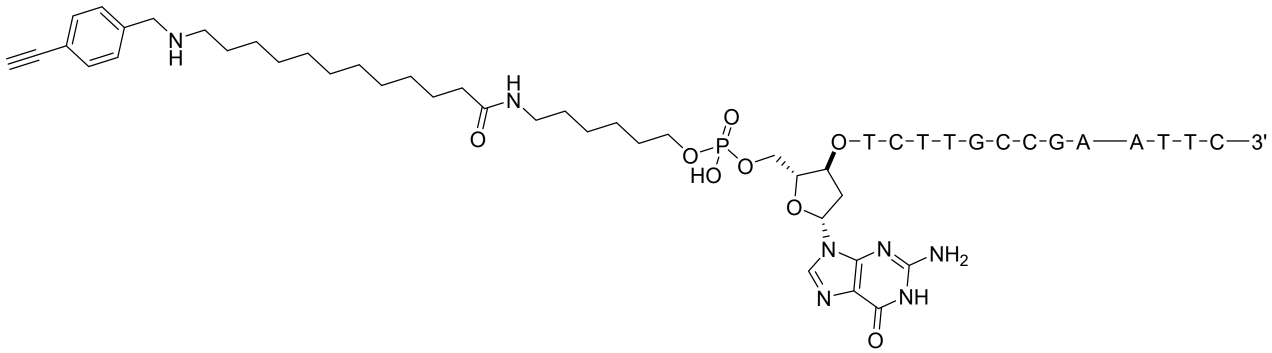

Figure S133: Mass spectrum of DNA-tagged product of reductive amination of HP2 and 4-ethynylbenzaldehyde using TPGS-750-M as the surfactant analysed by DNA mass spectrometry gradient A.

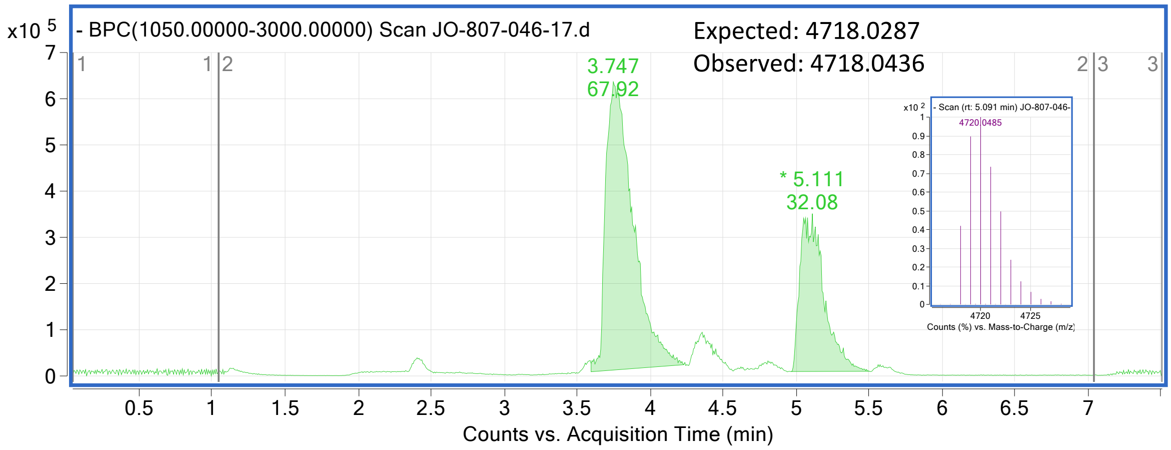

Figure S134: Mass spectrum of DNA-tagged product of reductive amination of HP2 and 4-ethynylbenzaldehyde using TTAC as the surfactant analysed by DNA mass spectrometry gradient A.

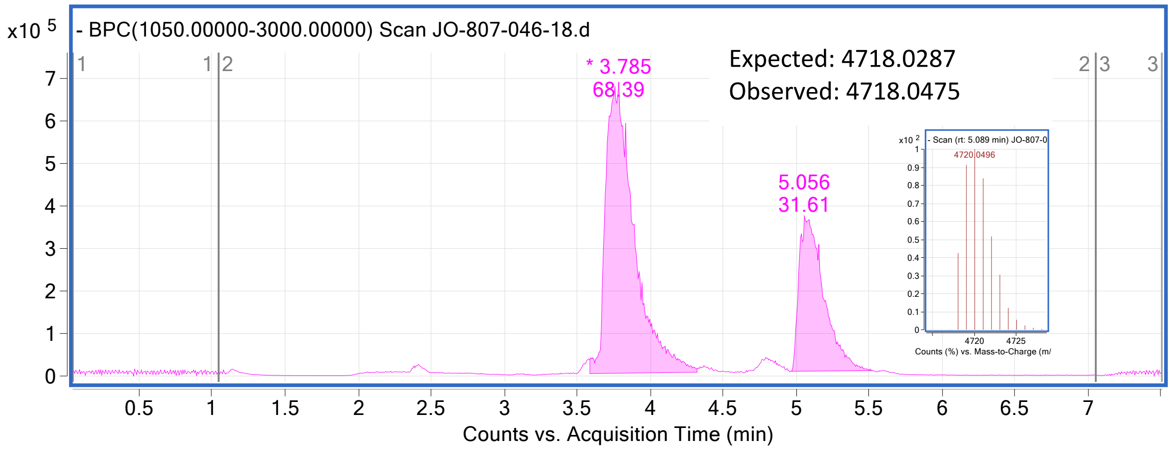

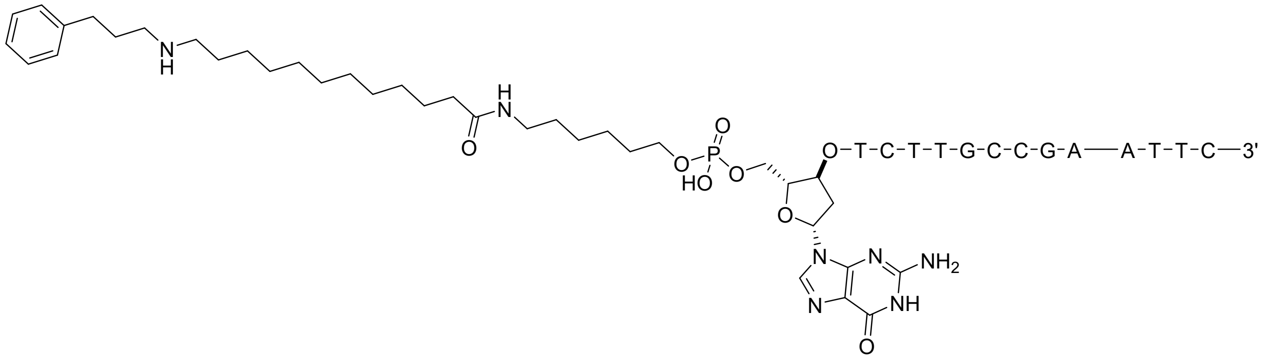

Figure S135: Mass spectrum of DNA-tagged product of reductive amination of HP2 and 3-phenylpropanal using TPGS-750-M as the surfactant analysed by DNA mass spectrometry gradient A.

Expected: 4722.0600
Observed: 4722.1138

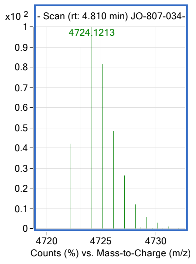

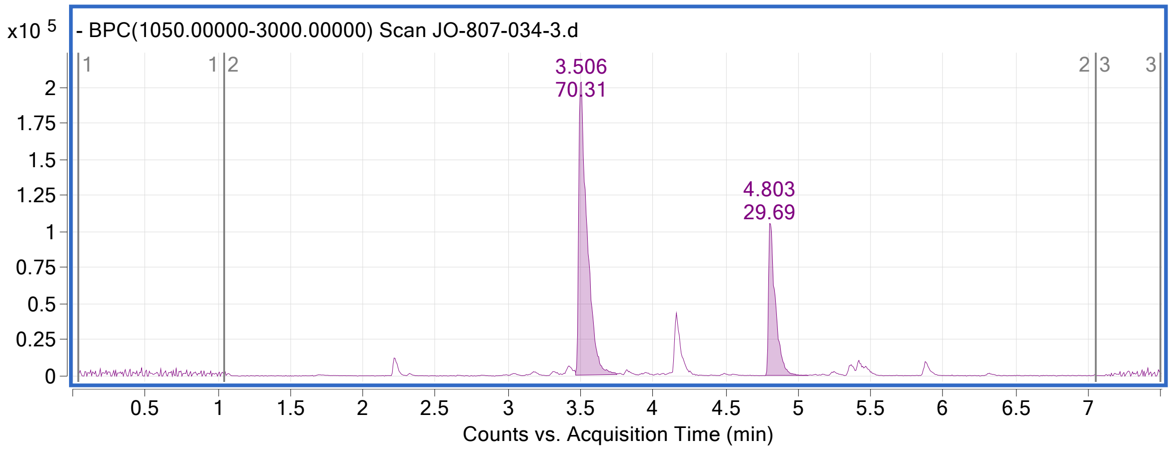

Figure S136: Mass spectrum of DNA-tagged product of reductive amination of HP2 and 3-phenylpropanal using TTAC as the surfactant analysed by DNA mass spectrometry gradient A.

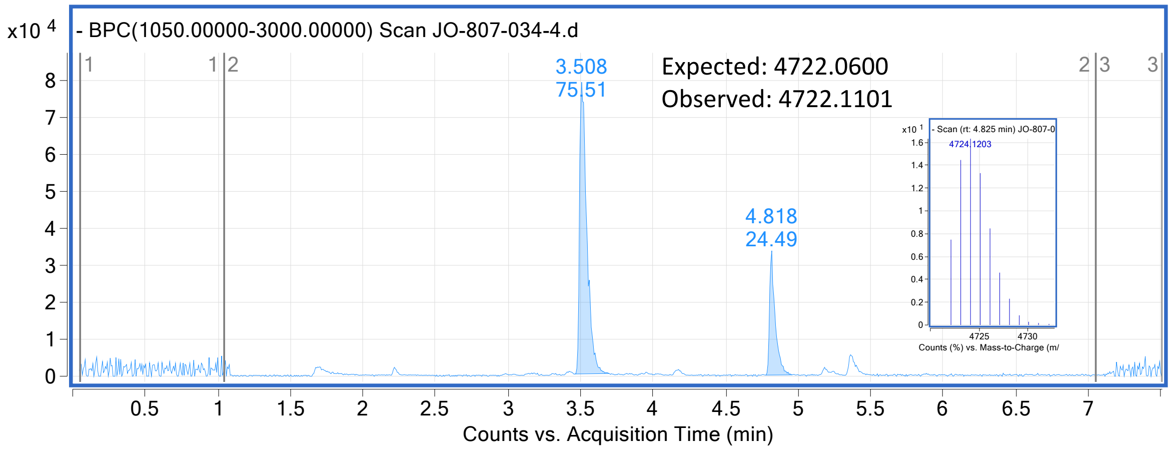

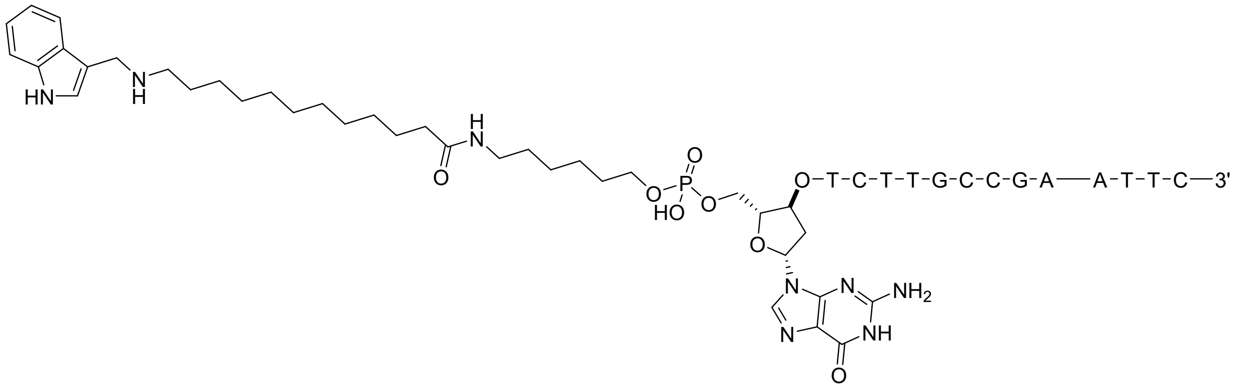

Figure S137: Mass spectrum of DNA-tagged product of reductive amination of HP2 and 1*H*-indole-3-carbaldehyde using TPGS-750-M as the surfactant analysed by DNA mass spectrometry gradient A.

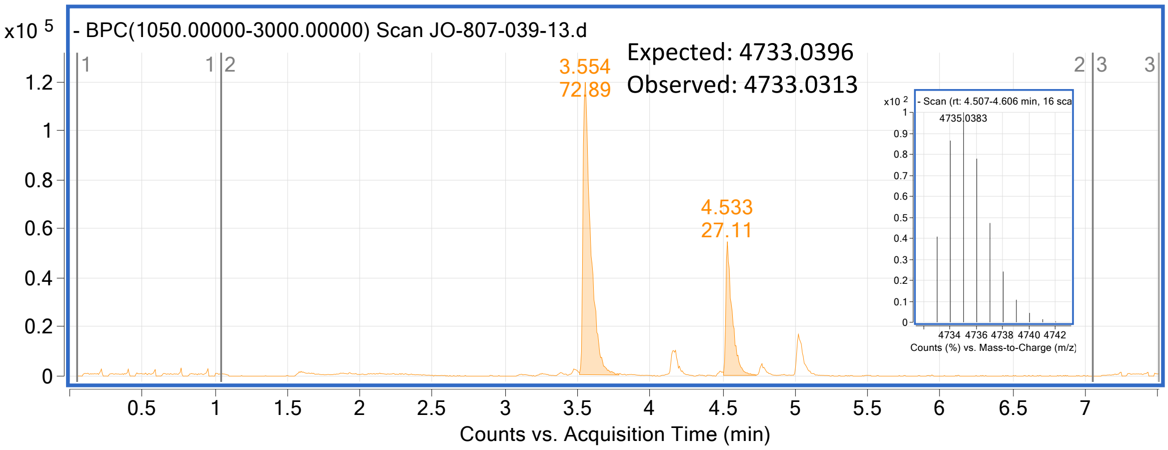

Figure S138: Mass spectrum of DNA-tagged product of reductive amination of HP2 and 1*H*-indole-3-carbaldehyde using TTAC as the surfactant analysed by DNA mass spectrometry gradient A.

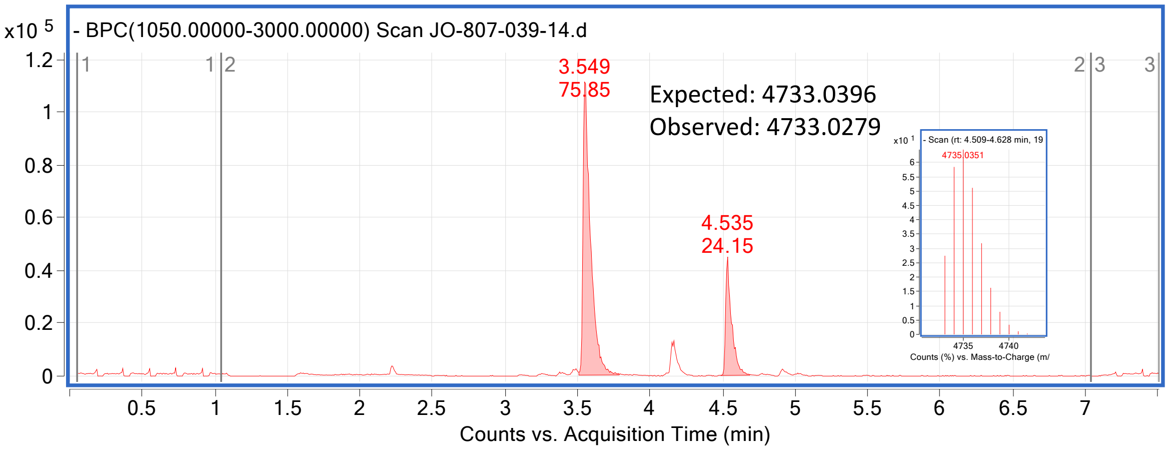

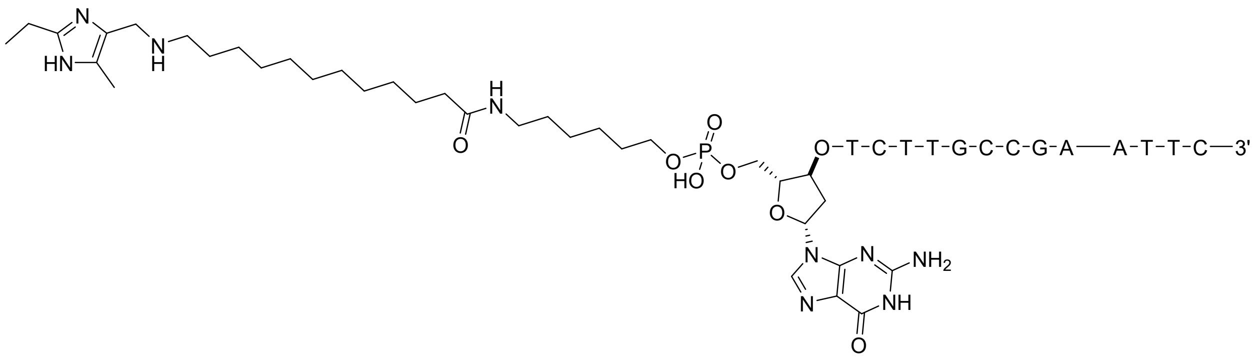

Figure S139: Mass spectrum of DNA-tagged product of reductive amination of HP2 and 2-ethyl-5-methyl-1*H*-imidazole-4-carbaldehyde using TPGS-750-M as the surfactant analysed by DNA mass spectrometry gradient A.

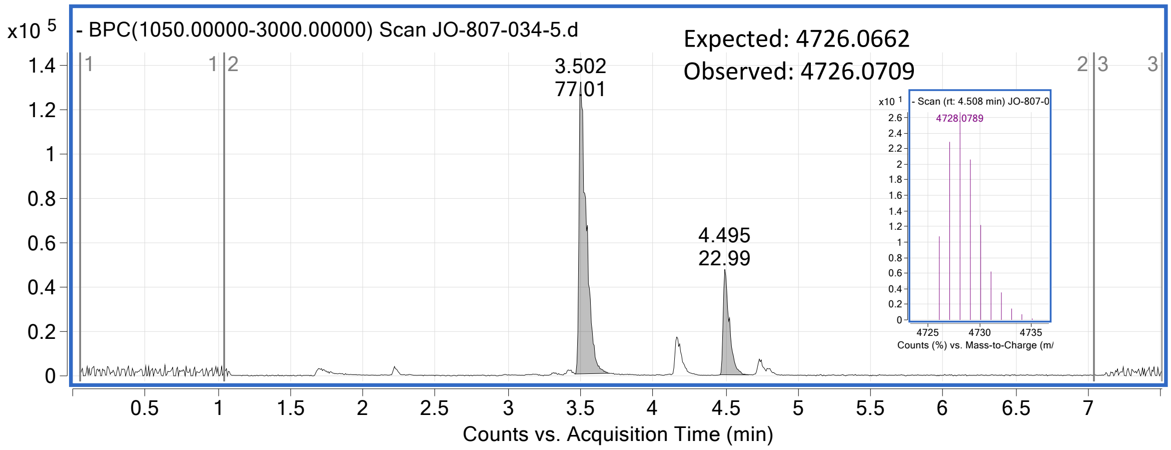

Figure S140: Mass spectrum of DNA-tagged product of reductive amination of HP2 and 2-ethyl-5-methyl-1*H*-imidazole-4-carbaldehyde using TTAC as the surfactant analysed by DNA mass spectrometry gradient A.

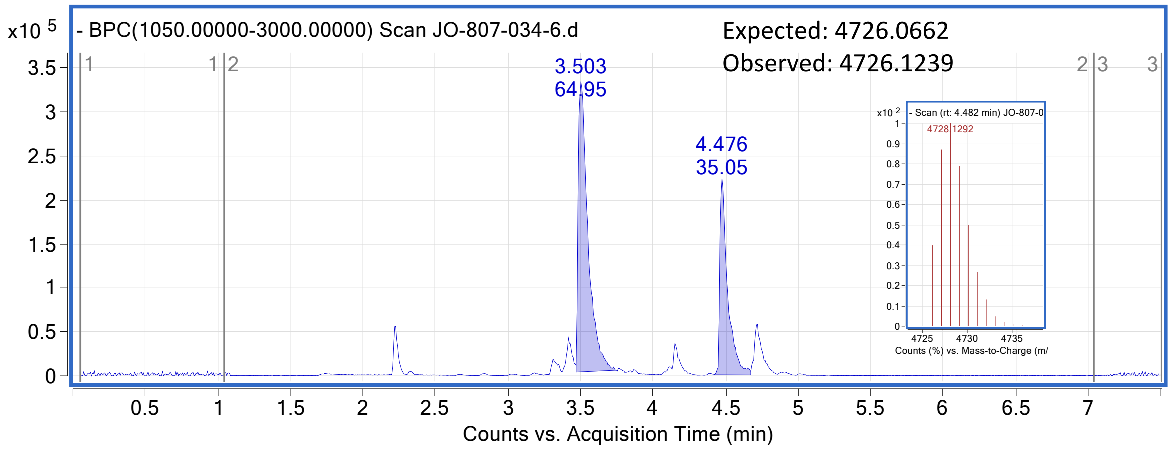

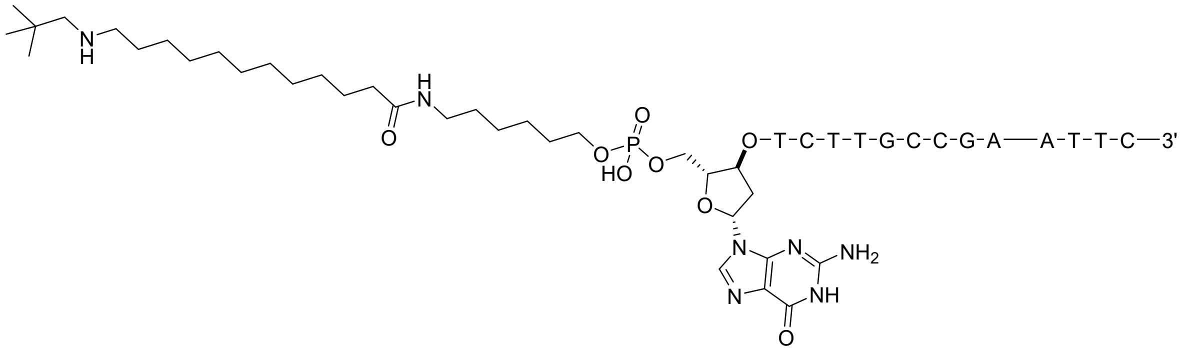

Figure S141: Mass spectrum of DNA-tagged product of reductive amination of HP2 and pivalaldehyde using TPGS-750-M as the surfactant analysed by DNA mass spectrometry gradient A.

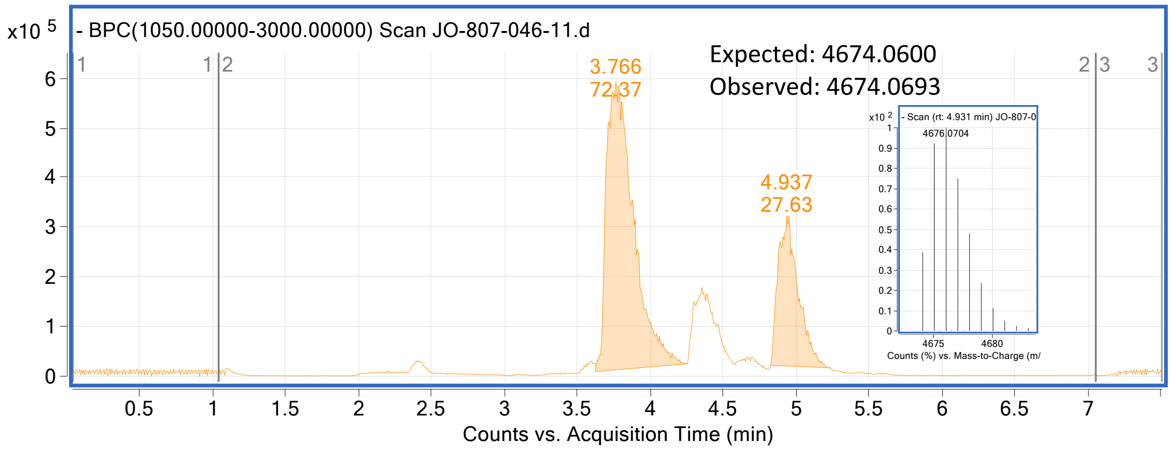

Figure S142: Mass spectrum of DNA-tagged product of reductive amination of HP2 and pivalaldehyde using TTAC as the surfactant analysed by DNA mass spectrometry gradient A.

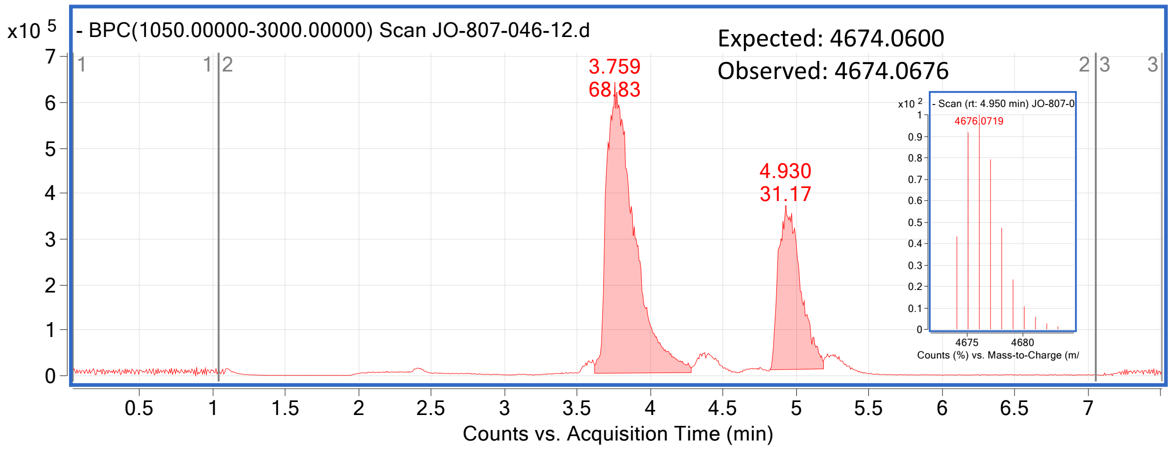

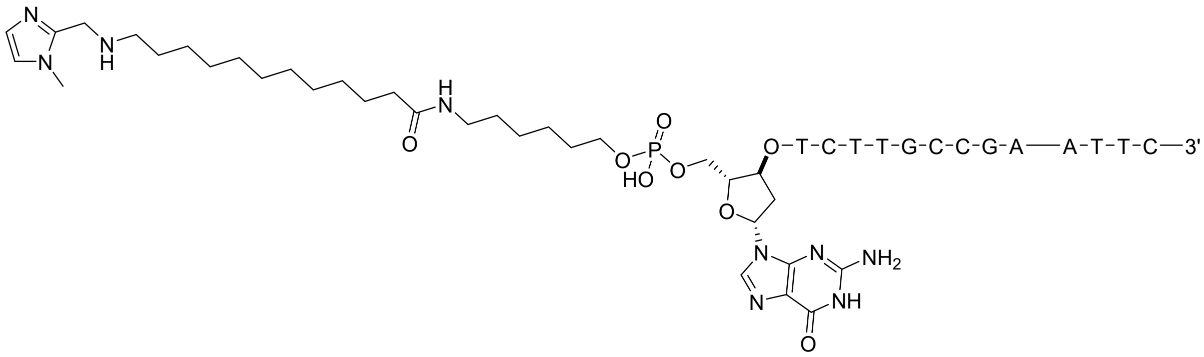

Figure S143: Mass spectrum of DNA-tagged product of reductive amination of HP2 and 1-methyl-1*H*-imidazole-2-carbaldehyde using TPGS-750-M as the surfactant analysed by DNA mass spectrometry gradient A.

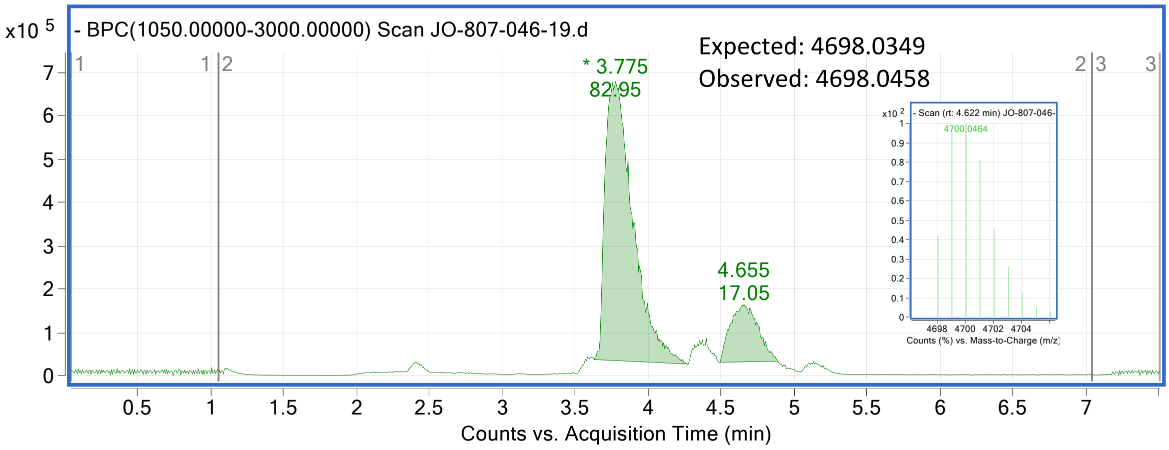

Figure S144: Mass spectrum of DNA-tagged product of reductive amination of HP2 and 1-methyl-1*H*-imidazole-2-carbaldehyde using TTAC as the surfactant analysed by DNA mass spectrometry gradient A.

Expected: 4698.0349
Observed: 4698.0475

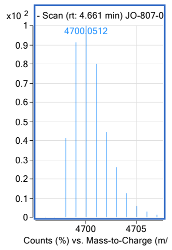

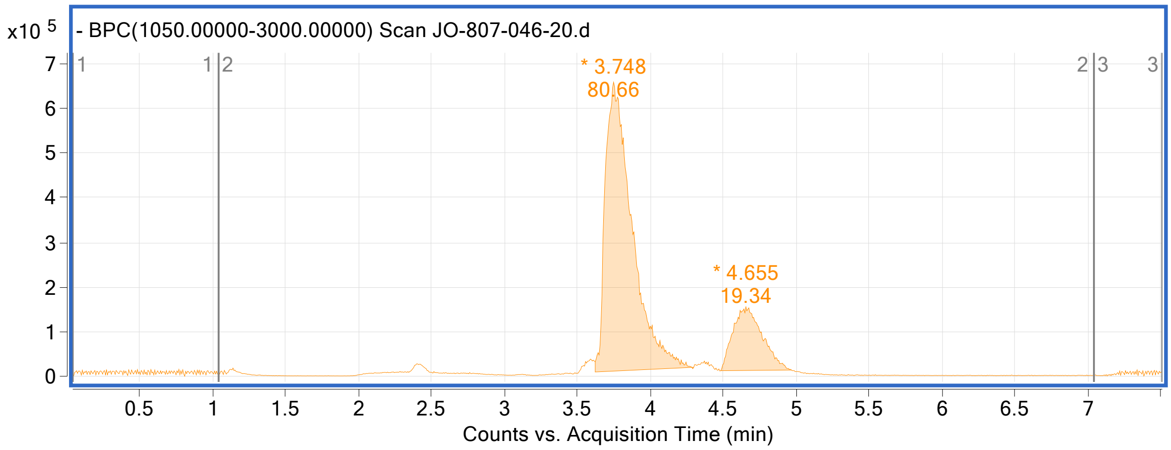

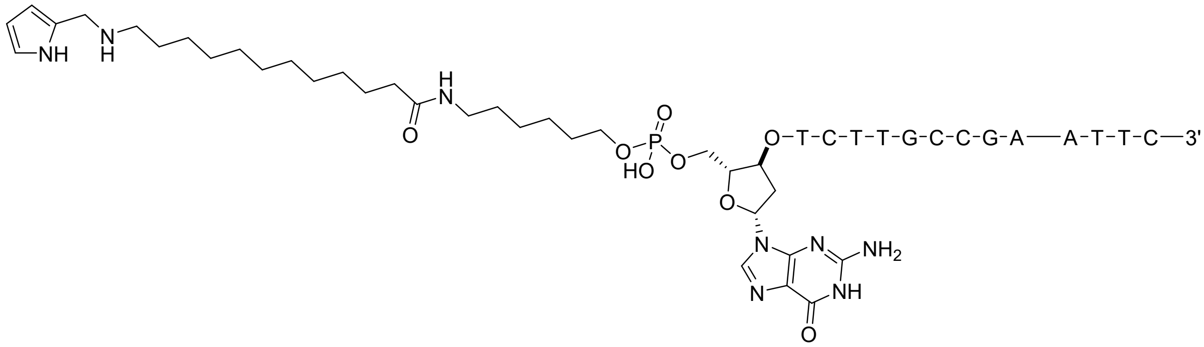

Figure S145: Mass spectrum of DNA-tagged product of reductive amination of HP2 and 1*H*-pyrrole-2-carbaldehyde using TPGS-750-M as the surfactant analysed by DNA mass spectrometry gradient A.

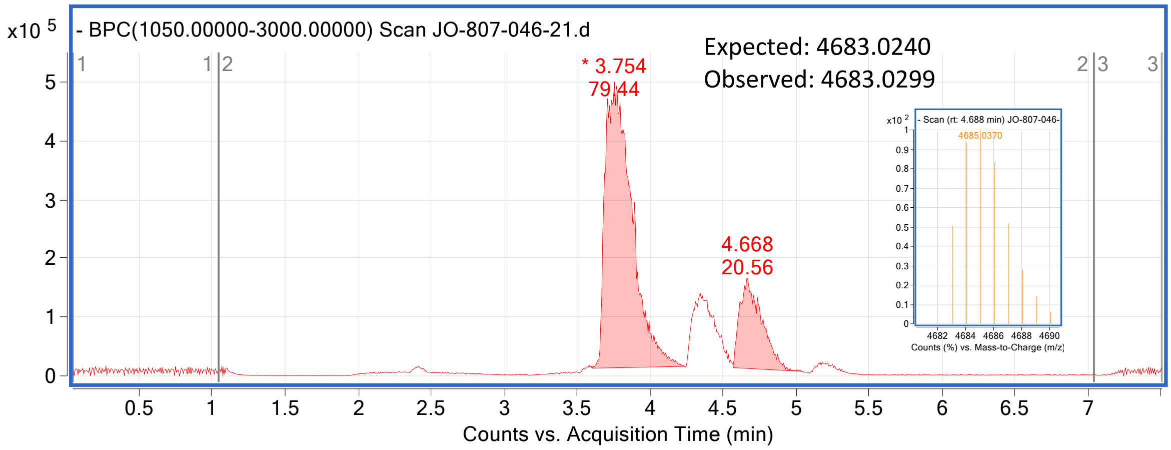

Figure S146: Mass spectrum of DNA-tagged product of reductive amination of HP2 and 1*H*-pyrrole-2-carbaldehyde using TTAC as the surfactant analysed by DNA mass spectrometry gradient A.

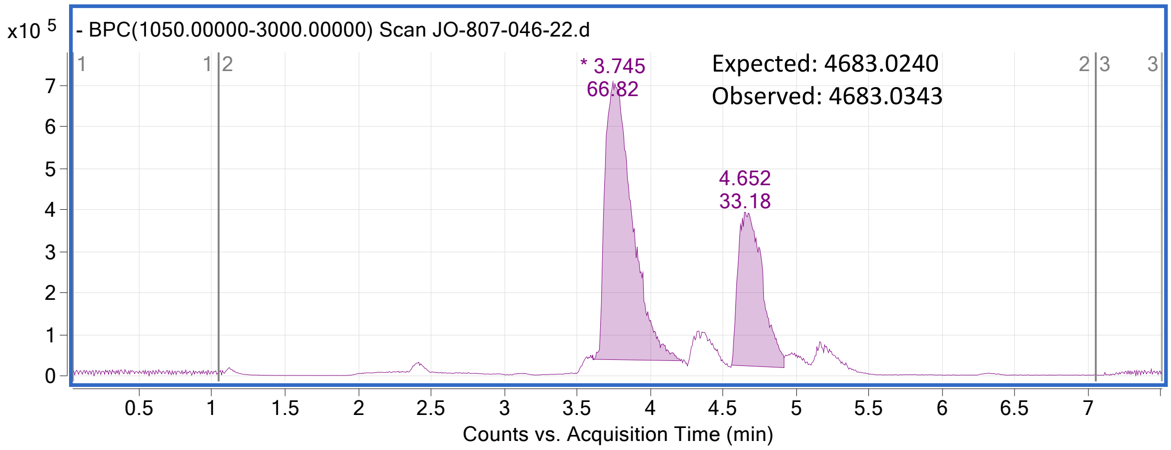

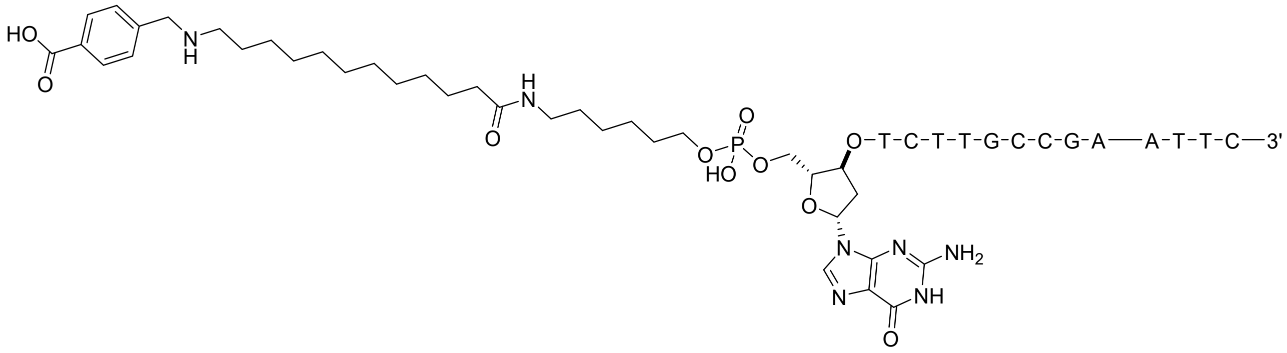

Figure S147: Mass spectrum of DNA-tagged product of reductive amination of HP2 and 4-formylbenzoic acid using TPGS-750-M as the surfactant analysed by DNA mass spectrometry gradient A.

Expected: 4738.0185
Observed: 4738.0723

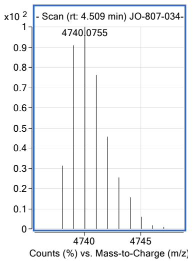

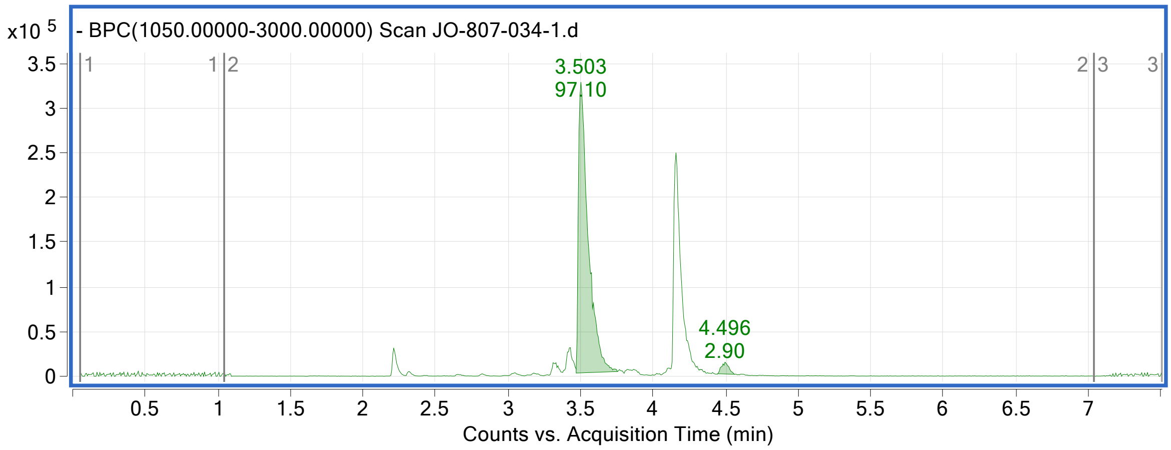

Figure S148: Mass spectrum of DNA-tagged product of reductive amination of HP2 and 4-formylbenzoic acid using TTAC as the surfactant analysed by DNA mass spectrometry gradient A.

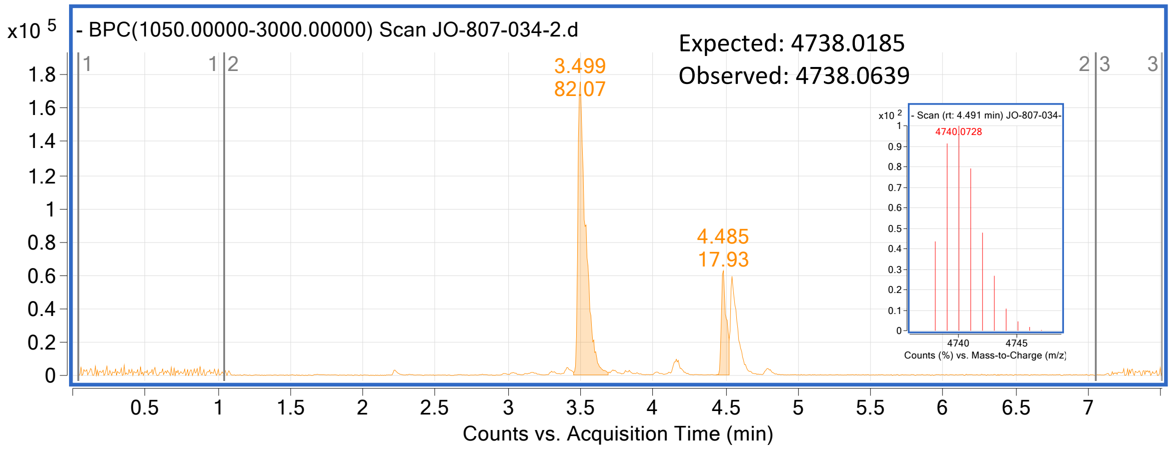

On-DNA Reverse Amide Coupling Reaction of DNA-Conjugated Acid HP3

Table 8/9 Chromatograms

Compound 6 was synthesised according to general reverse amide coupling procedure.

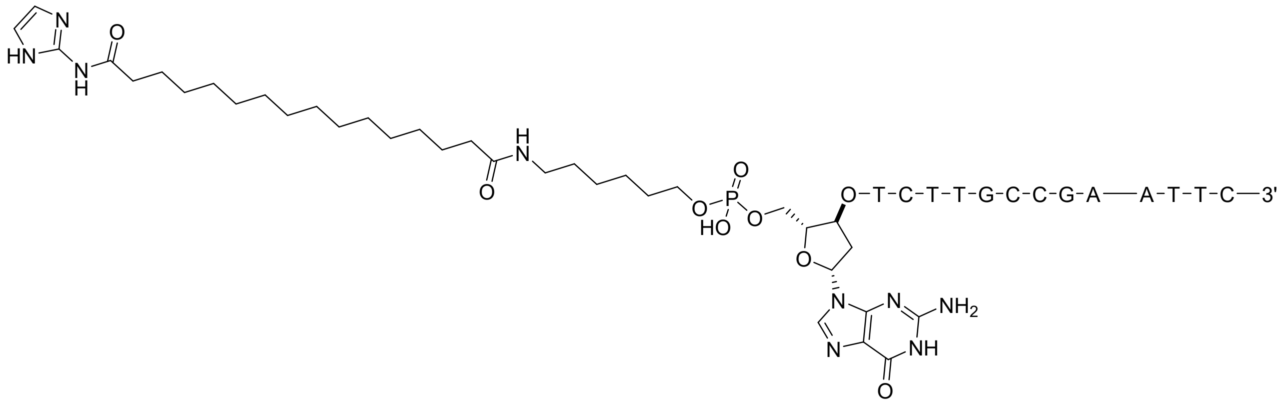

Figure S149: Mass spectrum of DNA-tagged product of reverse amide coupling of HP3 and 1*H*-imidazol-2-amine hemisulfate with no surfactant analysed by DNA mass spectrometry gradient C.

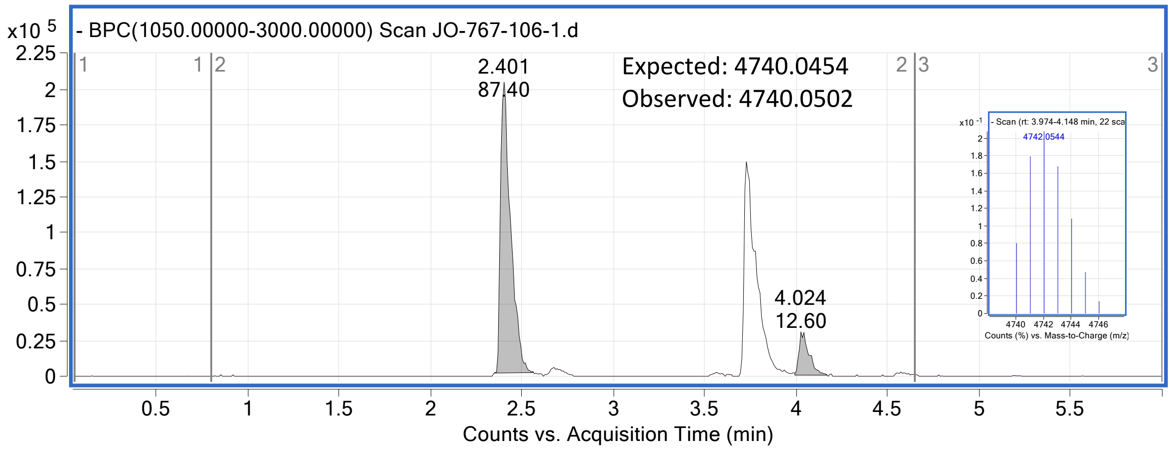

Figure S150: Mass spectrum of DNA-tagged product of reverse amide coupling of HP3 and 1*H*-imidazol-2-amine hemisulfate using TPGS-750-M as the surfactant analysed by DNA mass spectrometry gradient C.

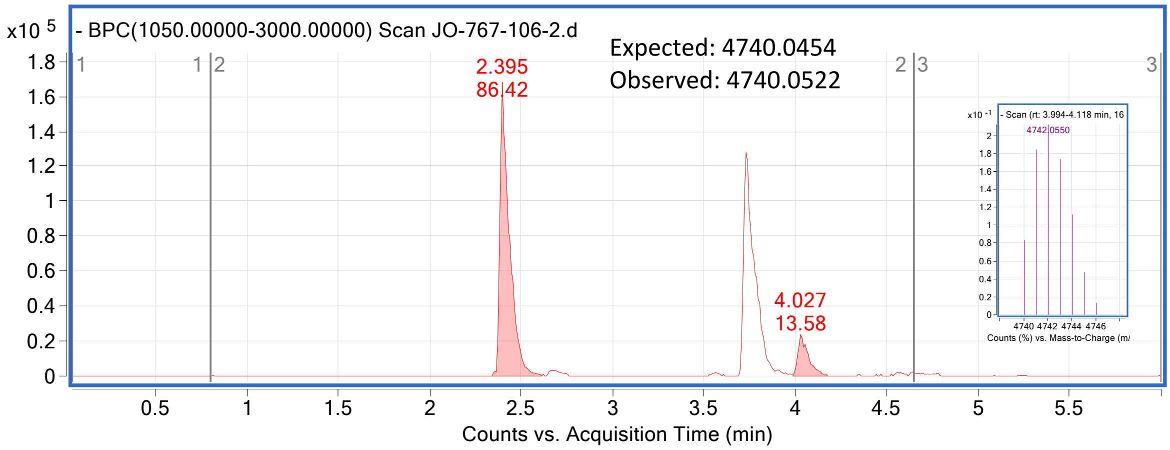

Figure S151: Mass spectrum of DNA-tagged product of reverse amide coupling of HP3 and 1*H*-imidazol-2-amine hemisulfate using Tween65 as the surfactant analysed by DNA mass spectrometry gradient C.

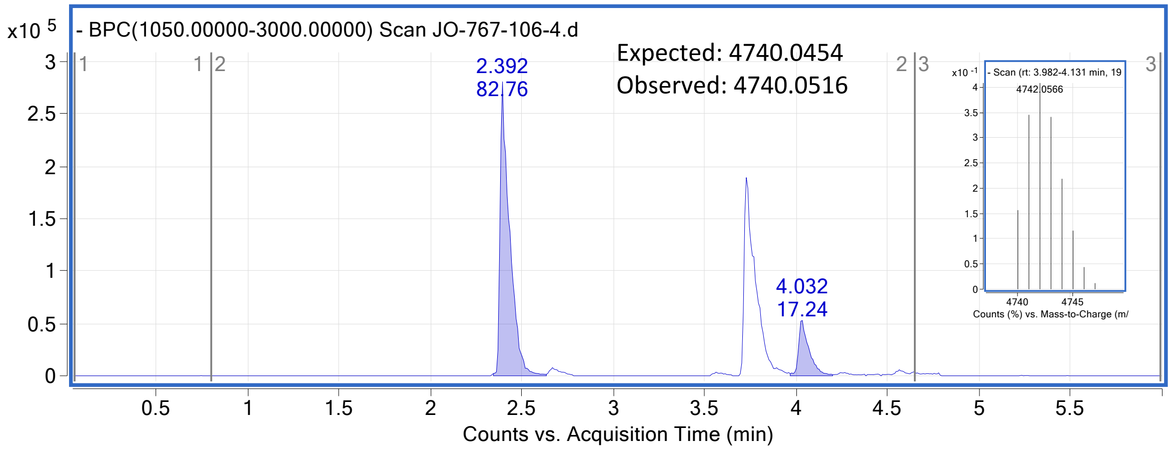

Figure S152: Mass spectrum of DNA-tagged product of reverse amide coupling of HP3 and 1*H*-imidazol-2-amine hemisulfate using Brij700 as the surfactant analysed by DNA mass spectrometry gradient C.

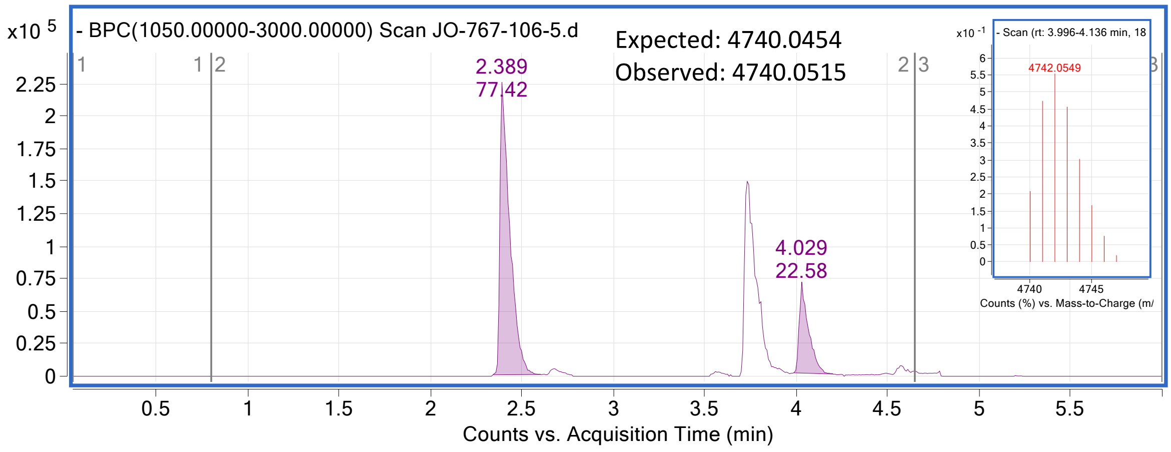

Figure S153: Mass spectrum of DNA-tagged product of reverse amide coupling of HP3 and 1*H*-imidazol-2-amine hemisulfate using BrijS20 as the surfactant analysed by DNA mass spectrometry gradient C.

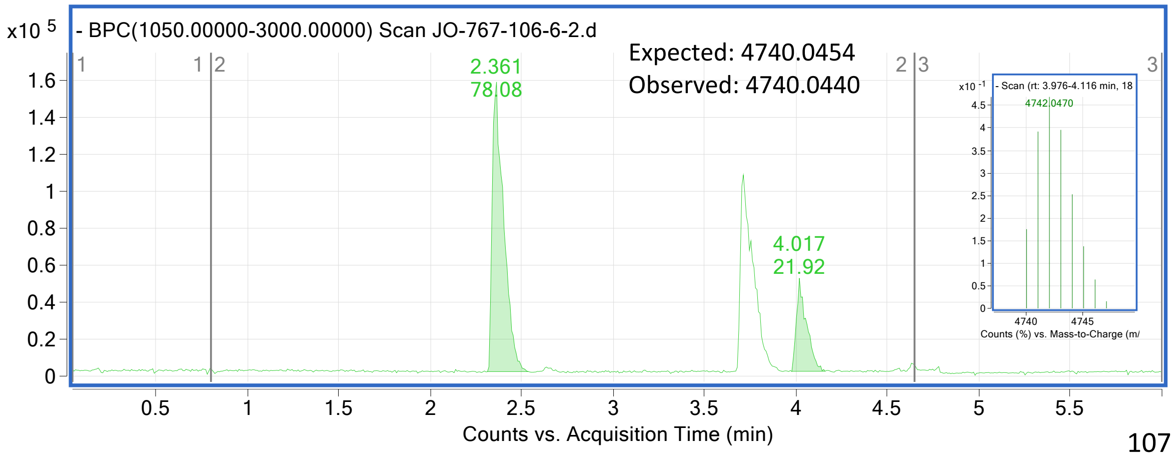

Figure S154: Mass spectrum of DNA-tagged product of reverse amide coupling of HP3 and 1*H*-imidazol-2-amine hemisulfate using Triton-X-405 as the surfactant analysed by DNA mass spectrometry gradient C.

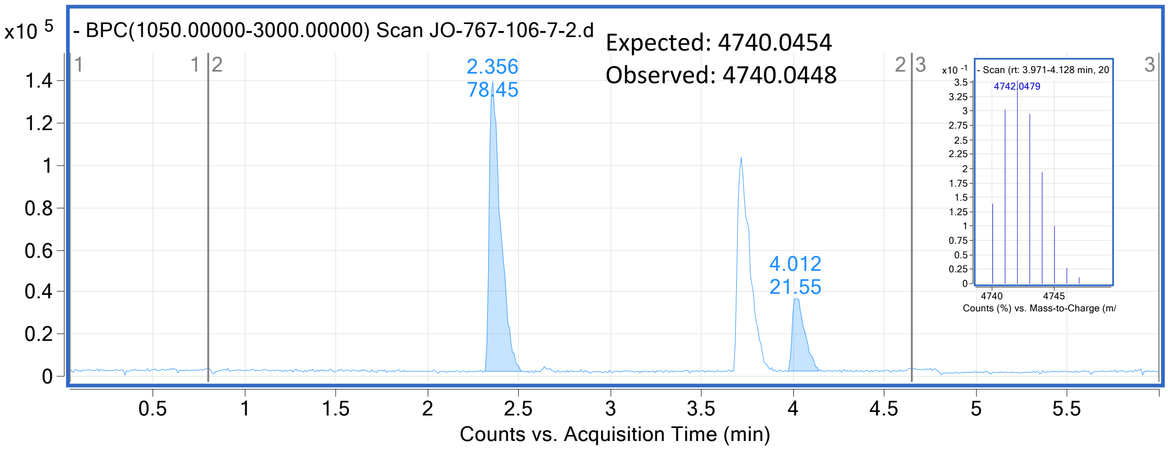

Figure S155: Mass spectrum of DNA-tagged product of reverse amide coupling of HP3 and 1*H*-imidazol-2-amine hemisulfate using TTAC as the surfactant analysed by DNA mass spectrometry gradient C.

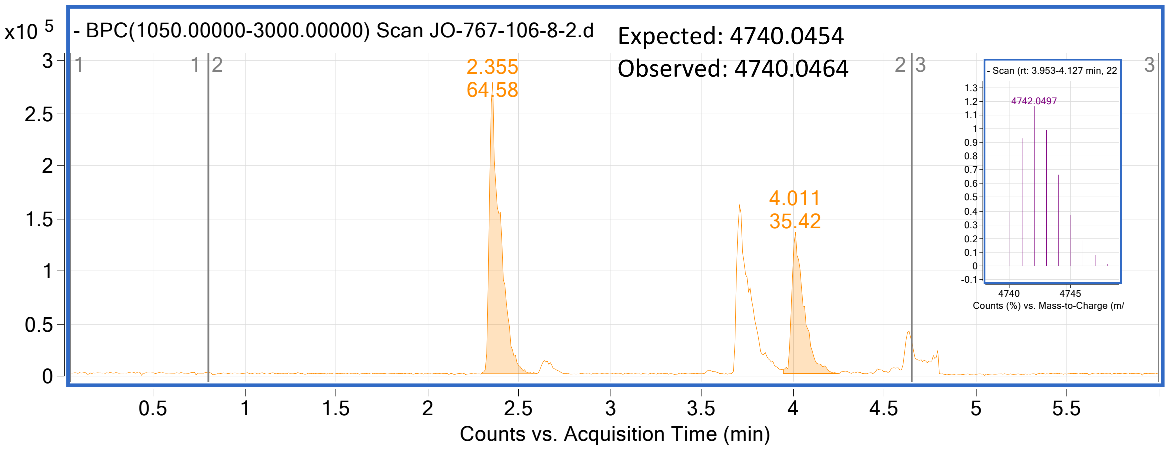

Figure S156: Mass spectrum of DNA-tagged product of reverse amide coupling of HP3 and 1*H*-imidazol-2-amine hemisulfate using Sulfobetaine-16 as the surfactant analysed by DNA mass spectrometry gradient C.

Expected: 4740.0454
Observed: 4740.0504

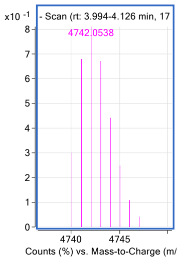

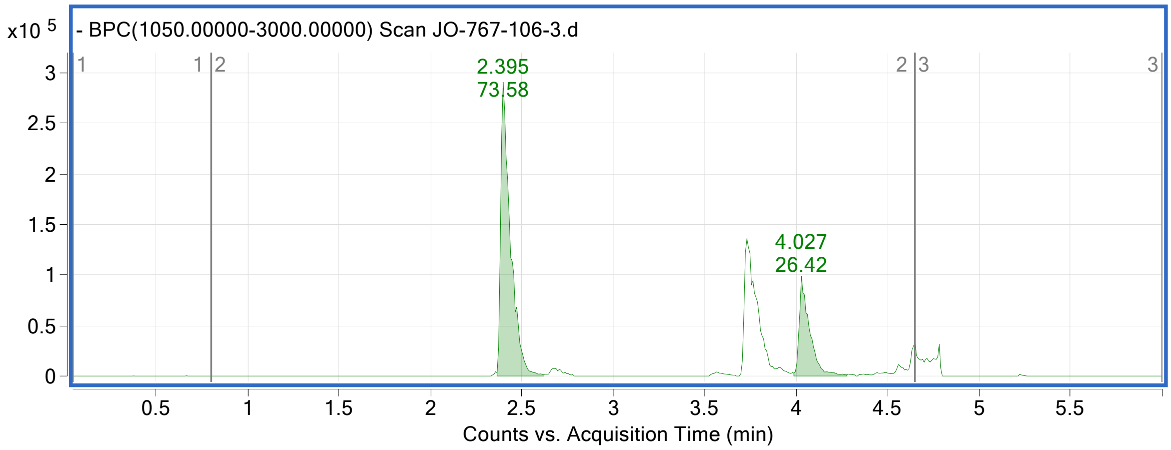

Figure S157: Mass spectrum of DNA-tagged product of reverse amide coupling of HP3 and 1*H*-imidazol-2-amine hemisulfate using Tween85 as the surfactant analysed by DNA mass spectrometry gradient A.

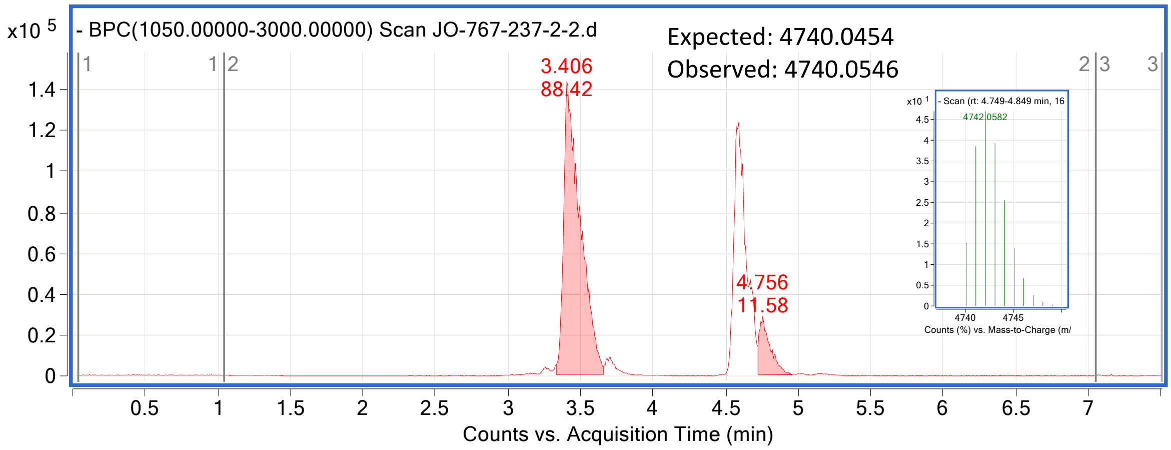

Figure S158: Mass spectrum of DNA-tagged product of reverse amide coupling of HP3 and 1*H*-imidazol-2-amine hemisulfate using Citrol 4DS as the surfactant analysed by DNA mass spectrometry gradient A.

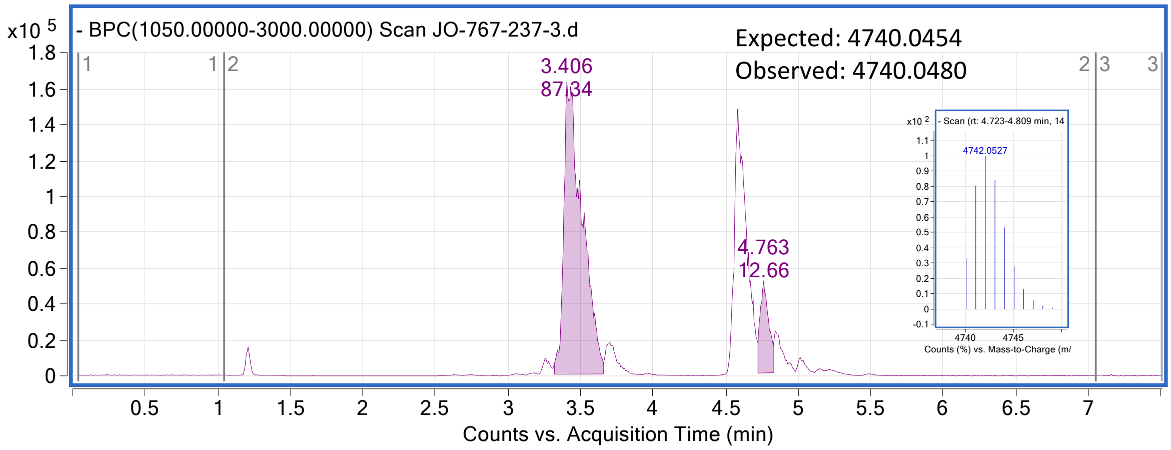

Figure S159: Mass spectrum of DNA-tagged product of reverse amide coupling of HP3 and 1*H*-imidazol-2-amine hemisulfate using Span65 as the surfactant analysed by DNA mass spectrometry gradient A.

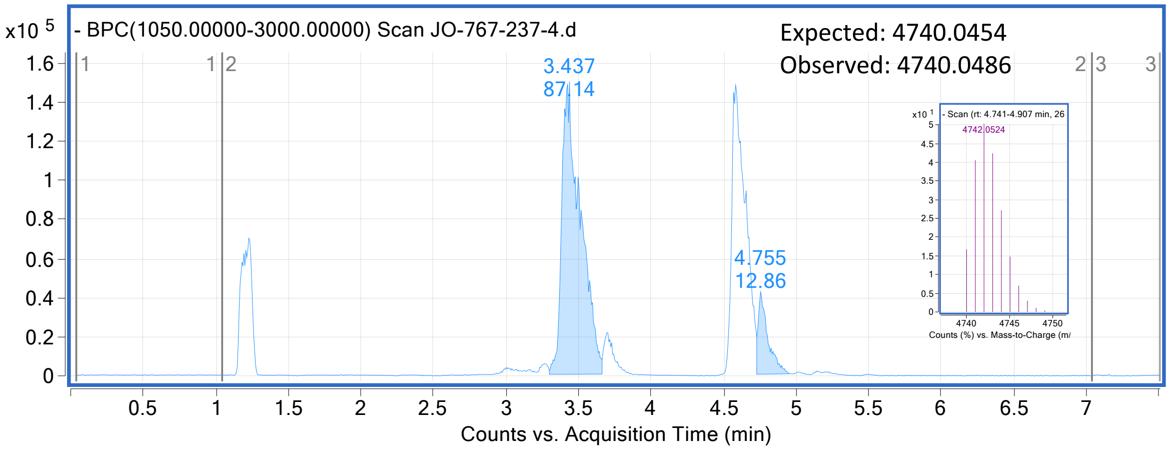

Figure S160: Mass spectrum of DNA-tagged product of reverse amide coupling of HP3 and 1*H*-imidazol-2-amine hemisulfate using Span85 as the surfactant analysed by DNA mass spectrometry gradient A.

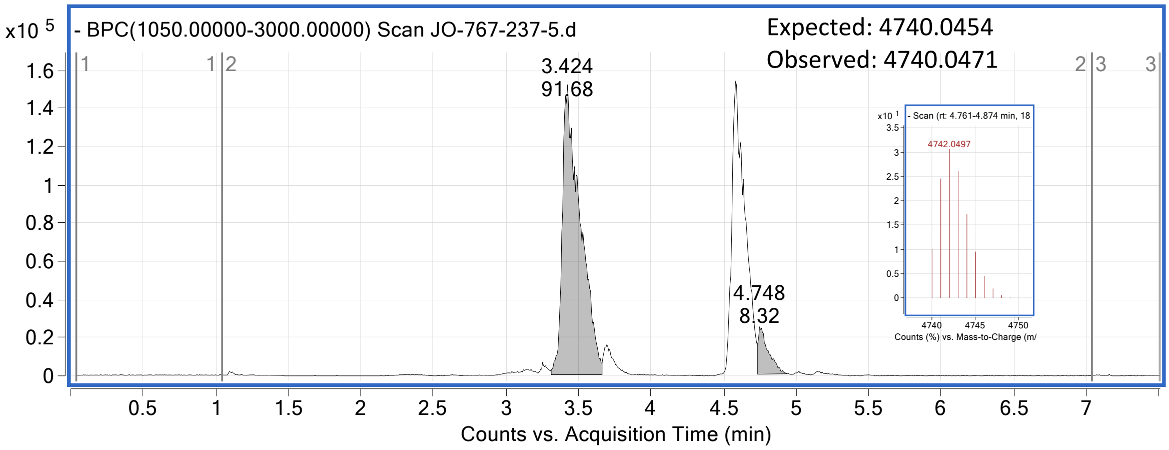

Table 10 Chromatograms

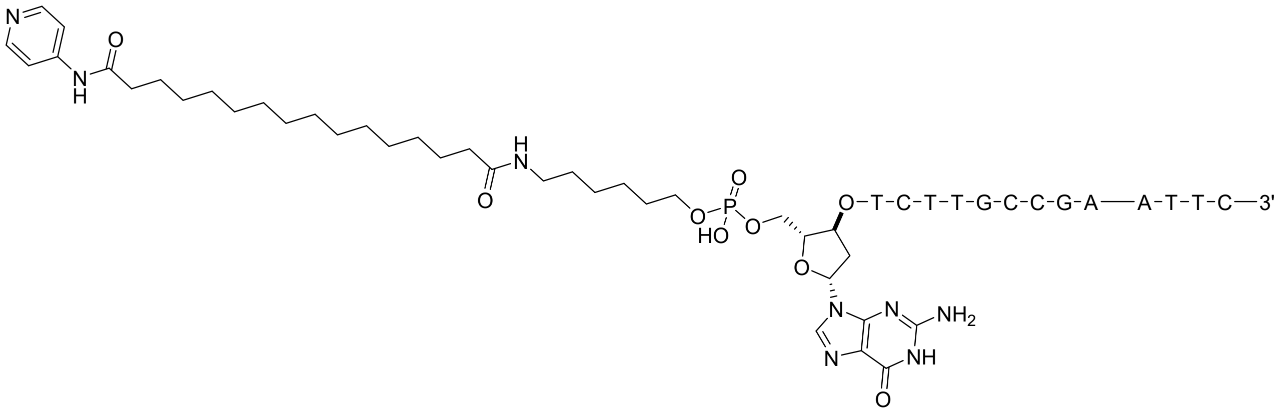

Figure S161: Mass spectrum of DNA-tagged product of reverse amide coupling of HP3 and pyridin-4-amine using TPGS-750-M as the surfactant analysed by DNA mass spectrometry gradient A.

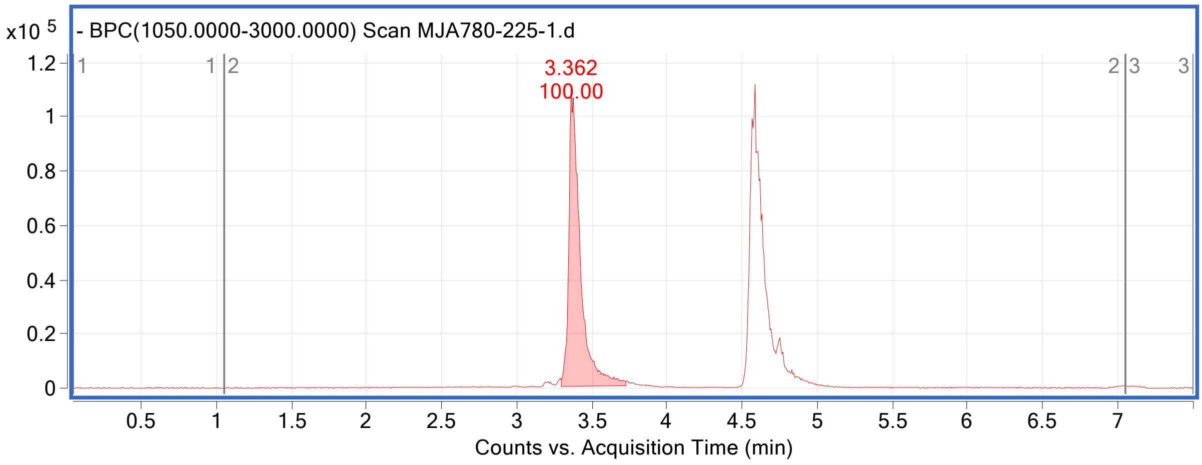

Figure S162: Mass spectrum of DNA-tagged product of reverse amide coupling of HP3 and pyridin-4-amine using TTAC as the surfactant analysed by DNA mass spectrometry gradient A.

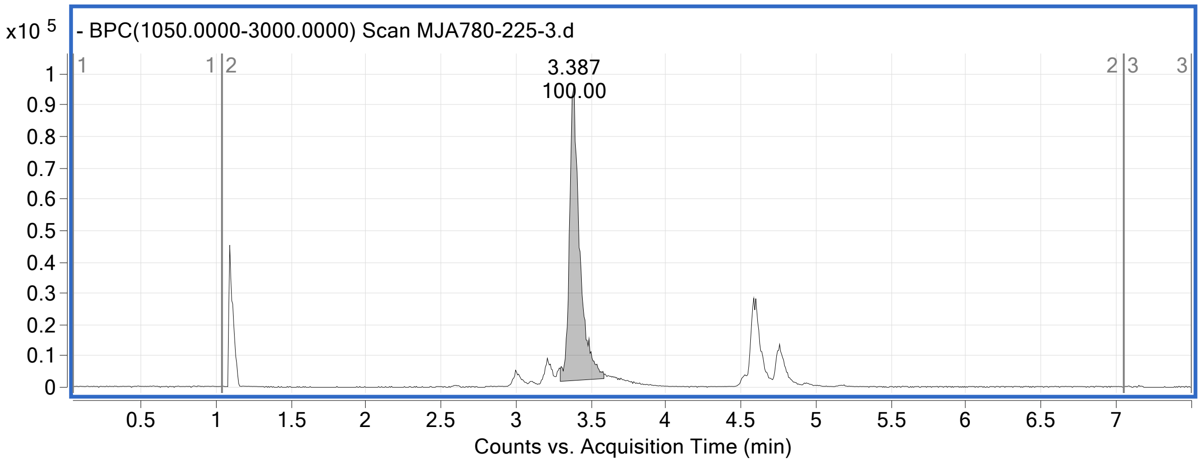

Figure S163: Mass spectrum of DNA-tagged product of reverse amide coupling of HP3 and pyridin-4-amine using Sulfobetaine-16 as the surfactant analysed by DNA mass spectrometry gradient A.

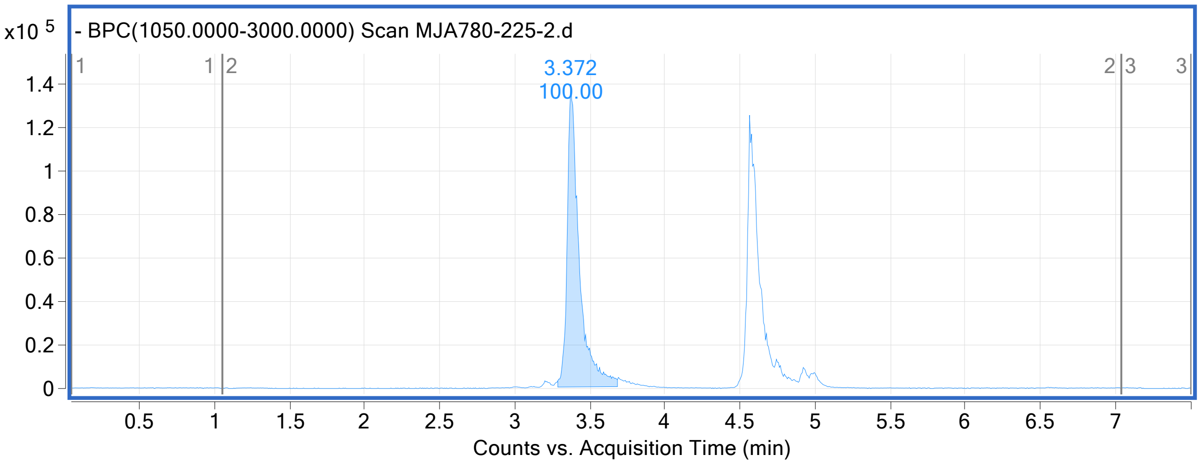

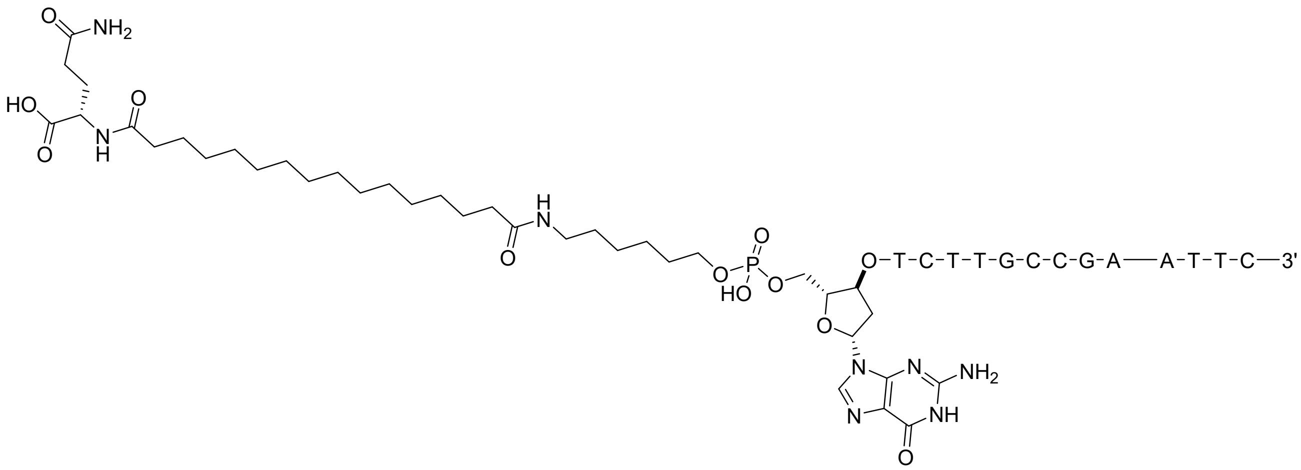

Figure S164: Mass spectrum of DNA-tagged product of reverse amide coupling of HP3 and *l*-glutamine using TPGS-750-M as the surfactant analysed by DNA mass spectrometry gradient A.

Expected: 4803.0662
Observed: 4803.0653

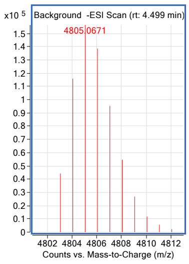

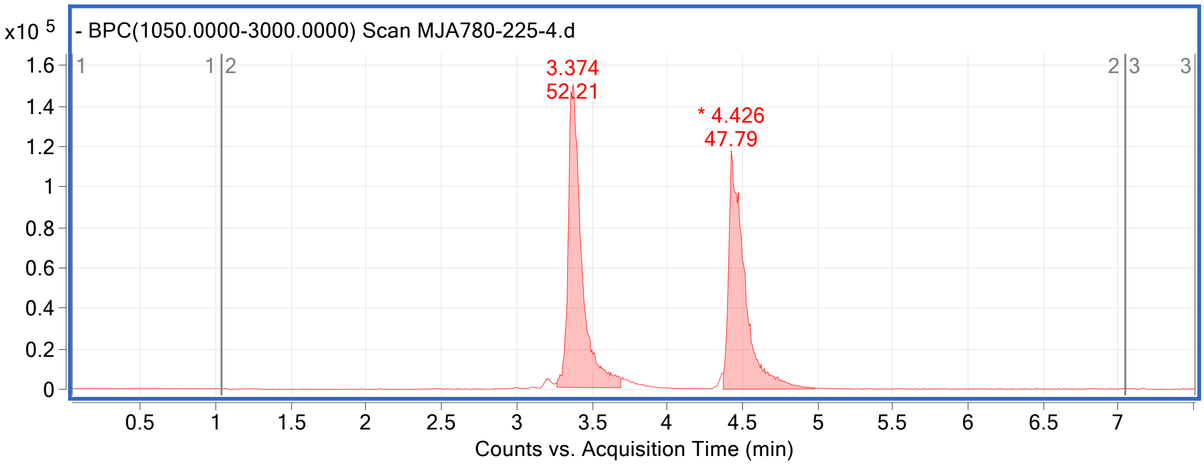

Figure S165: Mass spectrum of DNA-tagged product of reverse amide coupling of HP3 and *l*-glutamine using TTAC as the surfactant analysed by DNA mass spectrometry gradient A.

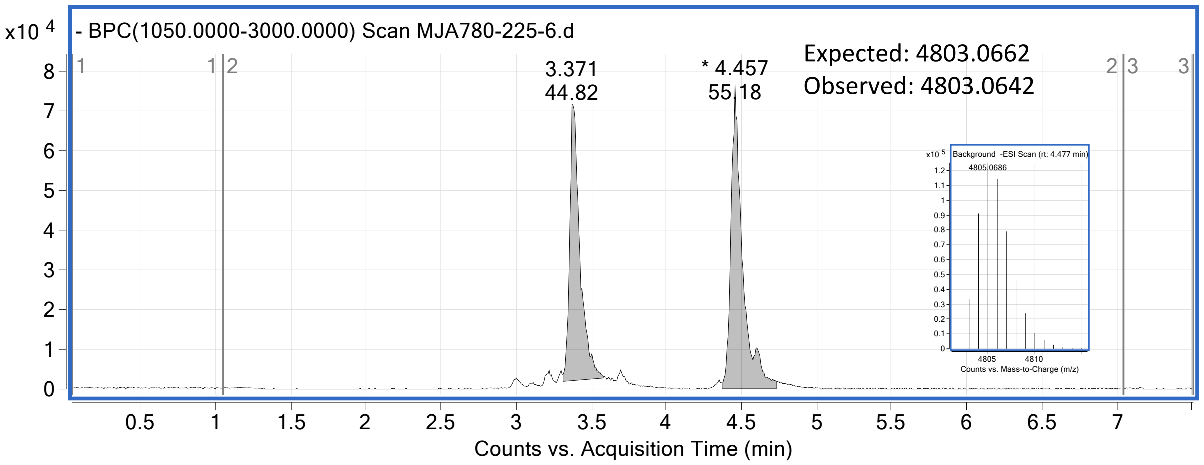

Figure S166: Mass spectrum of DNA-tagged product of reverse amide coupling of HP3 and *l*-glutamine using Sulfobetaine-16 as the surfactant analysed by DNA mass spectrometry gradient A.

Expected: 4803.0662
Observed: 4803.0647

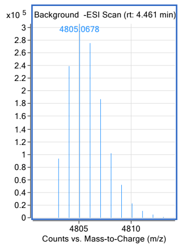

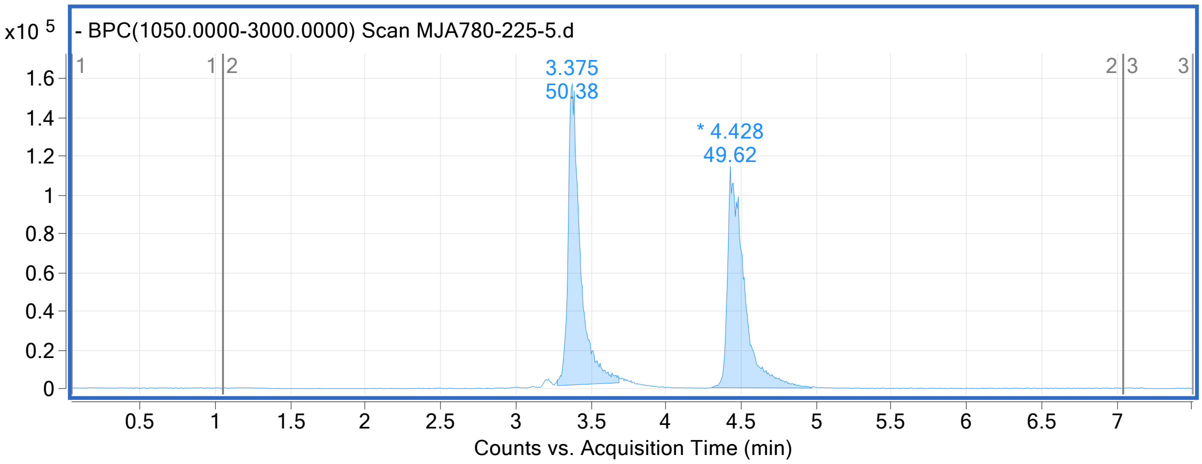

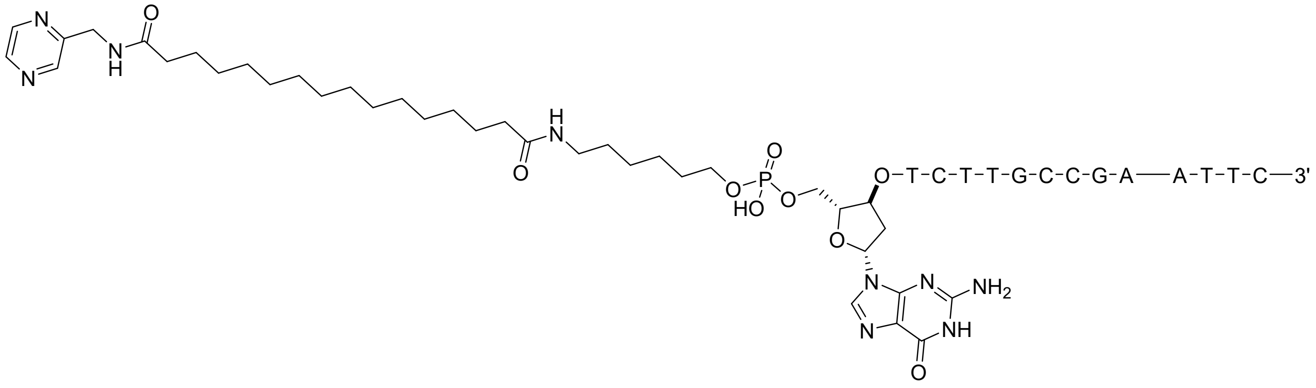

Figure S167: Mass spectrum of DNA-tagged product of reverse amide coupling of HP3 and pyrazin-2-ylmethanamine using TPGS-750-M as the surfactant analysed by DNA mass spectrometry gradient A.

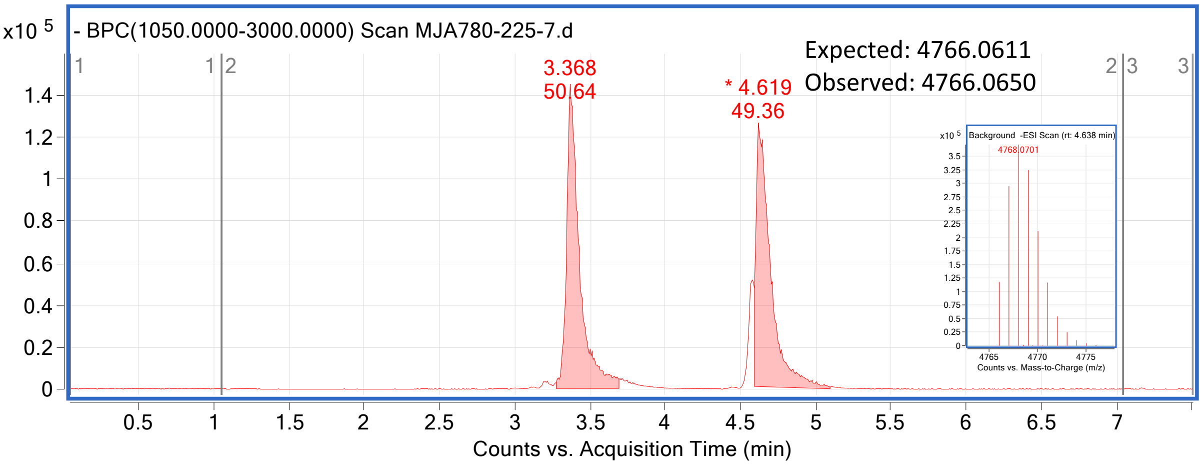

Figure S168: Mass spectrum of DNA-tagged product of reverse amide coupling of HP3 and pyrazin-2-ylmethanamine using TTAC as the surfactant analysed by DNA mass spectrometry gradient A.

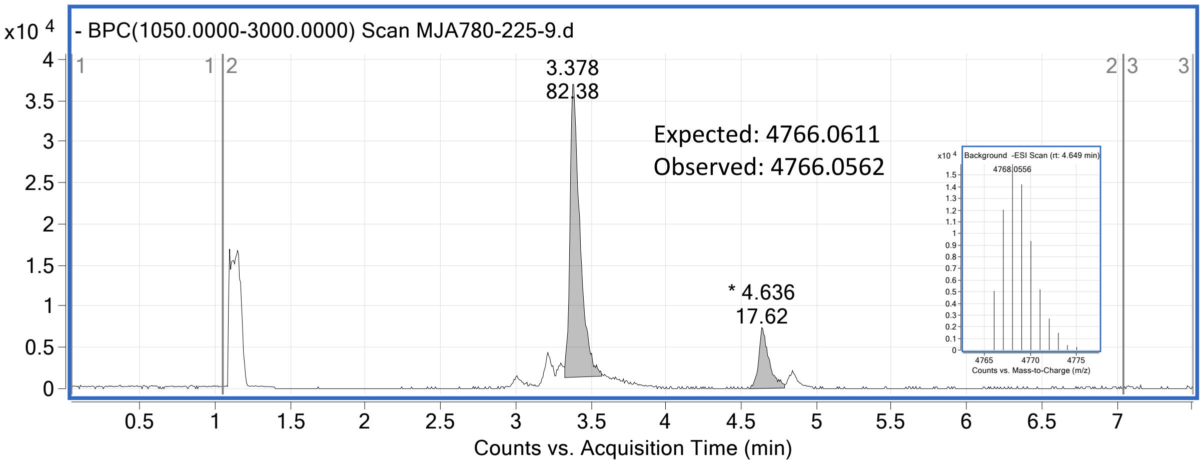

Figure S169: Mass spectrum of DNA-tagged product of reverse amide coupling of HP3 and pyrazin-2-ylmethanamine using Sulfobetaine-16 as the surfactant analysed by DNA mass spectrometry gradient A.

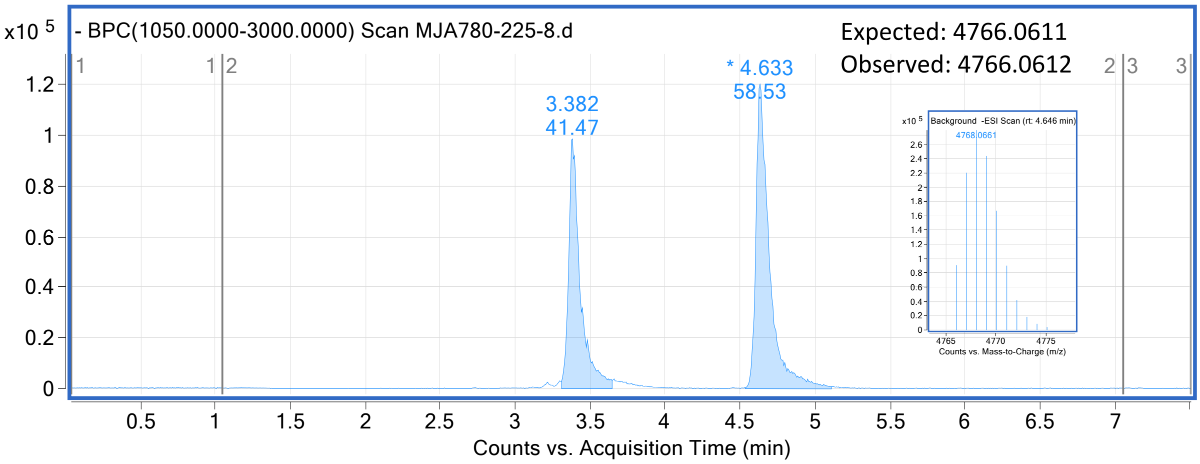

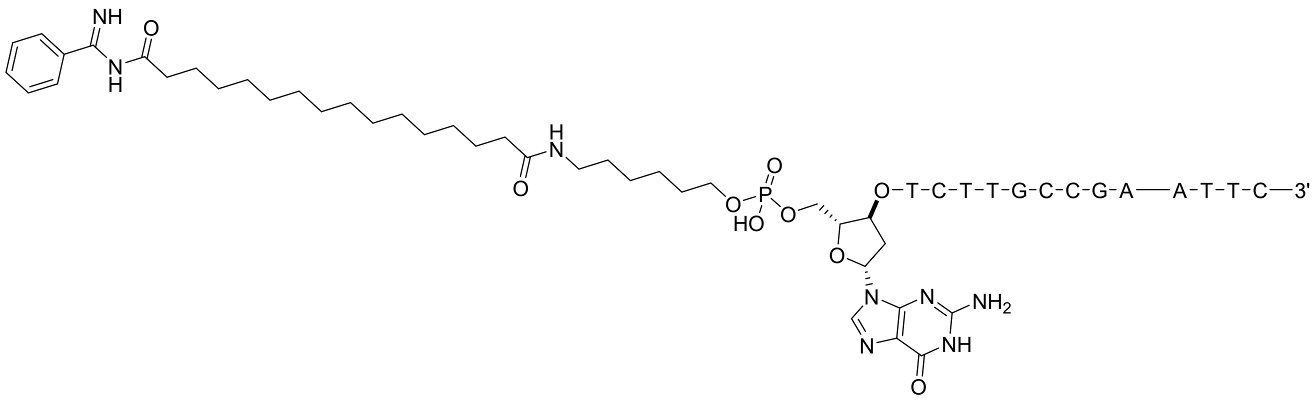

Figure S170: Mass spectrum of DNA-tagged product of reverse amide coupling of HP3 and benzimidamide using TPGS-750-M as the surfactant analysed by DNA mass spectrometry gradient A.

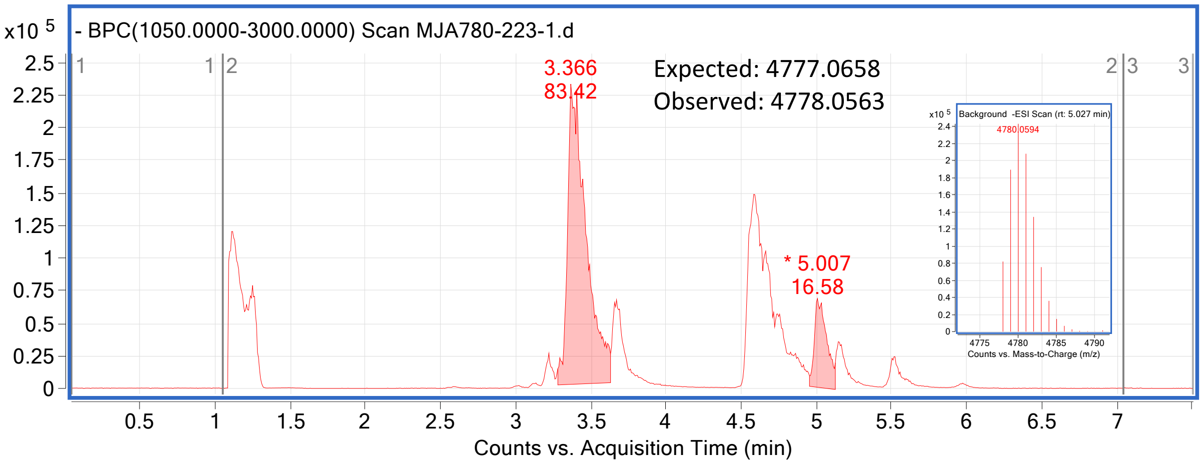

Figure S171: Mass spectrum of DNA-tagged product of reverse amide coupling of HP3 and benzimidamide using TTAC as the surfactant analysed by DNA mass spectrometry gradient A.

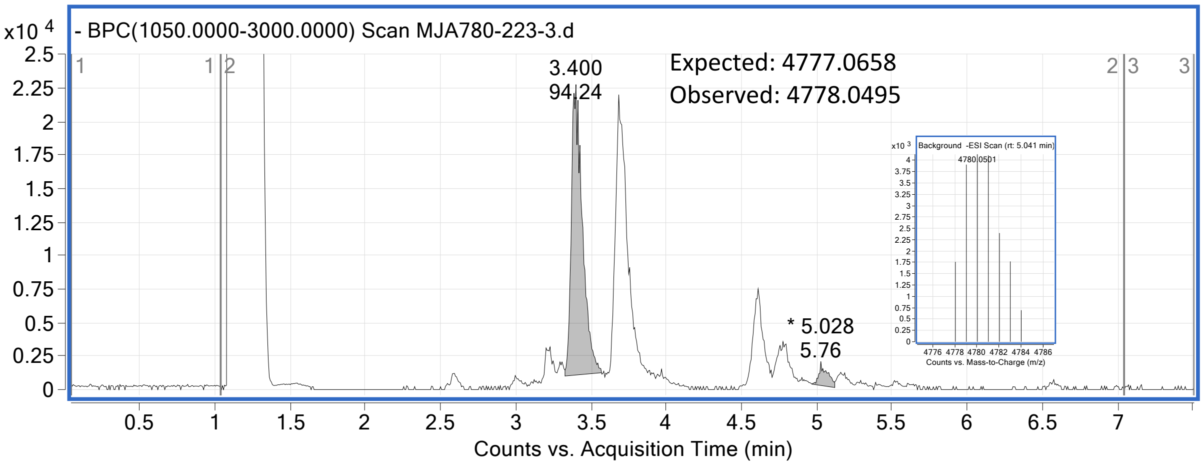

Figure S172: Mass spectrum of DNA-tagged product of reverse amide coupling of HP3 and benzimidamide using Sulfobetaine-16 as the surfactant analysed by DNA mass spectrometry gradient A.

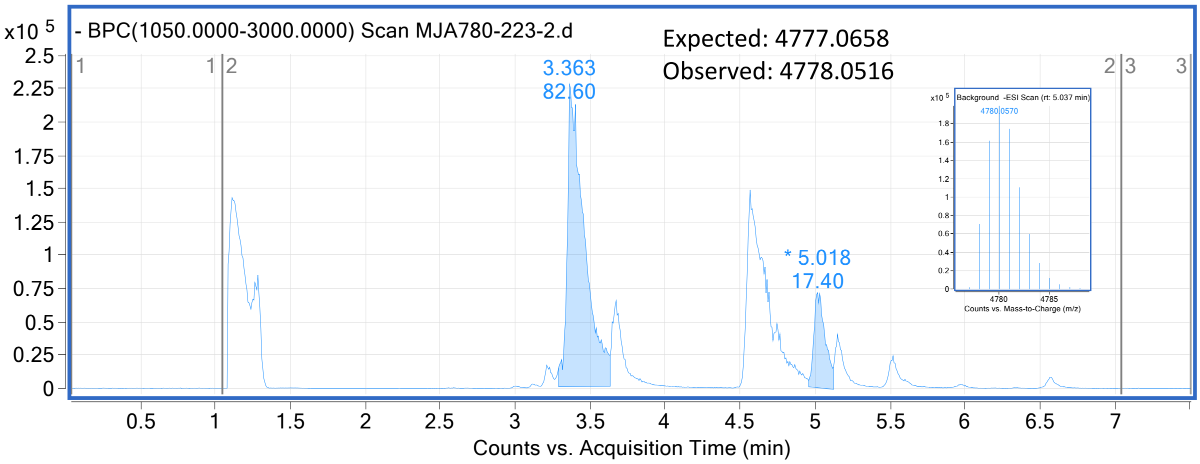

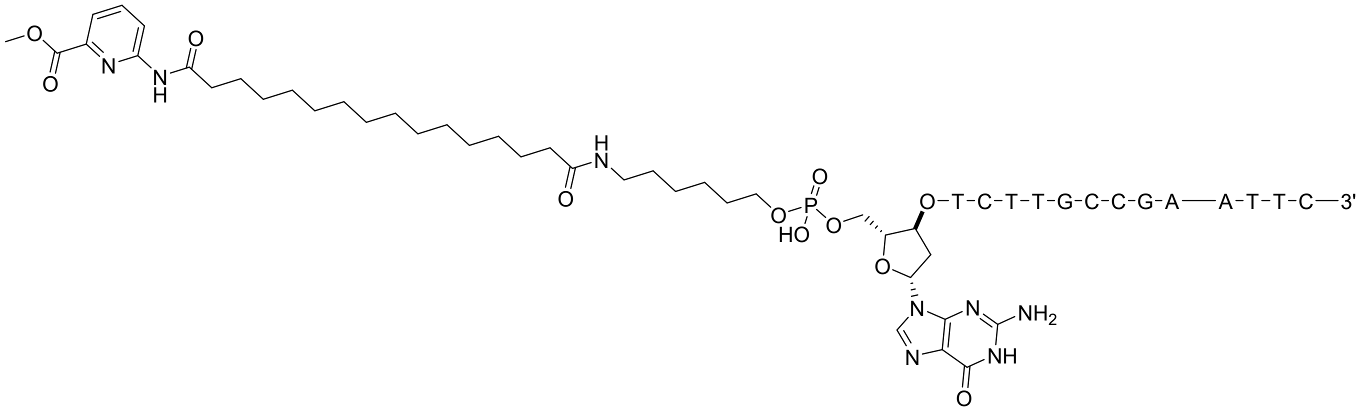

Figure S173: Mass spectrum of DNA-tagged product of reverse amide coupling of HP3 and methyl 6-aminopicolinate using TPGS-750-M as the surfactant analysed by DNA mass spectrometry gradient A.

Expected: 4795.0040 (ester hydrolysis product)
Observed: 4795.0286

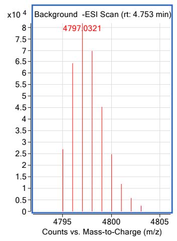

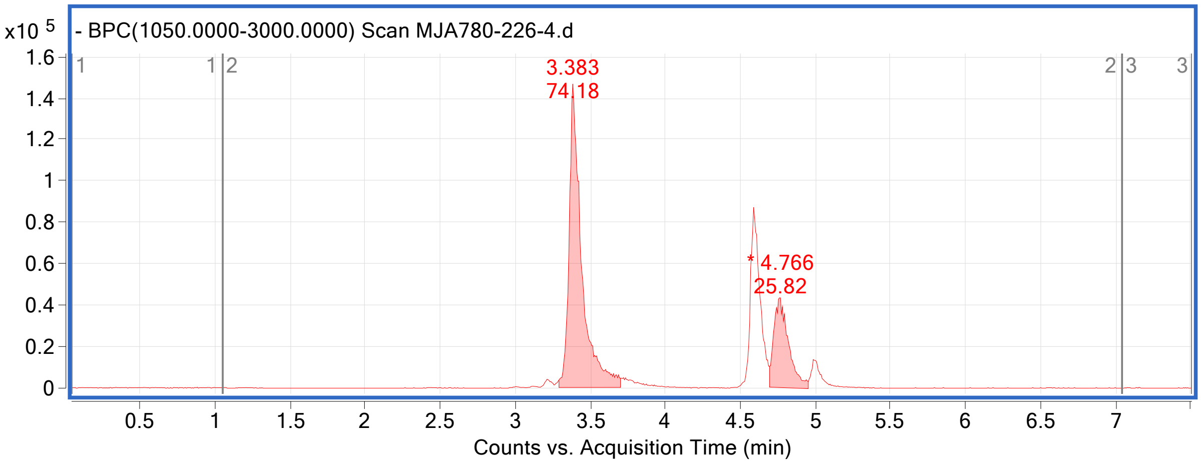

Figure S174: Mass spectrum of DNA-tagged product of reverse amide coupling of HP3 and methyl 6-aminopicolinate using TTAC as the surfactant analysed by DNA mass spectrometry gradient A.

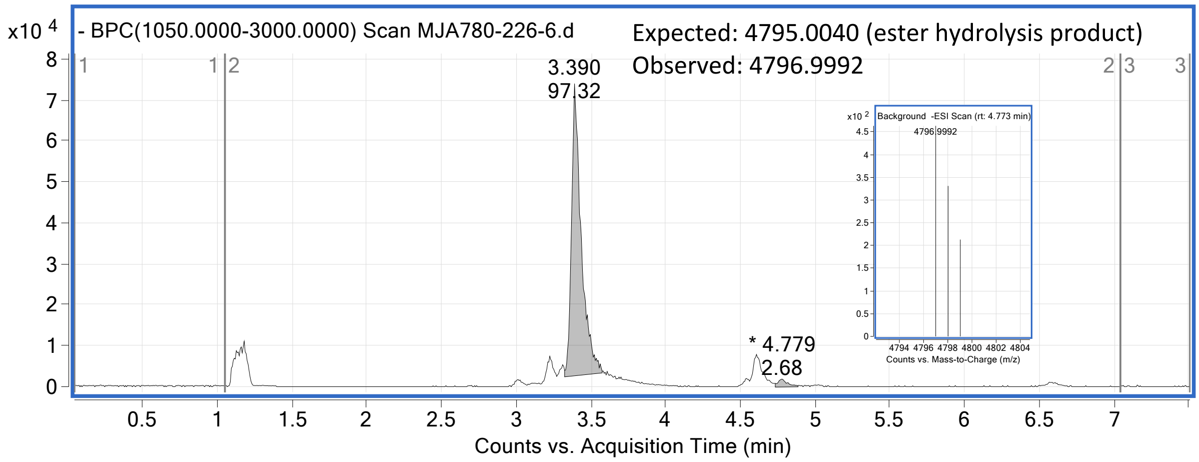

Figure S175: Mass spectrum of DNA-tagged product of reverse amide coupling of HP3 and methyl 6-aminopicolinate using Sulfobetaine-16 as the surfactant analysed by DNA mass spectrometry gradient A.

Expected: 4795.0040 (ester hydrolysis product)
Observed: 4795.0288

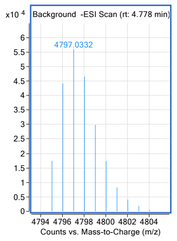

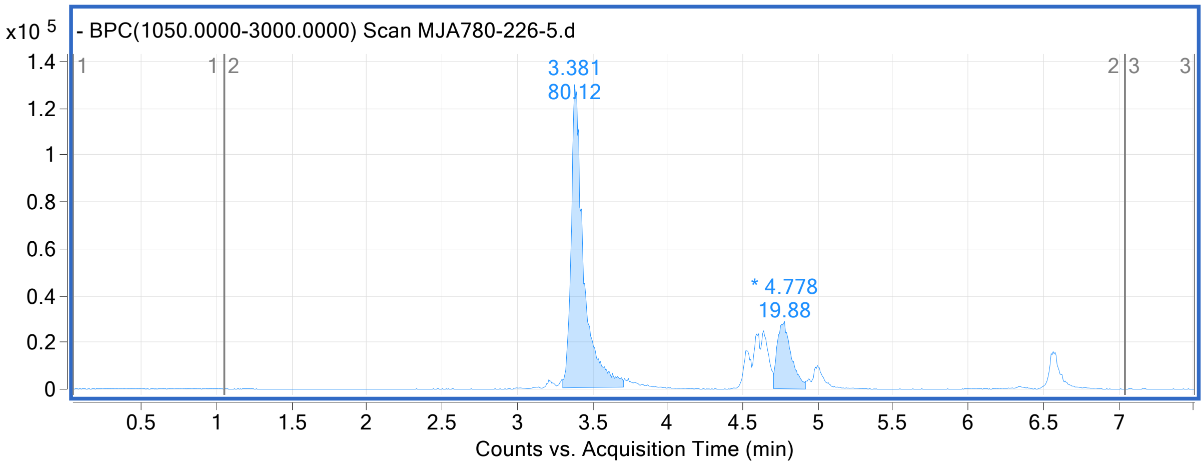

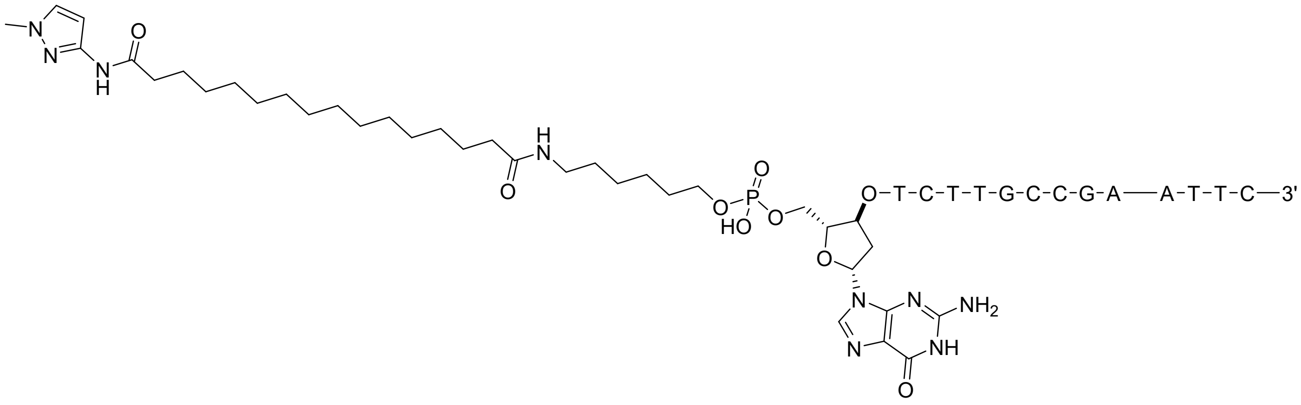

Figure S176: Mass spectrum of DNA-tagged product of reverse amide coupling of HP3 and 1-methyl-1*H*-pyrazol-3-amine using TPGS-750-M as the surfactant analysed by DNA mass spectrometry gradient A.

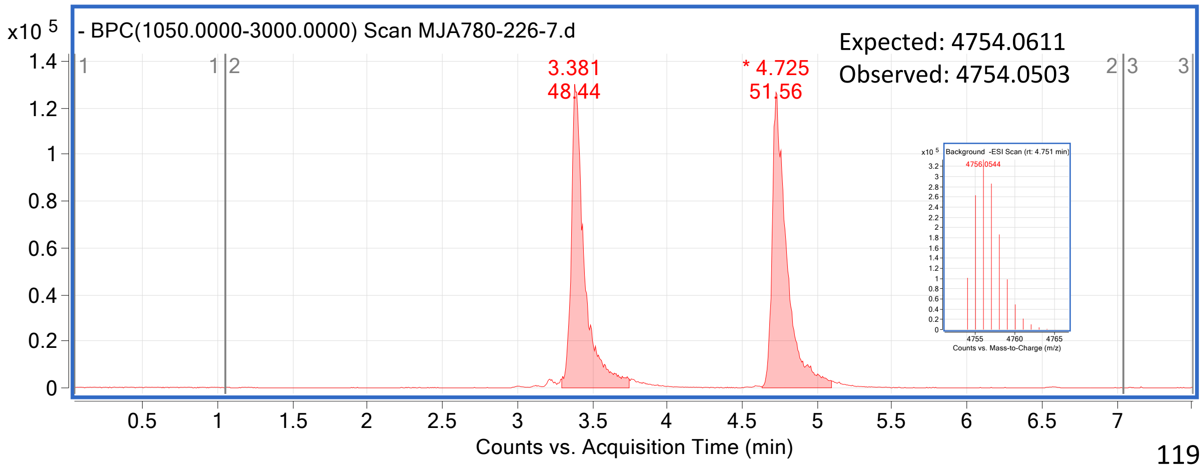

Figure S177: Mass spectrum of DNA-tagged product of reverse amide coupling of HP3 and 1-methyl-1*H*-pyrazol-3-amine using TTAC as the surfactant analysed by DNA mass spectrometry gradient A.

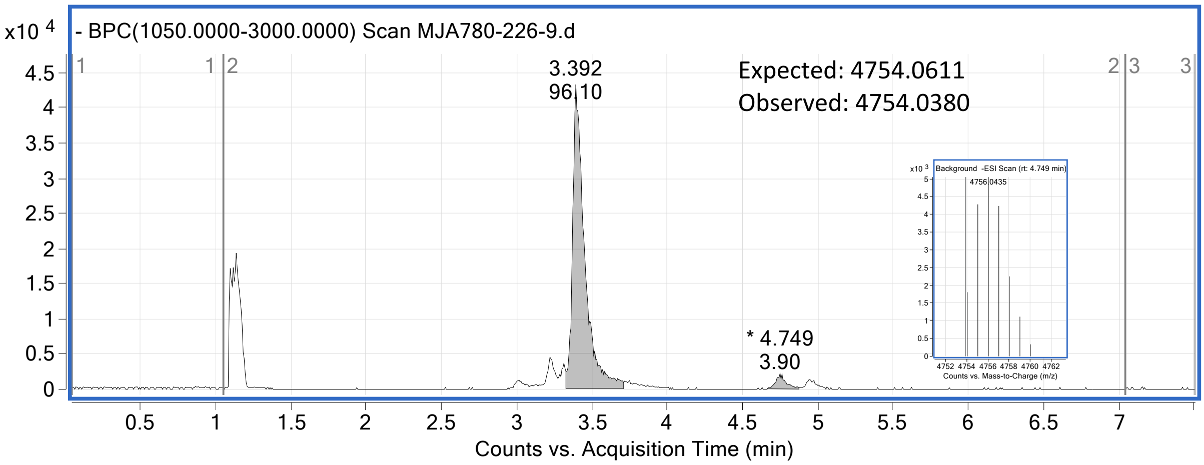

Figure S178: Mass spectrum of DNA-tagged product of reverse amide coupling of HP3 and 1-methyl-1*H*-pyrazol-3-amine using Sulfobetaine-16 as the surfactant analysed by DNA mass spectrometry gradient A.

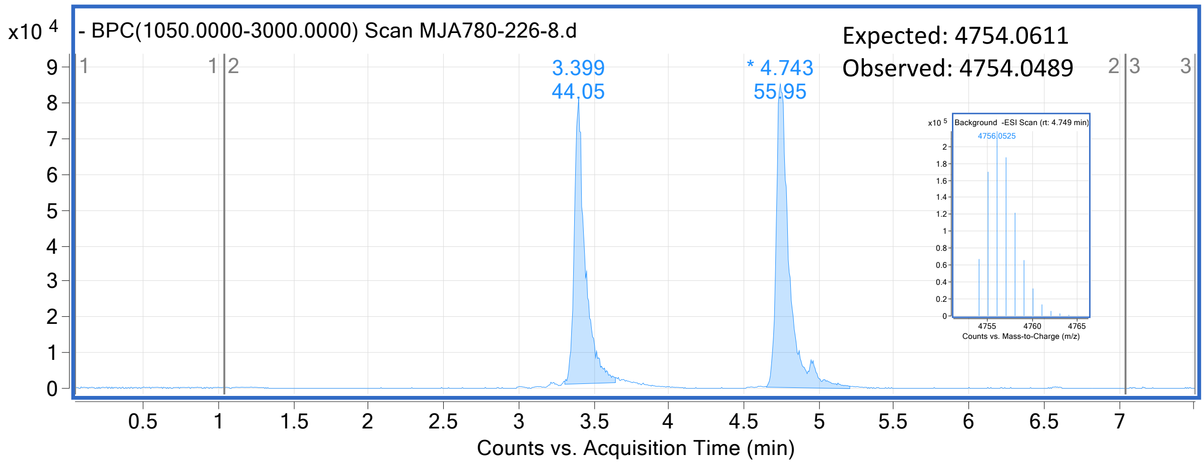

Table 11 Chromatograms

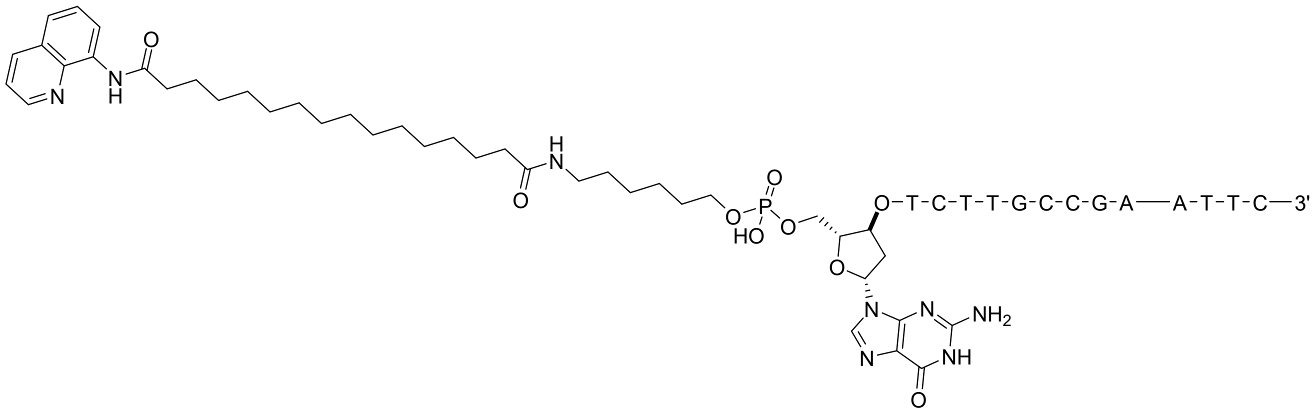

Figure S179: Mass spectrum of DNA-tagged product of reverse amide coupling of HP3 and quinolin-8-amine using TPGS-750-M as the surfactant analysed by DNA mass spectrometry gradient A.

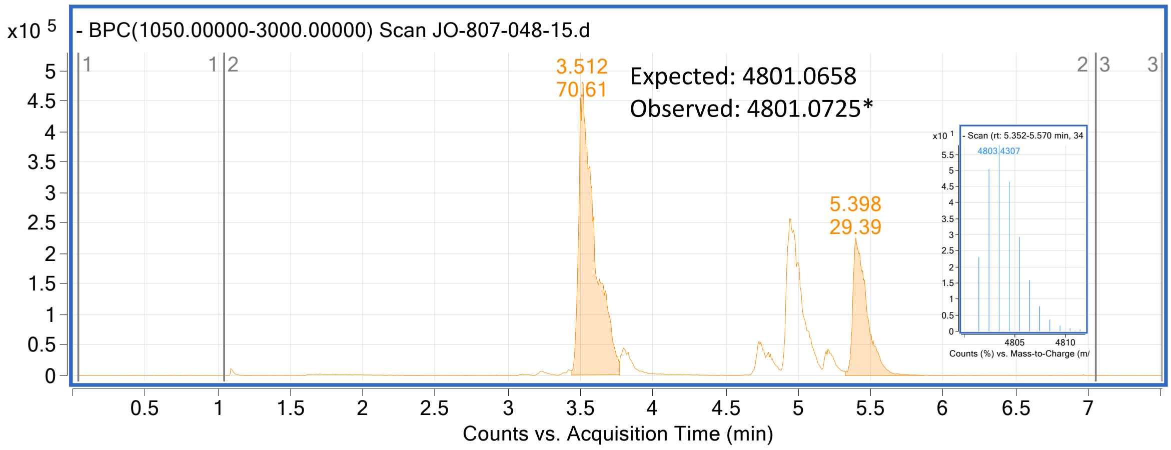

Figure S180: Mass spectrum of DNA-tagged product of reverse amide coupling of HP3 and quinolin-8-amine using Sulfobetaine-16 as the surfactant analysed by DNA mass spectrometry gradient A.

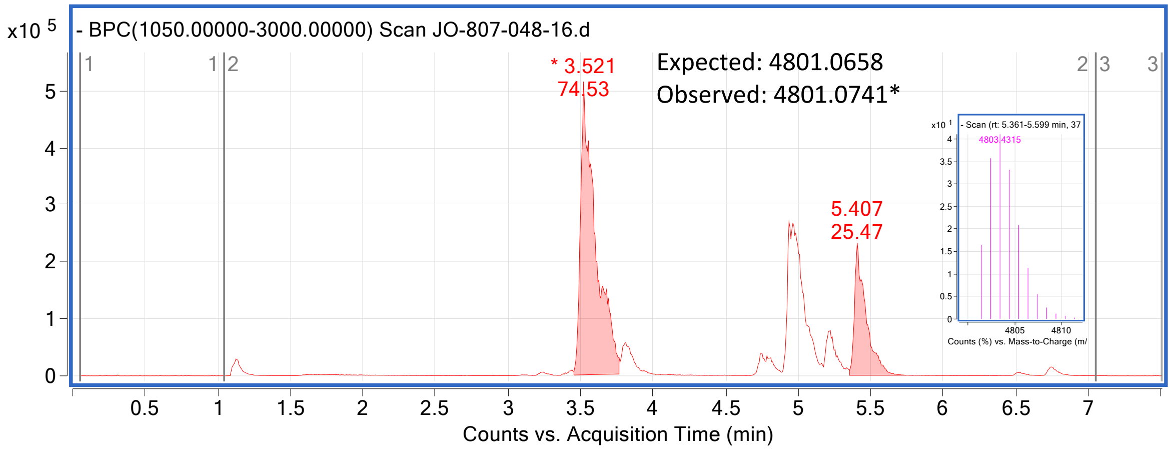

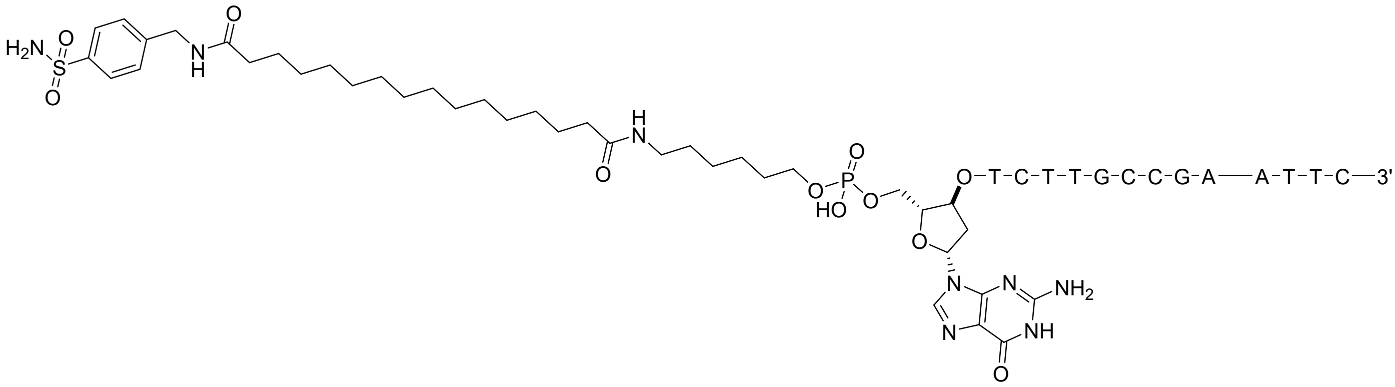

Figure S181: Mass spectrum of DNA-tagged product of reverse amide coupling of HP3 and 4-aminobenzenesulfonamide using TPGS-750-M as the surfactant analysed by DNA mass spectrometry gradient A.

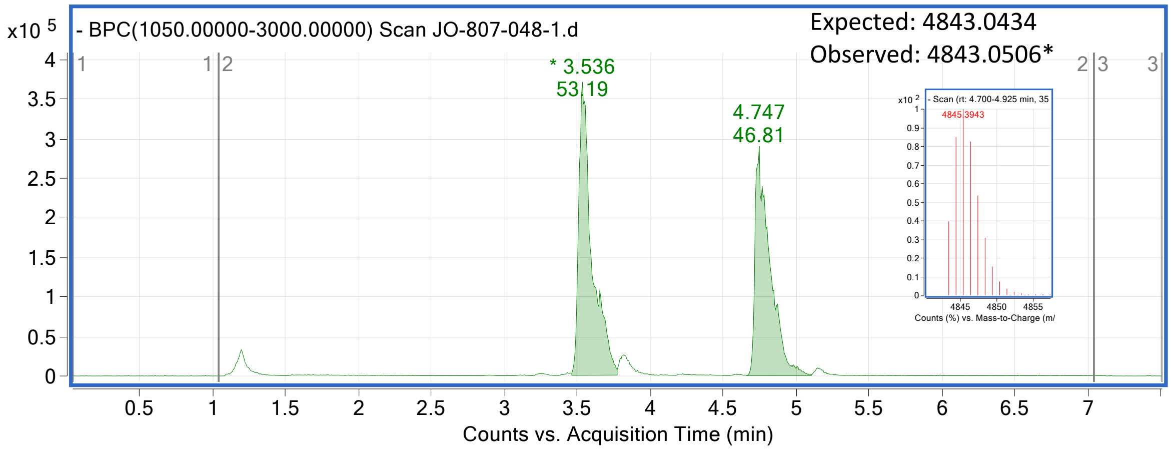

Figure S182: Mass spectrum of DNA-tagged product of reverse amide coupling of HP3 and 4-aminobenzenesulfonamide using Sulfobetaine-16 as the surfactant analysed by DNA mass spectrometry gradient A.

Expected: 4843.0434
Observed: 4843.0513*

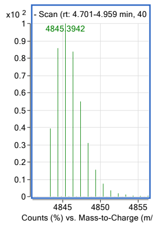

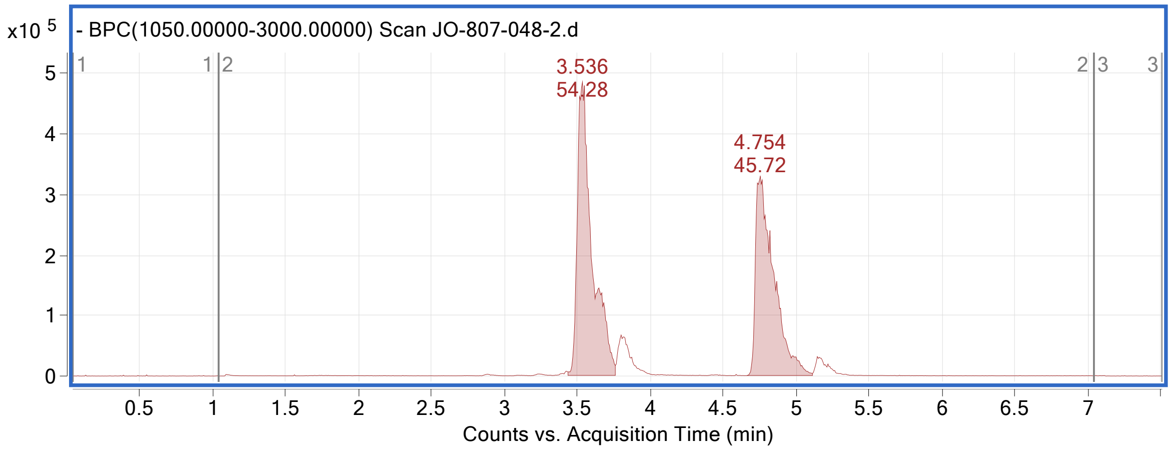

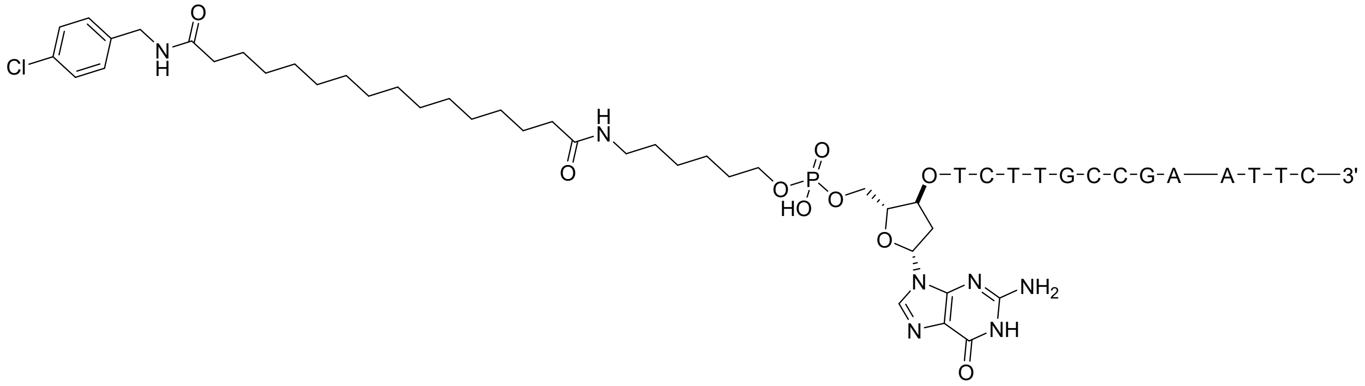

Figure S183: Mass spectrum of DNA-tagged product of reverse amide coupling of HP3 and (4-chlorophenyl)methanamine using TPGS-750-M as the surfactant analysed by DNA mass spectrometry gradient A.

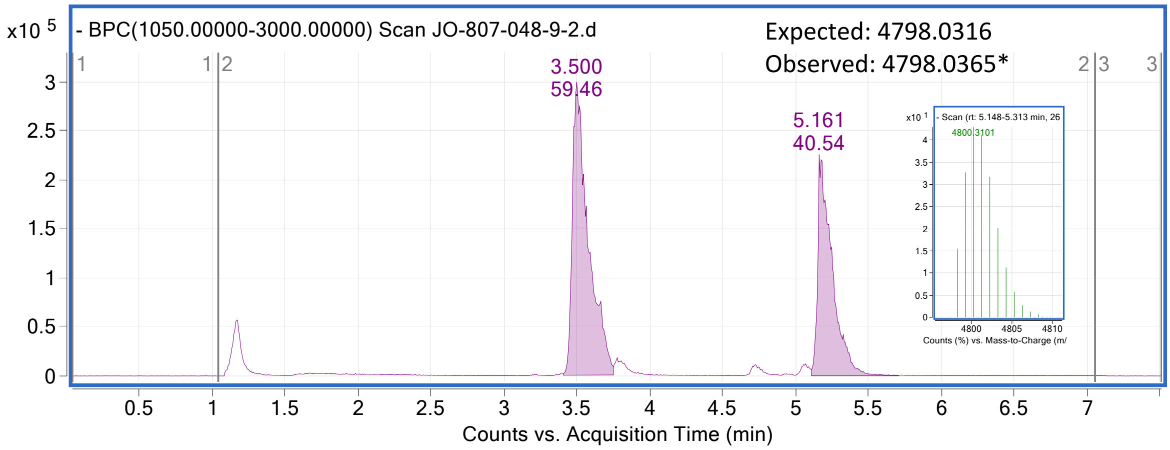

Figure S184: Mass spectrum of DNA-tagged product of reverse amide coupling of HP3 and (4-chlorophenyl)methanamine using Sulfobetaine-16 as the surfactant analysed by DNA mass spectrometry gradient A.

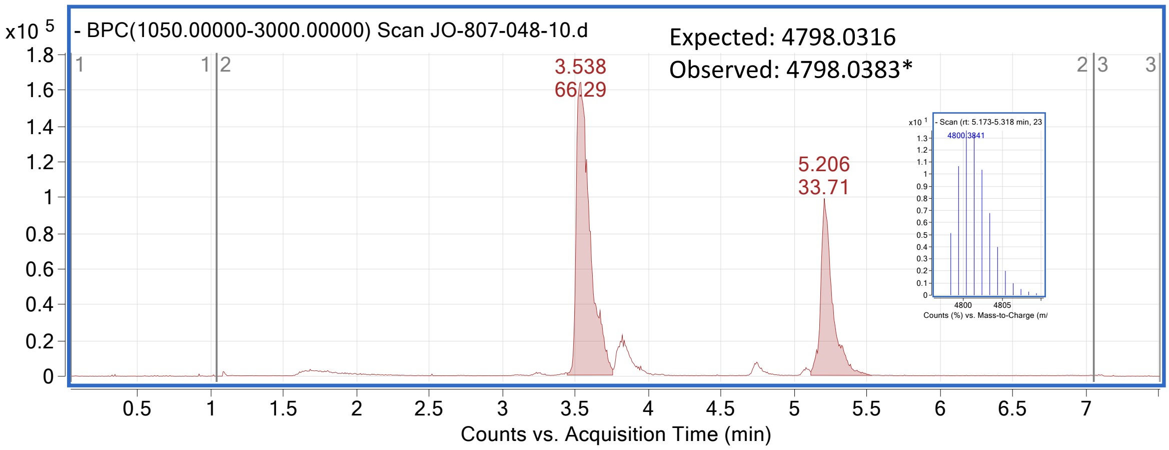

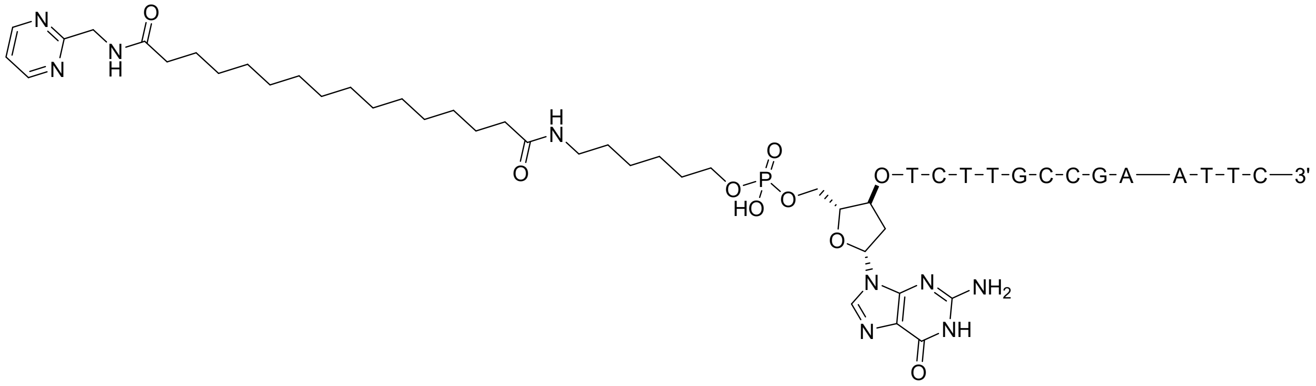

Figure S185: Mass spectrum of DNA-tagged product of reverse amide coupling of HP3 and pyrimidin-2-ylmethanamine using TPGS-750-M as the surfactant analysed by DNA mass spectrometry gradient A.

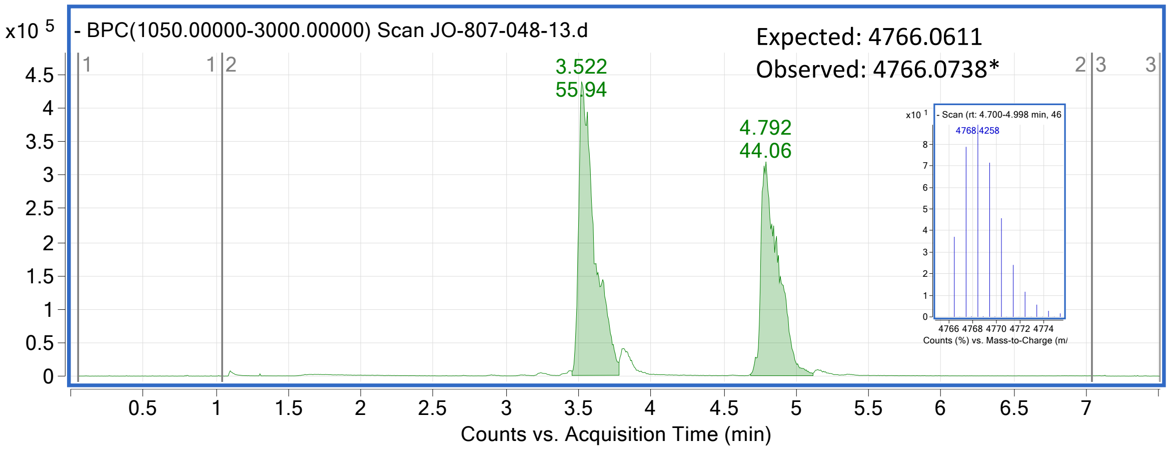

Figure S186: Mass spectrum of DNA-tagged product of reverse amide coupling of HP3 and pyrimidin-2-ylmethanamine using Sulfobetaine-16 as the surfactant analysed by DNA mass spectrometry gradient A.

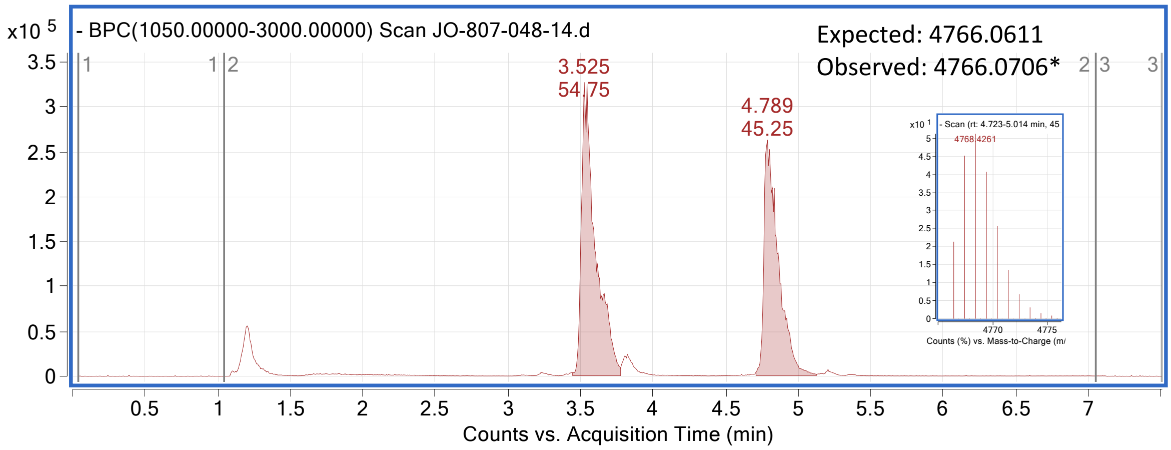

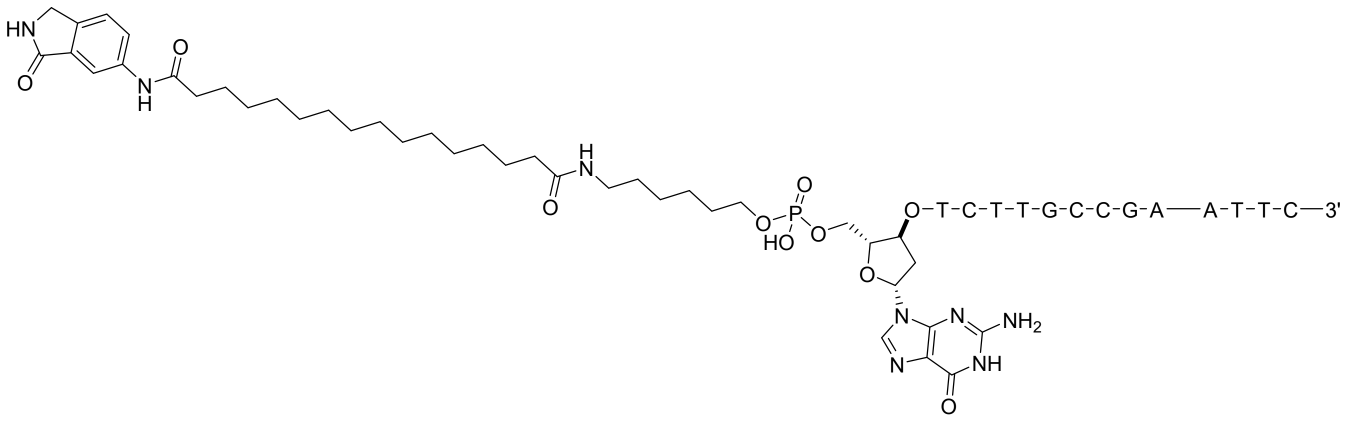

Figure S187: Mass spectrum of DNA-tagged product of reverse amide coupling of HP3 and 6-aminoisoindolin-1-one using TPGS-750-M as the surfactant analysed by DNA mass spectrometry gradient A.

Expected: 4805.0608
Observed: 4805.0675*

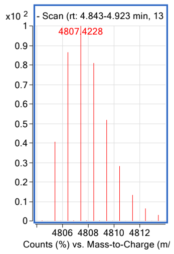

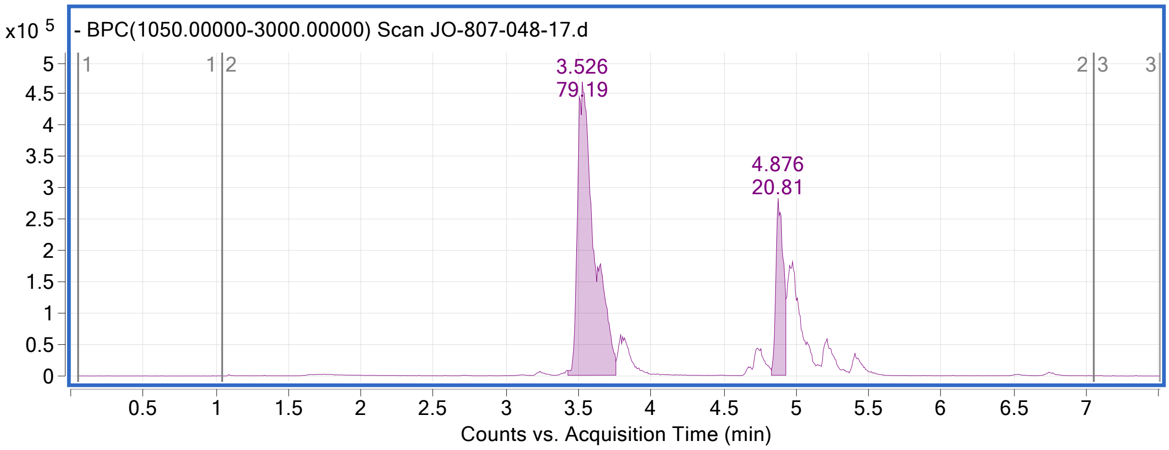

Figure S188: Mass spectrum of DNA-tagged product of reverse amide coupling of HP3 and 6-aminoisoindolin-1-one using Sulfobetaine-16 as the surfactant analysed by DNA mass spectrometry gradient A.

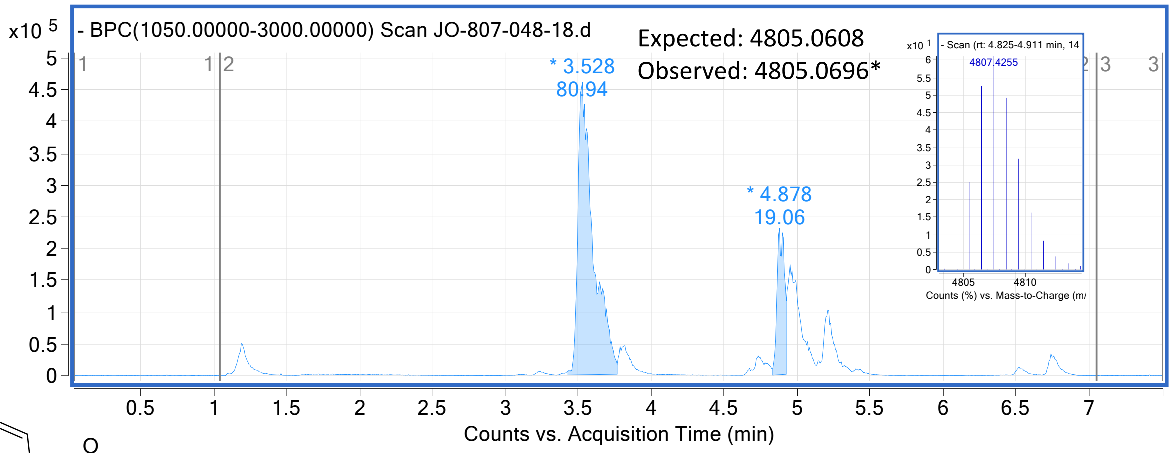

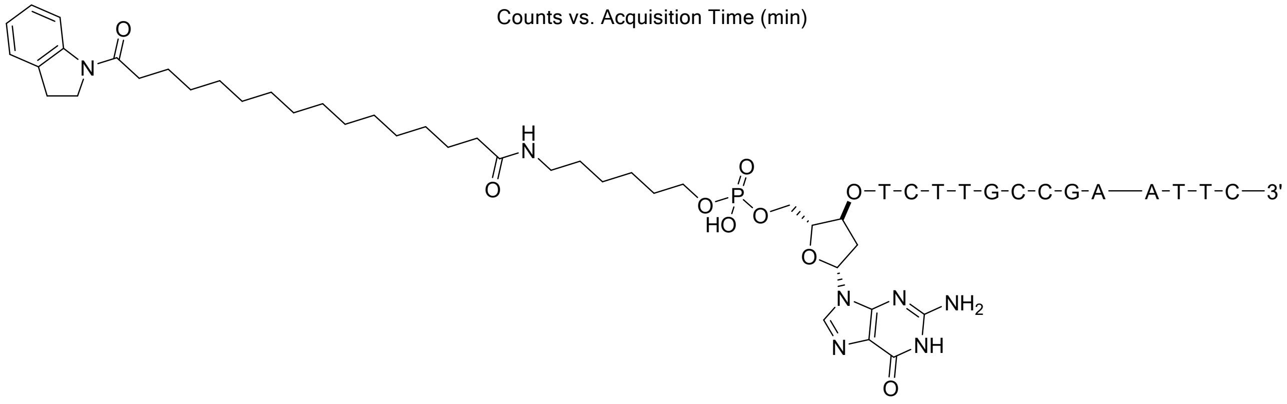

Figure S189: Mass spectrum of DNA-tagged product of reverse amide coupling of HP3 and indoline using TPGS-750-M as the surfactant analysed by DNA mass spectrometry gradient A.

Expected: 4776.0706
Observed: 4776.1514*

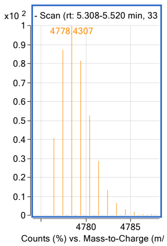

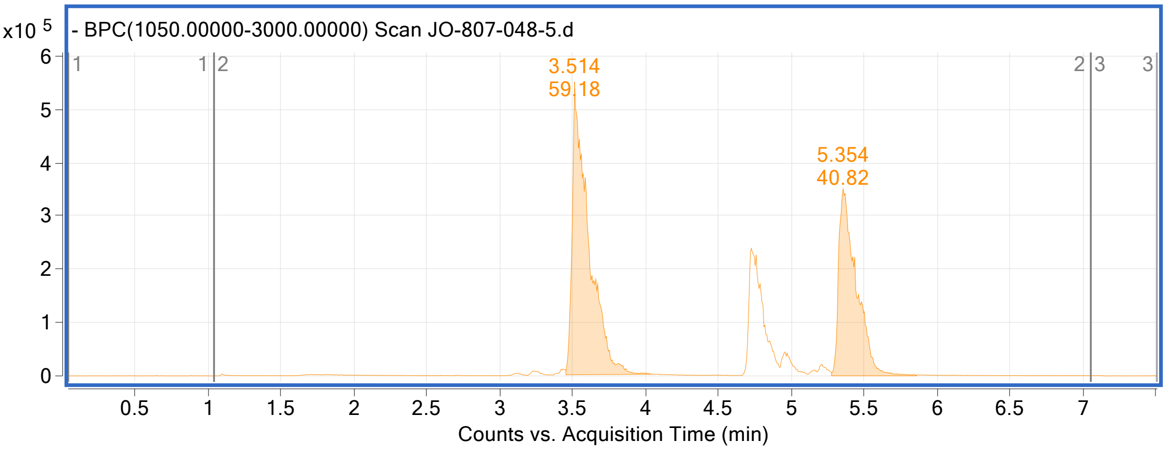

Figure S190: Mass spectrum of DNA-tagged product of reverse amide coupling of HP3 and indoline using Sulfobetaine-16 as the surfactant analysed by DNA mass spectrometry gradient A.

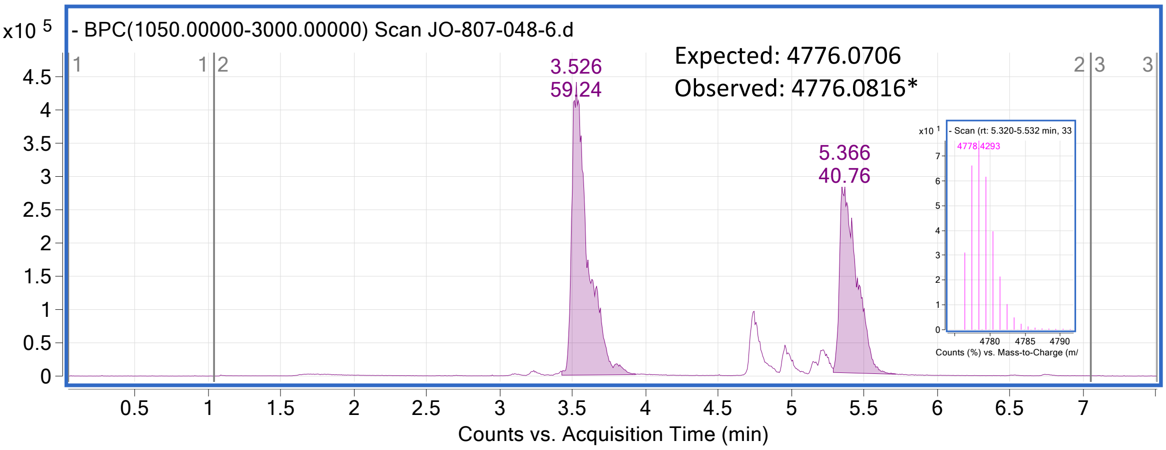

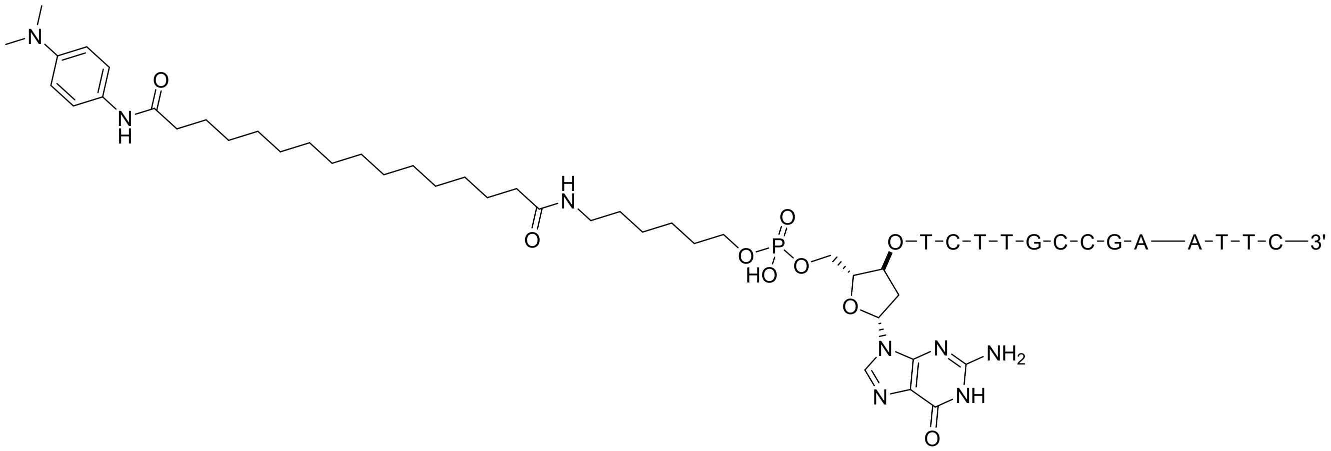

Figure S191: Mass spectrum of DNA-tagged product of reverse amide coupling of HP3 and *N’*,*N’*-dimethylbenzene-1,4-diamine using TPGS-750-M as the surfactant analysed by DNA mass spectrometry gradient A.

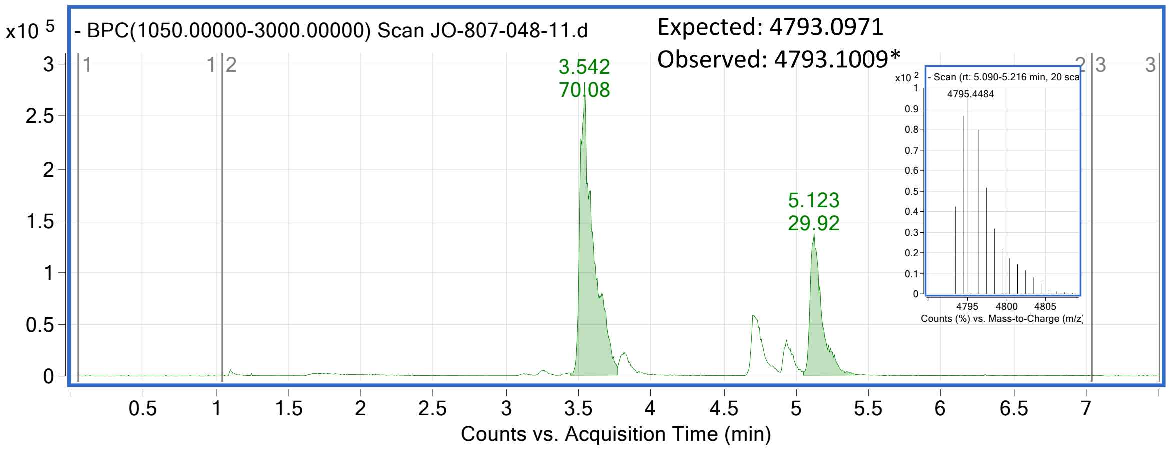

Figure S192: Mass spectrum of DNA-tagged product of reverse amide coupling of HP3 and *N’*,*N’*-dimethylbenzene-1,4-diamine using Sulfobetaine-16 as the surfactant analysed by DNA mass spectrometry gradient A.

Expected: 4793.0971
Observed: 4793.1060*

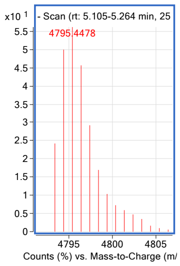

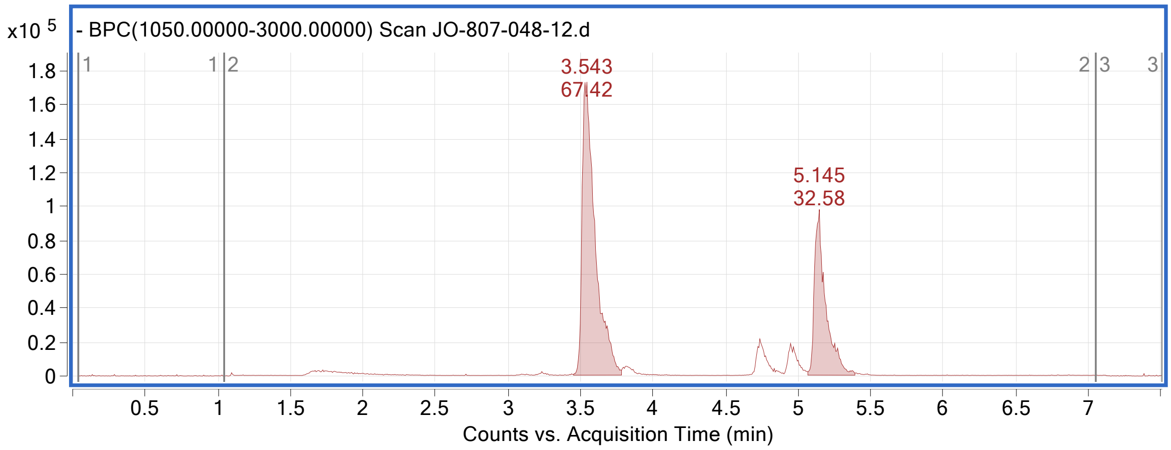

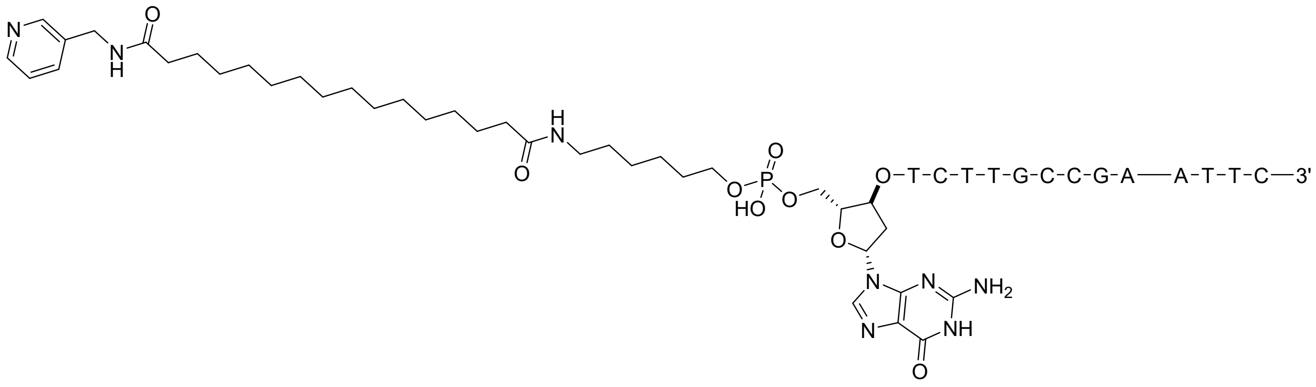

Figure S193: Mass spectrum of DNA-tagged product of reverse amide coupling of HP3 and pyridin-3-ylmethanamine using TPGS-750-M as the surfactant analysed by DNA mass spectrometry gradient A.

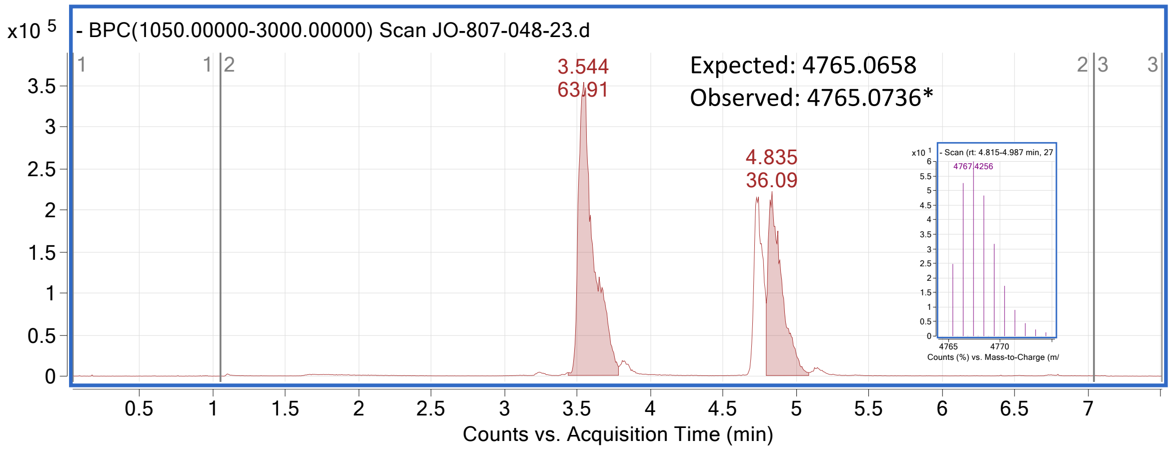

Figure S194: Mass spectrum of DNA-tagged product of reverse amide coupling of HP3 and pyridin-3-ylmethanamine using Sulfobetaine-16 as the surfactant analysed by DNA mass spectrometry gradient A.

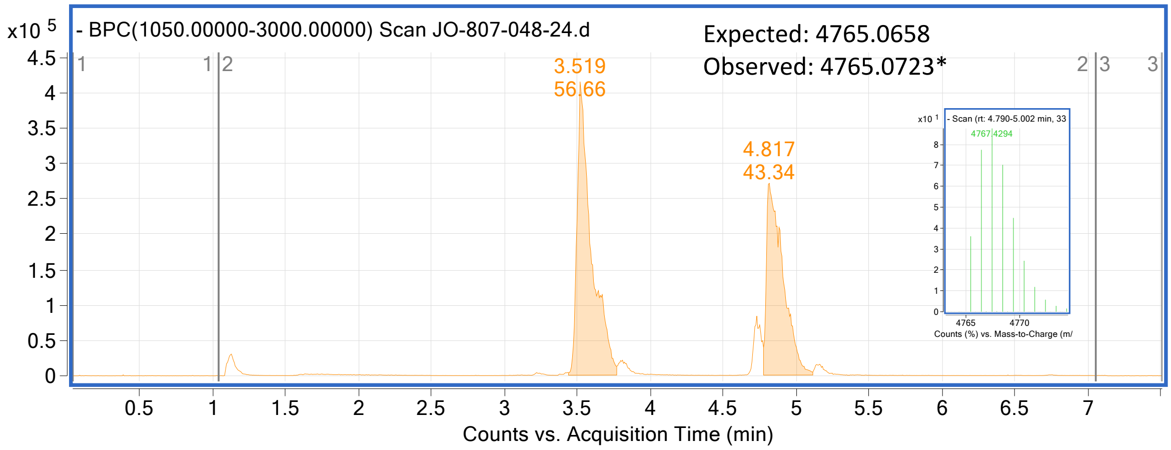

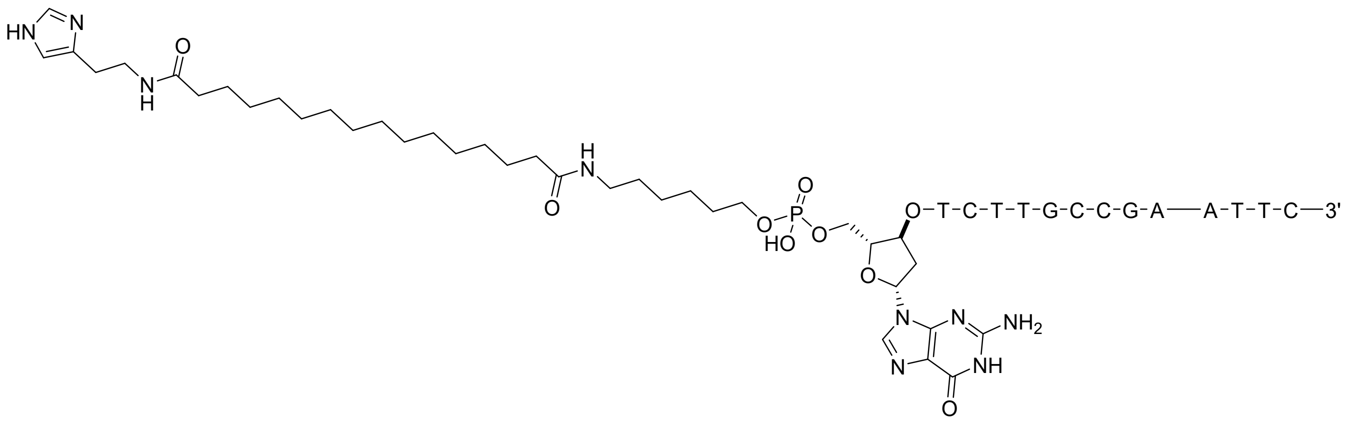

Figure S195: Mass spectrum of DNA-tagged product of reverse amide coupling of HP3 and 2-(1*H*-imidazol-4-yl)ethan-1-amine using TPGS-750-M as the surfactant analysed by DNA mass spectrometry gradient A.

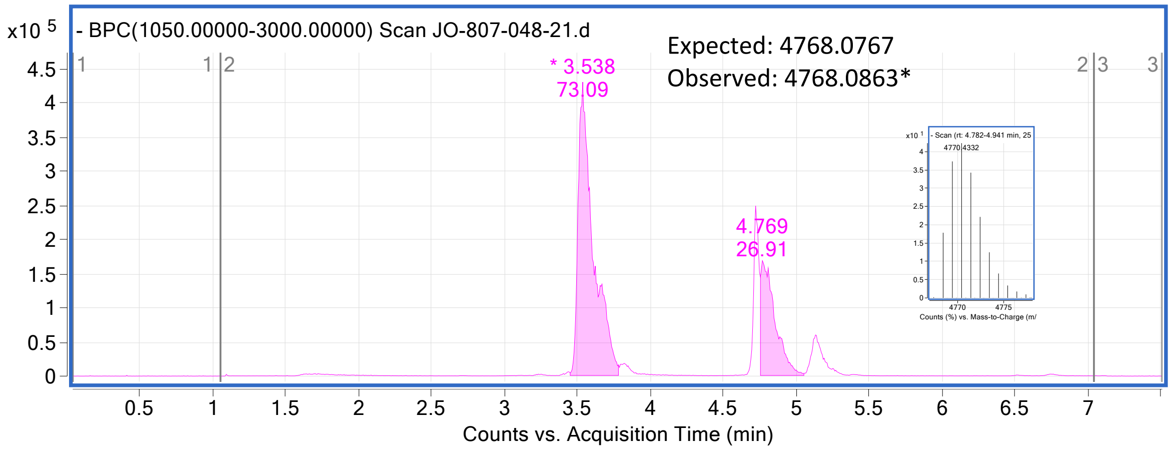

Figure S196: Mass spectrum of DNA-tagged product of reverse amide coupling of HP3 and 2-(1*H*-imidazol-4-yl)ethan-1-amine using Sulfobetaine-16 as the surfactant analysed by DNA mass spectrometry gradient A.

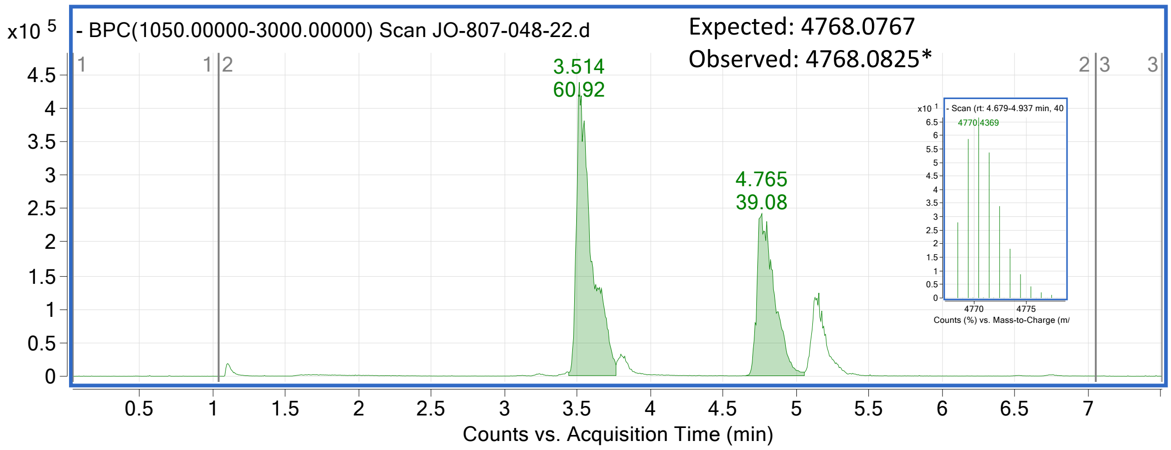

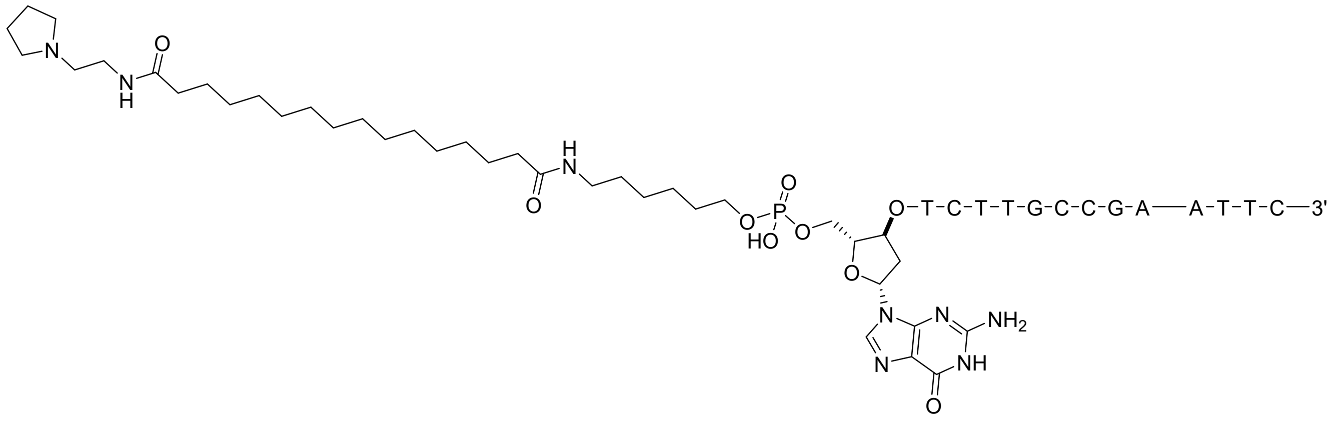

Figure S197: Mass spectrum of DNA-tagged product of reverse amide coupling of HP3 and 2-(pyrrolidin-1-yl)ethan-1-amine using TPGS-750-M as the surfactant analysed by DNA mass spectrometry gradient A.

Expected: 4771.1128
Observed: 4771.1123*

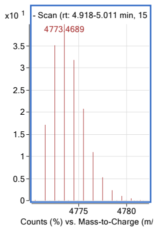

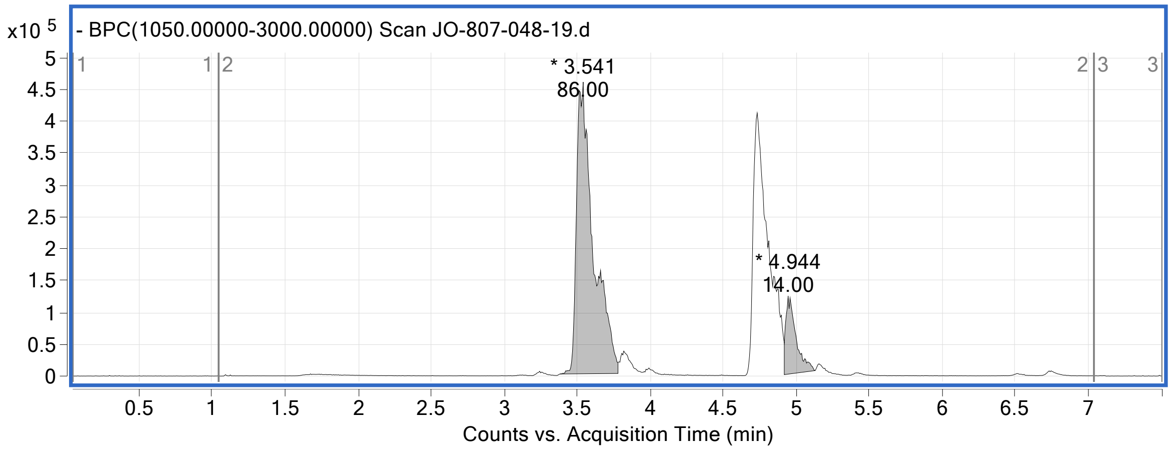

Figure S198: Mass spectrum of DNA-tagged product of reverse amide coupling of HP3 and 2-(pyrrolidin-1-yl)ethan-1-amine using Sulfobetaine-16 as the surfactant analysed by DNA mass spectrometry gradient A.

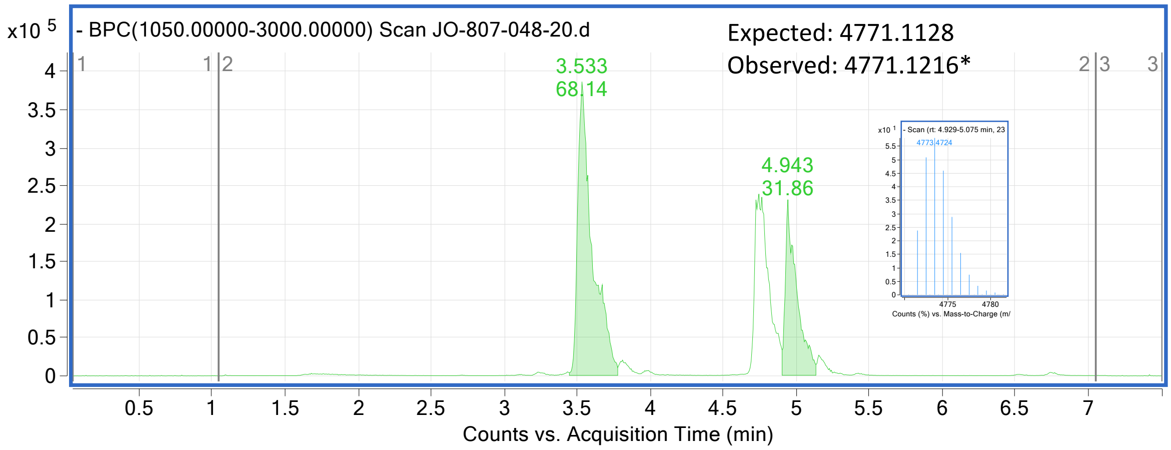

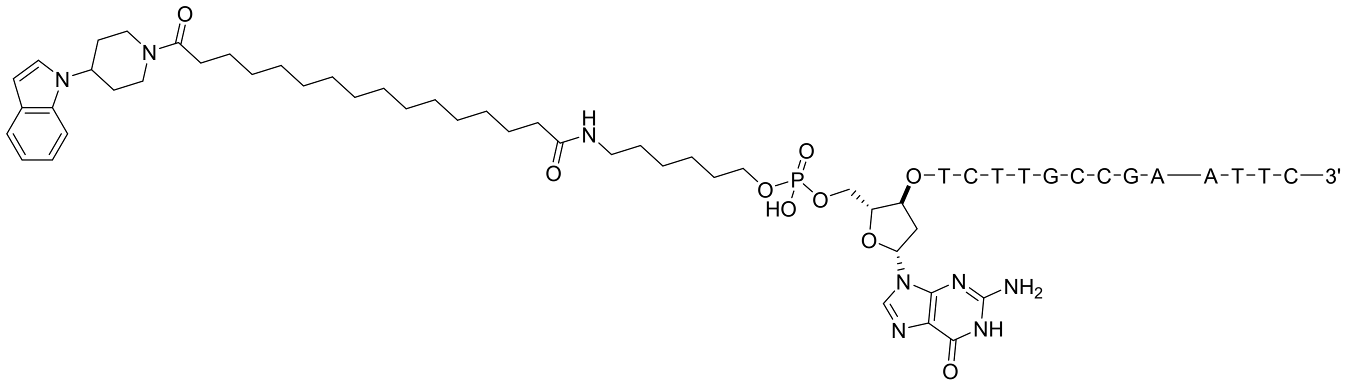

Figure S199: Mass spectrum of DNA-tagged product of reverse amide coupling of HP3 and 1-(piperidin-4-yl)-1*H*-indole using TPGS-750-M as the surfactant analysed by DNA mass spectrometry gradient A.

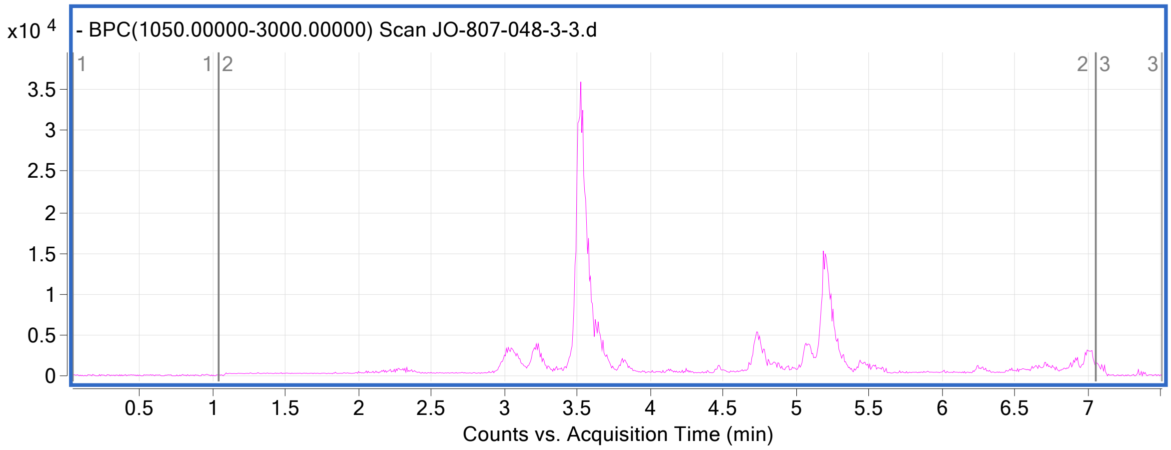

Figure S200: Mass spectrum of DNA-tagged product of reverse amide coupling of HP3 and 1-(piperidin-4-yl)-1*H*-indole using Sulfobetaine-16 as the surfactant analysed by DNA mass spectrometry gradient A.

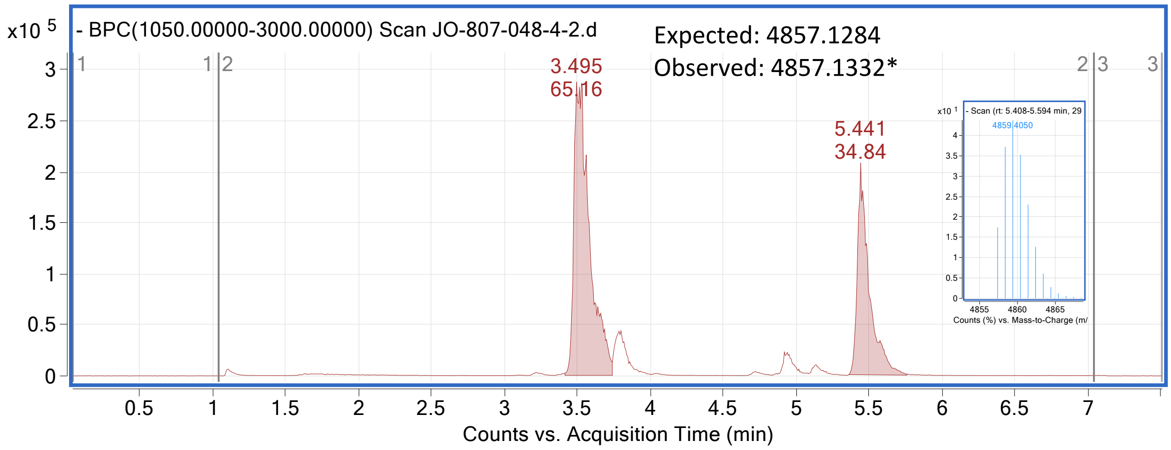

Representative Encoded Compound Synthesis

Experimental Procedure and Characterisation of compounds **7-10**:

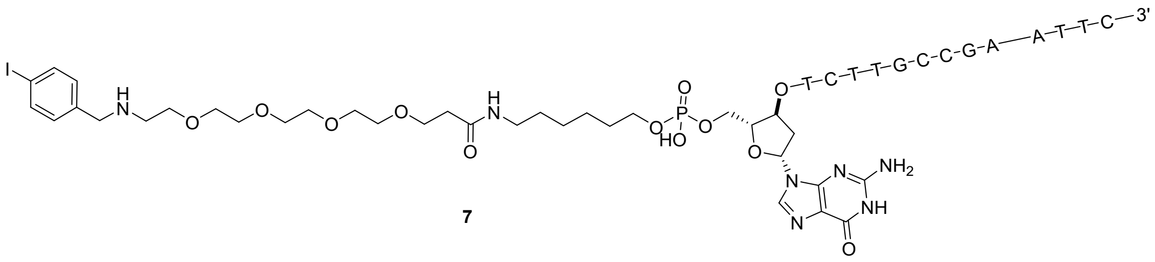

7

PEG amine HP5 was reacted with 4-iodobenzaldehyde according to the general on-DNA reductive amination procedure to give product **7** following ethanol precipitation.

Figure S201: Mass spectrum of product **7** from the reaction of PEG amine HP with 4-iodobenzaldehyde using TTAC as the surfactant analysed by DNA mass spectrometry gradient A.

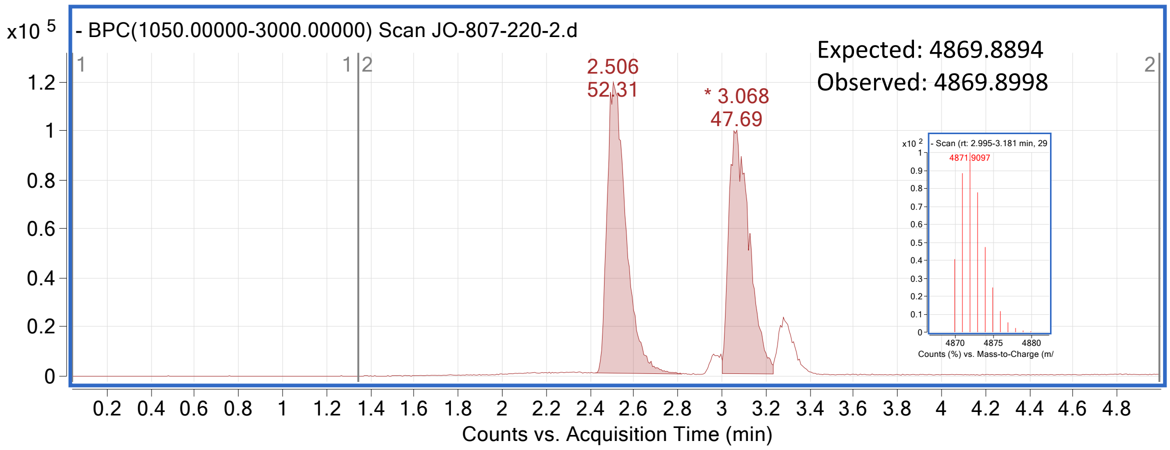

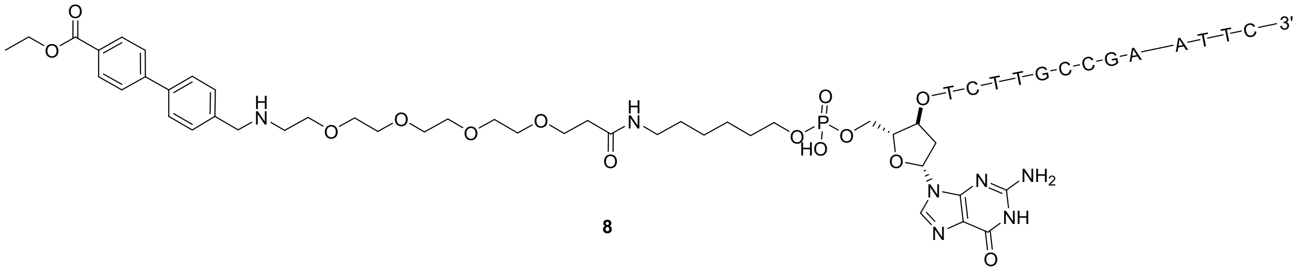

8

Compound **7** was reacted with (4-(ethoxycarbonyl)phenyl)boronic acid according to the general on-DNA Suzuki-Miyaura cross-coupling to give product **8** following ethanol precipitation.

Figure S202: Mass spectrum of product **8** from the reaction of **7** and (4-(ethoxycarbonyl)phenyl)boronic acid using 4-dodecylbenzenesulfonic acid as the surfactant analysed by DNA mass spectrometry gradient A.

Expected (acid): 4864.0139
Observed: 4864.0064

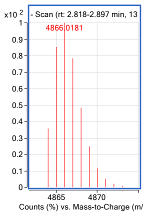

Expected (ester): 4892.0452
Observed: 4892.0394

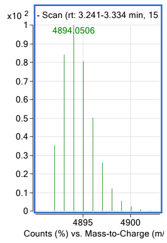

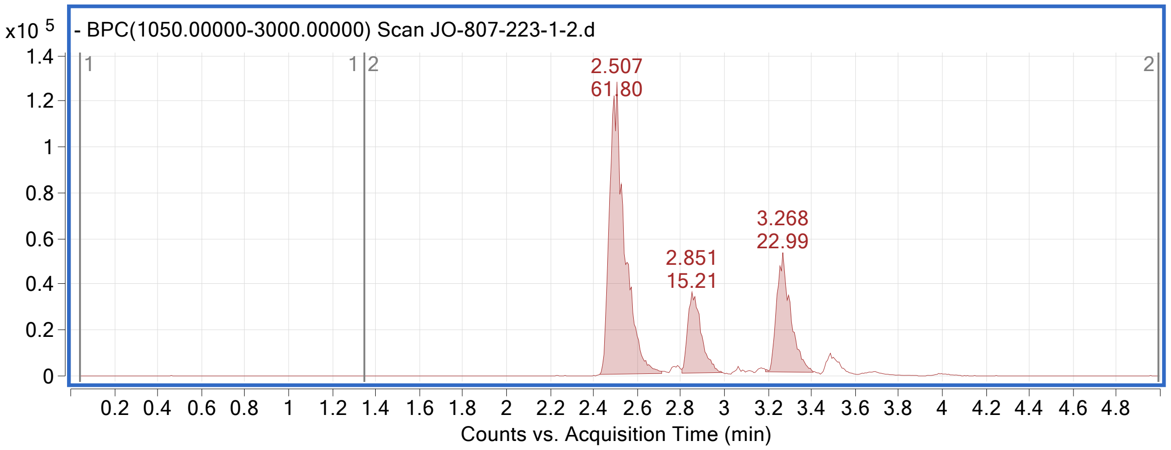

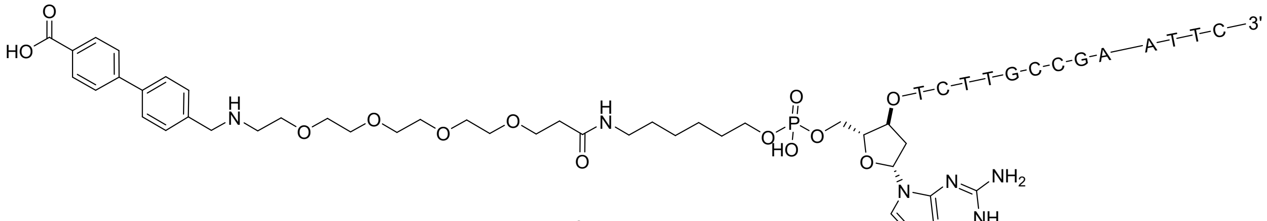

9

To a 1.5 mL Eppendorf™ Polypropylene DNA LoBind Tube was added compound **8** (2 nmol in 50 µL H_2O) and LiOH (0.5 M in H_2O, 50 µL) and the reaction mixture vortexed at room temperature at 800 rpm for 30 mins. The product was added to an Amicon Ultra-0.5 Centrifugal 3 kDa Cut-off Filter Unit, DEPC-treated water (350 µL was added and the solution centrifuged at room temperature for 10 mins. DEPC-treated water (200 µL) was added to the filter and the mixture centrifuged at room temperature for 10 mins. This was repeated 2 more times, then the filter was inverted and centrifuged at 4,300 rpm at room temperature for 2 mins to yield the aqueous DNA product **9** which was analysed by mass spectrometry.

Figure S203: Mass spectrum of product **9** analysed by DNA mass spectrometry method A.

Expected (acid): 4864.0139
Observed: 4864.0363

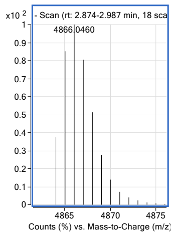

Expected (ester): 4892.0452
Observed: 4892.0571

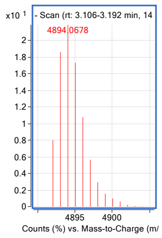

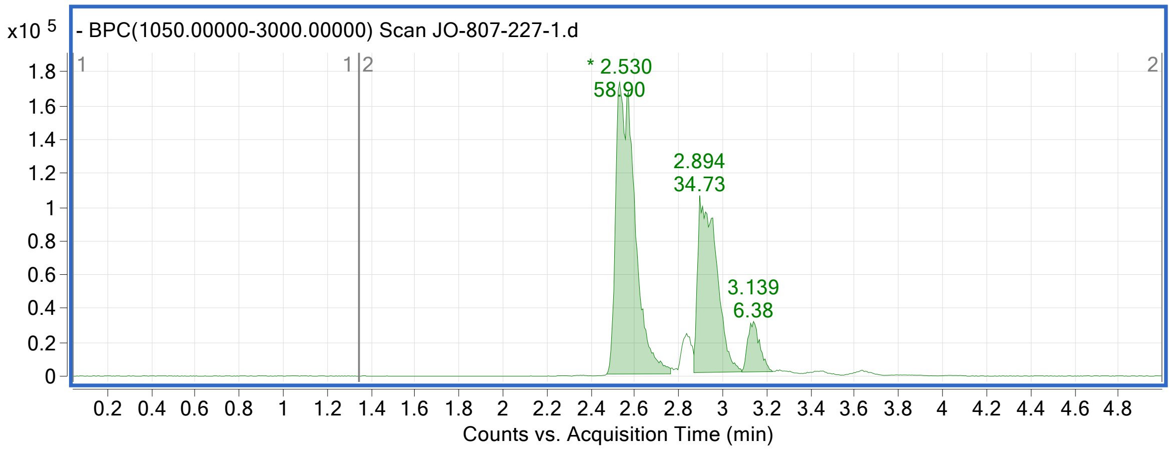

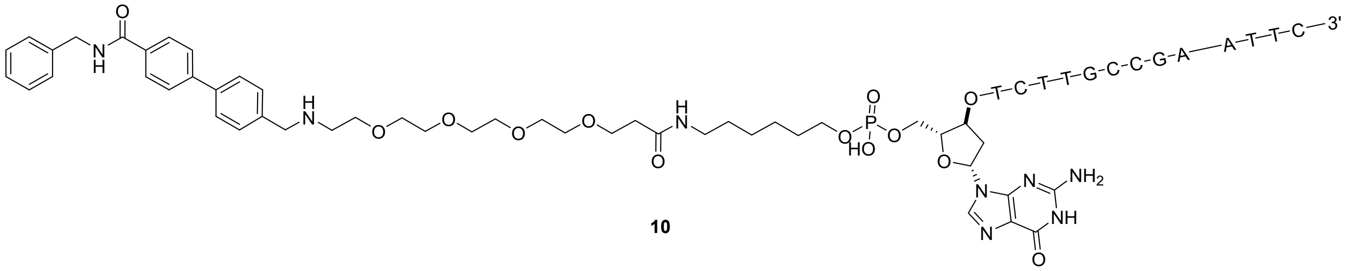

10

Compound **10** was synthesised from compound **9** and benzylamine according to the general on-DNA reverse amide coupling procedure.

Figure S204: Mass spectrum of product **10** from the reaction of **9** with benzylamine using sulfobetaine-16 as the surfactant analysed by DNA mass spectrometry gradient A.

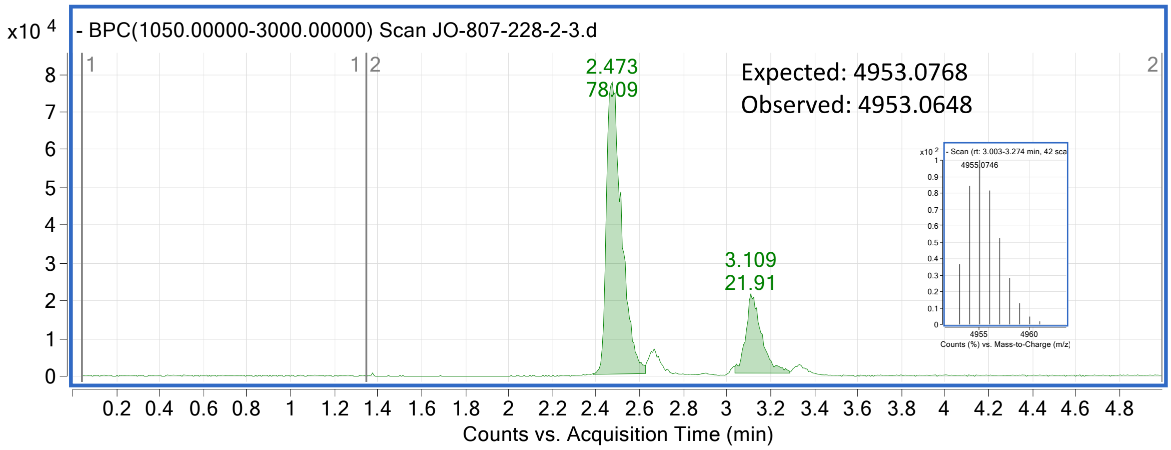

1x1x1 Library Synthesis

Figure S205: Synthetic scheme for the synthesis of a 1x1x1 library incorporating all 3 reactions.

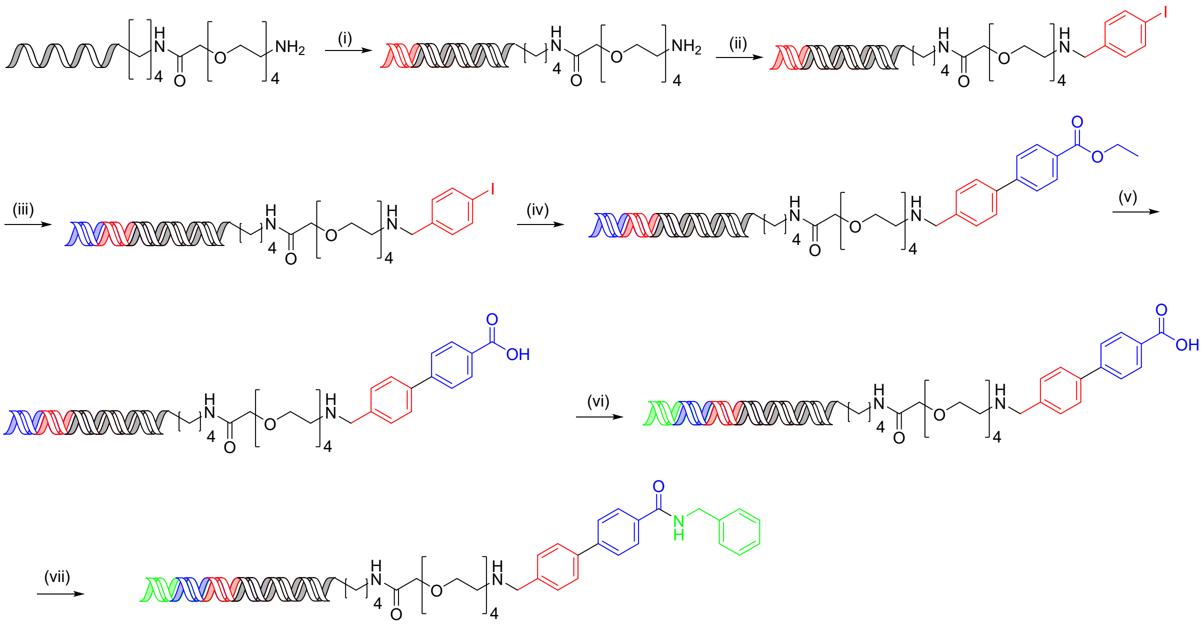

Conditions: (i) first ligation (DNA headpiece, primer, library codon and first DNA building block); (ii) borate buffer (350 mM, pH=10.8), aldehyde (400 mM), 5% surfactant, rt, 1.5 h then $NaBH_4$ (440 mM), rt 16 h; (iii) second ligation (second DNA building block); (iv) boronate (500 mM), Pd(dtbpf)Cl_2 (7.3 mM), K_3PO_4 (530 mM), 2% surfactant, 15% THF, 60 °C, 5 h; (v) LiOH (250 mM), rt, 30 mins; (vi) third ligation (third DNA building block); (vii) amine (0.5 M), HOAt (0.5 M), lutidine (1.5 M), DIC (0.5 M), 4.5% surfactant, 45 °C, 3 h.

The following code abbreviations for each DNA section have been used:

Code	Function	Sequence (5'-3')
A	Adapter – 5' amino-linked headpiece	GTCTTGCCGAATTC
A'	Complementary adapter	GAATTCGGCAAGAC
P	Primer	AGGTCGGTGTGAACGGATTTG
P'	Complementary primer	CAAATCCGTTCACACCGACCT
L	Library codon	AGTCGCGGAA
L'	Complementary library codon	TTCCGCGACT
OH1	Ligation overhang 1	GTAT
OH1'	Complementary overhang 1	ATAC
BB1	Building block 1	AGTGCATTCA
BB1'	Complementary building block 1	TGAATGCACT
OH2	Ligation overhang 2	CCTA
OH2'	Complementary overhang 2	TAGG
BB2	Building block 2	CCGCTAGGCT
BB2'	Complementary building block 2	AGCCTAGCGG
OH3	Ligation overhang 3	TACG
OH3'	Complementary overhang 3	CGTA

BB3	Building block 3	GAGGTCTTAC
BB3'	Complementary building block 3	GTAAGACCTC
P2	Complementary to P2'	TGACCTCAACTACATGGTCTACA
P2'	Primer (reverse)	TGTAGACCATGTAGTTGAGGTC A

Ligation Strategy:

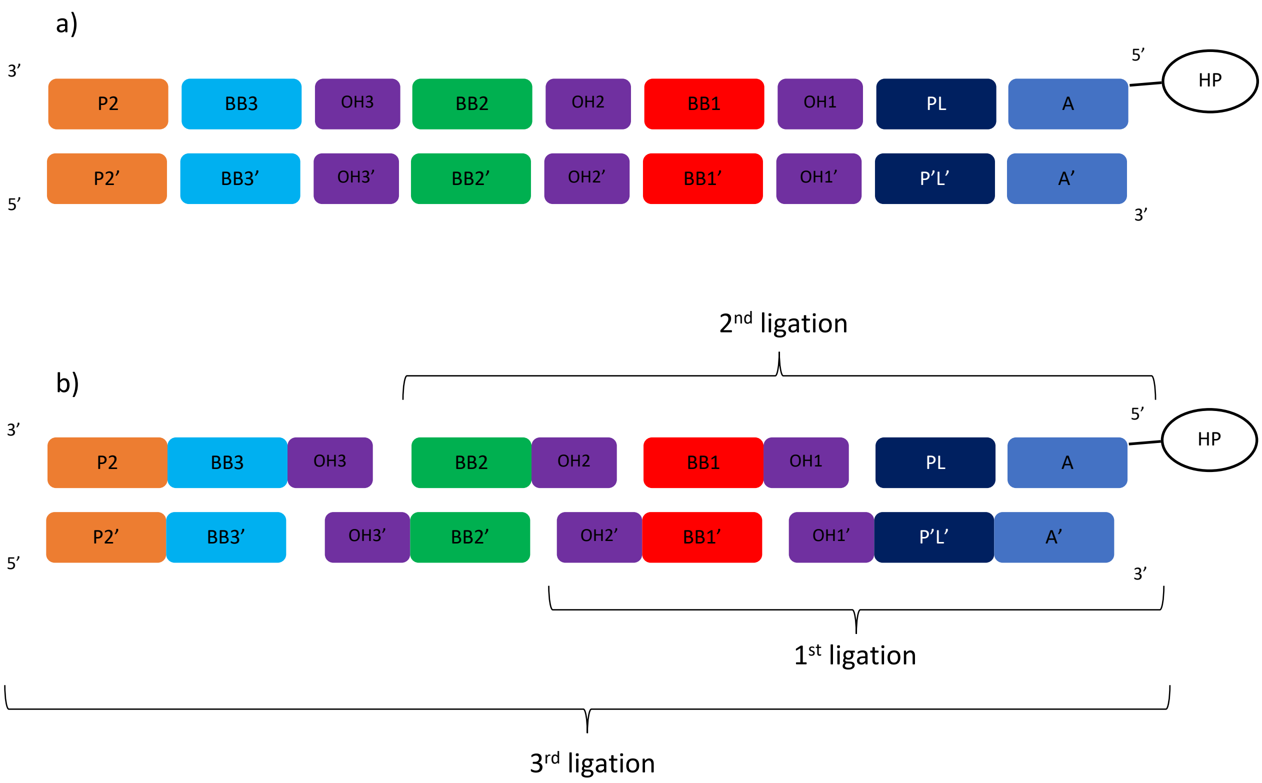

Cycle 1:

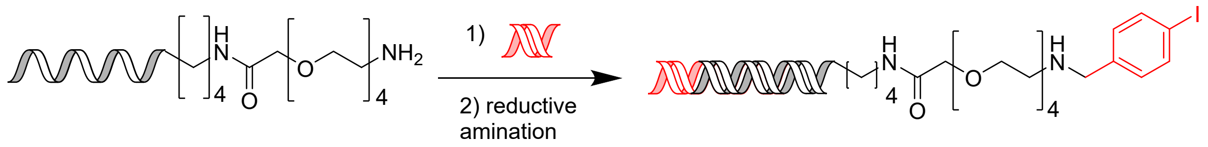

To individual Applied Biosystems™ MicroAmp® 96-well Reaction Plate wells was added the following DNA sequences (10 nmol total): P (AGGTCGGTGTGAACGGATTTGAGTCGCGGAA), OH1BB1 (GTAT AGTGCATTCA) and A'P'OH1' (GAATTCGGCAAGAC TTCCGCGACTCAAATCCGTTCACACCGACCT ATAC) (10 µL, 1 nmol), which were combined with ATP (2 µL, 10 mM in H_2O), PNK reaction buffer (2 µL, 500 mM Tris-HCl [pH 7.6 at 25 °C], 100 mM $MgCl_2$, 50 mM DTT, 1 mM spermidine), T4 Polynucleotide Kinase (1 µL, 10 U/µL) and DEPC-treated water (5 µL). The reaction was conducted at 37 °C for 1 h, followed by heating to 75 °C for 10 mins. DNA was used in subsequent ligation without further purification.

20 µL of each phosphorylation reaction was added to non-phosphorylated DNA sequences A (GTCTTGCCGAATTC) (5 µL, 1 nmol) and OH2'BB1' (TAGG TGAATGCACT) (10 µL, 1 nmol). To the DNA sequences was added 10X T4 DNA ligase buffer (9 µL, 400 mM Tris-HCl, 100 mM $MgCl_2$, 100 mM DTT, 5 mM ATP), T4 DNA ligase (1.5 µL, 30 U/µL) and DEPC-treated water (4.5 µL). The reactions were

conducted at 37 °C for 16 h followed by heating to 75 °C for 10 mins. The product was then visualised by gel electrophoresis, showing a band between 50 and 75 base pairs (expected 59 base pairs), before being purified according to the general ethanol precipitation procedure. The resulting pellet was reconstituted in water (5 µL) for the reductive amination step.

The double-stranded DNA was reacted with 4-iodobenzaldehyde according to the general on-DNA reductive amination procedure to give 8.7 nmol DNA product following ethanol precipitation.

Figure S206: Gel electrophoresis visualisation of 1st ligation showing a band between 50 and 75 base pairs in length (expected 59 base pairs).

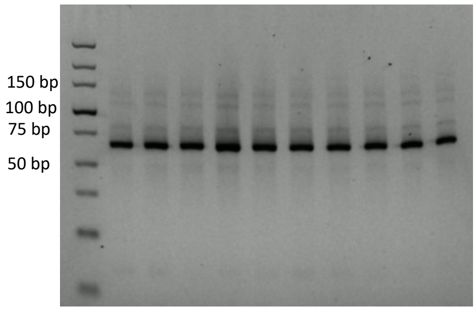

Cycle 2:

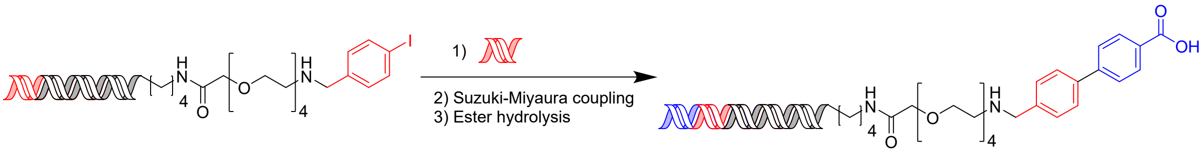

To individual Applied Biosystems™ MicroAmp® 96-well Reaction Plate wells was added DNA sequences cycle 1 product (10 µL, 1.1 nmol) and OH2BB2 (CCTA TGTCGCATTA) (10 µL, 1 nmol), which were combined with ATP (2 µL, 10 mM in H_2O), PNK reaction buffer (2 µL, 500 mM Tris-HCl [pH 7.6 at 25 °C], 100 mM $MgCl_2$, 50 mM DTT, 1 mM spermidine), T4 Polynucleotide Kinase (1 µL, 10 U/µL) and DEPC-treated water (5 µL). The reaction was conducted at 37 °C for 1 h, followed by heating to 75 °C for 10 mins. DNA was used in subsequent ligation without further purification.

22 µL of the combined phosphorylation reaction of OH2BB2 and 20 µL of the combined phosphorylation reaction of cycle 1 product (1.1 nmol) were added to non-phosphorylated DNA sequence OH3'BB2' (CGTA AGCCTAGCGG) (11 µL, 1.1 nmol). To the DNA sequences was added 10X T4 DNA ligase buffer (9 µL, 400 mM Tris-HCl, 100 mM $MgCl_2$, 100 mM DTT, 5 mM ATP), T4 DNA ligase (1.5 µL, 30 U/µL) and DEPC-treated water (26.5 µL). The reactions were conducted at 37 °C for 16 h followed by heating to 75 °C for 10 mins. The product was then visualised by gel electrophoresis, showing a band around 75 base pairs (expected 73 base pairs), before being purified according to the general ethanol precipitation procedure. The resulting pellet was reconstituted in water (12 µL) for the Suzuki-Miyaura step.

The double-stranded DNA was reacted with (4-(ethoxycarbonyl)phenyl)boronic acid according to the general on-DNA Suzuki-Miyaura Cross-Coupling procedure to give DNA product (4.7 nmol).

To the DNA product (4.7 nmol in 50 µL H_2O) was added $LiOH.H_2O$ (50 µL, 0.5 M in H_2O) and the reaction mixture stirred at room temperature at 800 rpm for 30 mins. The solution was added to an Amicon

Ultra-0.5 Centrifugal 10 kDa Cut-off Filter Unit, DEPC-treated water (200 µL was added and the solution centrifuged at room temperature for 10 mins. DEPC-treated water (200 µL) was added to the filter and the mixture centrifuged at room temperature for 10 mins. This was repeated 2 more times, then the filter was inverted and centrifuged at 4,300 rpm at room temperature for 2 mins to yield the aqueous DNA product (4.6 nmol).

Figure S207: Gel electrophoresis visualisation of 2nd ligation showing a band around 75 base pairs in length (expected 73 base pairs).

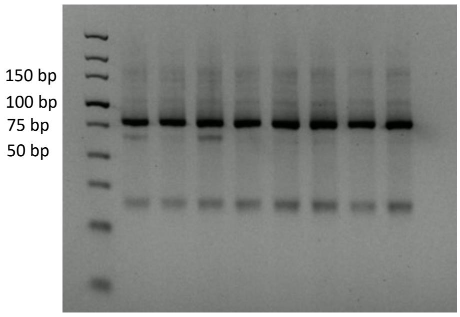

Cycle 3:

To individual Applied Biosystems™ MicroAmp® 96-well Reaction Plate wells was added DNA sequences cycle 2 product (10 µL, 0.92 nmol) and OH3BB3P2 (TACG ATAGAGGGTC TGACCTCAACTACATGGTCTACA) (10 µL, 1 nmol), which were combined with ATP (2 µL, 10 mM in H_2O), PNK reaction buffer (2 µL, 500 mM Tris-HCl [pH 7.6 at 25 °C], 100 mM $MgCl_2$, 50 mM DTT, 1 mM spermidine), T4 Polynucleotide Kinase (1 µL, 10 U/µL) and DEPC-treated water (5 µL). The reaction was conducted at 37 °C for 1 h, followed by heating to 75 °C for 10 mins. DNA was used in subsequent ligation without further purification.

18.4 µL of the combined phosphorylation reaction of OH3BB3P2 and 20 µL of the combined phosphorylation reaction of the cycle 2 product (0.92 nmol) were added to non-phosphorylated P2'BB3' (TGTAGACCATGTAGTTGAGGTCA GTAAGACCTC) (9.2 µL, 0.92 nmol). To the DNA sequences was added 10X T4 DNA ligase buffer (9 µL, 400 mM Tris-HCl, 100 mM $MgCl_2$, 100 mM DTT, 5 mM ATP), T4 DNA ligase (1.5 µL, 30 U/µL) and DEPC-treated water (31.9 µL). The reactions were conducted at 37 °C for 16 h followed by heating to 75 °C for 10 mins. The product was then visualised by gel electrophoresis, showing a band just above 100 base pairs (expected 110 base pairs), before being purified according to the general ethanol precipitation procedure. The resulting pellet was reconstituted in water (4 µL) for the reverse amide step.

The double stranded DNA was reacted with benzylamine according to the general on-DNA reverse amide coupling procedure to give DNA product (1.65 nmol).

Figure S208: Gel electrophoresis visualisation of 3rd ligation showing a band just above 100 base pairs in length (expected 110 base pairs).

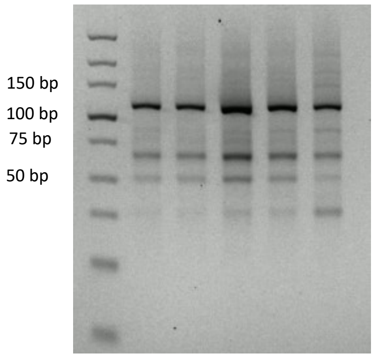

PCR Amplification and Sequencing:

PCR amplification was performed in a 200 µL PCR tube. Library DNA (1 pmol, 20 µL), combined PCR primer and reverse PCR primer (2 µL, 10 µM in H_2O), DEPC-treated H_2O (3 µL) and Applied Biosystems™ AmpliTaq Gold™ 360 Master Mix (25 µL) were combined and exposed to thermal cycling conditions consisting of 10 min at 95 °C, followed by 38 cycles of 30 s at 95 °C, 30 s at 55°C, and 1 min at 72°C, with a final extension time of 420 s at 72°C. The PCR products were cleaned up using NucleoSpin Gel and PCR Clean-up Columns for gel extraction and PCR clean up (Macherey-Nagel, Item number: 740609.250) (according to manufacturer guidelines). The PCR product was visualised by gel electrophoresis showing a band just above 150 base pairs (expected 161 base pairs). The DNA was diluted to 21 ng/µL and analysed by NGS (Genewiz, South Plainfield, NJ, USA), 78% of 86,126 corresponded to the expected sequence.

Figure S209: Gel electrophoresis visualisation of PCR product showing a band just above 150 base pairs in length (expected 161 base pairs).

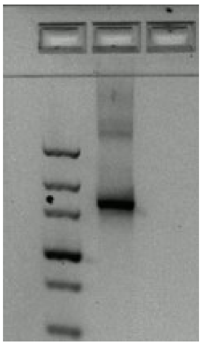

Sequences and frequencies of the top 10 most frequent reads for the substrate strand (total reads 86,126):

Sequence				Count	Relative Frequency
Library Code	BB1 Code	BB2 Code	BB3 Code		
AGTCGCGGAA	AGTGCATTCA	CCGCTAGGCT	GAGGTCTTAC	66,760	0.775
GTCGCGGAAG	GTGCATTCAC	CGCTAGGCTT	AGGTCTTACT	1,698	0.020
AGTCGCGGAA	TGCCGAATTC	CGGTGTGAAC	TTGAGTCGCG	1,416	0.016
AGTCGCGGAA	AGTGCATTCA	CCGCTAGGCT	TGCCGAATTC	773	0.009
AGTCGCGGAA	AGTGCATTCA	TGCCGAATTC	CGGTGTGAAC	675	0.008
AGTCGCGGAA	AGTGCATTCA	CCGCTAGGCT	GAGGCCTTAC	489	0.006
AGTCGCGGAA	AGTGCATTCA	CCGCTAGGCT	TGCCGCATTC	418	0.005
GAGTCGCGGA	TAGTGCATTC	ACCGCTAGGC	GGAGGTCTTA	314	0.004
ACGAATCGTA	AGGCGGCAGA	GTAGAAGCGA	ACTGATTAGA	221	0.003
AGTCTTGCCG	CAGGTCGGTG	CGGATTTGAG	GGAAGTATAG	217	0.003
